# Pooled prevalence and associated factors of ECG abnormality among type 2 diabetic patients in the last ten years: Systematic review and meta-analysis

**DOI:** 10.1371/journal.pone.0319173

**Published:** 2025-03-13

**Authors:** Mihret Getnet, Habtu Kifle Negash, Hailu Aragie, Hiwot Tezera Endale, Tseganesh Asefa, Winta Tesfaye, Yibeltal Yismaw Gela

**Affiliations:** 1 Department of Human Physiology, School of Medicine, College of Medicine and Health Sciences, University of Gondar, Gondar, Ethiopia; 2 Department of Epidemiology and Biostatistics, Institute of Public Health, College of Medicine and Health Sciences, University of Gondar, Gondar, Ethiopia; 3 Department of Human Anatomy, School of Medicine, College of Medicine and Health Sciences, University of Gondar, Gondar, Ethiopia; 4 Department of Medical Biochemistry, School of Medicine, College of Medicine and Health Sciences, University of Gondar, Gondar, Ethiopia; 5 Department of Medical Nursing, School of Nursing, College of Medicine and Health Sciences, University of Gondar, Gondar, Ethiopia; Albert Einstein College of Medicine, UNITED STATES OF AMERICA

## Abstract

**Background:**

Type 2 diabetes mellitus is a global epidemic affecting millions of individuals worldwide. It is considered a chronic metabolic disorder of impaired glucose homeostasis, associated with various long-term complications and poor prognosis of cardiovascular performance. Therefore, this systematic review aimed to determine the pooled prevalence of ECG abnormality among type 2 diabetic patients both in the hospital setting and the general population based on the existing literature.

**Methods:**

This systematic review has been conducted on the ECG abnormality of patients with Type 2 diabetes. Following the establishment of eligibility criteria, a literature search was conducted using three databases and two search engines. Included articles were then screened, critically appraised, and data extracted independently by two reviewers, and any disagreements were handled by the involvement of a third party. The quality of the included studies had been assessed using the New Castle Ottawa quality assessment scale tool. Pooled prevalence and sensitivity were determined by random effect analysis. Heterogeneity was assessed by Higgins’s *I*^2^, and its presence was alleviated by using sub-group analysis.

**Result:**

Following the identification of 32, 785 studies, 33 publications were eligible for the review with a sample size of 31, 449. The pooled prevalence of ECG abnormality among Type 2 diabetic patients was 31% (95% CI: 25, 36%). It was 26% (95% CI: 1, 51%), and 31% (95% CI: 24, 37%) in the hospital and general/ community population, respectively. Body mass index (AOR =  5.90; 95%CI: 4.96, 7.03), duration of diabetic mellitus (AOR = 9.21; 95%CI: 9.12, 9.31), and being hypertensive (AOR = 5.17; 95%CI: 4.90, 5.46), were significantly associated factors with ECG abnormality among patients with Type 2 diabetic mellitus.

**Conclusion:**

The pooled prevalence of ECG abnormality among Type 2 diabetic mellitus patients was high, while its magnitude was higher among patients attending hospital settings than in the community. Duration of diabetic mellitus, high body mass index and presence of hypertension were significant factors in this review. Moreover, we advise more longitudinal researches to determine the incidence of ECG abnormality among patients with diabetes considering time duration and sex differences.

**Ethical consideration:**

Since our study was on the review of secondary data, ethical issues are not necessary

## Introduction

Type 2 diabetes mellitus (T2DM) is a global problem reaching millions of individuals worldwide [1]. It is considered a chronic metabolic disorder of impaired glucose homeostasis, resulting in insulin resistance and prolonged hyperglycemia, various long-term complications and is associated with poor prognosis of cardiovascular performance [2]. Cardiac problems are one of the greatest causes of morbidity and mortality in T2DM due to the associated high risk of developing silent coronary artery disease, heart failure, arrhythmias, and sudden cardiac death; and impose a considerable burden on the health of the societies both in developed and developing nations [3,4]. Mortality and hospitalization rates also radically increased among individuals with both type 2 diabetes and cardiovascular disease (CVD) than in those suffering from either alone [5,6].

Mostly, these conditions arise without the classic presentation, and for this reason, early recognition and vigorous screening are needed to detect these complications as soon as possible [7]. The electrocardiogram (ECG), as a non-invasive, cost-effective method, plays a significant role in detecting abnormalities such as myocardial ischemia, arrhythmias, and ventricular hypertrophy which are frequently discovered in T2DM patients [8,9]. This routine approach is not encouraged among asymptomatic patients with no history of CVD undergoing treatment for T2DM, and the prevalence of ECG abnormalities among T2DM patients remained unrecognized [10,11]. Electrocardiographic abnormalities among these patients frequently make them present with the possibility of an unknown risk of an impending cardiovascular event with a potentially lethal result [11].

Even though ECG abnormalities are under-diagnosed, these are common among patients with type 2 diabetes mellitus and have shown a direct correlation to the incidence of major cardiovascular events [12,13]. The relationship between T2DM and cardiovascular diseases is known [14,15], still, routine ECG screening is not consistently performed among diabetic patients, which leads to delayed diagnosis and treatment of potentially life-threatening cardiovascular issues, largely due to the lack of strong clinical data and discrepancies concerning their reliability of ECG in the clinical setting [16].

A recent study showed that diabetes and cardiovascular disease in the general population account for more than 80% of mortality and morbidity in developing countries [17]. The International Diabetes Federation (IDF) indicates the most common cause of mortality and disability is CVD among people with type 2 diabetes [18]. Besides, IDF reported the trend of diabetes increasing from 8.8% in 2015 to 10.4% in 2040; which has social, financial and developmental implications [19]. Over the last decades, the worsening of overweight and type 2 diabetic mellitus affecting societies may be one of the major explanations for the increasing trend of ECG abnormalities [14,20]. However, the details regarding the prevalence and types of known ECG abnormalities among T2DM patients across the general population remain uncertain [21].

Previous studies identified that duration of diabetic mellitus, hypertension, advanced age, glucose level over 200 mg/dl and smoking were significantly associated with ECG changes among Type-2 DM patients [15,22]. This study will help to understand the various aspects from this point of view, the actual issues with ECG changes among T2DM patients, along with scientific evidence to develop distinct and targeted strategies to reduce the high cardiovascular risk for Type 2 DM patients. Given the above, determining the pooled prevalence of ECG abnormality and clinical consequences among T2DM patients gives an insight into early detection and preventive measures for cardiovascular complications in this high-risk population.

Studying ECG abnormalities among Type 2 diabetic mellitus patients has significant implications for national and international health strategies, such as Sustainable Development Goal-3 (SDG-3) [23], which focuses on ensuring healthy lives and promoting well-being for people of all ages. Hence, early detection and management of ECG abnormalities in diabetic patients contributed to reducing the burden of non-communicable disease, enhancing health care systems and improving long-term outcomes [24]. This approach aids in optimizing resource allocation, advancing universal health coverage and promoting global equity in managing diabetes-related complications and addressing health disparities across populations.

Furthermore, some studies focusing solely on ventricular hypertrophy were conducted exclusively in diabetic patients, making them unrepresentative of the broader population [25]. Therefore, we reviewed the existing literature to estimate the prevalence of ECG abnormality among type 2 diabetes patients both in the hospital and the community setting.

## Methods and materials

### Protocol and registration

A protocol adhering to the Preferred Reporting Items for Systematic Review and Meta-Analysis Protocols (PRISMA-P) statement was developed. It outlines the detailed plans for conducting the systematic review and meta-analysis on electrocardiographic abnormalities in patients with type 2 diabetic mellitus. This protocol has been registered on PROSPERO under the reference number of CRD42024597177.

### Eligibility criteria

Studies that involve non-human subjects, duplicates, abstract-only papers (editorials), case reports, case series, systematic reviews and those without full text were not included.

### Inclusion criteria

Using the Population, Index/ Reference Test, and Diseases (PIRD) approach, the included articles meet the following criteria: Articles on ECG abnormality with all people regardless of their age, gender, country, or race, Studies where ECG was performed on type 2 DM patients, studies which provide sufficient data to estimate prevalence of ECG abnormality, studies that report at least one wave change in ECG, original research articles, and articles published in or translated into English language, regardless of publication date or study design.

The selection of publications and data extraction were done independently by two reviewers (YYG and HKN). With the help of a third author (MG), differences have been resolved by consensus.

### Information sources and searches

A comprehensive search was conducted using three electronic databases and two search engines were used to identify relevant articles. These databases used for searching include PubMed, Hinari, and Cochrane library, all accessed on 29 August 2024. Additional, searches were performed using Google Scholar on 29 August 2024 and Google search engine on 30 August 2024. The search restricted to studies published between 2015 and 2024.

### Searching strategy

Keywords: Electrocardiography, abnormality, Type-2 diabetic mellitus and patients were extracted from the study question. Entry terms were searched for all the keywords from the MeSH word browser to identify the synonyms of the words. For electrocardiography, synonyms like ECG, EKG, and Electrocardiograph were obtained; for abnormality, the synonym obtained was changes; for Type-2 diabetic mellitus no synonym was obtained; and for patients, the synonyms were client, clients. Finally, our searching for all databases became:

(“ECG”) OR (“EKG”) OR (“Electrocardiograph”) OR (“Electrocardiography”) AND (“Abnormality”) OR (“Changes”) AND (“Type-2 diabetic mellitus”) AND (“Patient*”) OR (“Client*”). Various searching techniques were applied according to the databases and search engines to facilitate the search process. Phrases were enclosed by quotation marks to be treated as a single term; truncation was used to capture all variations of a specific word, and Boolean operators were used to connect keywords, synonyms and Medical Subject Headings (MeSH) words. The keyword search targeted the titles and abstracts of articles.

As a result, we retrieved a total of 32, 785 articles and subsequently added to the EndNote library. The majority of these articles (n = 27, 200) were sourced from Google Scholar (Table 1).

**Table 1:** Searching strategy electrocardiographic abnormality among Type 2 diabetic mellitus patients.

PubMed				
Mesh heading	Entry term	Combination	Finding result	Searching Date
Electrocardiography	12-Lead ECG 12-Lead EKG 12-Lead Electrocardiography ECG EKG Electrocardiogram Electrocardiograph	(((“12-Lead ECG”[Title/Abstract] OR “12-Lead EKG”[Title/Abstract] OR “12-Lead Electrocardiography”[Title/Abstract] OR “ECG”[Title/Abstract] OR “EKG”[Title/Abstract] OR “Electrocardiogram”[Title/Abstract] OR “Electrocardiograph”[Title/Abstract]) AND (“Abnormality”[Title/Abstract] OR “Changes”[Title/Abstract])) AND (“Type 2 diabetic mellitus”[Title/Abstract])) AND (“patients”[Title/Abstract] OR “clients”[Title/Abstract])	272	8/29/2024
Abnormality	Abnormal, change			
Type 2 diabetic mellitus	Type 2 diabetic mellitus			
Patients	Patients, clients			
Google scholar				
Electrocardiographic	ECG, EKG, Electrocardiograph, Electrocardiography	“ECG” OR “EKG” OR “Electrocardiograph” OR “Electrocardiography” AND “Abnormality” OR “Changes” AND “Type-2 diabetic mellitus” AND “Patient” OR “Patients” OR “Client”	27, 200	8/29/2024
Abnormality	Abnormality, changes			
T2 diabetic mellitus	T2 diabetic mellitus			
patients	Patient, patients, client			
Cochrane				
Electrocardiographic	12-Lead ECG 12-Lead EKG 12-Lead Electrocardiography ECG EKG Electrocardiogram Electrocardiograph	“12-Lead ECG” OR “12-Lead EKG” OR “12-Lead Electrocardiography” OR “ECG” OR “EKG” OR “Electrocardiogram” OR “Electrocardiograph” in Title Abstract Keyword AND “abnormality” OR “changes” in Title Abstract Keyword AND “Type 2 diabetic mellitus” in Title Abstract Keyword AND “patient” OR “patients” OR “clients” in Title Abstract Keyword	5224	8/29/2024
Abnormality	Abnormality, changes			
Type 2 diabetic mellitus	Type 2 diabetic mellitus			
Patients	Patient, patients, client			
Hinari				
Electrocardiographic abnormality Type 2 diabetic mellitus patients	ECG change ECG variation	(TitleCombined: (Electrocardiographic)) AND (TitleCombined: (abnormality)) AND (TitleCombined: (Type 2 diabetic mellitus)) AND (TitleCombined: (Patients))	9	8/29/2024
Google				
Electrocardiographic abnormalities among Type 2 diabetic mellitus patients			80	8/30/2024

### Study selection

Two authors (HTE and HA) independently reviewed the publications, after exporting all search results to EndNote., and the initially identified 301 duplicate articles were eliminated. The remaining articles, free of duplication, undergo further screening. About 32,430 irrelevant articles were eliminated after the title was evaluated in accordance with the eligibility criteria. Subsequently, abstracts were assessed leading to the removal of 24 articles that do not align with the research question. Articles available only as abstract and those inaccessible were excluded from the analysis.

**Fig 1:**
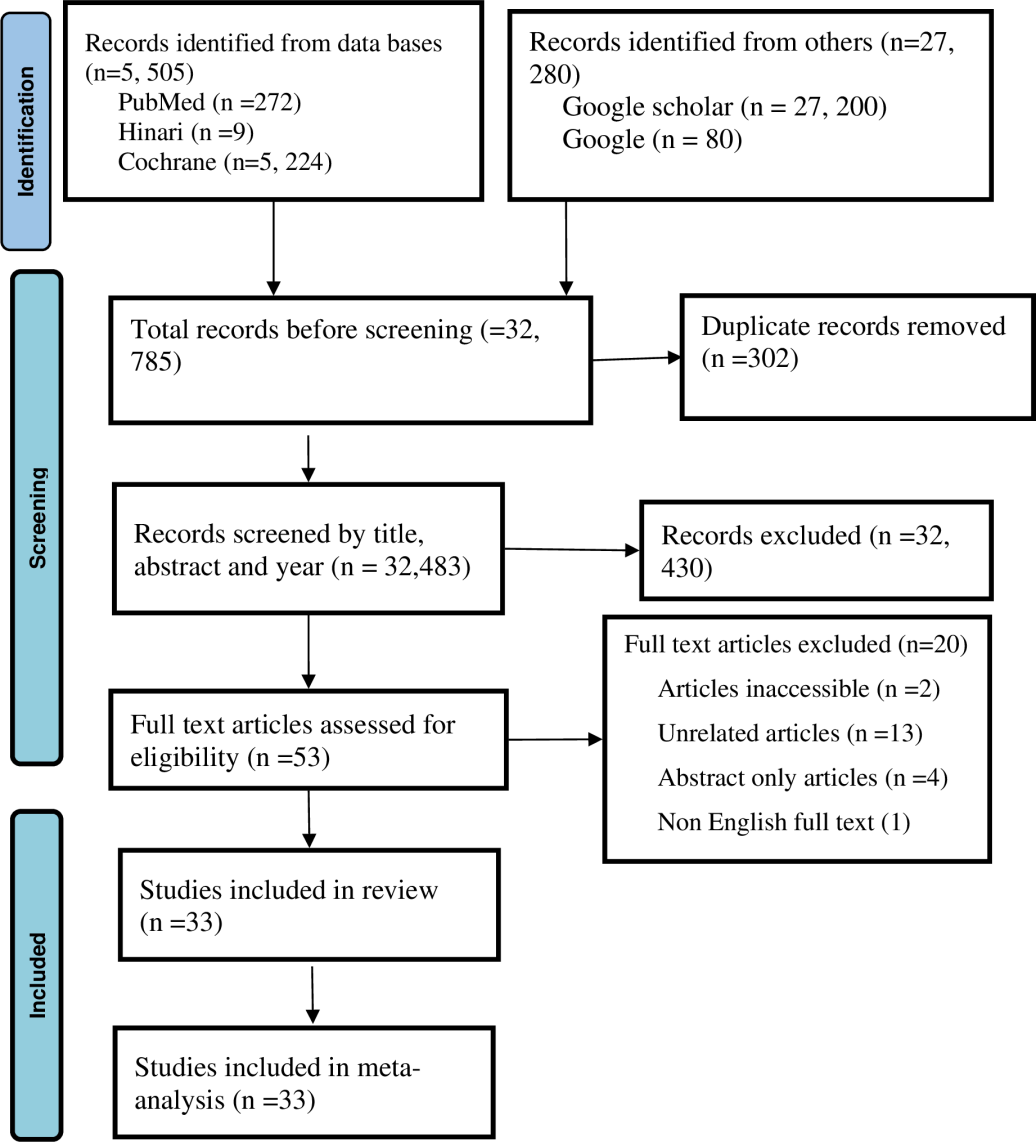
PRISMA flow diagram showing the mechanism of extracting related articles.

The freely available full-text articles were downloaded, while the remaining ones were sourced from various research websites. Following a through full-text review, 13 articles were excluded due to a lack of relevance to the research question. Ultimately, 33 articles met the inclusion criteria and were deemed appropriate to the systematic review and subsequent meta-analysis. The study selection process is summarized below in the PRISMA flow diagram (Fig 1).

### Data collection process

The data was extracted from included studies by using previously prepared data extraction format to make the extraction process easy. To evaluate the level of agreement between the reviewers, initial data extraction was performed on a sample of randomly selected studies. Data from the full text were compiled into an Excel sheet. After both authors completed their data extraction, they compared and contrasted their results. For missing data, attempts were made to contact authors via email; however, as no responses were received, the studies were retained as long as they met the eligibility requirements.

### Data items

Since this is a systematic review and meta-analysis of electrocardiographic abnormality, the mnemonic for the research question is CoCoPop. The ‘people’ in the study are all people with presumptive Type-2 diabetic mellitus.

The following data were collected for all included study: Study information (first author’s name, years of publication, publication journal (if published)); Study characteristics (study design, country and region); features of Population (sample size, age, DM duration, electrocardiographic measurements and other correlated factors); and Participant selection (continuous or random sampling).

### Risk of bias and applicability

The Newcastle Ottawa Quality Assessment Scale (NOQAS) tool was used to critically evaluate the risk of bias of relevant studies. It describes four domains and nine items and each of the items scored as “yes” or “no” by using ‘*’ and ‘-’, respectively.

By using Excel, the risk of bias based on the four NOQAS tool items and three of the four items’ applicability issues were examined. These findings are shown in (Table 2) below.

**Table 2:** New castle Ottawa Quality assessment of included studies.

Study	Year	Clear aim	Representativeness of the case	Sample size	Non response rate	Ascertainment of screening	Potential confounders investigated	Comparability	Outcome assessment	Statistical test	Overall	Total
Roy etal	2021	*	*	*	*	*	–	*	*	*	8	Low risk
Ezeude etal	2024	*	*	*	*	*	–	*	*	–	7	Medium risk
CM(Malachy) etal	2023	*	–		*	*	*	*	*	*	7	Medium risk
Bayramoglu etal	2017	*	*	*	*	*	–	*	*	*	8	Low risk
Ostrowska etal	2016	*	*	*	*	*	*	*	*	*	9	Low risk
Kumar etal	2022	*	*	*	*	–	*	*	*	*	8	Low risk
Khaire et al	2018	*	–		*	*	*	*	*	–	6	Medium risk
Jani et al	2015	*	*	*	*	*	*	*	*	*	9	Low risk
Bosone et al	2016	*	*	*	*	*	*	*	*	*	9	Low risk
Roy etal	2020	*	*	*	–	*	*	*	*	–	7	Medium risk
Gokhale et al	2020	*	–		*	*	–	*	*	–	5	High risk
Bhatt et al	2016	*	*	*	*	*	–	*	*	*	8	Low risk
Gregors et al	2022	*	*	*	*	*	*	*	*	*	9	Low risk
Desai et al	2018	*	–	–	*	*	–	*	*	–	5	High risk
Sinamaw et al	2022	*	*	*	*	*	*	*	*	*	9	Low risk
Cha	2022	*	*	*	*	*	*	*	*	*	9	Low risk
Kobayashi et al	2018	*	*	*	*	*	*	*	*	*	9	Low risk
Khanal et al	2023	*	–	–	*	*	*	*	*	*	7	Medium risk
Zhou et al	2017	*	–	–	*	*	*	*	*	*	7	Medium risk
Gupta et al	2017	*	–	–	*	*	–	*	–	–	4	High risk
Jorgensen et al	2016	*	*	*	*	*	*	*	*	*	9	Low risk
Nijpels et al	2021	*	–	–	*	*	–	*	*	–	5	High risk
Ndour et al	2017	*	–	–	*	*	–	*	*	–	5	High risk
Sertbas et al	2017	*	*	*	*	*	–	*	*	–	7	Medium risk
Fatih Kuzu	2018	*	*	*	*	*	*	*	*	*	9	Low risk
Lu et al	2017	*	*	*	*	*	*	*	*	*	9	Good
Solanki et al	2017	*	–	–	*	*	*	*	*	*	7	Medium risk
Ninkovic et al	2016	*	*	*	*	*	*	*	*	*	9	Low risk
Bedane et al	2021	*	*	*	*	*	*	*	*	*	9	Low risk
Sinamaw et al	2022	*	*	*	*	*	*	*	*	*	9	Low risk
Saffar et al	2024	*	*	*	*	*	*	*	*	*	9	Low risk
Chang et al	2021	*	–	–	*	*	*	*	*	*	7	Medium risk
Harms et al	2023	*	*	*	*	*	*	*	*	*	9	Low risk

The following questions were employed to identify potential issues the study’s design, conduct and analysis that may induce bias or raise doubt regarding the generalizability of the findings. The signaling questions used are as follows [26]:

A Is a case definition for ECG abnormality clearly applied in the study?
B Are the included study participants truly representative of the average in the target population (type 2 diabetes patients from the community, and/or hospital center)?
C Is the sample size adequately justified and sufficient?
D Is the response rate assessed and satisfactory (>70%), and comparable characteristics assessed?
E Is the instrument used to measure ECG abnormality was valid and reliable?
F Does the study consider any possible confounder(s)?
G Are the study subjects in different groups are comparable by design or analysis?
H Is it independent blind assessment or record linkage used to assess outcome?
I Were the statistical tests used to analyze the data are clearly described and appropriate, including confidence intervals and probability level?

Each signaling question was rated as having low, medium and high risk of bias. Studies were classified as having an overall low risk of bias if ≤1 question had a risk of bias, medium risk of bias if 2–3 questions had a high risk bias, and a high risk of bias if more than 3 questions had a risk of bias [25].

### Data synthesis and analysis

Different softwares such as Excel, R-software version 4.2.1, and STATA version 17 used for data analysis. The prevalence rate for each study, along with their respective 95% confidence intervals (CIs) was calculated for all studies included for this review. Meta-analysis was performed using STATA version 17 software to calculate the pooled prevalence of ECG abnormality among Type-2 diabetic mellitus patients. Since a random-effects model better accounts for between-study heterogeneity than a fixed-effects model, it was used to generate pooled estimates (along with the corresponding 95% CI) of the logit-transformed prevalence data. Heterogeneity among studies was evaluated using the *I*^2^ statistic. The *I*^2^- statistic indicates the percentage of variability in effect estimates across studies that are due to heterogeneity rather than chance, with values ranging from 0% and 100%. Generally, *I*^2^ values of 25%, 50%, and 75% correspond to low, moderate and high heterogeneity, respectively [27]. For all included studies, as well as for studies including the general community and hospital population, the pooled prevalence estimate was computed. A forest plot was used to display the results of the computation of the pooled prevalence estimate for all included studies and visual inspection of a funnel plot used to evaluate the presence of publication bias. To further quantify the bias, Begg’s and Egger’s tests were employed [28,29]. Statistical significance is defined as p-value of <  0.05.

### Additional analysis

To investigate the source of heterogeneity, subgroup analysis was done by categorizing studies based on sub-regions and quality of the study using the Meta package of R-software. Moreover, outliers were detected using the find.outlier function [29]. By selecting a study with an extreme effect size, the pooled effect and heterogeneity were looked at after the outliers had been eliminated.

Using R software “Influence Analysis” function, the leave-one-out method was used to quantify influential studies. The pooled results of the meta-analysis were recalculated by leaving-out one article each time to determine the influential one.

## Results

### Searching results

A total of 32, 785 unique studies were identified from online databases and search engines without the consideration of year or place of publication. After duplicate elimination, 32, 483 articles were screened, and these articles before 2015, unrelated articles/titles, and abstract-only articles were excluded (n = 32, 450). With a sample size of 31,449, the remaining 33 publications were part of the systematic review.

### Characteristics of included studies

The characteristics of all eligible articles encompassing 33 datasets focused on the use of Electrocardiography to measure cardiac wave changes among patients with type 2 diabetic mellitus. From the total studies, sixteen (48.48%) were from Asia, 8(24.24%) from Europe and 6 (18.18%) from Africa (Table 1). The sample sizes of included studies ranged from 100 to 11993, and the sampling for about 21 of the included studies was done consecutively (Table 3).

**Table 3:** General characteristics and quality assessment of selected studies on ECG abnormality (n = 31, 449).

Study	Year of Study	Country	Country region	Study setting	Quality of Studies	Patient selection	Age group	Sample size	Event	Proportion
Roy et al [53]	2021	India	Asia	Hospital	Low risk	Consquetive	51.04 + -9.38	100	21	0.21
Ezeude et al [32]	2024	Nigeria	Africa	DM Clinic	Medium risk	Consquetive	58.43 ± 12.85	128	80	0.634
CM(Malachy) et al [54]	2023	Nigeria	Africa	Hospital	Medium risk	Consquetive	58.43 ± 12.85	128	58	0.453
Bayramoglu et al [55]	2017	Turkey	Asia	Hospital	Low risk	Consquetive	56.3 ± 6.7	178	50	0.28
Ostrowska et al [56]	2016	Poland	Europe	DM Clinic	Low risk	Simple random	65 ± 11	1001	200	0.2
Kumar et al [9]	2022	India	Asia	Hospital	Low risk	Simple random	20-50	270	68	0.25
Khaire et al [57]	2018	Maharashtra	Asia	Hospital	Medium risk	Consquetive	30-70	100	40	0.4
Jani et al [58]	2015	Macedonia	Europe	Hospital	Low risk	Simple random	49.9 ± 31	300	122	0.406
Bosone et al [59]	2016	Italy	Europe	Hospital	Low risk	Consquetive	65-75	243	51	0.209
Roy etal [60]	2020	India	Asia	Hospital	Medium risk	Simple random	53.78 ± 11.98	324	15	0.046
Gokhale et al [61]	2020	India	Asia	Hospital	High risk	Consquetive	18-80	100	44	0.44
Bhatt et al [62]	2016	USA	N.America	Research Center	Low risk	Simple random	35-75	1671	255	0.153
Gregors et al [63]	2022	Denmark	Europe	DM Clinic	Low risk	Consquetive	61-74	722	175	0.24
Desai et al [64]	2018	Kolhapur	Asia	Hospital	High risk	Consquetive		200	50	0.25
Sinamaw et al [21]	2022	Ethiopia	Africa	Hospital	Low risk	Simple random	28-80	259	56	0.217
Cha [65]	2022	Korea	Asia	DM Clinic	Low risk	Simple random	56.3 ± 10.6	411	90	0.219
Kobayashi et al [66]	2018	Japan	Asia	Hospital	Low risk	Consquetive	60 ± 13	219	76	0.35
Khanal et al [67]	2023	Nepal	Asia	DM clinic	Medium risk	Simple random	54.3 ± 10.8	345	165	0.478
Zhou et al [68]	2017	Albury-Wodonga	Australia	Community	Medium risk	Consquetive	69.76 ± 0.94	273	52	0.1905
Gupta et al [69]	2017	India	Asia	Hospital	High risk	Simple random	50.3 ± 11.90	100	26	0.26
Jorgensen et al [70]	2016	Denmark	Europe	Hospital	Low risk	Consquetive	67.6 ± 12.7	1030	257	0.261
Nijpels et al [71]	2021	Netherlands	Europe	Hospital	High risk	Simple random	68	180	31	0.172
Ndour et al [72]	2017	Senegal	Africa	Hospital	High risk	Consquetive	58.3	100	50	0.33
Sertbas et al [73]	2017	Turkey	Asia	Hospital	Medium risk	Consquetive	56 ± 9.95	275	58	0.21
Fatih Kuzu [74]	2018	Turkey	Asia	Hospital	Low risk	Consquetive	46.5 ± 6.2	200	72	0.36
Lu et al [75]	2017	USA	N.America	Hospital	Low risk	Consquetive	76.2 ± 0.9	154	79	0.513
Solanki et al [76]	2017	India	Asia	Hospital	Medium risk	Consquetive	56	199	40	0.2
Ninkovic et al [77]	2016	Serbia	Europe	Hospital	Low risk	Consquetive	60.4 ± 8.1	501	221	0.441
Bedane et al [31]	2021	Ethiopia	Africa	Hospital	Low risk	Simple random		344	209	0.61
Sinamaw et al [78]	2022	Ethiopia	Africa	Hospital	Low risk	Simple random		258	116	0.45
Saffar et al [79]	2024	Iran	Asia	Community	Low risk	Consquetive	47.45 ± 8.17	9035	1246	0.1379
Chang et al [80]	2021	China	Asia	Hospital	Medium risk	Consquetive	59.2 ± 9.7	108	11	0.102
Harms et al [81]	2023	Netherlands	Europe	Community	Low risk	Consquetive	62.4 ± 12.1	11993	5445	0.454

### Risk of bias and applicability

The included studies were generally considered high quality, as assed using the widely accepted Newcastle Ottawa Quality Assessment Scale. This scale evaluates the risk of biases affecting study applicability through 9 items and four domains to provide accurate bias assessment.

Based on our quality assessment, 58% of the studies have a low risk of bias, 27% medium risk and only 15% of them have high risk bias (Table 4).

**Table 4:** Sub-group analysis of electrocardiographic analysis among Type 2 diabetic mellitus patients.

Variable	Category	No of studies	Pooled (Random effects) (95%_CI)	I^2^	p-value
Continent	Asia	16	26% (20, 31)	95.7%	0.000
	Africa	6	45% (30, 59)	95.5%	0.000
	Europe	8	30%(20, 40)	99%	0.000
	N. America	2	33% (20, 68)	98.7%	0.000
	Australia	1	19%(14, 24)	0.00	0.000
Patient_ selection	Random_ selection	12	29% (20, 37)	98.4%	0.000
	Consecutive	21	32% (24, 40)	99.4%	0.000
Setting	Hospital	24	31% (24, 37)	97.1%	0.000
	General population	3	26% (1, 51)	99.9%	0.000
	DM clinic	5	35%(23, 46)	97.7%	0.000
	Research Center	1	15%(14, 17)	0.00	0.000
Quality_ Assessment	Low_ bias	19	31% (24, 39)	99.5%	0.000
	Medium_ bias	9	30% (17, 42)	98.2%	0.000
	High_ bias	5	28% (20, 37)	84.1%	0.000

Of the 33 studies analysed, 30 reported the age of their participants. The mean age ranged from 46.5 ± 6.2 years in an Asian cohort to 76.2 ± 0.9 years in an American cross-sectional study. Duration of type 2 diabetes was reported in 11 of the 33 studies and ranged from new-onset diabetes to a mean duration of more than 20 years.

### Prevalence of electrocardiographic abnormality

**Fig 2:**
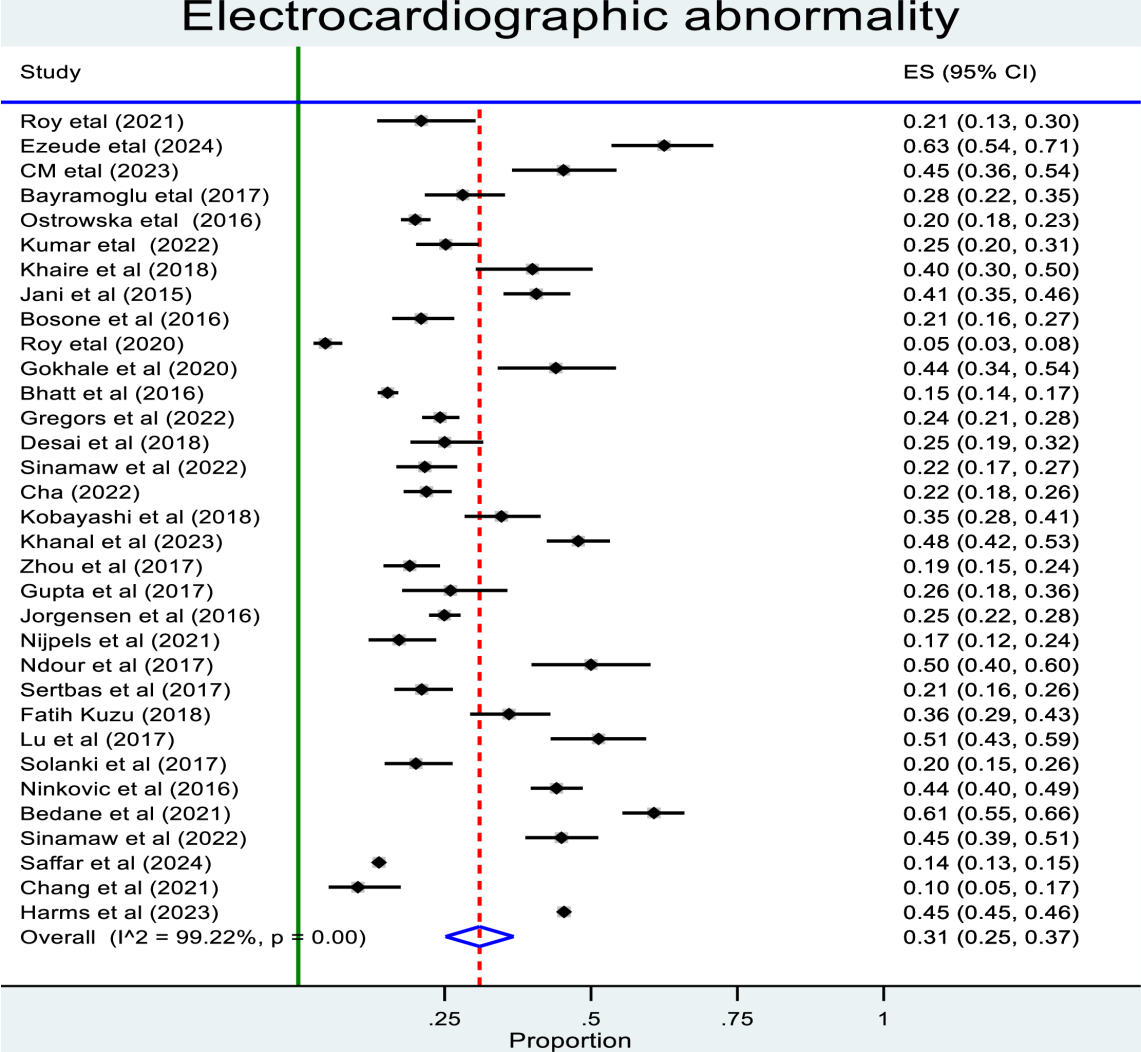
Forest plot showing random effect magnitude of individual studies.

All of the 33 studies reported the number of patients with ECG records, pooled prevalence estimates for ECG abnormality are presented for all of the included studies was 31% (95% CI: 25%–36%) (Fig 2). Estimates ranged from 1% to 51% within the community and from 24% to 37% in the hospital setting and there was a high level of study heterogeneity (in the general population: *p* <  0.001, *I*^2^ =  99.9%; hospital population: *p* <  0.001, *I*^2^ =  97.1%).

### Sub-group analysis

**Fig 3:**
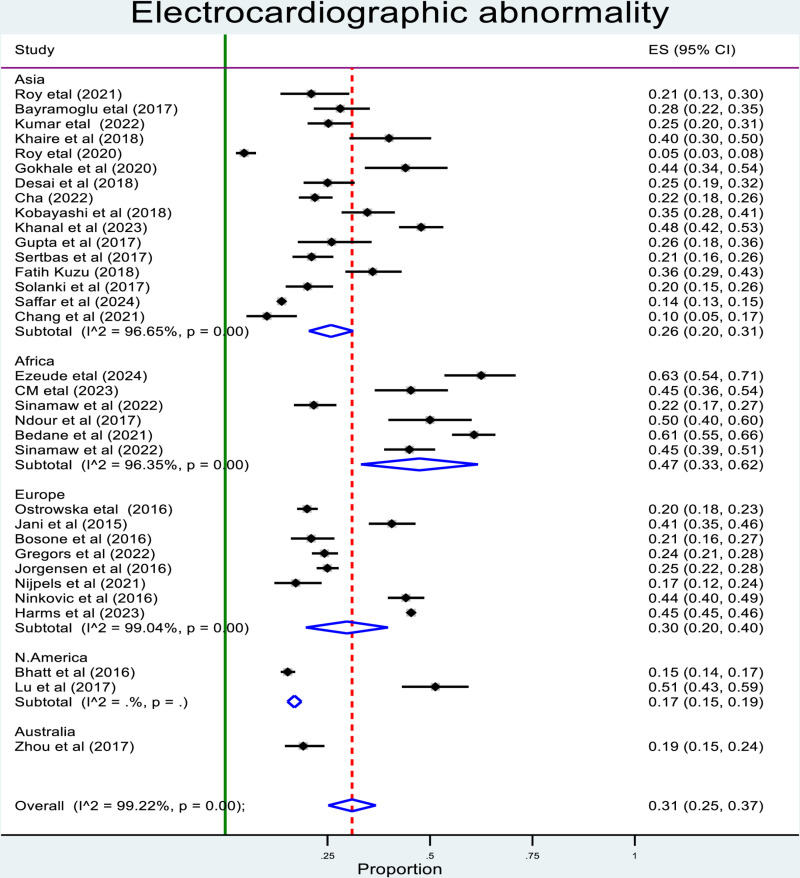
Sub-group analysis Electrocardiographic abnormality by sub-regions/ continent.

As indicated above on the forest plot (Fig 2), heterogeneity is high at I^2^ = 99.22. Therefore, for the management of heterogeneity, we have performed subgroup analysis by continent (Fig 3), patient selection, study setting and quality assessment. Even though the analysis is performed, I^2^ remains high as shown (Table 4).

### Publication bias

**Fig 4:**
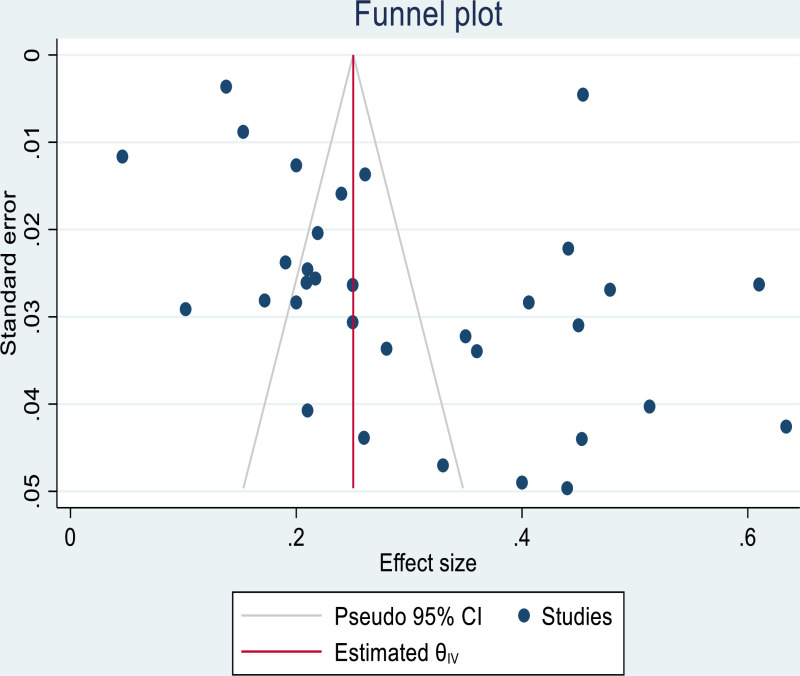
Funnel plot showing the results of publication bias electrocardiographic abnormality.

Publication bias refers to the tendency of studies with unfavorable results to be less likely to be published, potentially leading to a biased sample in the literature. If a publication bias occurs, then the published literature is a biased sample of all included studies and any meta-analysis based on it will be correspondingly biased. Funnel plot used for visual inspection of publication bias. Furthermore, on the Begg’s and Egger’s tests, there is an evidence of publication bias (P value = 0.013, and 0.0084, respectively). Since the p-value of the slope is significant, this indicates that the datasets in the study are asymmetric (Fig 4). Furthermore, we have conducted a trim and fill analysis.

### Trim and fill analysis

**Fig 5:**
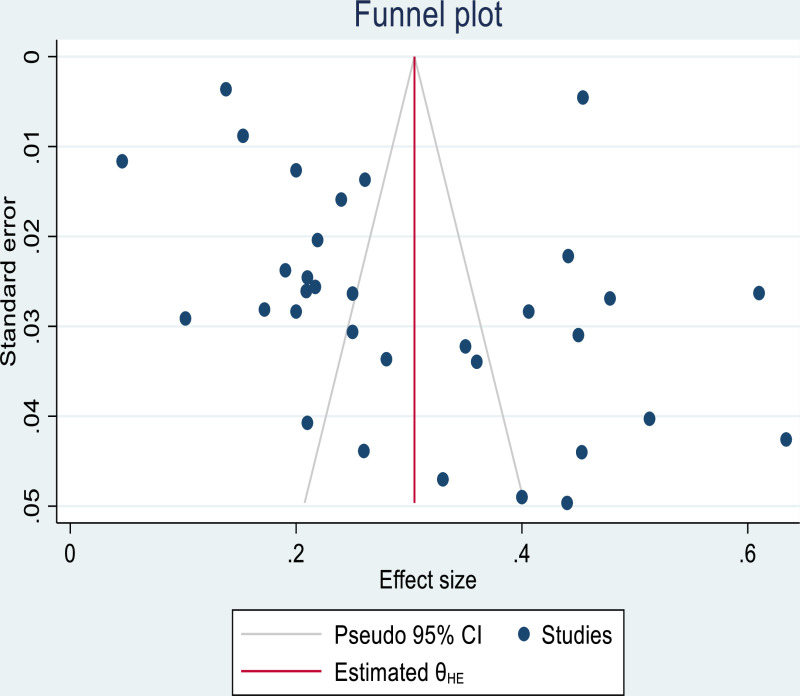
Shows trim and fill analysis electrocardiographic abnormality among Type 2 diabetic mellitus patients.

This analysis used a two-step, funnel plot-based method designed to detect and adjust for publication bias. Even though, this is performed to exclude small studies in order to have a symmetrical plot and then estimate an adjusted summary effect considering only the larger studies, the outcome of trim and fill analysis, revealed that no study is added or removed in order to readjust the publication bias (Fig 5).

**Fig 6:**
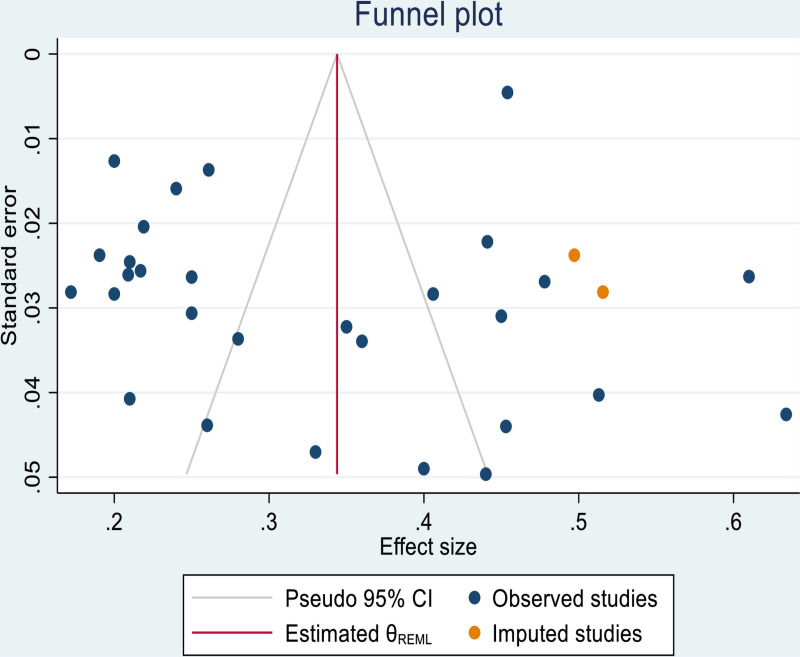
Funnel plot shows the adjusted effective size of electrocardiographic abnormality.

As illustrated above the trim and fill analysis could not handle the publication bias, we performed a leave-one-out analysis followed by a trim and trim meta-analysis after excluding the four small studies. Based on this, we got Egger’s test not significant at p = 0.1274, and the considered studies are valuable/ free from publication bias. Moreover, the final funnel plot demonstrates as follows by adding two studies (Table 5), on right side of the funnel plot (Fig 6).

**Table 5:** Shows imputed studies to handle publication bias followed by leave one out analysis.

		Number of studies = 31 Observed = 29 Imputed = 2
Studies	Effect size	[95% conf.interval]
Observed	0.332	0.2840.380
Observed+ Imputed	0.344	0.2970.391

### Sensitivity analysis

**Fig 7:**
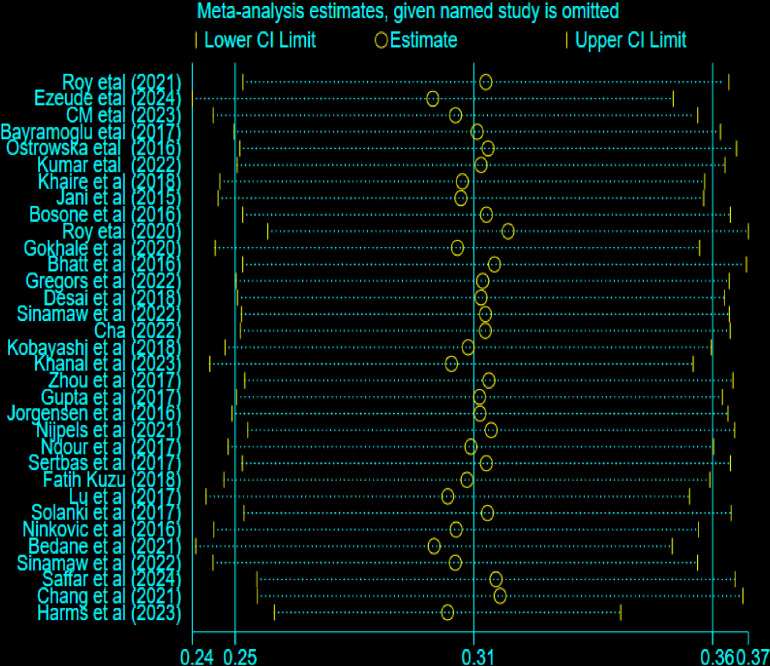
Shows sensitivity analysis of electrocardiographic abnormality among Type 2 diabetic mellitus patients.

The sensitivity analysis is done to determine the reliability of the included studies and which study more influences the overall estimated output. Based on the findings from our review, Ezeude et al (2024), Bedane et al (2021) and Bhatt et al (2016), highly affect the precision of the overall estimated effective size. This indicates the potential source of heterogeneity comes from these studies (Fig 7).

### Factors associated with ECG abnormality among type 2 diabetic patients

**Fig 8:**
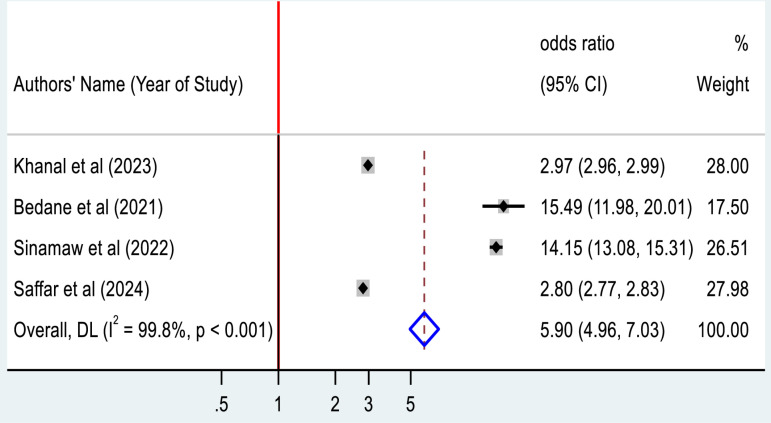
Forest plot for the association between body mass index and ECG abnormality among Type 2 diabetic mellitus patients.

In our review some factors were significantly associated with ECG abnormalities among patients with Type 2 diabetic mellitus and were pooled quantitatively. Four studies indicate that an increase in body mass index of study participants is significantly associated with ECG abnormality. Our pooled data indicated that patients with a higher body max index (BMI) was nearly 6 times more likely to have ECG abnormality than their counterparts (AOR =  5.90; 95%CI: 4.96, 7.03) (Fig 8).

**Fig 9:**
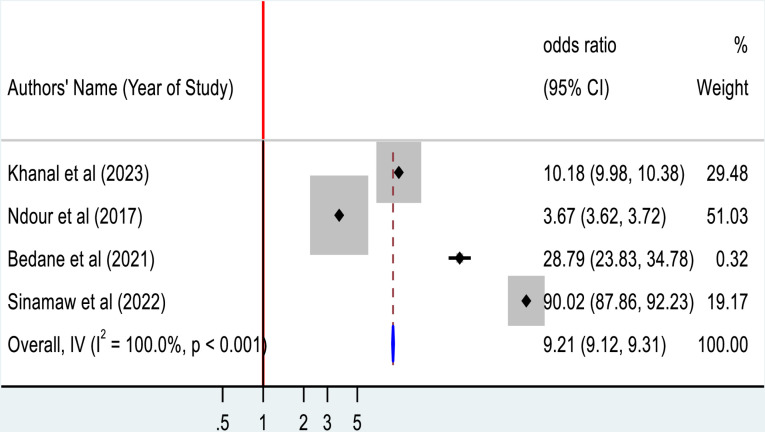
Forest plot for the association between duration of diabetes and ECG abnormality among Type 2 diabetic mellitus patients.

Duration of diabetic mellitus was assessed in four studies. The overall pooled estimate shows that the more prolonged duration of diabetes is highly associated with the development of ECG abnormality among Type 2 diabetic mellitus patients (AOR = 9.21; 95%CI: 9.12, 9.31) (Fig 9).

**Fig 10:**
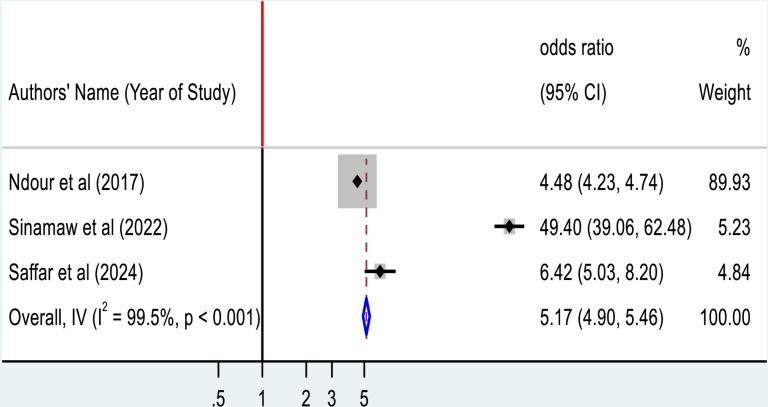
Forest plot for the association between hypertension and ECG abnormality among Type 2 diabetic mellitus patients.

As shown below (Fig 10), three studies assessed the presence of hypertension among Type 2 diabetic patients and its association with ECG abnormality. Our pooled estimate revealed that there is a statistically significant association between being hypertensive and ECG abnormality among diabetic patients (AOR = 5.17; 95%CI: 4.90, 5.46).

## Discussion

Type 2 diabetic mellitus is increasing dramatically and currently, it is a major health problem globally. Unhealthy feeding habits and a sedentary lifestyle causes an increase in body mass index and fasting plasma glucose levels have been responsible for the incidence and prevalence of Type 2 diabetic mellitus. Unexpectedly, Electrocardiographic abnormality has become common among patients with Type 2 Diabetic mellitus and our systematic review provides pooled estimates of the prevalence of ECG abnormality among type 2 diabetes patients and correlated factors of this public health problem.

The finding from this review, however, demonstrates a wide variation in the prevalence of electrocardiographic abnormalities among type 2 diabetes patients across the included studies in different countries and therefore the interpretation of pooled prevalence of each country to be with caution. Only three from 33 studies provided prevalence estimates of ECG abnormalities among type 2 diabetes patients; among 21, 646 type 2 diabetes patients the screening was taken from the general population within the community, the prevalence of ECG abnormality was 26% (95% CI: 1%–51%), and in majority of study conducted from a hospital setting among 5, 870 type 2 diabetes patients with pooled prevalence 31% (95% CI: 24%–37%). It was consistent with a study in Netherland 29.1% [30], but lower than another study in Ethiopia, which declared a prevalence of 60% [31] and a review study reported as 46% [25]. This review shows the pooled prevalence estimates of ECG abnormality in type 2 diabetes in the hospital setting was higher compared to community dwellers. This is due to the fact that early electrocardiographic screening within the community is difficult because of budget constraints to conduct it in resource limited-countries [32]. Besides, limited financial support, diabetic patients who visit the hospital setting can access better healthcare service and optimal intervention, while ECG abnormality within the community remains unknown [33].

Unfortunately, there were differences in the magnitude of ECG abnormality across the world with incidence of Type 2 Diabetic mellitus and future perspectives [34], and the prevalence varies across the countries of the included studies. The highest prevalence of ECG abnormality among Type 2 diabetic mellitus patients was observed in Nigeria 63% (95%CI: 55, 77) and the lowest prevalence in China 10% (95% CI: 4, 16). The high prevalence of ECG abnormality indicates that there is a high prevalence of cardiac abnormality in Nigeria than in China. The possible reason for this discrepancy may be in developed countries like China, comorbid conditions are screened early and further complications prevented. However, it will continue as major problem in developing countries [35]. Developing countries are on epidemiologic transition from infectious disease to chronic disease problems and most sub-Saharan African countries are more vulnerable to the problem [36]. Despite its variation the pooled prevalence of ECG pattern abnormality is high among Type 2 diabetic patients in our review. This is due to the association of diabetes with changes in cardiac metabolism, structure and function [37]. Myocardial dysfunction and affected electrophysiological properties in diabetes contribute mainly due to metabolic disturbances including hyperglycemia, lipotoxicity and insulin resistance [37,38].

In this systematic review and meta-analysis, an increase in body mass index (BMI), presence of hypertension and prolonged duration of diabetic mellitus were found to be associated with electrocardiographic abnormality. The meta-analysis demonstrated that an increase in BMI was more likely associated with the development of electrocardiographic abnormality. This finding is supported by previous studies in Ethiopia, Turkey and USA [21,39,40]. The possible reason might be due to an increase in BMI greater than or equal to 25 kg/m^2^ or being overweight/ obesity, causes changes in the morphological and electrophysiology of myocardial cells, myocardial perfusion, and this affects ECG parameters [30,41]. A systematic review revealed that being overweight/ obesity leads to several hemodynamic changes; including increased stroke volume, blood volume, and elevated left atrial and pulmonary pressure [42]. These changes result in structural alteration in cardiac tissue, such as left atrial enlargement, remodeling and ventricular hypertrophy, ultimately contributing to obesity-induced ECG changes [43]. Additionally, a high BMI may disrupt lipid profiles that promote atherosclerotic plaque formation in the blood vessels, which may lead to vasoconstriction, thrombosis, and myocardial ischemia [37,44].

A prolonged duration of diabetic mellitus is significantly associated with ECG pattern abnormality. Previous findings reported that the duration of diabetes mellitus 5–10 years had ECG changes in India [31], while in Denmark, heart failure is more prevalent among patients with diabetes lasting over ten years [45]. Similarly, the incidence of cardiovascular disease increases with the duration diabetic mellitus in Sweden [46]. This is due to the fact that prolonged duration of diabetes is attributed to elevation of blood sugar, which may damage both the blood vessels and the nerves that innervate the heart. Another reason may be, excess cholesterol and triglyceride lead to plaque formation causing the arterial wall hardening [30,47], and ultimately end up with ECG changes.

The presence of hypertension among Type 2 diabetic patients was significantly associated with ECG abnormality. Studies done in Ethiopia [48], also support our finding. Central obesity was the most common component of the metabolic syndrome to develop high blood pressure in patients with Type 2 diabetic mellitus [49,50]. Acknowledging this hypertension in diabetic patients gives an opportunity for intensive treatment, lifestyle change and addressing co-morbid factors so as to reduce cardiovascular risk [51]. This hypertension may lead to left ventricular hypertrophy resulting from high resistance of cardiac pumping as high blood pressure raises peripheral vascular resistance and left ventricular afterload, and this prolonged exposure to high pressure causes volume and pressure-induced structural remodeling and associated ECG parameter change [21,52].

### Limitations the study

So far some limitations observed from this review. The first was significant heterogeneity between the included studies, a common finding in meta-analyses concerning pooled prevalence estimates. The other is, that most studies done in the hospital setting of this review excluded patients with a history of comorbid health problems like; hypertension, valvular diseases and renal diseases, in contrast to the general population at the community level.

Even though we conducted sensitivity analysis by considering studies using similar case definitions for ECG abnormality, prevalence rates were largely different, suggesting that more factors may have influenced the results, including differences in source population, study setting, variation in age and duration of type 2 diabetes. Besides, survey year, study design and sample size may have an influence on the prevalence of ECG abnormality among patients with Type 2 diabetic mellitus.

## Conclusion

The prevalence of electrocardiographic abnormality among type 2 diabetes patients is high both in the hospital and general population. Duration of diabetic mellitus, high body mass index and presence of hypertension were significantly associated factors in this review. For a deeper understanding of the prevalence of undetected electrocardiographic abnormalities, more inclusive studies should be conducted among high risk groups of individuals at the community level. Moreover, we advise more longitudinal researches to determine the incidence of ECG abnormality among patients with diabetes considering time duration and sex differences.

## Supporting information

S1 File pdf: Pdf form of all Figs (1–10) used for presenting electrocardiographic abnormality among Type 2 diabetic mellitus patients. (PDF)

S2 Data Supplementary data for extracted/included publications. (XLSX)

S3 Data Supplementary table for excluded publications. (DOCX)

## Abbreviations

BMI: Body Mass Index
CVD: Cardiovascular Disease
DM: Diabetic Mellitus
ECG: Electrocardiography
IDF: International Diabetics Federation
MeSH: Medical Subject Headings
PIRD: People/population, Index test, Reference Test, Diseases
PRISMA: Preferred Reporting Items for Systematic Reviews and Meta-Analyses
SDGs: Sustainable Development Goals
T2DM: Type 2 Diabetic Mellitus
USA: United States America
