# Supplementary material for: Pooled prevalence and associated factors of ECG abnormality among type 2 diabetic patients in the last ten years: Systematic review and meta-analysis
Source: PLoS One. 2025 Mar 13;20(3):e0319173. doi: 10.1371/journal.pone.0319173 (PMC11906088; doi:10.1371/journal.pone.0319173)
Supplement: S1 File — (PDF) [file pone.0319173.s001.pdf]

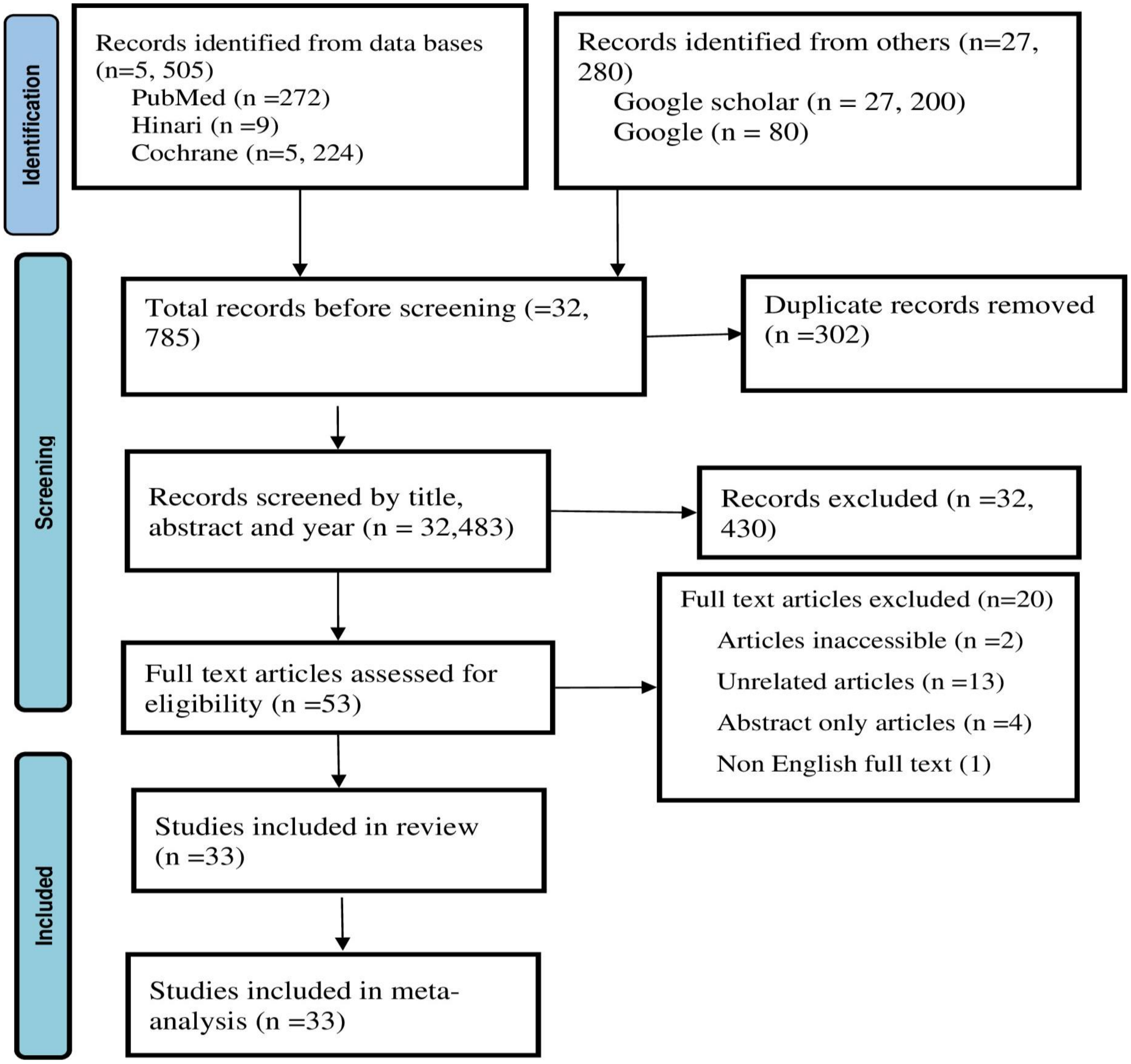

Figure 1: PRISMA flow diagram showing the mechanism of extracting related articles

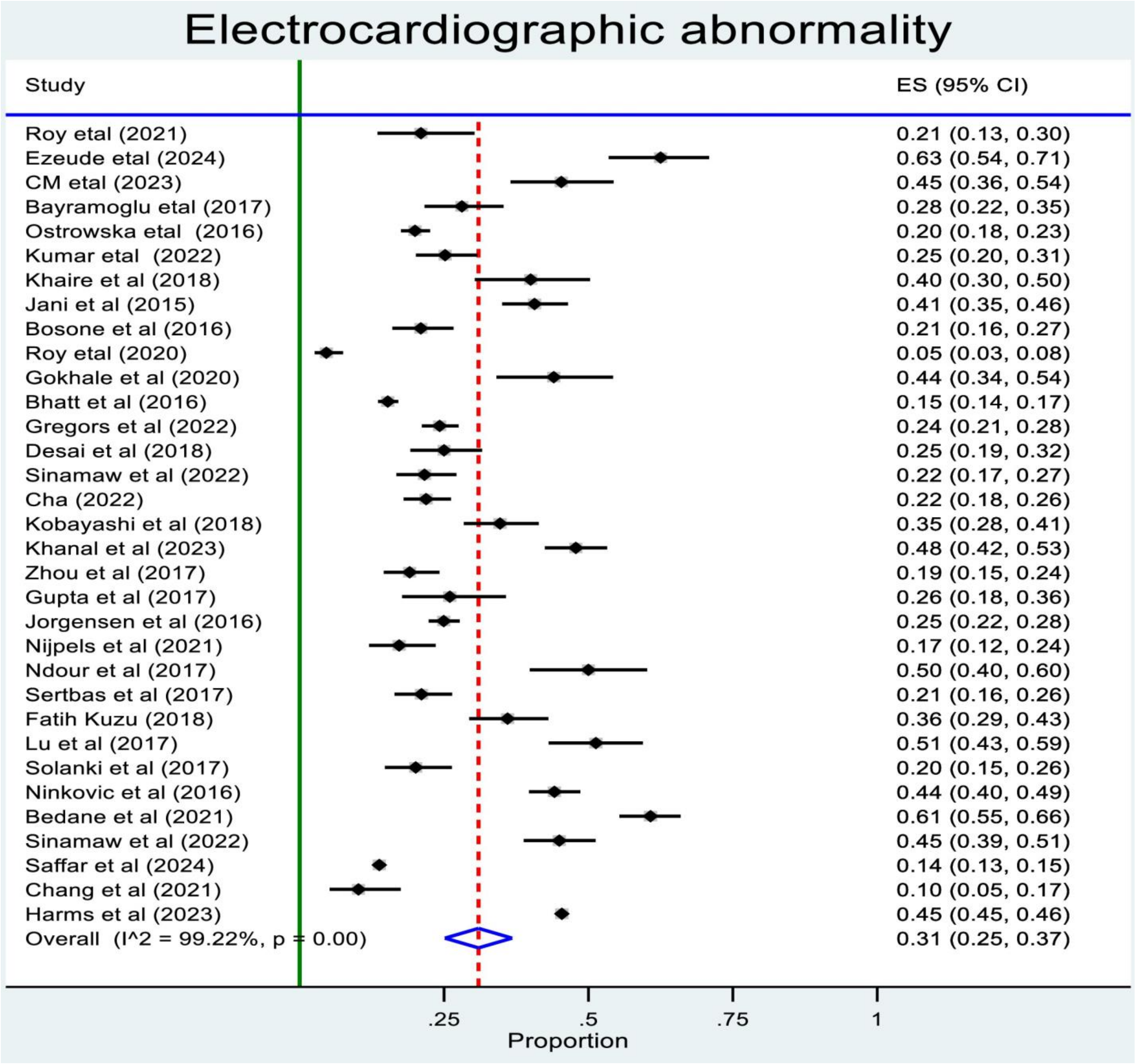

Figure 2: Forest plot showing random effect magnitude of individual studies

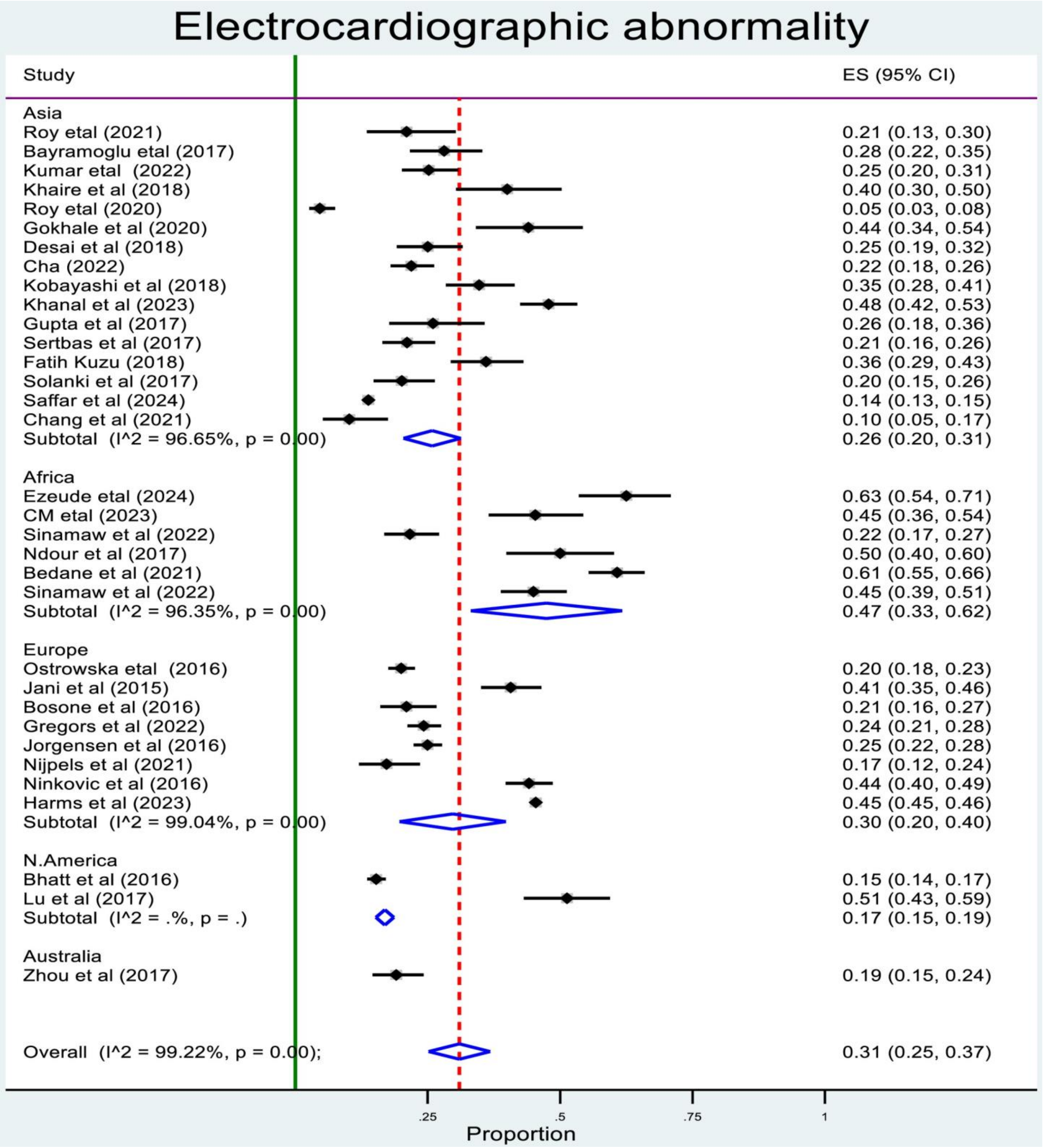

Figure 3: Sub-group analysis Electrocardiographic abnormality by sub-regions/ continent

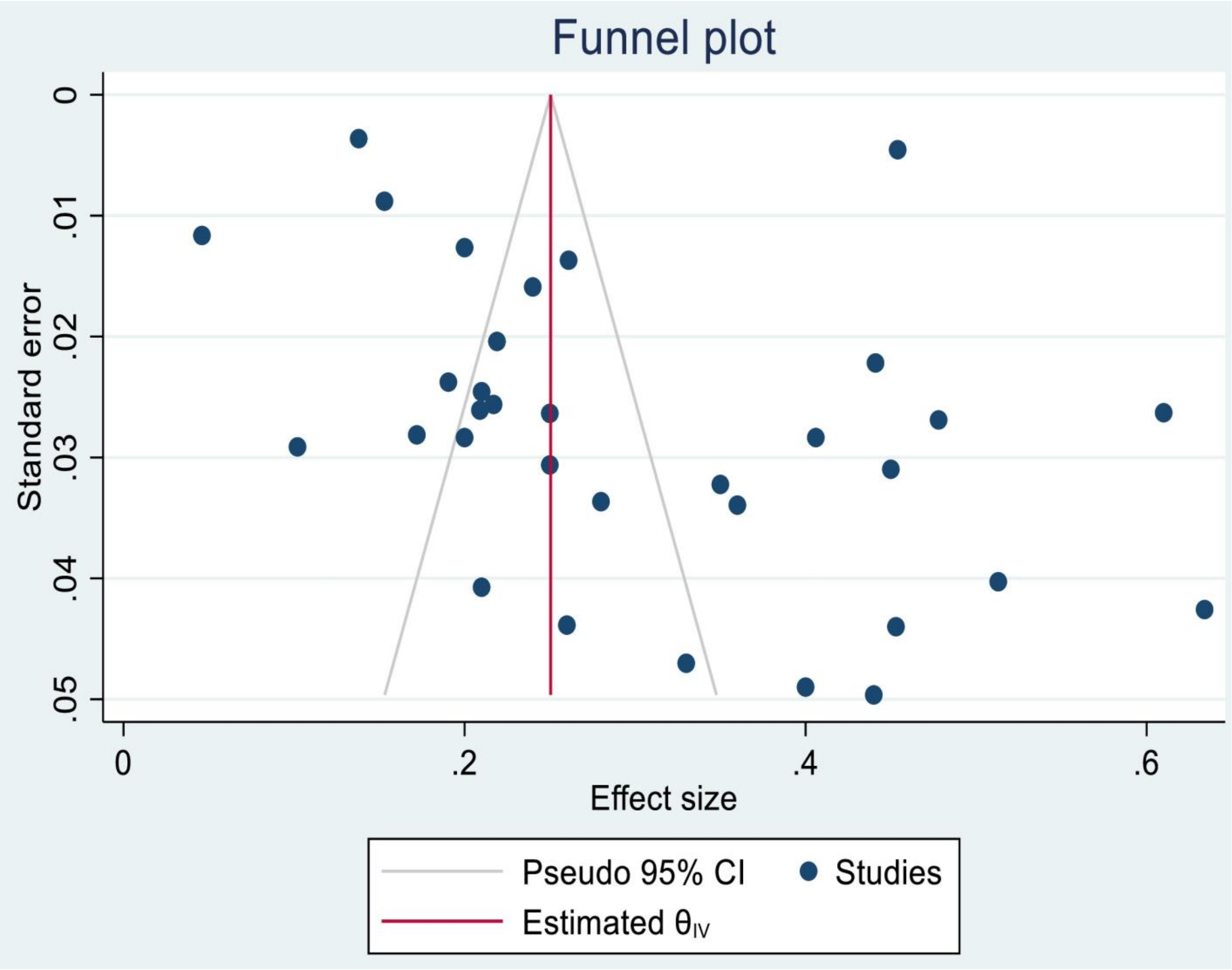

Figure 4: Funnel plot showing the results of publication bias electrocardiographic abnormality

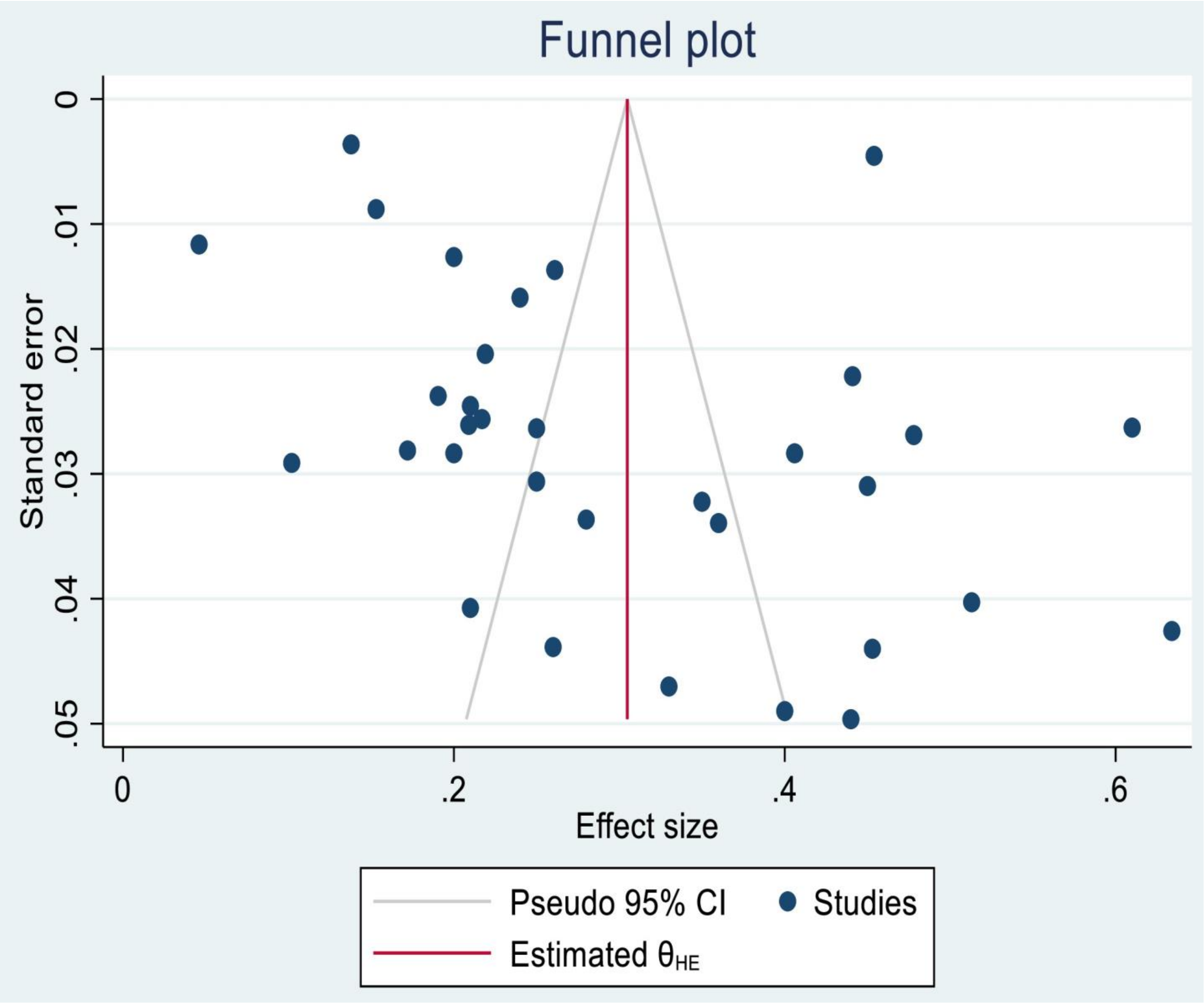

Figure 5: Shows trim and fill analysis electrocardiographic abnormality among Type 2 diabetic mellitus patients

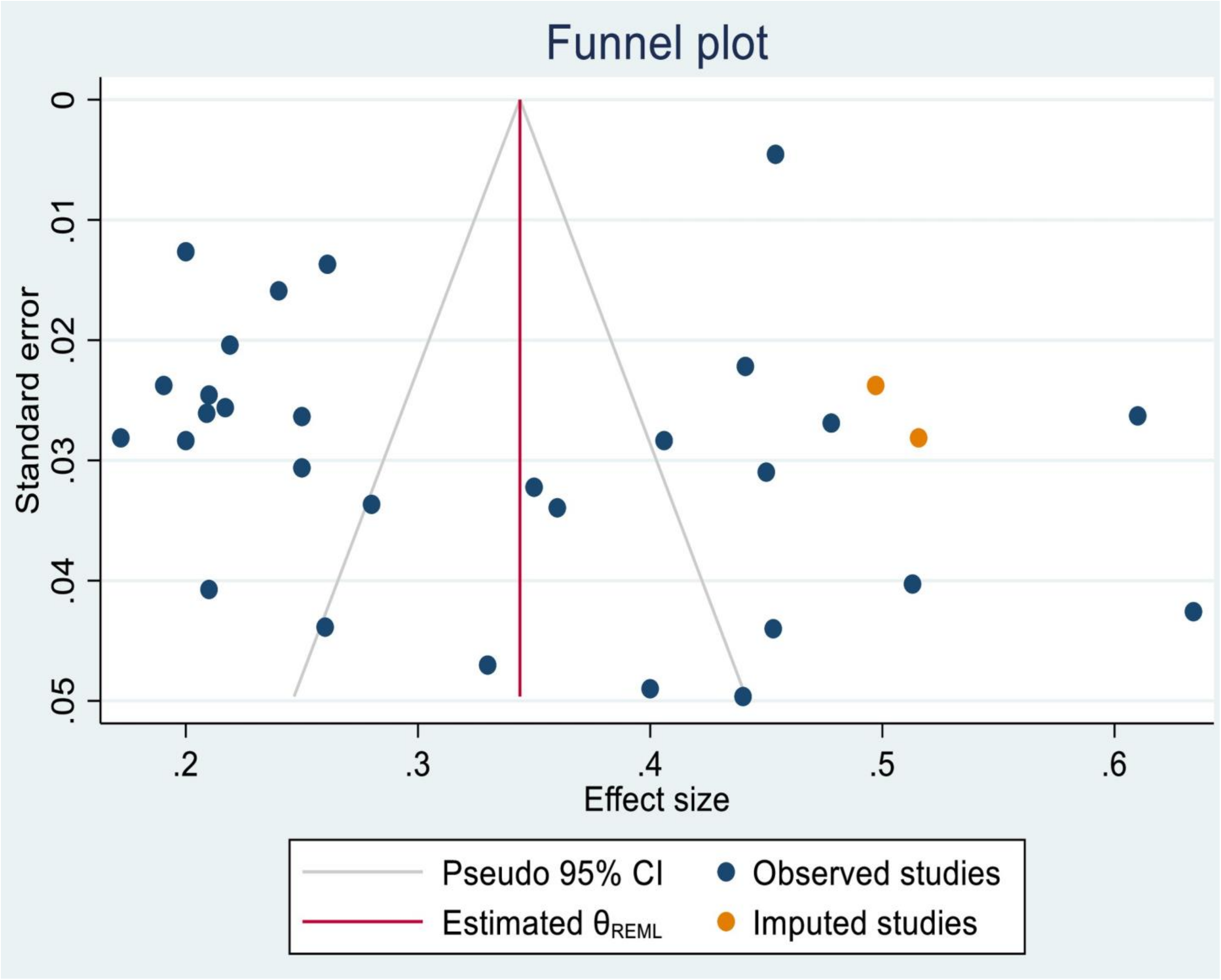

Figure 6: Funnel plot shows the adjusted effective size of electrocardiographic abnormality

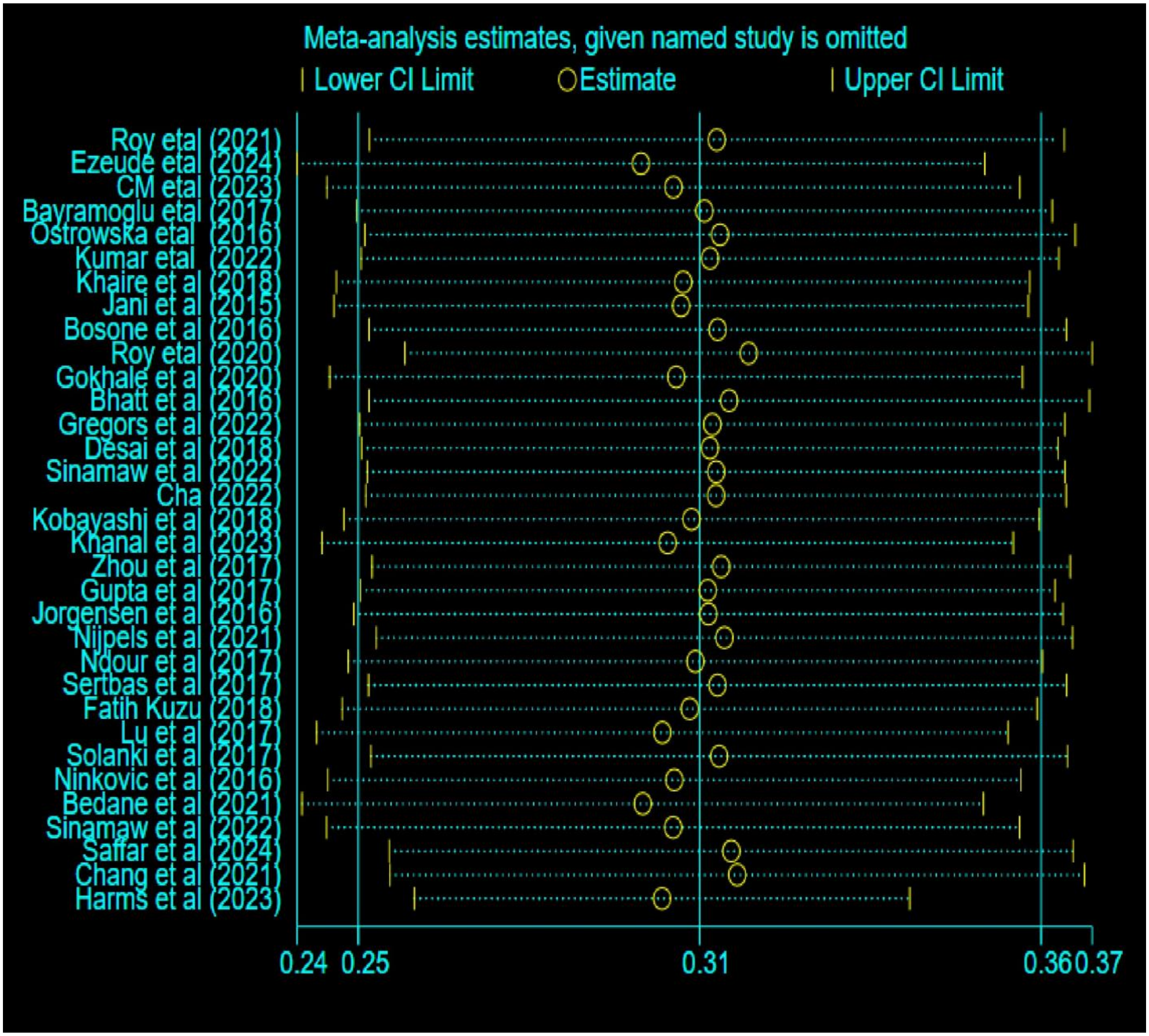

Figure 7: Shows sensitivity analysis of electrocardiographic abnormality among Type 2 diabetic mellitus patients

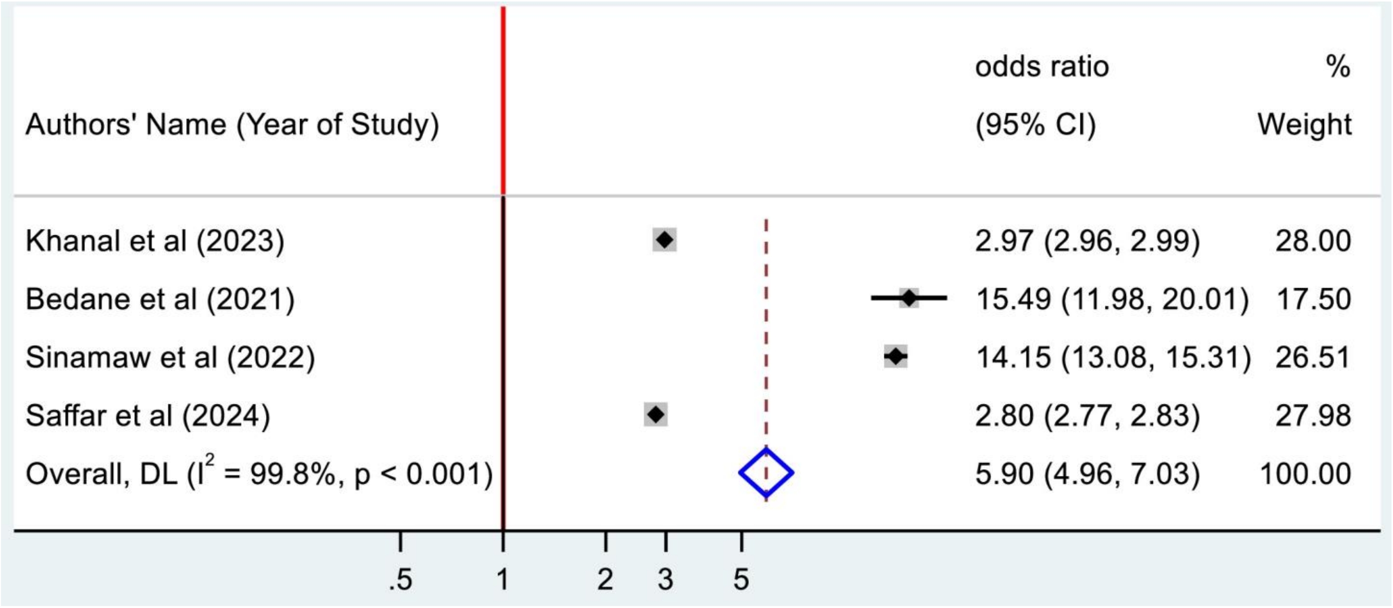

Figure 8: Forest plot for the association between body mass index and ECG abnormality among Type 2 diabetic mellitus patients

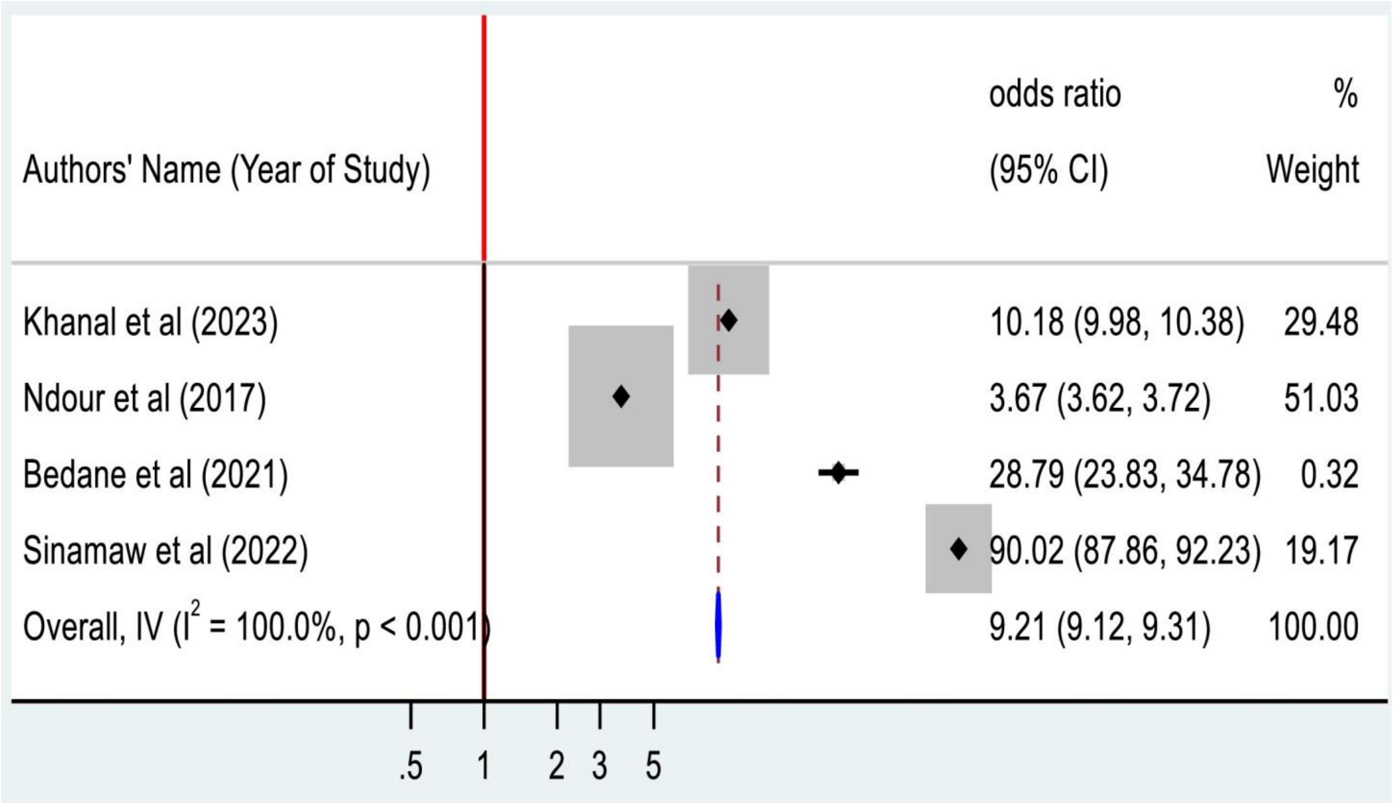

Figure 9: Forest plot for the association between duration of diabetes and ECG abnormality among Type 2 diabetic mellitus patients

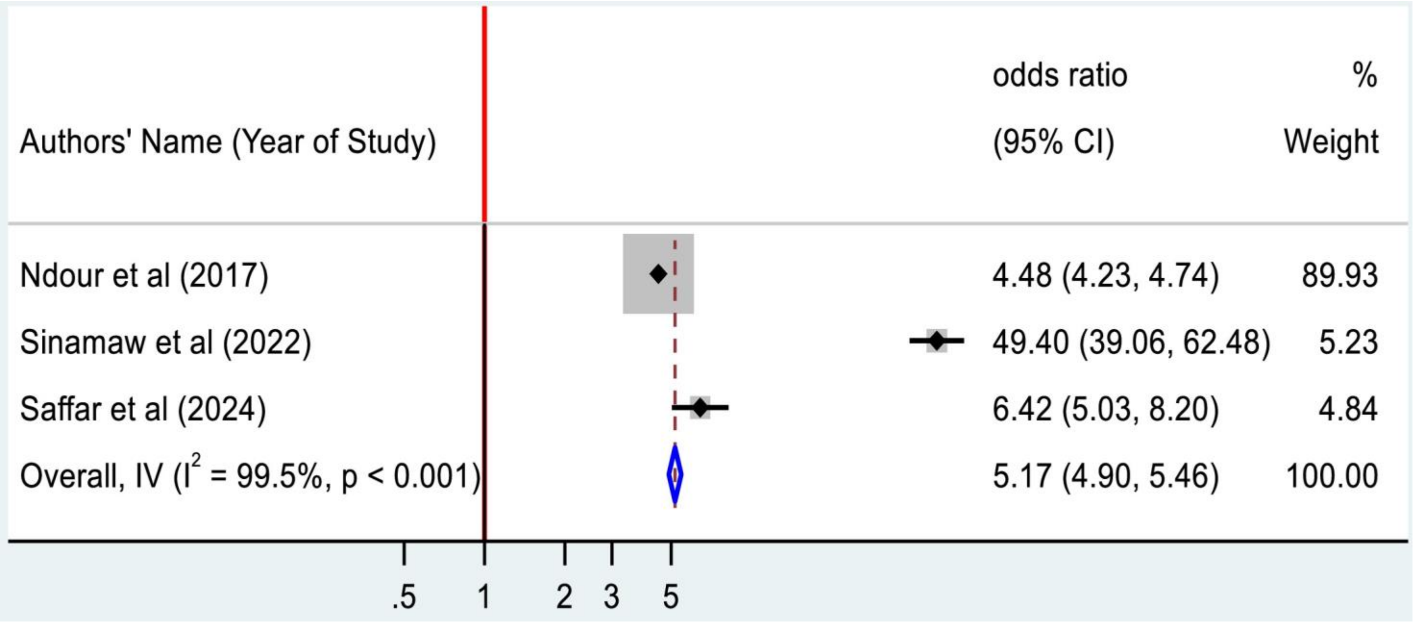

Figure 10: Forest plot for the association between hypertension and ECG abnormality among Type 2 diabetic mellitus patients
