# Supplementary material for: Pooled prevalence and associated factors of ECG abnormality among type 2 diabetic patients in the last ten years: Systematic review and meta-analysis
Source: PLoS One. 2025 Mar 13;20(3):e0319173. doi: 10.1371/journal.pone.0319173 (PMC11906088; doi:10.1371/journal.pone.0319173)
Supplement: S3 Data — (DOCX) [file pone.0319173.s003.docx]

| **Supplementary table showing reason for Excluded publications** | | | | | |
| --- | --- | --- | --- | --- | --- |
| Articles excluded by using full-text review | | | | | |
| **Ser/no** | | **Author name** | **Year** | **Title** | **Reason for exclusion** |
| 1 | | Udora NC, Ejim EC, Young EE, BJ CO: | 2023 | Assessment of left ventricular geometry in normotensive type II diabetic patients. Nigerian journal of clinical practice | Full text could not be accessed |
| 2 | | Shukla SK, Tilkar M, Kapur KS | 2023 | Left Ventricular Diastolic Dysfunction in Patients with Type 2 Diabetes Mellitus and its Association with Age, Gender, Duration and Glycosylated Haemoglobin: A Cross-sectional Study | Full text could not be accessed |
| 3 | | Zhang, Y. T., Li, H. Y., Sun, X. T., Tong, X. W., Shan, Y. Y., Xu, Y. X., … Gao, X. Y. | 2023 | Relationship Between Index of Cardiac Electrophysiological Balance, Frontal QRS-T Angle and Retinopathy in People with Type 2 Diabetes. | Incomplete information ( the content mainly focuses fundus examination rather than ECG changes). |
| 4 | | Sardesai VV, Kokane HT, Mukherjee S, Sangle SA: | 2022 | A study of electrocardiographic and 2D echocardiographic changes in type 2 diabetes mellitus patients without cardiovascular symptoms. | It was done mainly using echocardiography rather than ECG and required information not accessed. |
| 5 | | Mayasari NME, Ghiffari A, Iman AR, Salsabiila R: | 2022 | Factors Related to Electrocardiogram Abnormality in Type 2 Diabetes Mellitus Patients. | The content is compromised and difficult to get the required information. |
| 6 | | Kaze AD, Yuyun MF, Erqou S, Fonarow GC, Echouffo-Tcheugui JB: | 2022 | Severe Hypoglycemia and Incidence of QT Interval Prolongation Among Adults With Type 2 Diabetes. | Required information not found |
| 7 | | Ezeude CM, Ijoma UN, Oguejiofor OC, Young EE, Nwatu CB, Onyenekwe BM, Ugwueze CV: | 2020 | Asymptomatic Cardiovascular Disorders in a Cohort of Clinically Stable Type 2 Diabetes Mellitus Patients in South Eastern Nigeria: A Cross Sectional Study. | On full text review, the article discuss on atherosclerosis rather than ECG abnormality |
| 8 | | Aldous C, Hift R, Pillay S: | 2018 | A retrospective analysis of electrocardiographic abnormalities found in black South African patients with diabetes attending a regional hospital in KwaZulu-Natal. | Study participants not clear, since our review restricts to consider only patients with Type 2 Diabetic mellitus. |
| 9 | | Tanawuttiwat T, Wagner KR, Tomaselli G, Nazarian | 2017 | Left ventricular dysfunction and conduction disturbances in patients with myotonic muscular dystrophy type I and II. | The content of this article was unrelated with the research question. |
| 10 | | Ernande L, Audureau E, Jellis CL, Bergerot C, Henegar C, Sawaki D, Czibik G, Volpi C, Canoui-Poitrine F, Thibault H et al: | 2017 | Clinical Implications of Echocardiographic Phenotypes of Patients With Diabetes Mellitus. | Required information not found |
| 11 | | de Kreutzenberg SV, Solini A, Vitolo E, Boi A, Bacci S, Cocozza S, Nappo R, Rivellese A, Avogaro A, Baroni MG: | 2017 | Silent coronary heart disease in patients with type 2 diabetes: Application of a screening approach in a follow-up study. | The content of this article was unrelated with the research question. |
| 12 | | Zhen Z, Chen Y, Liu J-H, Chan CW-S, Yuen M, Lam KS-L, Tse H-F, Yiu K-H: | 2016 | Increased T-wave alternans is associated with subclinical myocardial structural and functional abnormalities in patients with type 2 diabetes. | The content of this article regarding its aim and approach differ from our research question |
| 13 | | Tokatli A, Kiliçaslan F, Alis M, Yiginer O, Uzun M: | 2016 | Prolonged Tp-e interval, Tp-e/QT ratio and Tp-e/QTc ratio in patients with type 2 diabetes mellitus. | Incomplete information (no prevalence) |
| 14 | | Li B, Pan Y, Li X: | 2016 | Type 2 diabetes induces prolonged p-wave duration without left atrial enlargement. | This was experimental studies with unrelated content. |
| 15 | | Levelt E, Mahmod M, Piechnik SK, Ariga R, Francis JM, Rodgers CT, Clarke WT, Sabharwal N, Schneider JE, Karamitsos TD: | 2016 | Relationship between left ventricular structural and metabolic remodeling in type 2 diabetes. | This article discuss about metabolism change rather than ECG abnormality |
| 16 | | Cha S-A, Yun J-S, Lim T-S, Kang Y-G, Lee K-M, Song K-H, Yoo K-D, Park Y-M, Ko S-H, Ahn Y-B: | 2016 | Baseline-corrected QT (QTc) interval is associated with prolongation of QTc during severe hypoglycemia in patients with type 2 diabetes mellitus. | Non-English language & abstract only. |
| 17 | | Al-Daydamony MM, El-Tahlawi MA, Shawky A: | 2016 | Can myocardial performance index predict the presence of silent ischemia in asymptomatic type 2 diabetic patients? | This was based on echocardiography |
| 18 | | Lutale J, Vetvik K, Gerdts E, Thordarson H, Gulam-Abbas Z: | 2015 | Prevalence and covariates of electrocardiographic left ventricular hypertrophy in diabetic patients in Tanzania. | Full text is not accessible |
| 19 | | Kittnar O: | 2015 | Electrocardiographic changes in diabetes mellitus. | On full text investigation required information’s are missed |
| **Articles excluded by year and topic screening** | | | | | **Reason for Exclusion** |
| 20 | - Zwetsloot PP, van der Naald M, Jones D, Reid A, Chamuleau S, Mathur A: Stem cell treatment for acute myocardial infarction. 2024(8). - Yeung E, Baessler K, Christmann-Schmid C, Haya N, Chen Z, Wallace SA, Mowat A, Maher C: Transvaginal mesh or grafts or native tissue repair for vaginal prolapse. 2024(3). - Wallis JA, Shepperd S, Makela P, Han JX, Tripp EM, Gearon E, Disher G, Buchbinder R, O'Connor D: Factors influencing the implementation of early discharge hospital at home and admission avoidance hospital at home: a qualitative evidence synthesis. 2024(3). - Wallis JA, Bourne AM, Jessup RL, Johnston RV, Frydman A, Cyril S, Buchbinder R: Manual therapy and exercise for lateral elbow pain. 2024(5). - Välimäki M, Lantta T, Kontio R: Risk assessment for aggressive behaviour in schizophrenia. 2024(5). - Umaefulam V, Safi S, Lingham G, Gordon I, Mueller A, Krishnam NS, Alves Carneiro V, Yu M, Evans JR, Keel S: Approaches for delivery of refractive and optical care services in community and primary care settings. 2024(5). - Tunnicliffe DJ, Reid S, Craig JC, Samuels JA, Molony DA, Strippoli GFM: Non‐immunosuppressive treatment for IgA nephropathy. 2024(2). - Tingle SJ, Thompson ER, Figueiredo RS, Moir JAG, Goodfellow M, Talbot D, Wilson CH: Normothermic and hypothermic machine perfusion preservation versus static cold storage for deceased donor kidney transplantation. 2024(7). - Tavender E, Eapen N, Wang J, Rausa VC, Babl FE, Phillips N: Triage tools for detecting cervical spine injury in paediatric trauma patients. *Cochrane Database of Systematic Reviews* 2024(3). - Tavassoli S, Ziaei H, Yadegarfar ME, Gokul A, Kernohan A, Evans JR, Ziaei M: Trifocal versus extended depth of focus (EDOF) intraocular lenses after cataract extraction. *Cochrane Database of Systematic Reviews* 2024(7). - Tan J, Renton WD, Whittle SL, Takken T, Johnston RV, Tiller G, Munro J, Buchbinder R: Methotrexate for juvenile idiopathic arthritis. *Cochrane Database of Systematic Reviews* 2024(2). - Sunaga T, Maeda M, Saulle R, Ng SM, Sato MT, Hasegawa T, Mason AN, Noma H, Ota E: Anti‐vascular endothelial growth factor biosimilars for neovascular age‐related macular degeneration. *Cochrane Database of Systematic Reviews* 2024(6). - Streck JM, Rigotti NA, Livingstone-Banks J, Tindle HA, Clair C, Munafò MR, Sterling-Maisel C, Hartmann-Boyce J: Interventions for smoking cessation in hospitalised patients. *Cochrane Database of Systematic Reviews* 2024(5). - Storman D, Swierz MJ, Mitus JW, Pedziwiatr M, Liang N, Wolff R, Bala MM: Microwave coagulation for liver metastases. *Cochrane Database of Systematic Reviews* 2024(3). - Stacey D, Lewis KB, Smith M, Carley M, Volk R, Douglas EE, Pacheco-Brousseau L, Finderup J, Gunderson J, Barry MJ *et al*: Decision aids for people facing health treatment or screening decisions. *Cochrane Database of Systematic Reviews* 2024(1). - Smith TO, Casey L, McNamara IR, Hing CB: Surgical fixation methods for tibial plateau fractures. *Cochrane Database of Systematic Reviews* 2024(8). - Ski CF, Taylor RS, McGuigan K, Long L, Lambert JD, Richards SH, Thompson DR: Psychological interventions for depression and anxiety in patients with coronary heart disease, heart failure or atrial fibrillation. *Cochrane Database of Systematic Reviews* 2024(4). - Sinha S, Gabriel VA, Arora RK, Shin W, Scott J, Bharadia SK, Verly M, Rahmani WM, Nickerson DA, Fraulin FOG *et al*: Interventions for postburn pruritus. *Cochrane Database of Systematic Reviews* 2024(6). - Singleton H, Hodder A, Almilaji O, Ersser SJ, Heaslip V, O'Meara S, Boyers D, Roberts A, Scott H, Van Onselen J *et al*: Educational and psychological interventions for managing atopic dermatitis (eczema). *Cochrane Database of Systematic Reviews* 2024(8). - Silva TO, Darzé ES, Costa MM, José Junior L, Ximenes AAB, Fernandes F, Rocha MS, Noya-Rabelo MM, Ritt LEF: Scintigraphic and Echocardiographic Study of Patients with Pathogenic or Probably Pathogenic Variants of the TTR Gene without Overt Cardiac Involvement. *Arq Bras Cardiol* 2024, 121(4):e20230216. - Sharrad KJ, Sanwo O, Cuevas-Asturias S, Kew KM, Carson-Chahhoud KV, Pike KC: Psychological interventions for asthma in children and adolescents. *Cochrane Database of Systematic Reviews* 2024(1). - Schults JA, Reynolds H, Rickard CM, Culwick MD, Mihala G, Alexandrou E, Ullman AJ: Dressings and securement devices to prevent complications for peripheral arterial catheters. *Cochrane Database of Systematic Reviews* 2024(5). - Schults JA, Kleidon T, Charles K, Young E, and Ullman AJ: Peripherally inserted central catheter design and material for reducing catheter failure and complications. *Cochrane Database of Systematic Reviews* 2024(6). - Sandall J, Fernandez Turienzo C, Devane D, Soltani H, Gillespie P, Gates S, Jones LV, Shennan AH, Rayment-Jones H: Midwife continuity of care models versus other models of care for childbearing women. *Cochrane Database of Systematic Reviews* 2024(4). - Sakai K, Niimi M, Momosaki R, Hoshino E, Yoneoka D, Nakayama E, Masuoka K, Maeda T, Takahashi N, Sakata N: Nutritional therapy for reducing disability and improving activities of daily living in people after stroke. *Cochrane Database of Systematic Reviews* 2024(8). - Sahranavard T, Soflaei SS, Alimi R, Pourali G, Nasrabadi M, Yadollahi A, Sharifi S, Alimi H, Shahri B, Ghalibaf AM *et al*: Factors associated with prolonged QTc interval in Iranian population: MASHAD cohort study. *J Electrocardiol* 2024, 84:112-122. - Romano M, Minozzi S, Bettany-Saltikov J, Zaina F, Chockalingam N, Kotwicki T, Maier-Hennes A, Arienti C, Negrini S: Therapeutic exercises for idiopathic scoliosis in adolescents. *Cochrane Database of Systematic Reviews* 2024(2). - Roheger M, Riemann S, Brauer A, McGowan E, Grittner U, Flöel A, Meinzer M: Non‐pharmacological interventions for improving language and communication in people with primary progressive aphasia. *Cochrane Database of Systematic Reviews* 2024(5). - Riganti P, Ruiz Yanzi MV, Escobar Liquitay CM, Sgarbossa NJ, Alarcon-Ruiz CA, Kopitowski KS, Franco JVA: Shared decision‐making for supporting women’s decisions about breast cancer screening. *Cochrane Database of Systematic Reviews* 2024(5). - Renner RM, Ennis M, McKercher AE, Henderson JT, Edelman A: Local anaesthesia for pain control in first trimester surgical abortion. *Cochrane Database of Systematic Reviews* 2024(2). - Reilly S, Hobson-Merrett C, Gibbons B, Jones B, Richards D, Plappert H, Gibson J, Green M, Gask L, Huxley PJ *et al*: Collaborative care approaches for people with severe mental illness. *Cochrane Database of Systematic Reviews* 2024(5). - Redfern J, Tu Q, Hyun K, Hollings MA, Hafiz N, Zwack C, Free C, Perel P, Chow CK: Mobile phone text messaging for medication adherence in secondary prevention of cardiovascular disease. *Cochrane Database of Systematic Reviews* 2024(3). - Rasoul D, Zhang J, Farnell E, Tsangarides AA, Chong S, Fernando R, Zhou C, Ihsan M, Ahmed S, Lwin TS *et al*: Continuous infusion versus bolus injection of loop diuretics for acute heart failure. *Cochrane Database of Systematic Reviews* 2024(5). - Radford M, Estcourt LJ, Sirotich E, Pitre T, Britto J, Watson M, Brunskill SJ, Fergusson DA, Dorée C, Arnold DM: Restrictive versus liberal red blood cell transfusion strategies for people with haematological malignancies treated with intensive chemotherapy or radiotherapy, or both, with or without haematopoietic stem cell support. *Cochrane Database of Systematic Reviews* 2024(5). - Pucker AD, Yim TW, Rueff E, Ngo W, Tichenor AA, Conto JE: LipiFlow for the treatment of dry eye disease. *Cochrane Database of Systematic Reviews* 2024(2). - Prajapathi S, Pradhan A: Predictors of permanent pacemaker implantation following transcatheter aortic valve replacement-the search is still on! *World J Cardiol* 2024, 16(3):104-108. - Pouncey AL, Yeldham G, Magan T, Lucenteforte E, Jaffer U, Virgili G: Halo sign on temporal artery ultrasound versus temporal artery biopsy for giant cell arteritis. *Cochrane Database of Systematic Reviews* 2024(2). - Pearce RB, Gontsarova A, Richardson D, Methley AM, Watt H, Tsang K, Carswell C: Shunting for idiopathic normal pressure hydrocephalus. *Cochrane Database of Systematic Reviews* 2024(8). - Patterson LG, Tingle SJ, Rix DA, Manas DM, Wilson CH: Routine intraoperative ureteric stenting for kidney transplant recipients. *Cochrane Database of Systematic Reviews* 2024(7). - Parker ED, Lin J, Mahoney T, Ume N, Yang G, Gabbay RA, ElSayed NA, Bannuru RR: Economic costs of diabetes in the US in 2022. *Diabetes Care* 2024, 47(1):26-43. - Owens D, Watkinson S, Harrison JE, Turner S, Worthington HV: Orthodontic treatment for prominent lower front teeth (Class III malocclusion) in children. *Cochrane Database of Systematic Reviews* 2024(4). - Orton J, Doyle LW, Tripathi T, Boyd R, Anderson PJ, Spittle A: Early developmental intervention programmes provided post hospital discharge to prevent motor and cognitive impairment in preterm infants. *Cochrane Database of Systematic Reviews* 2024(2). - Oostvogels L, Weibel S, Meißner M, Kranke P, Meyer-Frießem CH, Pogatzki-Zahn E, Schnabel A: Erector spinae plane block for postoperative pain. *Cochrane Database of Systematic Reviews* 2024(2). - Olasupo OO, Noronha N, Lowe MS, Ansel D, Bhatt M, Matino D: Non‐clotting factor therapies for preventing bleeds in people with congenital hemophilia A or B. *Cochrane Database of Systematic Reviews* 2024(2). - Okwundu CI, Bhutani VK, Uthman OA, Smith J, Olowoyeye A, Fiander M, Wiysonge CS: Transcutaneous bilirubinometry for detecting jaundice in term or late preterm neonates. *Cochrane Database of Systematic Reviews* 2024(5). - Ngwenya O, Lensen SF, Vail A, Mol BWJ, Broekmans FJ, Wilkinson J: Individualised gonadotropin dose selection using markers of ovarian reserve for women undergoing in vitro fertilisation plus intracytoplasmic sperm injection (IVF/ICSI). *Cochrane Database of Systematic Reviews* 2024(1). - Natale P, Tunnicliffe DJ, Toyama T, Palmer SC, Saglimbene VM, Ruospo M, Gargano L, Stallone G, Gesualdo L, Strippoli GFM: Sodium‐glucose co‐transporter protein 2 (SGLT2) inhibitors for people with chronic kidney disease and diabetes. *Cochrane Database of Systematic Reviews* 2024(5). - Natale P, Palmer SC, Ruospo M, Longmuir H, Dodds B, Prasad R, Batt TJ, Jose MD, Strippoli GFM: Anticoagulation for people receiving long‐term haemodialysis. *Cochrane Database of Systematic Reviews* 2024(1). - Natale P, Palmer SC, Navaneethan SD, Craig JC, Strippoli GFM: Angiotensin‐converting‐enzyme inhibitors and angiotensin receptor blockers for preventing the progression of diabetic kidney disease. *Cochrane Database of Systematic Reviews* 2024(4). - Natale P, Mooi PKL, Green SC, Cross NB, Cooper TE, Webster AC, Masson P, Craig JC, Strippoli GFM: Antihypertensive treatment for kidney transplant recipients. *Cochrane Database of Systematic Reviews* 2024(7). - Naing C, Ni H, Aung HH, Pavlov CS: Endoscopic sphincterotomy for adults with biliary sphincter of Oddi dysfunction. *Cochrane Database of Systematic Reviews* 2024(3). - Naing C, Ni H, Aung HH, Htet NH, Nikolova D: Gene therapy for people with hepatocellular carcinoma. *Cochrane Database of Systematic Reviews* 2024(6). - Naik S, Lepine S, Nagels HE, Siristatidis CS, Kroon B, McDowell S: Androgens (dehydroepiandrosterone or testosterone) for women undergoing assisted reproduction. *Cochrane Database of Systematic Reviews* 2024(6). - Munteanu SE, Buldt A, Lithgow MJ, Cotchett M, Landorf KB, Menz HB: Non‐surgical interventions for treating osteoarthritis of the big toe joint. *Cochrane Database of Systematic Reviews* 2024(6). - Molloy C, Long L, Mordi IR, Bridges C, Sagar VA, Davies EJ, Coats AJS, Dalal H, Rees K, Singh SJ *et al*: Exercise‐based cardiac rehabilitation for adults with heart failure. *Cochrane Database of Systematic Reviews* 2024(3). - Mdege ND, Shah S, Dogar O, Pool ERM, Weatherburn P, Siddiqi K, Zyambo C, Livingstone-Banks J: Interventions for tobacco use cessation in people living with HIV. *Cochrane Database of Systematic Reviews* 2024(8). - Mayer SF, Corcoran C, Kennedy L, Leucht S, Bighelli I: Cognitive behavioural therapy added to standard care for first‐episode and recent‐onset psychosis. *Cochrane Database of Systematic Reviews* 2024(3). - Marchand M, Erickson AC, Gillman L, Haywood R, Morrison J, Jaworsky D, Drouin O, Laksman Z, Krahn AD, Arbour L: The Impact of Chronic Disease on the Corrected QT (QTc) Value in Women in a British Columbia First Nations Population. *Can J Cardiol* 2024, 40(1):89-97. - Ma GW, Kucey A, Tyagi SC, Papia G, Kucey DS, Varcoe RL, Forbes T, Neville R, Dueck AD, Kayssi A: The role of sealants for achieving anastomotic hemostasis in vascular surgery. *Cochrane Database of Systematic Reviews* 2024(5). - Lusa V, Karjalainen TV, Pääkkönen M, Rajamäki T, Jaatinen K: Surgical versus non‐surgical treatment for carpal tunnel syndrome. *Cochrane Database of Systematic Reviews* 2024(1). - Liu C, Wei Z, Jian F, McIntyre G, Millett DT, Lai W, Wang Y: Initial arch wires used in orthodontic treatment with fixed appliances. *Cochrane Database of Systematic Reviews* 2024(2). - Lingineni VB, Mangudkar S, Gokhale VS, Malik S, Yadav P: Linking Diabetic Retinopathy Severity to Coronary Artery Disease Risk Factors in Type 2 Diabetic Patients. *Cureus* 2024, 16(7):e65018. - Lee CH, Wu YY, Huang TC, Lin C, Zou YF, Cheng JC, Chen PH, Jhou HJ, Ho CL: Maintenance therapy for chronic lymphocytic leukaemia. *Cochrane Database of Systematic Reviews* 2024(1). - Lax SJ, Van Vogt E, Candy B, Steele L, Reynolds C, Stuart B, Parker R, Axon E, Roberts A, Doyle M *et al*: Topical anti‐inflammatory treatments for eczema: network meta‐analysis. *Cochrane Database of Systematic Reviews* 2024(8). - Langer P, John L, Monsef I, Scheid C, Piechotta V, Skoetz N: Daratumumab and antineoplastic therapy versus antineoplastic therapy only for adults with newly diagnosed multiple myeloma ineligible for transplant. *Cochrane Database of Systematic Reviews* 2024(5). - Langer G, Wan CS, Fink A, Schwingshackl L, Schoberer D: Nutritional interventions for preventing and treating pressure ulcers. *Cochrane Database of Systematic Reviews* 2024(2). - Kulkarni M, Prabhu AR, Rao IR, Nagaraju SP: Interventions for preventing haemodialysis dysequilibrium syndrome. *Cochrane Database of Systematic Reviews* 2024(5). - Kukendrarajah K, Ahmad M, Carrington M, Ioannou A, Taylor J, Razvi Y, Papageorgiou N, Mead GE, Nevis IF, D'Ascenzo F *et al*: External electrical and pharmacological cardioversion for atrial fibrillation, atrial flutter or atrial tachycardias: a network meta‐analysis. *Cochrane Database of Systematic Reviews* 2024(6). - Kuehn R, Uchiumi LJ, Tamarozzi F: Treatment of uncomplicated hepatic cystic echinococcosis (hydatid disease). *Cochrane Database of Systematic Reviews* 2024(7). - Korula P, Alexander H, John J, Kirubakaran R, Singh B, Tharyan P, Rupali P: Favipiravir for treating COVID‐19. *Cochrane Database of Systematic Reviews* 2024(2). - Jain K, Wainwright CE, Smyth AR: Bronchoscopy‐guided antimicrobial therapy for cystic fibrosis. *Cochrane Database of Systematic Reviews* 2024(5). - Isaksen JL, Sivertsen CB, Jensen CZ, Graff C, Linz D, Ellervik C, Jensen MT, Jørgensen PG, Kanters JK: Electrocardiographic markers in patients with type 2 diabetes and the role of diabetes duration. *Journal of Electrocardiology* 2024, 84:129-136. - Harris IM, Lee KC, Deeks JJ, Moore DJ, Moiemen NS, Dretzke J: Pressure‐garment therapy for preventing hypertrophic scarring after burn injury. *Cochrane Database of Systematic Reviews* 2024(1). - Hajibandeh S, Hajibandeh S, Maw A: Purse‐string skin closure versus linear skin closure in people undergoing stoma reversal. *Cochrane Database of Systematic Reviews* 2024(3). - Gross AR, Lee H, Ezzo J, Chacko N, Gelley G, Forget M, Morien A, Graham N, Santaguida PL, Rice M *et al*: Massage for neck pain. *Cochrane Database of Systematic Reviews* 2024(2). - Groenewegen A, Zwartkruis VW, Rienstra M, Zuithoff NPA, Hollander M, Koffijberg H, Oude Wolcherink M, Cramer MJ, van der Schouw YT, Hoes AW *et al*: Diagnostic yield of a proactive strategy for early detection of cardiovascular disease versus usual care in adults with type 2 diabetes or chronic obstructive pulmonary disease in primary care in the Netherlands (RED-CVD): a multicentre, pragmatic, cluster-randomised, controlled trial. *Lancet Public Health* 2024, 9(2):e88-e99. - Gordon M, Sinopoulou V, Akobeng AK, Sarian A, Moran GW: Infliximab for maintenance of medically‐induced remission in Crohn's disease. *Cochrane Database of Systematic Reviews* 2024(2). - Gonzalez-Lorenzo M, Ridley B, Minozzi S, Del Giovane C, Peryer G, Piggott T, Foschi M, Filippini G, Tramacere I, Baldin E *et al*: Immunomodulators and immunosuppressants for relapsing‐remitting multiple sclerosis: a network meta‐analysis. *Cochrane Database of Systematic Reviews* 2024(1). - Glenton C, Paulsen E, Agarwal S, Gopinathan U, Johansen M, Kyaddondo D, Munabi-Babigumira S, Nabukenya J, Nakityo I, Namaganda R *et al*: Healthcare workers’ informal uses of mobile phones and other mobile devices to support their work: a qualitative evidence synthesis. *Cochrane Database of Systematic Reviews* 2024(8). - Gibbs VN, Champaneria R, Sandercock J, Welton NJ, Geneen LJ, Brunskill SJ, Dorée C, Kimber C, Palmer AJR, Estcourt LJ: Pharmacological interventions for the prevention of bleeding in people undergoing elective hip or knee surgery: a systematic review and network meta‐analysis. *Cochrane Database of Systematic Reviews* 2024(1). - Fung THM, Yim TW, Lois N, Wright DM, Liu SH, Williamson T: Face‐down positioning or posturing after pars plana vitrectomy for macula‐involving rhegmatogenous retinal detachments. *Cochrane Database of Systematic Reviews* 2024(3). - Franco JVA, Bongaerts B, Metzendorf MI, Risso A, Guo Y, Peña Silva L, Boeckmann M, Schlesinger S, Damen J, Richter B *et al*: Diabetes as a risk factor for tuberculosis disease. *Cochrane Database of Systematic Reviews* 2024(8). - Franco JVA, Bongaerts B, Metzendorf MI, Risso A, Guo Y, Peña Silva L, Boeckmann M, Schlesinger S, Damen J, Richter B *et al*: Undernutrition as a risk factor for tuberculosis disease. *Cochrane Database of Systematic Reviews* 2024(6). - Fonseca AV, Toledo Barros MG, Baptista-Silva JCC, Amorim JE, Vasconcelos V: Interventions for thrombosed haemodialysis arteriovenous fistulas and grafts. *Cochrane Database of Systematic Reviews* 2024(2). - Ferguson C, Shaikh F, Allida SM, Hendriks J, Gallagher C, Bajorek BV, Donkor A, Inglis SC: Clinical service organisation for adults with atrial fibrillation. *Cochrane Database of Systematic Reviews* 2024(7). - Ezeude C, Nkpozi MO, Abonyi MC, Onwuegbuna AA, Anyanwu AC, Ikeabbah HE, Ezeude AM, Anokwulu IO, Oguejiofor OC: Pattern of Electrocardiographic Abnormalities in Asymptomatic Type 2 Diabetes Mellitus Out-patients at Nnamdi Azikiwe University Teaching Hospital, Nigeria. *Tropical Journal of Medical Research* 2024, 23(1):17-25. - Ethier I, Hayat A, Pei J, Hawley CM, Francis RS, Wong G, Craig JC, Viecelli AK, Htay H, Ng S *et al*: Peritoneal dialysis versus haemodialysis for people commencing dialysis. *Cochrane Database of Systematic Reviews* 2024(6). - Elfghi M, Dunne D, Jones J, Gibson I, Flaherty G, McEvoy JW, Sultan S, Jordan F, Tawfick W: Mobile health technologies to improve walking distance in people with intermittent claudication. *Cochrane Database of Systematic Reviews* 2024(2). - El-Damanawi R, Stanley IK, Staatz C, Pascoe EM, Craig JC, Johnson DW, Mallett AJ, Hawley CM, Milanzi E, Hiemstra TF *et al*: Metformin for preventing the progression of chronic kidney disease. *Cochrane Database of Systematic Reviews* 2024(6). - Edgar K, Iliffe S, Doll HA, Clarke MJ, Gonçalves-Bradley DC, Wong E, Shepperd S: Admission avoidance hospital at home. *Cochrane Database of Systematic Reviews* 2024(3). - Dogan M, Baykiz D, Yilmaz I, Hacioglu Kasim FB, Bilir BE, Bilir B: Evaluation of the Effect of Favipiravir on QT Intervals in COVID-19 Patients with and without Diabetes Mellitus. *J Coll Physicians Surg Pak* 2024, 34(6):659-666. - Dias CGP, Godoy-Santos AL, Ferrari J, Ferretti M, Lenza M: Surgical interventions for treating hallux valgus and bunions. *Cochrane Database of Systematic Reviews* 2024(7). - De Siqueira J, Russell DA, Siddle HJ, Richards SH, McGinnis E: Non‐surgical interventions for preventing contralateral tissue loss and amputation in dysvascular patients with a primary major lower limb amputation. *Cochrane Database of Systematic Reviews* 2024(8). - De Rop L, Bos DAG, Stegeman I, Holtman G, Ochodo EA, Spijker R, Otieno JA, Alkhlaileh F, Deeks JJ, Dinnes J *et al*: Accuracy of routine laboratory tests to predict mortality and deterioration to severe or critical COVID‐19 in people with SARS‐CoV‐2. *Cochrane Database of Systematic Reviews* 2024(8). - de Boer A, Tamminga SJ, Boschman JS, Hoving JL: Non‐medical interventions to enhance return to work for people with cancer. *Cochrane Database of Systematic Reviews* 2024(3). - Dahm P, Ergun O, Uhlig A, Bellut L, Risk MC, Lyon JA, Kunath F: Cytoreductive nephrectomy in metastatic renal cell carcinoma. *Cochrane Database of Systematic Reviews* 2024(6). - Cucato G, Longano PPL, Perren D, Ritti-Dias RM, Saxton JM: Effects of additional exercise therapy after a successful vascular intervention for people with symptomatic peripheral arterial disease. *Cochrane Database of Systematic Reviews* 2024(5). - Cheetham MS, Ethier I, Krishnasamy R, Cho Y, Palmer SC, Johnson DW, Craig JC, Stroumza P, Frantzen L, Hegbrant J *et al*: Home versus in‐centre haemodialysis for people with kidney failure. *Cochrane Database of Systematic Reviews* 2024(4). - Çetin Ş, Bayraktar A, Demiröz Ö, Karabay KÖ, Yalçınkaya E: Fragmented QRS as a predictor of cardiovascular events in patients with type 2 diabetes mellitus: a 36-month follow-up data. *Anatolian Journal of Cardiology* 2024, 28(4):208. - Caulley L, Quinn JG, Doyle MA, Alkherayf F, Metzendorf MI, Kilty S, Hunink MG: Surgical and non‐surgical interventions for primary and salvage treatment of growth hormone‐secreting pituitary adenomas in adults. *Cochrane Database of Systematic Reviews* 2024(2). - Cashmore BA, Cooper TE, Evangelidis NM, Green SC, Lopez-Vargas P, Tunnicliffe DJ: Education programmes for people with chronic kidney disease and diabetes. *Cochrane Database of Systematic Reviews* 2024(8). - Cai Z, Zhao Z, Ma Q, Shen C, Jiang Z, Liu C, Liu C, Zhang B: Midline and off‐midline wound closure methods after surgical treatment for pilonidal sinus. *Cochrane Database of Systematic Reviews* 2024(1). - Cai Z, Mu M, Ma Q, Liu C, Jiang Z, Liu B, Ji G, Zhang B: Uncut Roux‐en‐Y reconstruction after distal gastrectomy for gastric cancer. *Cochrane Database of Systematic Reviews* 2024(2). - Buijtendijk MFJ, Bet BB, Leeflang MMG, Shah H, Reuvekamp T, Goring T, Docter D, Timmerman M, Dawood Y, Lugthart MA *et al*: Diagnostic accuracy of ultrasound screening for fetal structural abnormalities during the first and second trimester of pregnancy in low‐risk and unselected populations. *Cochrane Database of Systematic Reviews* 2024(5). - Botelho FE, Flumignan RLG, Shiomatsu GY, de Castro-Santos G, Cacione DG, Leite JO, Baptista-Silva JCC: Preoperative coronary interventions for preventing acute myocardial infarction in the perioperative period of major open vascular or endovascular surgery. *Cochrane Database of Systematic Reviews* 2024(7). - Boehlke C, Joos L, Coune B, Becker C, Meerpohl JJ, Buroh S, Hercz D, Schwarzer G, Becker G: Pharmacological interventions for pruritus in adult palliative care patients. *Cochrane Database of Systematic Reviews* 2024(8). - Bijkerk V, Jacobs LMC, Albers KI, Gurusamy KS, van Laarhoven C, Keijzer C, Warlé MC: Deep neuromuscular blockade in adults undergoing an abdominal laparoscopic procedure. *Cochrane Database of Systematic Reviews* 2024(1). - Bertolini F, Robertson L, Bisson JI, Meader N, Churchill R, Ostuzzi G, Stein DJ, Williams T, Barbui C: Early pharmacological interventions for prevention of post‐traumatic stress disorder (PTSD) in individuals experiencing acute traumatic stress symptoms. *Cochrane Database of Systematic Reviews* 2024(5). - Armarego M, Forde H, Wills K, Beggs SA: High‐flow nasal cannula therapy for infants with bronchiolitis. *Cochrane Database of Systematic Reviews* 2024(3). - Andresen K, Rosenberg J: Transabdominal pre‐peritoneal (TAPP) versus totally extraperitoneal (TEP) laparoscopic techniques for inguinal hernia repair. *Cochrane Database of Systematic Reviews* 2024(7). - Abbasciano RG, Olivieri GM, Chubsey R, Gatta F, Tyson N, Easwarakumar K, Fudulu DP, Marsico R, Kofler M, Elshafie G *et al*: Prophylactic corticosteroids for cardiopulmonary bypass in adult cardiac surgery. *Cochrane Database of Systematic Reviews* 2024(3). - Zhu P, Dong S, Sun P, Belgaumkar AP, Sun Y, Cheng X, Zheng Q, Li T: Expanded polytetrafluoroethylene (ePTFE)‐covered stents versus bare stents for transjugular intrahepatic portosystemic shunt in people with liver cirrhosis. *Cochrane Database of Systematic Reviews* 2023(8). - Zhang L, Mendoza-Sassi RA, Wainwright CE, Aregbesola A, Klassen TP: Nebulised hypertonic saline solution for acute bronchiolitis in infants. *Cochrane Database of Systematic Reviews* 2023(4). - Zeleke M, Badanie A, Asefa ET, Reta Demissie W, Chala G, Aman H, Feyisa TO, Habte ML: Assessment of Electrocardiographic Changes and Associated Factors Among Thyroid Dysfunction Patients Attending Jimma Medical Center, Southwest Ethiopia: A Cross-Sectional Study. *Int J Gen Med* 2023, 16:2035-2046. - Yoshino CA, Sidney-Annerstedt K, Wingfield T, Kirubi B, Viney K, Boccia D, Atkins S: Experiences of conditional and unconditional cash transfers intended for improving health outcomes and health service use: a qualitative evidence synthesis. *Cochrane Database of Systematic Reviews* 2023(3). - Worthington HV, Bulsara VM, Glenny AM, Clarkson JE, Conway DI, Macluskey M: Interventions for the treatment of oral cavity and oropharyngeal cancers: surgical treatment. *Cochrane Database of Systematic Reviews* 2023(8). - Willis MA, Toews I, Soltau SLV, Kalff JC, Meerpohl JJ, Vilz TO: Preoperative combined mechanical and oral antibiotic bowel preparation for preventing complications in elective colorectal surgery. *Cochrane Database of Systematic Reviews* 2023(2). - Williamson A, Martineau AR, Sheikh A, Jolliffe D, Griffiths CJ: Vitamin D for the management of asthma. *Cochrane Database of Systematic Reviews* 2023(2). - Williams NC, Jayaratnasingam J, Prayle AP, Nevitt SJ, Smyth AR: Prebiotics for people with cystic fibrosis. *Cochrane Database of Systematic Reviews* 2023(9). - Williams G, Stothart CI, Hahn D, Stephens JH, Craig JC, Hodson EM: Cranberries for preventing urinary tract infections. *Cochrane Database of Systematic Reviews* 2023(11). - Williams CM, Henschke N, Maher CG, van Tulder MW, Koes BW, Macaskill P, Irwig L: Red flags to screen for vertebral fracture in patients presenting with low‐back pain. *Cochrane Database of Systematic Reviews* 2023(11). - Wilhelm K, Hein S, Kunath F, Schoenthaler M, Schmidt S: Totally tubeless, tubeless, and tubed percutaneous nephrolithotomy for treating kidney stones. *Cochrane Database of Systematic Reviews* 2023(7). - Wilfling D, Calo S, Dichter MN, Meyer G, Möhler R, Köpke S: Non‐pharmacological interventions for sleep disturbances in people with dementia. *Cochrane Database of Systematic Reviews* 2023(1). - Webster KE, George B, Lee A, Galbraith K, Harrington-Benton NA, Judd O, Kaski D, Maarsingh OR, MacKeith S, Murdin L *et al*: Lifestyle and dietary interventions for Ménière’s disease. *Cochrane Database of Systematic Reviews* 2023(2). - Webster KE, Galbraith K, Harrington-Benton NA, Judd O, Kaski D, Maarsingh OR, MacKeith S, Ray J, Van Vugt VA, Burton MJ: Systemic pharmacological interventions for Ménière’s disease. *Cochrane Database of Systematic Reviews* 2023(2). - Wang X, Ma Y, Hui X, Li M, Li J, Tian J, Wang Q, Yan P, Li J, Xie P *et al*: Oral direct thrombin inhibitors or oral factor Xa inhibitors versus conventional anticoagulants for the treatment of deep vein thrombosis. *Cochrane Database of Systematic Reviews* 2023(4). - Vita G, Compri B, Matcham F, Barbui C, Ostuzzi G: Antidepressants for the treatment of depression in people with cancer. *Cochrane Database of Systematic Reviews* 2023(3). - Veth VB, van de Kar MMA, Duffy JMN, van Wely M, Mijatovic V, Maas JWM: Gonadotropin‐releasing hormone analogues for endometriosis. *Cochrane Database of Systematic Reviews* 2023(6). - Velez M, Lugo-Agudelo LH, Patiño Lugo DF, Glenton C, Posada AM, Mesa Franco LF, Negrini S, Kiekens C, Spir Brunal MA, Roberg AS *et al*: Factors that influence the provision of home‐based rehabilitation services for people needing rehabilitation: a qualitative evidence synthesis. *Cochrane Database of Systematic Reviews* 2023(2). - Vandevenne MMS, Favuzza E, Veta M, Lucenteforte E, Berendschot T, Mencucci R, Nuijts R, Virgili G, Dickman MM: Artificial intelligence for detecting keratoconus. *Cochrane Database of Systematic Reviews* 2023(11). - van Geffen WH, Tan DJ, Walters JAE, Walters EH: Inhaled corticosteroids with combination inhaled long‐acting beta2‐agonists and long‐acting muscarinic antagonists for chronic obstructive pulmonary disease. *Cochrane Database of Systematic Reviews* 2023(12). - Tzoumas N, Riding G, Williams MA, Steel DHW: Complement inhibitors for age‐related macular degeneration. *Cochrane Database of Systematic Reviews* 2023(6). - Tunnicliffe DJ, Palmer SC, Cashmore BA, Saglimbene VM, Krishnasamy R, Lambert K, Johnson DW, Craig JC, Strippoli GFM: HMG CoA reductase inhibitors (statins) for people with chronic kidney disease not requiring dialysis. *Cochrane Database of Systematic Reviews* 2023(11). - Tramacere I, Virgili G, Perduca V, Lucenteforte E, Benedetti MD, Capobussi M, Castellini G, Frau S, Gonzalez-Lorenzo M, Featherstone R *et al*: Adverse effects of immunotherapies for multiple sclerosis: a network meta‐analysis. *Cochrane Database of Systematic Reviews* 2023(11). - Tian F, Jiang Q, Chen J, Liu Z: Silicone gel sheeting for treating keloid scars. *Cochrane Database of Systematic Reviews* 2023(1). - Tan HS, Zeng Y, Qi Y, Sultana R, Tan CW, Sia AT, Sng BL, Siddiqui FJ: Automated mandatory bolus versus basal infusion for maintenance of epidural analgesia in labour. *Cochrane Database of Systematic Reviews* 2023(6). - Tadayon Najafabadi B, Rayner DG, Shokraee K, Shokraie K, Panahi P, Rastgou P, Seirafianpour F, Momeni Landi F, Alinia P, Parnianfard N *et al*: Obesity as an independent risk factor for COVID‐19 severity and mortality. *Cochrane Database of Systematic Reviews* 2023(5). - Szafran A, Dahms K, Ansems K, Skoetz N, Monsef I, Breuer T, Benstoem C: Early versus late tracheostomy in critically ill COVID‐19 patients. *Cochrane Database of Systematic Reviews* 2023(11). - Storebø OJ, Storm MR, Pereira Ribeiro J, Skoog M, Groth C, Callesen HE, Schaug JP, Darling Rasmussen P, Huus CM, Zwi M *et al*: Methylphenidate for children and adolescents with attention deficit hyperactivity disorder (ADHD). *Cochrane Database of Systematic Reviews* 2023(3). - Stoniute A, Madhuvrata P, Still M, Barron-Millar E, Nabi G, Omar MI: Oral anticholinergic drugs versus placebo or no treatment for managing overactive bladder syndrome in adults. *Cochrane Database of Systematic Reviews* 2023(5). - Stanford G, Morrison L, Brown C: Nebuliser systems for drug delivery in cystic fibrosis. *Cochrane Database of Systematic Reviews* 2023(11). - Stafford IG, Lai NM, Tan K: Automated oxygen delivery for preterm infants with respiratory dysfunction. *Cochrane Database of Systematic Reviews* 2023(11). - Spurling GKP, Dooley L, Clark J, Askew DA: Immediate versus delayed versus no antibiotics for respiratory infections. *Cochrane Database of Systematic Reviews* 2023(10). - Soderberg L, Ergun O, Ding M, Parker R, Borofsky MS, Pais V, Dahm P: Percutaneous nephrolithotomy versus retrograde intrarenal surgery for treatment of renal stones in adults. *Cochrane Database of Systematic Reviews* 2023(11). - Smith TO, Gaukroger A, Metcalfe A, Hing CB: Surgical versus non‐surgical interventions for treating patellar dislocation. *Cochrane Database of Systematic Reviews* 2023(1). - Singh S, Kirtschig G, Anchan VN, Chi CC, Taghipour K, Boyle RJ, Murrell DF: Interventions for bullous pemphigoid. *Cochrane Database of Systematic Reviews* 2023(8). - Singh S, Keller PR, Busija L, McMillan P, Makrai E, Lawrenson JG, Hull CC, Downie LE: Blue‐light filtering spectacle lenses for visual performance, sleep, and macular health in adults. *Cochrane Database of Systematic Reviews* 2023(8). - Shaw V, Yu A, Parsons M, Olsen T, Walker C: Acute assessment services for patient flow assistance in hospital emergency departments. *Cochrane Database of Systematic Reviews* 2023(7). - Searle HKC, Lewis SR, Coyle C, Welch M, Griffin XL: Ultrasound and shockwave therapy for acute fractures in adults. *Cochrane Database of Systematic Reviews* 2023(3). - Sbidian E, Chaimani A, Guelimi R, Garcia-Doval I, Hua C, Hughes C, Naldi L, Kinberger M, Afach S, Le Cleach L: Systemic pharmacological treatments for chronic plaque psoriasis: a network meta‐analysis. *Cochrane Database of Systematic Reviews* 2023(7). - Sayed S, Ngugi AK, Nwosu N, Mutebi MC, Ochieng P, Mwenda AS, Salam RA: Training health workers in clinical breast examination for early detection of breast cancer in low‐ and middle‐income countries. *Cochrane Database of Systematic Reviews* 2023(4). - Sarpe AKP, Flumignan CDQ, Nakano LCU, Trevisani VFM, Lopes RD, Guedes Neto HJ, Flumignan RLG: Duplex ultrasound for surveillance of lower limb revascularisation. *Cochrane Database of Systematic Reviews* 2023(7). - Santos BC, Flumignan RLG, Civile VT, Atallah Á, Nakano LCU: Prophylactic anticoagulants for non‐hospitalised people with COVID‐19. *Cochrane Database of Systematic Reviews* 2023(8). - Sandford A, Haywood A, Rickett K, Good P, Khan S, Foster K, Hardy JR: Corticosteroids for the management of cancer‐related fatigue in adults with advanced cancer. *Cochrane Database of Systematic Reviews* 2023(1). - Sæter AH, Fonnes S, Li S, Rosenberg J, Andresen K: Mesh versus non‐mesh for emergency groin hernia repair. *Cochrane Database of Systematic Reviews* 2023(11). - Sadler E, Khadjesari Z, Ziemann A, Sheehan KJ, Whitney J, Wilson D, Bakolis I, Sevdalis N, Sandall J, Soukup T *et al*: Case management for integrated care of older people with frailty in community settings. *Cochrane Database of Systematic Reviews* 2023(5). - Rouse B, Le JT, Gazzard G: Iridotomy to slow progression of visual field loss in angle‐closure glaucoma. *Cochrane Database of Systematic Reviews* 2023(1). - Rosenberg JE, Ergun O, Hwang EC, Risk MC, Jung JH, Edwards ME, Blair Y, Dahm P: Non‐surgical therapies for Peyronie's disease. *Cochrane Database of Systematic Reviews* 2023(7). - Roqué-Figuls M, Giné-Garriga M, Granados Rugeles C, Perrotta C, Vilaró J: Chest physiotherapy for acute bronchiolitis in paediatric patients between 0 and 24 months old. *Cochrane Database of Systematic Reviews* 2023(4). - Riera R, Torloni MR, Martimbianco ALC, Pacheco RL: Alemtuzumab for multiple sclerosis. *Cochrane Database of Systematic Reviews* 2023(6). - Richter B, Bongaerts B, Metzendorf MI: Thermal stability and storage of human insulin. *Cochrane Database of Systematic Reviews* 2023(11). - Reis S, Metzendorf MI, Kuehn R, Popp M, Gagyor I, Kranke P, Meybohm P, Skoetz N, Weibel S: Nirmatrelvir combined with ritonavir for preventing and treating COVID‐19. *Cochrane Database of Systematic Reviews* 2023(11). - Reinhart M, Puil L, Salzwedel DM, Wright JM: First‐line diuretics versus other classes of antihypertensive drugs for hypertension. *Cochrane Database of Systematic Reviews* 2023(7). - Reeve K, On BI, Havla J, Burns J, Gosteli-Peter MA, Alabsawi A, Alayash Z, Götschi A, Seibold H, Mansmann U *et al*: Prognostic models for predicting clinical disease progression, worsening and activity in people with multiple sclerosis. *Cochrane Database of Systematic Reviews* 2023(9). - Ramanadhan S, Hansen K, Henderson JT, Cohen MA, Paynter R, Edelman A: Risk of thromboembolism in patients with COVID‐19 who are using hormonal contraception. *Cochrane Database of Systematic Reviews* 2023(5). - Purcell C, Dibben G, Hilton Boon M, Matthews L, Palmer VJ, Thomson M, Smillie S, Simpson SA, Taylor RS: Social network interventions to support cardiac rehabilitation and secondary prevention in the management of people with heart disease. *Cochrane Database of Systematic Reviews* 2023(6). - Prior D, Win S, Hassiotis A, Hall I, Martiello MA, Ali AK: Behavioural and cognitive‐behavioural interventions for outwardly directed aggressive behaviour in people with intellectual disabilities. *Cochrane Database of Systematic Reviews* 2023(2). - Prabhu AR, Rao IR, Nagaraju SP, Rajwar E, Venkatesh BT, Nair N S, Pai G, Reddy NP, Suvarna D: Interventions for dialysis patients with hepatitis C virus (HCV) infection. *Cochrane Database of Systematic Reviews* 2023(4). - Pickett CM, Seeratan DD, Mol BWJ, Nieboer TE, Johnson N, Bonestroo T, Aarts JWM: Surgical approach to hysterectomy for benign gynaecological disease. *Cochrane Database of Systematic Reviews* 2023(8). - Pessano S, Bruschettini M, Prescott M, Romantsik O: Positioning for lumbar puncture in newborn infants. *Cochrane Database of Systematic Reviews* 2023(12). - Persad E, Pizarro AB, Bruschettini M: Non‐opioid analgesics for procedural pain in neonates. *Cochrane Database of Systematic Reviews* 2023(4). - Pérez-Amate È, Roqué-Figuls M, Fernández-González M, Giné-Garriga M: Exercise interventions for adults after liver transplantation. *Cochrane Database of Systematic Reviews* 2023(5). - Panebianco M, Walker L, Marson AG: Immunomodulatory interventions for focal epilepsy. *Cochrane Database of Systematic Reviews* 2023(10). - Oyo-Ita A, Oduwole O, Arikpo D, Effa EE, Esu EB, Balakrishna Y, Chibuzor MT, Oringanje CM, Nwachukwu CE, Wiysonge CS *et al*: Interventions for improving coverage of childhood immunisation in low‐ and middle‐income countries. *Cochrane Database of Systematic Reviews* 2023(12). - Obi MF, Sharma M, Namireddy V, Gargiulo P, Noel C, Hyun C, Gale B: Variant of Wellen's syndrome in type 1 diabetic patient: A case report. *World J Cardiol* 2023, 15(9):462-468. - Newhouse R, Nelissen E, El-Shakankery KH, Rogozińska E, Bain E, Veiga S, Morrison J: Pegylated liposomal doxorubicin for relapsed epithelial ovarian cancer. *Cochrane Database of Systematic Reviews* 2023(7). - Neligan A, Adan G, Nevitt SJ, Pullen A, Sander JW, Bonnett L, Marson AG: Prognosis of adults and children following a first unprovoked seizure. *Cochrane Database of Systematic Reviews* 2023(1). - Natale P, Ju A, Strippoli GFM, Craig JC, Saglimbene VM, Unruh ML, Stallone G, Jaure A: Interventions for fatigue in people with kidney failure requiring dialysis. *Cochrane Database of Systematic Reviews* 2023(8). - Narayan A, Evans JR, O'Brart D, Bunce C, Gore DM, Day AC: Laser‐assisted cataract surgery versus standard ultrasound phacoemulsification cataract surgery. *Cochrane Database of Systematic Reviews* 2023(6). - Mulimani P, Abas ABL, Karanth L, Colombatti R, Kulkarni P: Treatment of dental and orthodontic complications in thalassaemia. *Cochrane Database of Systematic Reviews* 2023(2). - Motamedi MAK, Mak NT, Brown CJ, Raval MJ, Karimuddin AA, Giustini D, Phang P: Local versus radical surgery for early rectal cancer with or without neoadjuvant or adjuvant therapy. *Cochrane Database of Systematic Reviews* 2023(6). - Mota BS, Bevilacqua JLB, Barrett J, Ricci M, Munhoz AM, Filassi JR, Baracat E, Riera R: Skin‐sparing mastectomy for the treatment of breast cancer. *Cochrane Database of Systematic Reviews* 2023(3). - Morris NR, Kermeen FD, Jones AW, Lee JYT, Holland AE: Exercise‐based rehabilitation programmes for pulmonary hypertension. *Cochrane Database of Systematic Reviews* 2023(3). - Molenaar CJL, van Rooijen SJ, Fokkenrood HJP, Roumen RMH, Janssen L, Slooter GD: Prehabilitation versus no prehabilitation to improve functional capacity, reduce postoperative complications and improve quality of life in colorectal cancer surgery. *Cochrane Database of Systematic Reviews* 2023(5). - Möhler R, Richter T, Köpke S, Meyer G: Interventions for preventing and reducing the use of physical restraints for older people in all long‐term care settings. *Cochrane Database of Systematic Reviews* 2023(7). - Mohamed Z, Varghese C, Sudhakar A, Kumar L, Gopalakrishnan U, Balakrishnan D, Narayanamenon R, Sudhindran S: Prostaglandins for adult liver transplanted recipients. *Cochrane Database of Systematic Reviews* 2023(8). - Miranda F, Gonzalez F, Plana MN, Zamora J, Quinn TJ, Seron P: Confusion Assessment Method for the Intensive Care Unit (CAM‐ICU) for the diagnosis of delirium in adults in critical care settings. *Cochrane Database of Systematic Reviews* 2023(11). - Merner B, Schonfeld L, Virgona A, Lowe D, Walsh L, Wardrope C, Graham-Wisener L, Xafis V, Colombo C, Refahi N *et al*: Consumers’ and health providers’ views and perceptions of partnering to improve health services design, delivery and evaluation: a co‐produced qualitative evidence synthesis. *Cochrane Database of Systematic Reviews* 2023(3). - McDonagh STJ, Dalal H, Moore S, Clark CE, Dean SG, Jolly K, Cowie A, Afzal J, Taylor RS: Home‐based versus centre‐based cardiac rehabilitation. *Cochrane Database of Systematic Reviews* 2023(10). - Mateos-Haro M, Novoa-Candia M, Sánchez Vanegas G, Correa-Pérez A, Gaetano Gil A, Fernández-García S, Ortega-Quijano D, Urueña Rodriguez MG, Saceda-Corralo D, Bennouna-Dalero T *et al*: Treatments for alopecia areata: a network meta‐analysis. *Cochrane Database of Systematic Reviews* 2023(10). - Martinez-Zapata MJ, Salvador I, Martí-Carvajal AJ, Pijoan JI, Cordero JA, Ponomarev D, Kernohan A, Solà I, Virgili G: Anti‐vascular endothelial growth factor for proliferative diabetic retinopathy. *Cochrane Database of Systematic Reviews* 2023(3). - Martin C, Littlewood SJ, Millett DT, Doubleday B, Bearn D, Worthington HV, Limones A: Retention procedures for stabilising tooth position after treatment with orthodontic braces. *Cochrane Database of Systematic Reviews* 2023(5). - Maltez N, Maxwell LJ, Rirash F, Tanjong Ghogomu E, Harding SE, Tingey PC, Wells GA, Tugwell P, Pope J: Phosphodiesterase 5 inhibitors (PDE5i) for the treatment of Raynaud's phenomenon. *Cochrane Database of Systematic Reviews* 2023(11). - Malik AK, Amer AO, Tingle SJ, Thompson ER, White SA, Manas DM, Wilson C: Fibrin‐based haemostatic agents for reducing blood loss in adult liver resection. *Cochrane Database of Systematic Reviews* 2023(8). - MacKeith S, Mulvaney CA, Galbraith K, Webster KE, Paing A, Connolly R, Marom T, Daniel M, Venekamp RP, Schilder AGM: Adenoidectomy for otitis media with effusion (OME) in children. *Cochrane Database of Systematic Reviews* 2023(10). - Lynch EA, Bulto LN, Cheng H, Craig L, Luker JA, Bagot KL, Thayabaranathan T, Janssen H, McInnes E, Middleton S *et al*: Interventions for the uptake of evidence‐based recommendations in acute stroke settings. *Cochrane Database of Systematic Reviews* 2023(8). - Lühnen J, Richter T, Calo S, Meyer G, Köpke S, Möhler R: Psychosocial interventions for reducing antipsychotic medication in care home residents. *Cochrane Database of Systematic Reviews* 2023(8). - Lourijsen E, Avdeeva K, Gan KL, Pundir V, Fokkens W: Tranexamic acid for the reduction of bleeding during functional endoscopic sinus surgery. *Cochrane Database of Systematic Reviews* 2023(2). - Lloyd TD, Geneen LJ, Bernhardt K, McClune W, Fernquest SJ, Brown T, Dorée C, Brunskill SJ, Murphy MF, Palmer AJR: Cell salvage for minimising perioperative allogeneic blood transfusion in adults undergoing elective surgery. *Cochrane Database of Systematic Reviews* 2023(9). - Lin ZC, Bennett MH, Hawkins GC, Azzopardi CP, Feldmeier J, Smee R, Milross C: Hyperbaric oxygen therapy for late radiation tissue injury. *Cochrane Database of Systematic Reviews* 2023(8). - Liang F, Liu S, Liu G, Liu H, Wang Q, Song B, Yao L: Remote ischaemic preconditioning versus no remote ischaemic preconditioning for vascular and endovascular surgical procedures. *Cochrane Database of Systematic Reviews* 2023(1). - Lewis SR, Pritchard MW, Solomon JL, Griffin XL, Bruce J: Surgical versus non‐surgical interventions for displaced intra‐articular calcaneal fractures. *Cochrane Database of Systematic Reviews* 2023(11). - Lewis SR, Pritchard MW, Estcourt LJ, Stanworth SJ, Griffin XL: Interventions for reducing red blood cell transfusion in adults undergoing hip fracture surgery: an overview of systematic reviews. *Cochrane Database of Systematic Reviews* 2023(6). - Lemyre B, Deguise MO, Benson P, Kirpalani H, Ekhaguere OA, Davis PG: Early nasal intermittent positive pressure ventilation (NIPPV) versus early nasal continuous positive airway pressure (NCPAP) for preterm infants. *Cochrane Database of Systematic Reviews* 2023(7). - Lee SW, Chen WS, Sellappans R, Md.Sharif SB, Metzendorf MI, Lai NM: Interventions for people with type 2 diabetes mellitus fasting during Ramadan. *Cochrane Database of Systematic Reviews* 2023(7). - Lee S, You C, Kucey A, Alam F, Papia G, Kucey DS, Forbes T, Choi S, Dueck AD, Kayssi A: General versus loco‐regional anesthesia for endovascular aortic aneurysm repair. *Cochrane Database of Systematic Reviews* 2023(4). - Lee A, Webster KE, George B, Harrington-Benton NA, Judd O, Kaski D, Maarsingh OR, MacKeith S, Ray J, Van Vugt VA *et al*: Surgical interventions for Ménière’s disease. *Cochrane Database of Systematic Reviews* 2023(2). - Laloo R, Dewi M, Gwilym BL, Richards OJ, McLain AD, Bosanquet D: Tourniquet use for people with peripheral arterial disease undergoing major lower limb amputations. *Cochrane Database of Systematic Reviews* 2023(7). - Lai M, Zhou S, He S, Cheng Y, Cheng N, Deng Y, Ding X: Fibrin sealants for the prevention of postoperative pancreatic fistula following pancreatic surgery. *Cochrane Database of Systematic Reviews* 2023(6). - Konnyu KJ, Yogasingam S, Lépine J, Sullivan K, Alabousi M, Edwards A, Hillmer M, Karunananthan S, Lavis JN, Linklater S *et al*: Quality improvement strategies for diabetes care: Effects on outcomes for adults living with diabetes. *Cochrane Database of Systematic Reviews* 2023(5). - Klitgaard TL, Schjørring OL, Nielsen FM, Meyhoff CS, Perner A, Wetterslev J, Rasmussen BS, Barbateskovic M: Higher versus lower fractions of inspired oxygen or targets of arterial oxygenation for adults admitted to the intensive care unit. *Cochrane Database of Systematic Reviews* 2023(9). - Kirmani BH, Jones SG, Muir A, Malaisrie SC, Chung DA, Williams R, Akowuah E: Limited versus full sternotomy for aortic valve replacement. *Cochrane Database of Systematic Reviews* 2023(12). - Kinoshita M, Stempel KS, Borges do Nascimento IJ, Bruschettini M: Systemic opioids versus other analgesics and sedatives for postoperative pain in neonates. *Cochrane Database of Systematic Reviews* 2023(3). - Kimber C, Valk SJ, Chai KL, Piechotta V, Iannizzi C, Monsef I, Wood EM, Lamikanra AA, Roberts DJ, McQuilten Z *et al*: Hyperimmune immunoglobulin for people with COVID‐19. *Cochrane Database of Systematic Reviews* 2023(1). - Kim S, Kong JH, Lee Y, Lee JY, Kang TW, Kong TH, Kim MH, You SH: Dose‐escalated radiotherapy for clinically localized and locally advanced prostate cancer. *Cochrane Database of Systematic Reviews* 2023(3). - Ki S, Lee SJ, Oh J, Cho SB, Park S, An T, Lee J: Asymptomatic Type 2 Perioperative Myocardial Infarction Detected before Anesthetic Induction in a Patient Undergoing Non-Cardiac Surgery-A Case Report. *Medicina (Kaunas)* 2023, 59(6). - Karjalainen TV, Lusa V, Page MJ, O'Connor D, Massy-Westropp N, Peters SE: Splinting for carpal tunnel syndrome. *Cochrane Database of Systematic Reviews* 2023(2). - Jones M, Moffatt F, Harvey A, Ryan JM: Interventions for improving adherence to airway clearance treatment and exercise in people with cystic fibrosis. *Cochrane Database of Systematic Reviews* 2023(7). - Jiang S, Fang J, Li W: Protein restriction for diabetic kidney disease. *Cochrane Database of Systematic Reviews* 2023(1). - Jefferson T, Dooley L, Ferroni E, Al-Ansary LA, van Driel ML, Bawazeer GA, Jones MA, Hoffmann TC, Clark J, Beller EM *et al*: Physical interventions to interrupt or reduce the spread of respiratory viruses. *Cochrane Database of Systematic Reviews* 2023(1). - Jassim GA, Doherty S, Whitford DL, Khashan AS: Psychological interventions for women with non‐metastatic breast cancer. *Cochrane Database of Systematic Reviews* 2023(1). - Imdad A, Pandit NG, Zaman M, Minkoff NZ, Tanner-Smith EE, Gomez-Duarte OG, Acra S, Nicholson MR: Fecal transplantation for treatment of inflammatory bowel disease. *Cochrane Database of Systematic Reviews* 2023(4). - Ijzelenberg W, Oosterhuis T, Hayden JA, Koes BW, van Tulder MW, Rubinstein SM, de Zoete A: Exercise therapy for treatment of acute non‐specific low back pain. *Cochrane Database of Systematic Reviews* 2023(8). - Iannizzi C, Chai KL, Piechotta V, Valk SJ, Kimber C, Monsef I, Wood EM, Lamikanra AA, Roberts DJ, McQuilten Z *et al*: Convalescent plasma for people with COVID‐19: a living systematic review. *Cochrane Database of Systematic Reviews* 2023(5). - Hodson EM, Cooper TE: Altered dietary salt intake for preventing diabetic kidney disease and its progression. *Cochrane Database of Systematic Reviews* 2023(1). - Heneghan M, Southern KW, Murphy J, Sinha IP, Nevitt SJ: Corrector therapies (with or without potentiators) for people with cystic fibrosis with class II CFTR gene variants (most commonly F508del). *Cochrane Database of Systematic Reviews* 2023(11). - Hedin K, Thorning S, van Driel ML: Different antibiotic treatments for group A streptococcal pharyngitis. *Cochrane Database of Systematic Reviews* 2023(11). - Hay S, Ovelman C, Zupancic JAF, Doyle LW, Onland W, Konstantinidis M, Shah PS, Soll R: Systemic corticosteroids for the prevention of bronchopulmonary dysplasia, a network meta‐analysis. *Cochrane Database of Systematic Reviews* 2023(8). - Haworth K, Travis D, Leslie L, Fuller D, Pucker AD: Silicone hydrogel versus hydrogel soft contact lenses for differences in patient‐reported eye comfort and safety. *Cochrane Database of Systematic Reviews* 2023(9). - Häuser W, Welsch P, Radbruch L, Fisher E, Bell RF, Moore RA: Cannabis‐based medicines and medical cannabis for adults with cancer pain. *Cochrane Database of Systematic Reviews* 2023(6). - Hanna M, Shah R, Marquez L, Barzegar R, Gordon A, Pammi M: Infant isolation and cohorting for preventing or reducing transmission of healthcare‐associated infections in neonatal units. *Cochrane Database of Systematic Reviews* 2023(6). - Han CS, Hancock MJ, Downie A, Jarvik JG, Koes BW, Machado GC, Verhagen AP, Williams CM, Chen Q, Maher CG: Red flags to screen for vertebral fracture in people presenting with low back pain. *Cochrane Database of Systematic Reviews* 2023(8). - Hahn D, Hodson EM, Craig JC: Interventions for preventing and treating kidney disease in IgA vasculitis. *Cochrane Database of Systematic Reviews* 2023(2). - Gutiérrez-Díaz GI, Buenrostro-Jiménez AD, Rojas-Castillo R, Amador-Avendaño V, Jaime-Zúñiga AY, Zambada-Gamboa AJ, Velázquez-García MA, Gudiño-Amezcua DA: [Acute coronary syndrome provoked by a coronary intramural hematoma]. *Rev Med Inst Mex Seguro Soc* 2023, 61(3):380-385. - Guaiana G, Meader N, Barbui C, Davies SJC, Furukawa TA, Imai H, Dias S, Caldwell DM, Koesters M, Tajika A *et al*: Pharmacological treatments in panic disorder in adults: a network meta‐analysis. *Cochrane Database of Systematic Reviews* 2023(11). - Grundeis F, Ansems K, Dahms K, Thieme V, Metzendorf MI, Skoetz N, Benstoem C, Mikolajewska A, Griesel M, Fichtner F *et al*: Remdesivir for the treatment of COVID‐19. *Cochrane Database of Systematic Reviews* 2023(1). - Greene MC, Kane J, Alto M, Giusto A, Lovero K, Stockton M, McClendon J, Nicholson T, Wainberg ML, Johnson RM *et al*: Psychosocial and pharmacologic interventions to reduce harmful alcohol use in low‐ and middle‐income countries. *Cochrane Database of Systematic Reviews* 2023(5). - Gottlieb M, Peksa GD, Carlson JN: Head impulse, nystagmus, and test of skew examination for diagnosing central causes of acute vestibular syndrome. *Cochrane Database of Systematic Reviews* 2023(11). - Gorry C, McCullagh L, O'Donnell H, Barrett S, Schmitz S, Barry M, Curtin K, Beausang E, Barry R, Coyne I: Neoadjuvant treatment for stage III and IV cutaneous melanoma. *Cochrane Database of Systematic Reviews* 2023(1). - Gordon M, Wallace C, Sinopoulou V, Akobeng AK: Probiotics for management of functional abdominal pain disorders in children. *Cochrane Database of Systematic Reviews* 2023(2). - Gordon M, Sinopoulou V, Lakunina S, Gjuladin-Hellon T, Bracewell K, Akobeng AK: Remote care through telehealth for people with inflammatory bowel disease. *Cochrane Database of Systematic Reviews* 2023(5). - Gordon M, Sinopoulou V, Ibrahim U, Abdulshafea M, Bracewell K, Akobeng AK: Patient education interventions for the management of inflammatory bowel disease. *Cochrane Database of Systematic Reviews* 2023(5). - Goodfellow M, Thompson ER, Tingle SJ, Wilson C: Early versus late removal of urinary catheter after kidney transplantation. *Cochrane Database of Systematic Reviews* 2023(7). - Glujovsky D, Pesce R, Miguens M, Sueldo C, Ciapponi A: Progestogens for prevention of luteinising hormone (LH) surge in women undergoing controlled ovarian hyperstimulation as part of an assisted reproductive technology (ART) cycle. *Cochrane Database of Systematic Reviews* 2023(11). - Gijtenbeek RGP, de Jong K, Venmans BJW, van Vollenhoven FHM, Ten Brinke A, Van der Wekken AJ, van Geffen WH: Best first‐line therapy for people with advanced non‐small cell lung cancer, performance status 2 without a targetable mutation or with an unknown mutation status. *Cochrane Database of Systematic Reviews* 2023(7). - Gibbs VN, Geneen LJ, Champaneria R, Raval P, Dorée C, Brunskill SJ, Novak A, Palmer AJR, Estcourt LJ: Pharmacological interventions for the prevention of bleeding in people undergoing definitive fixation or joint replacement for hip, pelvic and long bone fractures. *Cochrane Database of Systematic Reviews* 2023(6). - Ghosn L, Assi R, Evrenoglou T, Buckley BS, Henschke N, Probyn K, Riveros C, Davidson M, Graña C, Bonnet H *et al*: Interleukin‐6 blocking agents for treating COVID‐19: a living systematic review. *Cochrane Database of Systematic Reviews* 2023(6). - Gaitskell K, Rogozińska E, Platt S, Chen Y, Abd El Aziz M, Tattersall A, Morrison J: Angiogenesis inhibitors for the treatment of epithelial ovarian cancer. *Cochrane Database of Systematic Reviews* 2023(4). - Funada S, Yoshioka T, Luo Y, Sato A, Akamatsu S, Watanabe N: Bladder training for treating overactive bladder in adults. *Cochrane Database of Systematic Reviews* 2023(10). - Fulone I, Cadogan C, Barberato-Filho S, Bergamaschi CC, Mazzei LG, Lopes LP, Silva MT, Lopes LC: Pharmaceutical policies: effects of policies regulating drug marketing. *Cochrane Database of Systematic Reviews* 2023(6). - Fukuda N, Horita N, Kaneko A, Goto A, Kaneko T, Ota E, Kew KM: Long‐acting muscarinic antagonist (LAMA) plus long‐acting beta‐agonist (LABA) versus LABA plus inhaled corticosteroid (ICS) for stable chronic obstructive pulmonary disease. *Cochrane Database of Systematic Reviews* 2023(6). - Franciosi JP, Gordon M, Sinopoulou V, Dellon ES, Gupta SK, Reed CC, Gutiérrez-Junquera C, Venkatesh RD, Erwin EA, Egiz A *et al*: Medical treatment of eosinophilic esophagitis. *Cochrane Database of Systematic Reviews* 2023(7). - France E, Uny I, Turley R, Thomson K, Noyes J, Jordan A, Forbat L, Caes L, Silveira Bianchim M: A meta‐ethnography of how children and young people with chronic non‐cancer pain and their families experience and understand their condition, pain services, and treatments. *Cochrane Database of Systematic Reviews* 2023(10). - Fox T, Hunt BJ, Ariens RAS, Towers GJ, Lever R, Garner P, Kuehn R: Plasmapheresis to remove amyloid fibrin(ogen) particles for treating the post‐COVID‐19 condition. *Cochrane Database of Systematic Reviews* 2023(7). - Fox T, Gould S, Princy N, Rowland T, Lutje V, Kuehn R: Therapeutics for treating mpox in humans. *Cochrane Database of Systematic Reviews* 2023(3). - Fischer AL, Messer S, Riera R, Martimbianco ALC, Stegemann M, Estcourt LJ, Weibel S, Monsef I, Andreas M, Pacheco RL *et al*: Antiplatelet agents for the treatment of adults with COVID‐19. *Cochrane Database of Systematic Reviews* 2023(7). - El-Angbawi A, McIntyre G, Fleming PS, Bearn D: Non‐surgical adjunctive interventions for accelerating tooth movement in patients undergoing orthodontic treatment. *Cochrane Database of Systematic Reviews* 2023(6). - Edwards PJ, Roberts I, Clarke MJ, DiGuiseppi C, Woolf B, Perkins C: Methods to increase response to postal and electronic questionnaires. *Cochrane Database of Systematic Reviews* 2023(11). - Dopper A, Steele M, Bogossian F, Hough J: High flow nasal cannula for respiratory support in term infants. *Cochrane Database of Systematic Reviews* 2023(8). - Do DV, Han G, Abariga SA, Sleilati G, Vedula SS, Hawkins BS: Blood pressure control for diabetic retinopathy. *Cochrane Database of Systematic Reviews* 2023(3). - de Oliveira Gomes J, Gagliardi AMZ, Andriolo BNG, Torloni MR, Andriolo RB, Puga ME, Canteiro Cruz E: Vaccines for preventing herpes zoster in older adults. *Cochrane Database of Systematic Reviews* 2023(10). - Dawson S, Girling CJ, Cowap L, Clark-Carter D: Psychological interventions for improving adherence to inhaled therapies in people with cystic fibrosis. *Cochrane Database of Systematic Reviews* 2023(3). - Cundy O, Lange CAK, Bunce C, Bainbridge JW, Solebo AL: Face‐down positioning or posturing after macular hole surgery. *Cochrane Database of Systematic Reviews* 2023(11). - Cooper TE, Teng C, Tunnicliffe DJ, Cashmore BA, Strippoli GFM: Angiotensin‐converting enzyme inhibitors and angiotensin receptor blockers for adults with early (stage 1 to 3) non‐diabetic chronic kidney disease. *Cochrane Database of Systematic Reviews* 2023(7). - Cooper TE, Khalid R, Chan S, Craig JC, Hawley CM, Howell M, Johnson DW, Jaure A, Teixeira-Pinto A, Wong G: Synbiotics, prebiotics and probiotics for people with chronic kidney disease. *Cochrane Database of Systematic Reviews* 2023(10). - Colombijn JMT, Hooft L, Jun M, Webster AC, Bots ML, Verhaar MC, Vernooij RWM: Antioxidants for adults with chronic kidney disease. *Cochrane Database of Systematic Reviews* 2023(11). - Cole JA, Gonçalves-Bradley DC, Alqahtani M, Barry HE, Cadogan C, Rankin A, Patterson SM, Kerse N, Cardwell CR, Ryan C *et al*: Interventions to improve the appropriate use of polypharmacy for older people. *Cochrane Database of Systematic Reviews* 2023(10). - Cochrane A, Chen C, Stephen J, Rønning OM, Anderson CS, Hankey GJ, Al-Shahi Salman R: Antithrombotic treatment after stroke due to intracerebral haemorrhage. *Cochrane Database of Systematic Reviews* 2023(1). - Clezar CNB, Flumignan CDQ, Cassola N, Nakano LCU, Trevisani VFM, Flumignan RLG: Pharmacological interventions for asymptomatic carotid stenosis. *Cochrane Database of Systematic Reviews* 2023(8). - Cleveland B, Norling B, Wang H, Gandhi V, Price CL, Borofsky MS, Pais V, Dahm P: Tranexamic acid for percutaneous nephrolithotomy. *Cochrane Database of Systematic Reviews* 2023(10). - Chen YY, Liu AH, Nurmatov U, van Schayck OCP, Kuo IC: Antibiotics versus placebo for acute bacterial conjunctivitis. *Cochrane Database of Systematic Reviews* 2023(3). - Chakupurakal G, Freudenberger P, Skoetz N, Ahr H, Theurich S: Polyclonal anti‐thymocyte globulins for the prophylaxis of graft‐versus‐host disease after allogeneic stem cell or bone marrow transplantation in adults. *Cochrane Database of Systematic Reviews* 2023(6). - Cashin AG, Wand BM, O'Connell NE, Lee H, Rizzo RRN, Bagg MK, O'Hagan E, Maher CG, Furlan AD, van Tulder MW *et al*: Pharmacological treatments for low back pain in adults: an overview of Cochrane Reviews. *Cochrane Database of Systematic Reviews* 2023(4). - Carter E, Johnson EE, Still M, Al-Assaf AS, Bryant A, Aluko P, Jeffery ST, Nambiar A: Single‐incision sling operations for urinary incontinence in women. *Cochrane Database of Systematic Reviews* 2023(10). - Caro P, Turner W, Caldwell DM, Macdonald G: Comparative effectiveness of psychological interventions for treating the psychological consequences of sexual abuse in children and adolescents: a network meta‐analysis. *Cochrane Database of Systematic Reviews* 2023(6). - Cai PL, Hitchman LH, Mohamed AH, Smith GE, Chetter I, Carradice D: Endovenous ablation for venous leg ulcers. *Cochrane Database of Systematic Reviews* 2023(7). - Burrell A, Kim J, Alliegro P, Romero L, Serpa Neto A, Mariajoseph F, Hodgson C: Extracorporeal membrane oxygenation for critically ill adults. *Cochrane Database of Systematic Reviews* 2023(9). - Bülow C, Clausen SS, Lundh A, Christensen M: Medication review in hospitalised patients to reduce morbidity and mortality. *Cochrane Database of Systematic Reviews* 2023(1). - Buchan EJ, Haywood A, Syrmis W, Good P: Medically assisted hydration for adults receiving palliative care. *Cochrane Database of Systematic Reviews* 2023(12). - Broderick C, Kobayashi S, Suto M, Ito S, Kobayashi T: Intravenous immunoglobulin for the treatment of Kawasaki disease. *Cochrane Database of Systematic Reviews* 2023(1). - Briggs VR, Jacques RM, Fotheringham J, Maheswaran R, Campbell M, Wilkie ME: Catheter insertion techniques for improving catheter function and clinical outcomes in peritoneal dialysis patients. *Cochrane Database of Systematic Reviews* 2023(2). - Bray JJH, Warraich M, Whitfield MG, Peter CU, Baral R, Ahmad M, Ahmad S, Abraham GR, Kirresh A, Sahibzada MS *et al*: Oral Class I and III antiarrhythmic drugs for maintaining sinus rhythm after catheter ablation of atrial fibrillation. *Cochrane Database of Systematic Reviews* 2023(3). - Bittner AK, Yoshinaga PD, Rittiphairoj T, Li T: Telerehabilitation for people with low vision. *Cochrane Database of Systematic Reviews* 2023(1). - Birkinshaw H, Friedrich CM, Cole P, Eccleston C, Serfaty M, Stewart G, White S, Moore RA, Phillippo D, Pincus T: Antidepressants for pain management in adults with chronic pain: a network meta‐analysis. *Cochrane Database of Systematic Reviews* 2023(5). - Beverly A, Ong G, Kimber C, Sandercock J, Dorée C, Welton NJ, Wicks P, Estcourt LJ: Drugs to reduce bleeding and transfusion in major open vascular or endovascular surgery: a systematic review and network meta‐analysis. *Cochrane Database of Systematic Reviews* 2023(2). - Ballesteros J, Moreno-Calvete MC, Santos-Zorrozúa B, González-Fraile E: Cognitive behavioural therapy plus standard care versus standard care for persistent aggressive behaviour or agitation in people with schizophrenia. *Cochrane Database of Systematic Reviews* 2023(7). - Bai X, Zhang X, Gong H, Wang T, Wang X, Wang W, Yang K, Yang W, Feng Y, Ma Y *et al*: Different types of percutaneous endovascular interventions for acute ischemic stroke. *Cochrane Database of Systematic Reviews* 2023(5). - B Aledi L, Flumignan CDQ, Trevisani VFM, Miranda Jr F: Interventions for motor rehabilitation in people with transtibial amputation due to peripheral arterial disease or diabetes. *Cochrane Database of Systematic Reviews* 2023(6). - Aregbesola A, Tam CM, Kothari A, Le ML, Ragheb M, Klassen TP: Glucocorticoids for croup in children. *Cochrane Database of Systematic Reviews* 2023(1). - Ammous O, Feki W, Lotfi T, Khamis AM, Gosselink R, Rebai A, Kammoun S: Inspiratory muscle training, with or without concomitant pulmonary rehabilitation, for chronic obstructive pulmonary disease (COPD). *Cochrane Database of Systematic Reviews* 2023(1). - Ameratunga D, Yazdani A, Kroon B: Antibiotics prior to or at the time of embryo transfer in ART. *Cochrane Database of Systematic Reviews* 2023(11). - Aldin A, Besiroglu B, Adams A, Monsef I, Piechotta V, Tomlinson E, Hornbach C, Dressen N, Goldkuhle M, Maisch P *et al*: First‐line therapy for adults with advanced renal cell carcinoma: a systematic review and network meta‐analysis. *Cochrane Database of Systematic Reviews* 2023(5). - Aguiar Mesquita Galdino G, Eduardo Virgilio Silva L, Cristina Garcia Moura-Tonello S, Cristina Milan-Mattos J, Nogueira Linares S, Porta A, Marques da Silva T, Fazan R, Jr., Beltrame T, Maria Catai A: Heart rate fragmentation is impaired in type 2 diabetes mellitus patients. *Diabetes Res Clin Pract* 2023, 196:110223. - Agnew H, Kitson S, Crosbie EJ: Interventions for weight reduction in obesity to improve survival in women with endometrial cancer. *Cochrane Database of Systematic Reviews* 2023(3). - Aggarwal A, Adie S, Harris IA, Naylor J: Cryotherapy following total knee replacement. *Cochrane Database of Systematic Reviews* 2023(9). - Agabio R, Saulle R, Rösner S, Minozzi S: Baclofen for alcohol use disorder. *Cochrane Database of Systematic Reviews* 2023(1). - Afzali Rubin M, Svensson TLG, Herling SF, Jabre P, Møller AM: Family presence during resuscitation. *Cochrane Database of Systematic Reviews* 2023(5). - Abbasciano RG, Tomassini S, Roman MA, Rizzello A, Pathak S, Ramzi J, Lucarelli C, Layton G, Butt A, Lai F *et al*: Effects of interventions targeting the systemic inflammatory response to cardiac surgery on clinical outcomes in adults. *Cochrane Database of Systematic Reviews* 2023(10). - Zhu J, Chen N, Zhou M, Guo J, Zhu C, Zhou J, Ma M, He L: Calcium channel blockers versus other classes of drugs for hypertension. *Cochrane Database of Systematic Reviews* 2022(1). - Zhao M, Song L, Zhao Q, Chen Y, Li B, Xie Z, Fu Z, Zhang N, Cheng X, Li X: Elevated levels of body mass index and waist circumference, but not high variability, are associated with an increased risk of atrial fibrillation. *BMC medicine* 2022, 20(1):215. - Zhang J, Zhou K, Shan D, Luo X: Medical methods for first trimester abortion. *Cochrane Database of Systematic Reviews* 2022(5). - Zhang J, Ma B, Han X, Ding S, Li Y: Global, regional, and national burdens of HIV and other sexually transmitted infections in adolescents and young adults aged 10–24 years from 1990 to 2019: a trend analysis based on the Global Burden of Disease Study 2019. *The Lancet Child & Adolescent Health* 2022, 6(11):763-776. - Yoo HHB, Nunes-Nogueira VS, Fortes Villas Boas PJ, Broderick C: Outpatient versus inpatient treatment for acute pulmonary embolism. *Cochrane Database of Systematic Reviews* 2022(5). - Yin X, Tian F, Wu B, Xu T: Interventions for reducing inflammation in familial Mediterranean fever. *Cochrane Database of Systematic Reviews* 2022(3). - Xu R, Zhang X, Liu S, Wang X, Wang W, Yang K, Wang T, Dmytriw AA, Bai X, Ma Y *et al*: Percutaneous transluminal angioplasty and stenting for vertebral artery stenosis. *Cochrane Database of Systematic Reviews* 2022(5). - Wingert AM, Liu SH, Lin JC, Sridhar J: Non‐steroidal anti‐inflammatory agents for treating cystoid macular edema following cataract surgery. *Cochrane Database of Systematic Reviews* 2022(12). - Wagner C, Griesel M, Mikolajewska A, Metzendorf MI, Fischer AL, Stegemann M, Spagl M, Nair A, Daniel J, Fichtner F *et al*: Systemic corticosteroids for the treatment of COVID‐19: Equity‐related analyses and update on evidence. *Cochrane Database of Systematic Reviews* 2022(11). - Uneno Y, Imura H, Makuuchi Y, Tochitani K, Watanabe N: Pre‐emptive antifungal therapy versus empirical antifungal therapy for febrile neutropenia in people with cancer. *Cochrane Database of Systematic Reviews* 2022(11). - Tsujimoto Y, Kumasawa J, Shimizu S, Nakano Y, Kataoka Y, Tsujimoto H, Kono M, Okabayashi S, Imura H, Mizuta T: Doppler trans‐thoracic echocardiography for detection of pulmonary hypertension in adults. *Cochrane Database of Systematic Reviews* 2022(5). - Tse F, Liu J, Yuan Y, Moayyedi P, Leontiadis GI: Guidewire‐assisted cannulation of the common bile duct for the prevention of post‐endoscopic retrograde cholangiopancreatography (ERCP) pancreatitis. *Cochrane Database of Systematic Reviews* 2022(3). - Tosello R, Riera R, Tosello G, Clezar CNB, Amorim JE, Vasconcelos V, Joao BB, Flumignan RLG: Type of anaesthesia for acute ischaemic stroke endovascular treatment. *Cochrane Database of Systematic Reviews* 2022(7). - Todhunter-Brown A, Hazelton C, Campbell P, Elders A, Hagen S, McClurg D: Conservative interventions for treating urinary incontinence in women: an Overview of Cochrane systematic reviews. *Cochrane Database of Systematic Reviews* 2022(9). - Thomson SE, Ng NYB, Riehle MO, Kingham PJ, Dahlin LB, Wiberg M, Hart AM: Bioengineered nerve conduits and wraps for peripheral nerve repair of the upper limb. *Cochrane Database of Systematic Reviews* 2022(12). - Tattersall A, Ryan N, Wiggans AJ, Rogozińska E, Morrison J: Poly(ADP‐ribose) polymerase (PARP) inhibitors for the treatment of ovarian cancer. *Cochrane Database of Systematic Reviews* 2022(2). - Tada M, Yamada N, Matsumoto T, Takeda C, Furukawa TA, Watanabe N: Ultrasound guidance versus landmark method for peripheral venous cannulation in adults. *Cochrane Database of Systematic Reviews* 2022(12). - Sultan S, Concannon J, Veerasingam D, Tawfick W, McHugh P, Jordan F, Hynes N: Endovascular versus conventional open surgical repair for thoracoabdominal aortic aneurysms. *Cochrane Database of Systematic Reviews* 2022(4). - Struyf T, Deeks JJ, Dinnes J, Takwoingi Y, Davenport C, Leeflang MMG, Spijker R, Hooft L, Emperador D, Domen J *et al*: Signs and symptoms to determine if a patient presenting in primary care or hospital outpatient settings has COVID‐19. *Cochrane Database of Systematic Reviews* 2022(5). - Srivastava M, Harrison N, Caetano A, Tan AR, Law M: Ultrafiltration for acute heart failure. *Cochrane Database of Systematic Reviews* 2022(1). - Sothornwit J, Kaewrudee S, Lumbiganon P, Pattanittum P, Averbach SH: Immediate versus delayed postpartum insertion of contraceptive implant and IUD for contraception. *Cochrane Database of Systematic Reviews* 2022(10). - Smith R, Villanueva G, Probyn K, Sguassero Y, Ford N, Orrell C, Cohen K, Chaplin M, Leeflang MMG, Hine P: Accuracy of measures for antiretroviral adherence in people living with HIV. *Cochrane Database of Systematic Reviews* 2022(7). - Smedemark SA, Aabenhus R, Llor C, Fournaise A, Olsen O, Jørgensen KJ: Biomarkers as point‐of‐care tests to guide prescription of antibiotics in people with acute respiratory infections in primary care. *Cochrane Database of Systematic Reviews* 2022(10). - Smart KM, Ferraro MC, Wand BM, O'Connell NE: Physiotherapy for pain and disability in adults with complex regional pain syndrome (CRPS) types I and II. *Cochrane Database of Systematic Reviews* 2022(5). - Sinamaw D, Getnet M, Abdulkadir M, Abebaw K, Ebrahim M, Diress M, Akalu Y, Ambelu A, Dagnew B: Patterns and associated factors of electrocardiographic abnormality among type 2 diabetic patients in Amhara National Regional State Referral Hospitals, Ethiopia: a multicenter institution-based cross-sectional study. *BMC Cardiovascular Disorders* 2022, 22(1):230. - Sinamaw D, Getnet M, Abdulkadir M, Abebaw K, Ebrahim M, Diress M, Akalu Y, Ambelu A, Dagnew B: Patterns and associated factors of electrocardiographic abnormality among type 2 diabetic patients in Amhara National Regional State Referral Hospitals, Ethiopia: a multicenter institution-based cross-sectional study. *BMC Cardiovascular Disorders* 2022, 22(1):230. - Sinamaw D, Getnet M, Abdulkadir M, Abebaw K, Ebrahim M, Diress M, Akalu Y, Ambelu A, Dagnew B: Patterns and associated factors of electrocardiographic abnormality among type 2 diabetic patients in Amhara National Regional State Referral Hospitals, Ethiopia: a multicenter institution-based cross-sectional study. *BMC cardiovascular disorders* 2022, 22(1):230-230. - Sinamaw D, Getnet M, Abdulkadir M, Abebaw K, Ebrahim M, Diress M, Akalu Y, Ambelu A, Dagnew B: Patterns and associated factors of electrocardiographic abnormality among type 2 diabetic patients in Amhara National Regional State Referral Hospitals, Ethiopia: a multicenter institution-based cross-sectional study. *BMC Cardiovasc Disord* 2022, 22(1):230. - Simpson TC, Clarkson JE, Worthington HV, MacDonald L, Weldon JC, Needleman I, Iheozor-Ejiofor Z, Wild SH, Qureshi A, Walker A *et al*: Treatment of periodontitis for glycaemic control in people with diabetes mellitus. *Cochrane Database of Systematic Reviews* 2022(4). - Silva JA, Mininel VA, Fernandes Agreli H, Peduzzi M, Harrison R, Xyrichis A: Collective leadership to improve professional practice, healthcare outcomes and staff well‐being. *Cochrane Database of Systematic Reviews* 2022(10). - Shantsila E, Kozieł-Siołkowska M, Lip GYH: Antiplatelet agents and anticoagulants for hypertension. *Cochrane Database of Systematic Reviews* 2022(7). - Serednicki WT, Wrzosek A, Woron J, Garlicki J, Dobrogowski J, Jakowicka-Wordliczek J, Wordliczek J, Zajaczkowska R: Topical clonidine for neuropathic pain in adults. *Cochrane Database of Systematic Reviews* 2022(5). - Sereda M, Xia J, Scutt P, Hilton MP, El Refaie A, Hoare DJ: Ginkgo biloba for tinnitus. *Cochrane Database of Systematic Reviews* 2022(11). - Schmidt-Hansen M, Bennett MI, Arnold S, Bromham N, Hilgart JS, Page AJ, Chi Y: Oxycodone for cancer‐related pain. *Cochrane Database of Systematic Reviews* 2022(6). - Sardesai VV, Kokane HT, Mukherjee S, Sangle SA: A study of electrocardiographic and 2D echocardiographic changes in type 2 diabetes mellitus patients without cardiovascular symptoms. *J Family Med Prim Care* 2022, 11(3):1036-1039. - Ryan RE, Connolly M, Bradford NK, Henderson S, Herbert A, Schonfeld L, Young J, Bothroyd JI, Henderson A: Interventions for interpersonal communication about end of life care between health practitioners and affected people. *Cochrane Database of Systematic Reviews* 2022(7). - Rutherford SJ, Glenny AM, Roberts G, Hooper L, Worthington HV: Antibiotic prophylaxis for preventing bacterial endocarditis following dental procedures. *Cochrane Database of Systematic Reviews* 2022(5). - Reynolds-Wright JJ, Woldetsadik MA, Morroni C, Cameron S: Pain management for medical abortion before 14 weeks’ gestation. *Cochrane Database of Systematic Reviews* 2022(5). - Renton WD, Jung J, Palestine AG: Tumor necrosis factor (TNF) inhibitors for juvenile idiopathic arthritis‐associated uveitis. *Cochrane Database of Systematic Reviews* 2022(10). - Radtke T, Smith S, Nevitt SJ, Hebestreit H, Kriemler S: Physical activity and exercise training in cystic fibrosis. *Cochrane Database of Systematic Reviews* 2022(8). - Pryce J, Taylor M, Fox T, Hine P: Pyronaridine‐artesunate for treating uncomplicated Plasmodium falciparum malaria. *Cochrane Database of Systematic Reviews* 2022(6). - Popp M, Reis S, Schießer S, Hausinger R, Stegemann M, Metzendorf MI, Kranke P, Meybohm P, Skoetz N, Weibel S: Ivermectin for preventing and treating COVID‐19. *Cochrane Database of Systematic Reviews* 2022(6). - Plontke SK, Meisner C, Agrawal S, Cayé-Thomasen P, Galbraith K, Mikulec AA, Parnes L, Premakumar Y, Reiber J, Schilder AGM *et al*: Intratympanic corticosteroids for sudden sensorineural hearing loss. *Cochrane Database of Systematic Reviews* 2022(7). - Pinto AN, Rocha A, Drager LF, Lorenzi-Filho G, Pachito DV: Non‐invasive positive pressure ventilation for central sleep apnoea in adults. *Cochrane Database of Systematic Reviews* 2022(10). - Pillay S, Steingart KR, Davies GR, Chaplin M, De Vos M, Schumacher SG, Warren R, Theron G: Xpert MTB/XDR for detection of pulmonary tuberculosis and resistance to isoniazid, fluoroquinolones, ethionamide, and amikacin. *Cochrane Database of Systematic Reviews* 2022(5). - Pilkington K, Wieland LS, Teng L, Jin XY, Storey D, Liu JP: Coriolus (Trametes) versicolor mushroom to reduce adverse effects from chemotherapy or radiotherapy in people with colorectal cancer. *Cochrane Database of Systematic Reviews* 2022(11). - Perrotta C, Chahla J, Badariotti G, Ramos J: Interventions for preventing venous thromboembolism in adults undergoing knee arthroscopy. *Cochrane Database of Systematic Reviews* 2022(8). - Orrapin S, Benyakorn T, Siribumrungwong B, Rerkasem K: Patch angioplasty versus primary closure for carotid endarterectomy. *Cochrane Database of Systematic Reviews* 2022(8). - Orgeta V, Leung P, del-Pino-Casado R, Qazi A, Orrell M, Spector AE, Methley AM: Psychological treatments for depression and anxiety in dementia and mild cognitive impairment. *Cochrane Database of Systematic Reviews* 2022(4). - Ochodo EA, Olwanda E, Deeks JJ, Mallett S: Point‐of‐care viral load tests to detect high HIV viral load in people living with HIV/AIDS attending health facilities. *Cochrane Database of Systematic Reviews* 2022(3). - O'Byrne L, Webster KE, MacKeith S, Philpott C, Hopkins C, Burton MJ: Interventions for the treatment of persistent post‐COVID‐19 olfactory dysfunction. *Cochrane Database of Systematic Reviews* 2022(9). - Oba Y, Anwer S, Maduke T, Patel T, Dias S: Effectiveness and tolerability of dual and triple combination inhaler therapies compared with each other and varying doses of inhaled corticosteroids in adolescents and adults with asthma: a systematic review and network meta‐analysis. *Cochrane Database of Systematic Reviews* 2022(12). - Nyirenda JLZ, Sofroniou M, Toews I, Mikolajewska A, Lehane C, Monsef I, Abu-taha A, Maun A, Stegemann M, Schmucker C: Fluvoxamine for the treatment of COVID‐19. *Cochrane Database of Systematic Reviews* 2022(9). - Nunan D, Cai T, Gardener AD, Ordóñez-Mena JM, Roberts NW, Thomas ET, Mahtani KR: Physical activity for treatment of irritable bowel syndrome. *Cochrane Database of Systematic Reviews* 2022(6). - Norman G, Wong JKF, Amin K, Dumville JC, Pramod S: Reconstructive surgery for treating pressure ulcers. *Cochrane Database of Systematic Reviews* 2022(10). - Nishioka N, Luo Y, Taniguchi T, Ohnishi T, Kimachi M, Ng RCK, Watanabe N: Carnitine supplements for people with chronic kidney disease requiring dialysis. *Cochrane Database of Systematic Reviews* 2022(12). - Nevitt SJ, Sudell M, Cividini S, Marson AG, Tudur Smith C: Antiepileptic drug monotherapy for epilepsy: a network meta‐analysis of individual participant data. *Cochrane Database of Systematic Reviews* 2022(4). - Natale P, Palmer SC, Saglimbene VM, Ruospo M, Razavian M, Craig JC, Jardine MJ, Webster AC, Strippoli GFM: Antiplatelet agents for chronic kidney disease. *Cochrane Database of Systematic Reviews* 2022(2). - Natale P, Palmer SC, Jaure A, Hodson EM, Ruospo M, Cooper TE, Hahn D, Saglimbene VM, Craig JC, Strippoli GFM: Hypoxia‐inducible factor stabilisers for the anaemia of chronic kidney disease. *Cochrane Database of Systematic Reviews* 2022(8). - Nantakool S, Reanpang T, Prasannarong M, Pongtam S, Rerkasem K: Upper limb exercise for arteriovenous fistula maturation in people requiring permanent haemodialysis access. *Cochrane Database of Systematic Reviews* 2022(10). - Myint KT, Sahoo S, Thein AW, Moe S, Ni H: Laser therapy for retinopathy in sickle cell disease. *Cochrane Database of Systematic Reviews* 2022(12). - Moazzami B, Mohammadpour Z, Zabala ZE, Farokhi E, Roohi A, Dolmatova E, Moazzami K: Local intramuscular transplantation of autologous bone marrow mononuclear cells for critical lower limb ischaemia. *Cochrane Database of Systematic Reviews* 2022(7). - Midya S, Ghosh D, Mahmalat MW: Fundoplication in laparoscopic Heller's cardiomyotomy for achalasia. *Cochrane Database of Systematic Reviews* 2022(12). - Mellon L, Doyle F, Hickey A, Ward KD, de Freitas DG, McCormick PA, O'Connell O, Conlon P: Interventions for increasing immunosuppressant medication adherence in solid organ transplant recipients. *Cochrane Database of Systematic Reviews* 2022(9). - McTavish D, Thornton J: Appetite stimulants for people with cystic fibrosis. *Cochrane Database of Systematic Reviews* 2022(9). - McAleenan A, Jones HE, Kernohan A, Robinson T, Schmidt L, Dawson S, Kelly C, Spencer Leal E, Faulkner CL, Palmer A *et al*: Diagnostic test accuracy and cost‐effectiveness of tests for codeletion of chromosomal arms 1p and 19q in people with glioma. *Cochrane Database of Systematic Reviews* 2022(3). - Marson BA, Ikram A, Craxford S, Lewis SR, Price KR, Ollivere BJ: Interventions for treating supracondylar elbow fractures in children. *Cochrane Database of Systematic Reviews* 2022(6). - Manoharan MP, Raja R, Jamil A, Csendes D, Gutlapalli SD, Prakash K, Swarnakari KM, Bai M, Desai DM, Desai A: Obesity and coronary artery disease: an updated systematic review 2022. *Cureus* 2022, 14(9). - Ma Y, Wang H, Jiang J, Han C, Lu C, Zeng S, Wang Y, Zheng Z, Peng Y, Ding X: Prevalence of and risk factors for diabetic retinopathy in residents with different types of abnormal glucose metabolism with or without hypertension: A suburban community-based cross-sectional study. *Front Endocrinol (Lausanne)* 2022, 13:966619. - López-Briz E, Ruiz Garcia V, Cabello JB, Bort-Martí S, Carbonell Sanchis R: Heparin versus 0.9% sodium chloride locking for prevention of occlusion in central venous catheters in adults. *Cochrane Database of Systematic Reviews* 2022(7). - Liu SH, Saldanha IJ, Abraham AG, Rittiphairoj T, Hauswirth S, Gregory D, Ifantides C, Li T: Topical corticosteroids for dry eye. *Cochrane Database of Systematic Reviews* 2022(10). - Liu SH, Hawkins BS, Ng SM, Ren M, Leslie L, Han G, Kuo IC: Topical pharmacologic interventions versus placebo for epidemic keratoconjunctivitis. *Cochrane Database of Systematic Reviews* 2022(3). - Liao L, Shi B, Ding Z, Chen L, Dong F, Li J, Zhong Y, Xu J: Echocardiographic study of myocardial work in patients with type 2 diabetes mellitus. *BMC Cardiovascular Disorders* 2022, 22(1):59. - Lewis SR, Macey R, Lewis J, Stokes J, Gill JR, Cook JA, Eardley WGP, Parker MJ, Griffin XL: Surgical interventions for treating extracapsular hip fractures in older adults: a network meta‐analysis. *Cochrane Database of Systematic Reviews* 2022(2). - Lewis SR, Macey R, Gill JR, Parker MJ, Griffin XL: Cephalomedullary nails versus extramedullary implants for extracapsular hip fractures in older adults. *Cochrane Database of Systematic Reviews* 2022(1). - Leszczynski R, da Silva CAP, Pinto AN, Kuczynski U, da Silva EMK: Laser therapy for treating hypertrophic and keloid scars. *Cochrane Database of Systematic Reviews* 2022(9). - Leaney AA, Lyttle JR, Segan J, Urquhart DM, Cicuttini FM, Chou L, Wluka AE: Antidepressants for hip and knee osteoarthritis. *Cochrane Database of Systematic Reviews* 2022(10). - Lax SJ, Harvey J, Axon E, Howells L, Santer M, Ridd MJ, Lawton S, Langan S, Roberts A, Ahmed A *et al*: Strategies for using topical corticosteroids in children and adults with eczema. *Cochrane Database of Systematic Reviews* 2022(3). - Kwan J, Hafdi M, Chiang LW, Myint PK, Wong LS, Quinn TJ: Antithrombotic therapy to prevent cognitive decline in people with small vessel disease on neuroimaging but without dementia. *Cochrane Database of Systematic Reviews* 2022(7). - Kumbargere Nagraj S, Eachempati P, Paisi M, Nasser M, Sivaramakrishnan G, Francis T, Verbeek JH: Preprocedural mouth rinses for preventing transmission of infectious diseases through aerosols in dental healthcare providers. *Cochrane Database of Systematic Reviews* 2022(8). - Kuehn R, Stoesser N, Eyre D, Darton TC, Basnyat B, Parry CM: Treatment of enteric fever (typhoid and paratyphoid fever) with cephalosporins. *Cochrane Database of Systematic Reviews* 2022(11). - Kreuzberger N, Hirsch C, Andreas M, Böhm L, Bröckelmann PJ, Di Cristanziano V, Golinski M, Hausinger R, Mellinghoff S, Lange B *et al*: Immunity after COVID‐19 vaccination in people with higher risk of compromised immune status: a scoping review. *Cochrane Database of Systematic Reviews* 2022(8). - Kramer A, Prinz C, Fichtner F, Fischer AL, Thieme V, Grundeis F, Spagl M, Seeber C, Piechotta V, Metzendorf MI *et al*: Janus kinase inhibitors for the treatment of COVID‐19. *Cochrane Database of Systematic Reviews* 2022(6). - Kotani Y, Kataoka Y, Izawa J, Fujioka S, Yoshida T, Kumasawa J, Kwong JSW: High versus low blood pressure targets for cardiac surgery while on cardiopulmonary bypass. *Cochrane Database of Systematic Reviews* 2022(11). - Kirkman MA, Day J, Gehring K, Zienius K, Grosshans D, Taphoorn M, Li J, Brown PD: Interventions for preventing and ameliorating cognitive deficits in adults treated with cranial irradiation. *Cochrane Database of Systematic Reviews* 2022(11). - Kew KM, Flemyng E, Quon BS, Leung C: Increased versus stable doses of inhaled corticosteroids for exacerbations of chronic asthma in adults and children. *Cochrane Database of Systematic Reviews* 2022(9). - Kaze AD, Yuyun MF, Erqou S, Fonarow GC, Echouffo‐Tcheugui JB: Cardiac autonomic neuropathy and risk of incident heart failure among adults with type 2 diabetes. *European journal of heart failure* 2022, 24(4):634-641. - Kang S, Hamed Azzam S, Minakaran N, Ezra DG: Rituximab for thyroid‐associated ophthalmopathy. *Cochrane Database of Systematic Reviews* 2022(6). - Kamo T, Wada Y, Okamura M, Sakai K, Momosaki R, Taito S: Repetitive peripheral magnetic stimulation for impairment and disability in people after stroke. *Cochrane Database of Systematic Reviews* 2022(9). - Kakkos S, Kirkilesis G, Caprini JA, Geroulakos G, Nicolaides A, Stansby G, Reddy DJ: Combined intermittent pneumatic leg compression and pharmacological prophylaxis for prevention of venous thromboembolism. *Cochrane Database of Systematic Reviews* 2022(1). - Jervøe-Storm PM, Eberhard J, Needleman I, Worthington HV, Jepsen S: Full‐mouth treatment modalities (within 24 hours) for periodontitis in adults. *Cochrane Database of Systematic Reviews* 2022(6). - James E, Ellis C, Brassington R, Sathasivam S, Young CA: Treatment for sialorrhea (excessive saliva) in people with motor neuron disease/amyotrophic lateral sclerosis. *Cochrane Database of Systematic Reviews* 2022(5). - Jacobsen A, Olabi B, Langley A, Beecker J, Mutter E, Shelley A, Worley B, Ramsay T, Saavedra A, Parker R *et al*: Systemic interventions for treatment of Stevens‐Johnson syndrome (SJS), toxic epidermal necrolysis (TEN), and SJS/TEN overlap syndrome. *Cochrane Database of Systematic Reviews* 2022(3). - Hüttner FJ, Klotz R, Ulrich A, Büchler MW, Probst P, Diener MK: Antecolic versus retrocolic reconstruction after partial pancreaticoduodenectomy. *Cochrane Database of Systematic Reviews* 2022(1). - Hohmann F, Wedekind L, Grundeis F, Dickel S, Frank J, Golinski M, Griesel M, Grimm C, Herchenhahn C, Kramer A *et al*: Early spontaneous breathing for acute respiratory distress syndrome in individuals with COVID‐19. *Cochrane Database of Systematic Reviews* 2022(6). - Hodson EM, Sinha A, Cooper TE: Interventions for focal segmental glomerulosclerosis in adults. *Cochrane Database of Systematic Reviews* 2022(2). - Hendrix CLF, van den Heuvel FMA, Rodwell L, Timmermans J, Nijveldt R, Janssen MCH, Saris CGJ: Screening and prevalence of cardiac abnormalities on electro- and echocardiography in a large cohort of patients with mitochondrial disease. *Mol Genet Metab* 2022, 136(3):219-225. - Heinz KD, Walsh A, Southern KW, Johnstone Z, Regan KH: Exercise versus airway clearance techniques for people with cystic fibrosis. *Cochrane Database of Systematic Reviews* 2022(6). - Hayes K, Fernando MC, Jordan V: Prothrombin complex concentrate in cardiac surgery for the treatment of coagulopathic bleeding. *Cochrane Database of Systematic Reviews* 2022(11). - Harvey JJ, Huang S, Uberoi R: Catheter‐directed therapies for the treatment of high risk (massive) and intermediate risk (submassive) acute pulmonary embolism. *Cochrane Database of Systematic Reviews* 2022(8). - Hartley P, Keating JL, Jeffs KJ, Raymond MJM, Smith TO: Exercise for acutely hospitalised older medical patients. *Cochrane Database of Systematic Reviews* 2022(11). - Hansel J, Rogers AM, Lewis SR, Cook TM, Smith AF: Videolaryngoscopy versus direct laryngoscopy for adults undergoing tracheal intubation. *Cochrane Database of Systematic Reviews* 2022(4). - Handoll HHG, Elliott J, Thillemann TM, Aluko P, Brorson S: Interventions for treating proximal humeral fractures in adults. *Cochrane Database of Systematic Reviews* 2022(6). - Griesel M, Wagner C, Mikolajewska A, Stegemann M, Fichtner F, Metzendorf MI, Nair A, Daniel J, Fischer AL, Skoetz N: Inhaled corticosteroids for the treatment of COVID‐19. *Cochrane Database of Systematic Reviews* 2022(3). - Gregers MC, Schou M, Jensen MT, Jensen J, Petrie MC, Vilsbøll T, Goetze JP, Rossing P, Jørgensen PG: Diagnostic and prognostic value of the electrocardiogram in stable outpatients with type 2 diabetes. *Scandinavian Cardiovascular Journal* 2022, 56(1):256-263. - Granger C, Cavalheri V: Preoperative exercise training for people with non‐small cell lung cancer. *Cochrane Database of Systematic Reviews* 2022(9). - Graña C, Ghosn L, Evrenoglou T, Jarde A, Minozzi S, Bergman H, Buckley BS, Probyn K, Villanueva G, Henschke N *et al*: Efficacy and safety of COVID‐19 vaccines. *Cochrane Database of Systematic Reviews* 2022(12). - Gottlieb M, Carlson JN, Peksa GD: Prophylactic antiemetics for adults receiving intravenous opioids in the acute care setting. *Cochrane Database of Systematic Reviews* 2022(5). - Gordon M, Sinopoulou V, Grafton-Clarke C, Akobeng AK: Antibiotics for the induction and maintenance of remission in ulcerative colitis. *Cochrane Database of Systematic Reviews* 2022(5). - Gordon M, Sinopoulou V, Akobeng AK, Pana M, Gasiea R, Moran GW: Tacrolimus (FK506) for induction of remission in corticosteroid‐refractory ulcerative colitis. *Cochrane Database of Systematic Reviews* 2022(4). - Gonçalves-Bradley DC, Lannin NA, Clemson L, Cameron ID, Shepperd S: Discharge planning from hospital. *Cochrane Database of Systematic Reviews* 2022(2). - Gibson E, Koh CL, Eames S, Bennett S, Scott AM, Hoffmann TC: Occupational therapy for cognitive impairment in stroke patients. *Cochrane Database of Systematic Reviews* 2022(3). - Ghossein MA, Driessen RGH, van Rosmalen F, Sels JEM, Delnoij T, Geyik Z, Mingels AMA, van Stipdonk AMW, Prinzen FW, Ghossein-Doha C *et al*: Serial Assessment of Myocardial Injury Markers in Mechanically Ventilated Patients With SARS-CoV-2 (from the Prospective MaastrICCht Cohort). *Am J Cardiol* 2022, 170:118-127. - Georgiou EX, Melo P, Cheong YC, Granne IE: Follicular flushing during oocyte retrieval in assisted reproductive techniques. *Cochrane Database of Systematic Reviews* 2022(11). - French HP, Abbott JH, Galvin R: Adjunctive therapies in addition to land‐based exercise therapy for osteoarthritis of the hip or knee. *Cochrane Database of Systematic Reviews* 2022(10). - Fraser A, Poole P: Immunostimulants versus placebo for preventing exacerbations in adults with chronic bronchitis or chronic obstructive pulmonary disease. *Cochrane Database of Systematic Reviews* 2022(11). - Fox T, Geppert J, Dinnes J, Scandrett K, Bigio J, Sulis G, Hettiarachchi D, Mathangasinghe Y, Weeratunga P, Wickramasinghe D *et al*: Antibody tests for identification of current and past infection with SARS‐CoV‐2. *Cochrane Database of Systematic Reviews* 2022(11). - Flumignan CDQ, Nakano LCU, Baptista-Silva JCC, Flumignan RLG: Antiplatelet agents for the treatment of deep venous thrombosis. *Cochrane Database of Systematic Reviews* 2022(7). - Filippini G, Minozzi S, Borrelli F, Cinquini M, Dwan K: Cannabis and cannabinoids for symptomatic treatment for people with multiple sclerosis. *Cochrane Database of Systematic Reviews* 2022(5). - Fernandez R, Green HL, Griffiths R, Atkinson RA, Ellwood LJ: Water for wound cleansing. *Cochrane Database of Systematic Reviews* 2022(9). - Fayad AI, Buamscha DG, Ciapponi A: Timing of kidney replacement therapy initiation for acute kidney injury. *Cochrane Database of Systematic Reviews* 2022(11). - Farne HA, Wilson A, Milan S, Banchoff E, Yang F, Powell CVE: Anti‐IL‐5 therapies for asthma. *Cochrane Database of Systematic Reviews* 2022(7). - Faltinsen E, Todorovac A, Staxen Bruun L, Hróbjartsson A, Gluud C, Kongerslev MT, Simonsen E, Storebø OJ: Control interventions in randomised trials among people with mental health disorders. *Cochrane Database of Systematic Reviews* 2022(4). - Fairhall NJ, Dyer SM, Mak JCS, Diong J, Kwok WS, Sherrington C: Interventions for improving mobility after hip fracture surgery in adults. *Cochrane Database of Systematic Reviews* 2022(9). - Engel N, Ochodo EA, Karanja PW, Schmidt BM, Janssen R, Steingart KR, Oliver S: Rapid molecular tests for tuberculosis and tuberculosis drug resistance: a qualitative evidence synthesis of recipient and provider views. *Cochrane Database of Systematic Reviews* 2022(4). - Emami S, Kitayama K, Coleman AL: Adjunctive steroid therapy versus antibiotics alone for acute endophthalmitis after intraocular procedure. *Cochrane Database of Systematic Reviews* 2022(6). - El-Nakeep S, Shawky A, Abbas SF, Abdel Latif O: Stem cell transplantation for induction of remission in medically refractory Crohn’s disease. *Cochrane Database of Systematic Reviews* 2022(5). - Dowling N, Merkouris S, Lubman D, Thomas S, Bowden-Jones H, Cowlishaw S: Pharmacological interventions for the treatment of disordered and problem gambling. *Cochrane Database of Systematic Reviews* 2022(9). - Dower A, Mulcahy M, Maharaj M, Chen H, Lim CE, Li Y, Sheridan M: Surgical decompression for malignant cerebral oedema after ischaemic stroke. *Cochrane Database of Systematic Reviews* 2022(11). - Dinnes J, Sharma P, Berhane S, van Wyk SS, Nyaaba N, Domen J, Taylor M, Cunningham J, Davenport C, Dittrich S *et al*: Rapid, point‐of‐care antigen tests for diagnosis of SARS‐CoV‐2 infection. *Cochrane Database of Systematic Reviews* 2022(7). - Dickman MM, Spekreijse LS, Winkens B, Schouten J, Simons RWP, Dirksen CD, Nuijts R: Immediate sequential bilateral surgery versus delayed sequential bilateral surgery for cataracts. *Cochrane Database of Systematic Reviews* 2022(4). - Dichman ML, Rosenstock SJ, Shabanzadeh DM: Antibiotics for uncomplicated diverticulitis. *Cochrane Database of Systematic Reviews* 2022(6). - de Baat EC, Mulder RL, Armenian S, Feijen EAM, Grotenhuis H, Hudson MM, Mavinkurve-Groothuis AM, Kremer LCM, van Dalen EC: Dexrazoxane for preventing or reducing cardiotoxicity in adults and children with cancer receiving anthracyclines. *Cochrane Database of Systematic Reviews* 2022(9). - Davidson M, Menon S, Chaimani A, Evrenoglou T, Ghosn L, Graña C, Henschke N, Cogo E, Villanueva G, Ferrand G *et al*: Interleukin‐1 blocking agents for treating COVID‐19. *Cochrane Database of Systematic Reviews* 2022(1). - Davenport C, Rai N, Sharma P, Deeks JJ, Berhane S, Mallett S, Saha P, Champaneria R, Bayliss SE, Snell KIE *et al*: Menopausal status, ultrasound and biomarker tests in combination for the diagnosis of ovarian cancer in symptomatic women. *Cochrane Database of Systematic Reviews* 2022(7). - Daly BJM, Sharif MO, Jones K, Worthington HV, Beattie A: Local interventions for the management of alveolar osteitis (dry socket). *Cochrane Database of Systematic Reviews* 2022(9). - Cross AJ, Liang J, Thomas D, Zairina E, Abramson MJ, George J: Educational interventions for health professionals managing chronic obstructive pulmonary disease in primary care. *Cochrane Database of Systematic Reviews* 2022(5). - Creavin ST, Noel-Storr AH, Langdon RJ, Richard E, Creavin AL, Cullum S, Purdy S, Ben-Shlomo Y: Clinical judgement by primary care physicians for the diagnosis of all‐cause dementia or cognitive impairment in symptomatic people. *Cochrane Database of Systematic Reviews* 2022(6). - Cormick G, Ciapponi A, Cafferata ML, Cormick MS, Belizán JM: Calcium supplementation for prevention of primary hypertension. *Cochrane Database of Systematic Reviews* 2022(1). - Cooper TE, Teng C, Howell M, Teixeira-Pinto A, Jaure A, Wong G: D‐mannose for preventing and treating urinary tract infections. *Cochrane Database of Systematic Reviews* 2022(8). - Cooper TE, Scholes-Robertson N, Craig JC, Hawley CM, Howell M, Johnson DW, Teixeira-Pinto A, Jaure A, Wong G: Synbiotics, prebiotics and probiotics for solid organ transplant recipients. *Cochrane Database of Systematic Reviews* 2022(9). - Cooke G, Watson C, Deckx L, Pirotta M, Smith J, van Driel ML: Treatment for recurrent vulvovaginal candidiasis (thrush). *Cochrane Database of Systematic Reviews* 2022(1). - Clare G, Bunce C, Tuft S: Amniotic membrane transplantation for acute ocular burns. *Cochrane Database of Systematic Reviews* 2022(9). - Claireaux HA, Searle HKC, Parsons NR, Griffin XL: Interventions for treating fractures of the distal femur in adults. *Cochrane Database of Systematic Reviews* 2022(10). - Chuatrakoon B, Nantakool S, Rerkasem A, Orrapin S, Howard DPJ, Rerkasem K: Routine or selective carotid artery shunting for carotid endarterectomy (and different methods of monitoring in selective shunting). *Cochrane Database of Systematic Reviews* 2022(6). - Chou R, Pinto RZ, Fu R, Lowe RA, Henschke N, McAuley JH, Dana T: Systemic corticosteroids for radicular and non‐radicular low back pain. *Cochrane Database of Systematic Reviews* 2022(10). - Chetty RR, Pillay S: Electrocardiogram (ECG) Diagnosis of Left Ventricular Hypertrophy and its Associations in Patients Living with Diabetes. *Indian Journal of Endocrinology and Metabolism* 2022, 26(5):465-470. - Cheng Y, Wang K, Gong J, Liu Z, Gong J, Zeng Z, Wang X: Negative pressure wound therapy for managing the open abdomen in non‐trauma patients. *Cochrane Database of Systematic Reviews* 2022(5). - Chen X, Jiang J, Wang R, Fu H, Lu J, Yang M: Chest physiotherapy for pneumonia in adults. *Cochrane Database of Systematic Reviews* 2022(9). - Chan A, De Simoni A, Wileman V, Holliday L, Newby CJ, Chisari C, Ali S, Zhu N, Padakanti P, Pinprachanan V *et al*: Digital interventions to improve adherence to maintenance medication in asthma. *Cochrane Database of Systematic Reviews* 2022(6). - Cassola N, Baptista-Silva JCC, Nakano LCU, Flumignan CDQ, Sesso R, Vasconcelos V, Carvas Junior N, Flumignan RLG: Duplex ultrasound for diagnosing symptomatic carotid stenosis in the extracranial segments. *Cochrane Database of Systematic Reviews* 2022(7). - Candy B, Jones L, Vickerstaff V, Larkin PJ, Stone P: Mu‐opioid antagonists for opioid‐induced bowel dysfunction in people with cancer and people receiving palliative care. *Cochrane Database of Systematic Reviews* 2022(9). - Campbell ZC, Dawson JK, Kirkendall SM, McCaffery KJ, Jansen J, Campbell KL, Lee VWS, Webster AC: Interventions for improving health literacy in people with chronic kidney disease. *Cochrane Database of Systematic Reviews* 2022(12). - Byrne A, Torrens-Burton A, Sivell S, Moraes F, Bulbeck H, Bernstein M, Nelson A, Fielding H: Early palliative interventions for improving outcomes in people with a primary malignant brain tumour and their carers. *Cochrane Database of Systematic Reviews* 2022(1). - Buder K, Zirngibl M, Bapistella S, Meerpohl JJ, Strahm B, Bassler D, Weitz M: Extracorporeal photopheresis versus standard treatment for acute graft‐versus‐host disease after haematopoietic stem cell transplantation in children and adolescents. *Cochrane Database of Systematic Reviews* 2022(9). - Buder K, Zirngibl M, Bapistella S, Meerpohl JJ, Strahm B, Bassler D, Weitz M: Extracorporeal photopheresis versus alternative treatment for chronic graft‐versus‐host disease after haematopoietic stem cell transplantation in children and adolescents. *Cochrane Database of Systematic Reviews* 2022(6). - Bruschettini M, Hassan KO, Romantsik O, Banzi R, Calevo MG, Moresco L: Interventions for the management of transient tachypnoea of the newborn ‐ an overview of systematic reviews. *Cochrane Database of Systematic Reviews* 2022(2). - Bresnahan R, Panebianco M, Marson AG: Brivaracetam add‐on therapy for drug‐resistant epilepsy. *Cochrane Database of Systematic Reviews* 2022(3). - Borges L, Fernandes A, Oliveira dos Passos J, Rego IO, Campos TF: Action observation for upper limb rehabilitation after stroke. *Cochrane Database of Systematic Reviews* 2022(8). - Boomsma CM, Kamath MS, Keay SD, Macklon NS: Peri‐implantation glucocorticoid administration for assisted reproductive technology cycles. *Cochrane Database of Systematic Reviews* 2022(6). - Bofill Rodriguez M, Dias S, Jordan V, Lethaby A, Lensen SF, Wise MR, Wilkinson J, Brown J, Farquhar C: Interventions for heavy menstrual bleeding; overview of Cochrane reviews and network meta‐analysis. *Cochrane Database of Systematic Reviews* 2022(5). - Boesen K, Paludan-Müller AS, Gøtzsche PC, Jørgensen KJ: Extended‐release methylphenidate for attention deficit hyperactivity disorder (ADHD) in adults. *Cochrane Database of Systematic Reviews* 2022(2). - Bhandari AP, Nnate DA, Vasanthan L, Konstantinidis M, Thompson J: Positioning for acute respiratory distress in hospitalised infants and children. *Cochrane Database of Systematic Reviews* 2022(6). - Beth-Tasdogan NH, Mayer B, Hussein H, Zolk O, Peter JU: Interventions for managing medication‐related osteonecrosis of the jaw. *Cochrane Database of Systematic Reviews* 2022(7). - Bernier-Jean A, Beruni NA, Bondonno NP, Williams G, Teixeira-Pinto A, Craig JC, Wong G: Exercise training for adults undergoing maintenance dialysis. *Cochrane Database of Systematic Reviews* 2022(1). - Azukaitis K, Palmer SC, Strippoli GFM, Hodson EM: Interventions for minimal change disease in adults with nephrotic syndrome. *Cochrane Database of Systematic Reviews* 2022(3). - Aoki Y, Yaju Y, Utsumi T, Sanyaolu L, Storm M, Takaesu Y, Watanabe K, Watanabe N, Duncan E, Edwards AGK: Shared decision‐making interventions for people with mental health conditions. *Cochrane Database of Systematic Reviews* 2022(11). - Antonio AA, Santos RN, Abariga SA: Tocilizumab for giant cell arteritis. *Cochrane Database of Systematic Reviews* 2022(5). - Andreas M, Iannizzi C, Bohndorf E, Monsef I, Piechotta V, Meerpohl JJ, Skoetz N: Interventions to increase COVID‐19 vaccine uptake: a scoping review. *Cochrane Database of Systematic Reviews* 2022(8). - Amaral FCF, Baptista-Silva JCC, Nakano LCU, Flumignan RLG: Pharmacological interventions for preventing venous thromboembolism in people undergoing bariatric surgery. *Cochrane Database of Systematic Reviews* 2022(11). - Alvarez Campano CG, Macleod MJ, Aucott L, Thies F: Marine‐derived n‐3 fatty acids therapy for stroke. *Cochrane Database of Systematic Reviews* 2022(6). - Almeida BM, Moreno DH, Vasconcelos V, Cacione DG: Interventions for treating catheter‐related bloodstream infections in people receiving maintenance haemodialysis. *Cochrane Database of Systematic Reviews* 2022(4). - Allen EN, Wiyeh A, McCaul M: Adding rapid diagnostic tests to community‐based programmes for treating malaria. *Cochrane Database of Systematic Reviews* 2022(9). - Adams A, Scheckel B, Habsaoui A, Haque M, Kuhr K, Monsef I, Bohlius J, Skoetz N: Intravenous iron versus oral iron versus no iron with or without erythropoiesis‐ stimulating agents (ESA) for cancer patients with anaemia: a systematic review and network meta‐analysis. *Cochrane Database of Systematic Reviews* 2022(6). - Abraham J, Hirt J, Richter C, Köpke S, Meyer G, Möhler R: Interventions for preventing and reducing the use of physical restraints of older people in general hospital settings. *Cochrane Database of Systematic Reviews* 2022(8). - Aboursheid T, Albaroudi O, Alahdab F: Inhaled nitric oxide for treating pain crises in people with sickle cell disease. *Cochrane Database of Systematic Reviews* 2022(7). - Zubkiewicz-Kucharska A, Noczyńska A, Sobieszczańska M, Poręba M, Chrzanowska J, Poręba R, Seifert M, Janocha A, Laszki-Szcząchor K: Disturbances in the intraventricular conduction system in teenagers with type 1 diabetes. A pilot study. *J Diabetes Complications* 2021, 35(11):108043. - Zifodya JS, Kreniske JS, Schiller I, Kohli M, Dendukuri N, Schumacher SG, Ochodo EA, Haraka F, Zwerling AA, Pai M *et al*: Xpert Ultra versus Xpert MTB/RIF for pulmonary tuberculosis and rifampicin resistance in adults with presumptive pulmonary tuberculosis. *Cochrane Database of Systematic Reviews* 2021(2). - Zegeye B, Anyiam FE, Ahinkorah BO, Ameyaw EK, Budu E, Seidu A-A, Yaya S: Prevalence of anemia and its associated factors among married women in 19 sub-Saharan African countries. *Archives of Public Health* 2021, 79:1-12. - Zayas-Arrabal J, Alquiza A, Tuncay E, Turan B, Gallego M, Casis O: Molecular and Electrophysiological Role of Diabetes-Associated Circulating Inflammatory Factors in Cardiac Arrhythmia Remodeling in a Metabolic-Induced Model of Type 2 Diabetic Rat. *International journal of molecular sciences* 2021, 22(13):6827. - Zahid M: PREVALENCE OF SMARTPHONE ADDICTION AMONG STUDENTS OF COLLEGES OF REHABILITATION SCIENCES. *Pakistan Journal of Rehabilitation* 2021, 10(2). - Zahid M: PREVALENCE OF SMARTPHONE ADDICTION AMONG STUDENTS OF COLLEGES OF REHABILITATION SCIENCES. *Pakistan Journal of Rehabilitation* 2021, 10(2). - Zadro J, Rischin A, Johnston RV, Buchbinder R: Image‐guided glucocorticoid injection versus injection without image guidance for shoulder pain. *Cochrane Database of Systematic Reviews* 2021(8). - Yusuf AM, Bizrah M, Bunce C, Bainbridge JW: Surgery for idiopathic epiretinal membrane. *Cochrane Database of Systematic Reviews* 2021(3). - Yonemoto N, Nagai S, Mori R: Schedules for home visits in the early postpartum period. *Cochrane Database of Systematic Reviews* 2021(7). - Yasuda H, Kondo N, Yamamoto R, Asami S, Abe T, Tsujimoto H, Tsujimoto Y, Kataoka Y: Monitoring of gastric residual volume during enteral nutrition. *Cochrane Database of Systematic Reviews* 2021(9). - Xyrichis A, Iliopoulou K, Mackintosh NJ, Bench S, Terblanche M, Philippou J, Sandall J: Healthcare stakeholders’ perceptions and experiences of factors affecting the implementation of critical care telemedicine (CCT): qualitative evidence synthesis. *Cochrane Database of Systematic Reviews* 2021(2). - Worthington HV, Khangura S, Seal K, Mierzwinski-Urban M, Veitz-Keenan A, Sahrmann P, Schmidlin PR, Davis D, Iheozor-Ejiofor Z, Rasines Alcaraz MG: Direct composite resin fillings versus amalgam fillings for permanent posterior teeth. *Cochrane Database of Systematic Reviews* 2021(8). - Witt KG, Hetrick SE, Rajaram G, Hazell P, Taylor Salisbury TL, Townsend E, Hawton K: Interventions for self‐harm in children and adolescents. *Cochrane Database of Systematic Reviews* 2021(3). - Witt KG, Hetrick SE, Rajaram G, Hazell P, Taylor Salisbury TL, Townsend E, Hawton K: Pharmacological interventions for self‐harm in adults. *Cochrane Database of Systematic Reviews* 2021(1). - Whing J, Nandhra S, Nesbitt C, Stansby G: Interventions for great saphenous vein incompetence. *Cochrane Database of Systematic Reviews* 2021(8). - Wang X, Ouyang M, Yang J, Song L, Yang M, Anderson CS: Anticoagulants for acute ischaemic stroke. *Cochrane Database of Systematic Reviews* 2021(10). - Walton D, Castell H, Collie C, Wood GK, Sharma M, Singh T, Michael BD: Antiepileptic drugs for seizure control in people with neurocysticercosis. *Cochrane Database of Systematic Reviews* 2021(11). - Walter MA, Nesti C, Spanjol M, Kollár A, Bütikofer L, Gloy VL, Dumont RA, Seiler CA, Christ ER, Radojewski P *et al*: Treatment for gastrointestinal and pancreatic neuroendocrine tumours: a network meta‐analysis. *Cochrane Database of Systematic Reviews* 2021(11). - Walsh T, Macey R, Riley P, Glenny AM, Schwendicke F, Worthington HV, Clarkson JE, Ricketts D, Su TL, Sengupta A: Imaging modalities to inform the detection and diagnosis of early caries. *Cochrane Database of Systematic Reviews* 2021(3). - Walsh T, Macey R, Kerr AR, Lingen MW, Ogden GR, Warnakulasuriya S: Diagnostic tests for oral cancer and potentially malignant disorders in patients presenting with clinically evident lesions. *Cochrane Database of Systematic Reviews* 2021(7). - von Groote TC, Williams G, Au EH, Chen Y, Mathew AT, Hodson EM, Tunnicliffe DJ: Immunosuppressive treatment for primary membranous nephropathy in adults with nephrotic syndrome. *Cochrane Database of Systematic Reviews* 2021(11). - Vernooij LM, van Klei WA, Moons KG, Takada T, van Waes J, Damen J: The comparative and added prognostic value of biomarkers to the Revised Cardiac Risk Index for preoperative prediction of major adverse cardiac events and all‐cause mortality in patients who undergo noncardiac surgery. *Cochrane Database of Systematic Reviews* 2021(12). - van Hoesel MH, Chen YL, Zheng A, Wan Q, Mourad SM: Selective oestrogen receptor modulators (SERMs) for endometriosis. *Cochrane Database of Systematic Reviews* 2021(5). - van Gruting IA, Stankiewicz A, Thakar R, Santoro GA, IntHout J, Sultan AH: Imaging modalities for the detection of posterior pelvic floor disorders in women with obstructed defaecation syndrome. *Cochrane Database of Systematic Reviews* 2021(9). - van Ginneken N, Chin WY, Lim YC, Ussif A, Singh R, Shahmalak U, Purgato M, Rojas-García A, Uphoff E, McMullen S *et al*: Primary‐level worker interventions for the care of people living with mental disorders and distress in low‐ and middle‐income countries. *Cochrane Database of Systematic Reviews* 2021(8). - Vaarwerk B, Breunis WB, Haveman LM, de Keizer B, Jehanno N, Borgwardt L, van Rijn RR, van den Berg H, Cohen JF, van Dalen EC *et al*: Fluorine‐18‐fluorodeoxyglucose (FDG) positron emission tomography (PET) computed tomography (CT) for the detection of bone, lung, and lymph node metastases in rhabdomyosarcoma. *Cochrane Database of Systematic Reviews* 2021(11). - Undela K, Goldsmith L, Kew KM, Ferrara G: Macrolides versus placebo for chronic asthma. *Cochrane Database of Systematic Reviews* 2021(11). - Ugolini A, Agostino P, Silvestrini-Biavati A, Harrison JE, Batista K: Orthodontic treatment for posterior crossbites. *Cochrane Database of Systematic Reviews* 2021(12). - Tully PJ, Ang SY, Lee EJL, Bendig E, Bauereiß N, Bengel J, Baumeister H: Psychological and pharmacological interventions for depression in patients with coronary artery disease. *Cochrane Database of Systematic Reviews* 2021(12). - Theventhiran AB, Kim G, Yao W: Fornix‐based versus limbal‐based conjunctival trabeculectomy flaps for glaucoma. *Cochrane Database of Systematic Reviews* 2021(8). - Teshale AB, Tessema ZT, Alem AZ, Yeshaw Y, Liyew AM, Alamneh TS, Tesema GA, Worku MG: Knowledge about mother to child transmission of HIV/AIDS, its prevention and associated factors among reproductive-age women in sub-Saharan Africa: Evidence from 33 countries recent Demographic and Health Surveys. *PloS one* 2021, 16(6):e0253164. - Taylor GMJ, Lindson N, Farley A, Leinberger-Jabari A, Sawyer K, te Water Naudé R, Theodoulou A, King N, Burke C, Aveyard P: Smoking cessation for improving mental health. *Cochrane Database of Systematic Reviews* 2021(3). - Taylor AM, Chan DH, Tio M, Patil SM, Traina TA, Robson ME, Khasraw M: PARP (Poly ADP‐Ribose Polymerase) inhibitors for locally advanced or metastatic breast cancer. *Cochrane Database of Systematic Reviews* 2021(4). - Surianarayanan V, Hoather TJ, Tingle SJ, Thompson ER, Hanley J, Wilson CH: Interventions for preventing thrombosis in solid organ transplant recipients. *Cochrane Database of Systematic Reviews* 2021(3). - Subramaniam P, Ho JJ, Davis PG: Prophylactic or very early initiation of continuous positive airway pressure (CPAP) for preterm infants. *Cochrane Database of Systematic Reviews* 2021(10). - Stroehlein JK, Wallqvist J, Iannizzi C, Mikolajewska A, Metzendorf MI, Benstoem C, Meybohm P, Becker M, Skoetz N, Stegemann M *et al*: Vitamin D supplementation for the treatment of COVID‐19: a living systematic review. *Cochrane Database of Systematic Reviews* 2021(5). - Storman D, Swierz MJ, Riemsma RP, Wolff R, Mitus JW, Pedziwiatr M, Kleijnen J, Bala MM: Electrocoagulation for liver metastases. *Cochrane Database of Systematic Reviews* 2021(1). - Staerkle RF, Lunger F, Fink L, Sasse T, Lacher M, von Elm E, Marwan AI, Holland-Cunz S, Vuille-dit-Bille R: Open versus laparoscopic pyloromyotomy for pyloric stenosis. *Cochrane Database of Systematic Reviews* 2021(3). - Spinks A, Glasziou PP, Del Mar CB: Antibiotics for treatment of sore throat in children and adults. *Cochrane Database of Systematic Reviews* 2021(12). - Smith V, Kenny LC, Sandall J, Devane D, Noonan M: Physiological track‐and‐trigger/early warning systems for use in maternity care. *Cochrane Database of Systematic Reviews* 2021(9). - Smith SM, Wallace E, O'Dowd T, Fortin M: Interventions for improving outcomes in patients with multimorbidity in primary care and community settings. *Cochrane Database of Systematic Reviews* 2021(1). - Sinopoulou V, Gordon M, Dovey TM, Akobeng AK: Interventions for the management of abdominal pain in ulcerative colitis. *Cochrane Database of Systematic Reviews* 2021(7). - Sinopoulou V, Gordon M, Akobeng AK, Gasparetto M, Sammaan M, Vasiliou J, Dovey TM: Interventions for the management of abdominal pain in Crohn's disease and inflammatory bowel disease. *Cochrane Database of Systematic Reviews* 2021(11). - Singh B, Ryan H, Kredo T, Chaplin M, Fletcher T: Chloroquine or hydroxychloroquine for prevention and treatment of COVID‐19. *Cochrane Database of Systematic Reviews* 2021(2). - Shi C, Dumville JC, Cullum N, Rhodes S, McInnes E, Goh EL, Norman G: Beds, overlays and mattresses for preventing and treating pressure ulcers: an overview of Cochrane Reviews and network meta‐analysis. *Cochrane Database of Systematic Reviews* 2021(8). - Shi C, Dumville JC, Cullum N, Rhodes S, McInnes E: Foam surfaces for preventing pressure ulcers. *Cochrane Database of Systematic Reviews* 2021(5). - Shi C, Dumville JC, Cullum N, Rhodes S, McInnes E: Alternative reactive support surfaces (non‐foam and non‐air‐filled) for preventing pressure ulcers. *Cochrane Database of Systematic Reviews* 2021(5). - Shi C, Dumville JC, Cullum N, Rhodes S, Leung V, McInnes E: Reactive air surfaces for preventing pressure ulcers. *Cochrane Database of Systematic Reviews* 2021(5). - Shi C, Dumville JC, Cullum N, Rhodes S, Jammali-Blasi A, Ramsden V, McInnes E: Beds, overlays and mattresses for treating pressure ulcers. *Cochrane Database of Systematic Reviews* 2021(5). - Shi C, Dumville JC, Cullum N, Rhodes S, Jammali-Blasi A, McInnes E: Alternating pressure (active) air surfaces for preventing pressure ulcers. *Cochrane Database of Systematic Reviews* 2021(5). - Shi C, Dumville JC, Cullum N, Connaughton E, Norman G: Compression bandages or stockings versus no compression for treating venous leg ulcers. *Cochrane Database of Systematic Reviews* 2021(7). - Shepperd S, Gonçalves-Bradley DC, Straus SE, Wee B: Hospital at home: home‐based end‐of‐life care. *Cochrane Database of Systematic Reviews* 2021(3). - Shapiro AE, Ross JM, Yao M, Schiller I, Kohli M, Dendukuri N, Steingart KR, Horne DJ: Xpert MTB/RIF and Xpert Ultra assays for screening for pulmonary tuberculosis and rifampicin resistance in adults, irrespective of signs or symptoms. *Cochrane Database of Systematic Reviews* 2021(3). - Sethi NJ, Safi S, Korang SK, Hróbjartsson A, Skoog M, Gluud C, Jakobsen JC: Antibiotics for secondary prevention of coronary heart disease. *Cochrane Database of Systematic Reviews* 2021(2). - Sekhar P, Tee QX, Ashraf G, Trinh D, Shachar J, Jiang A, Hewitt J, Green S, Turner T: Mindfulness‐based psychological interventions for improving mental well‐being in medical students and junior doctors. *Cochrane Database of Systematic Reviews* 2021(12). - Schwendicke F, Walsh T, Lamont T, Al-yaseen W, Bjørndal L, Clarkson JE, Fontana M, Gomez Rossi J, Göstemeyer G, Levey C *et al*: Interventions for treating cavitated or dentine carious lesions. *Cochrane Database of Systematic Reviews* 2021(7). - Sayum Filho J, Lenza M, Tamaoki MJS, Matsunaga FT, Belloti JC: Interventions for treating fractures of the patella in adults. *Cochrane Database of Systematic Reviews* 2021(2). - Saunders DH, Mead GE, Fitzsimons C, Kelly P, van Wijck F, Verschuren O, Backx K, English C: Interventions for reducing sedentary behaviour in people with stroke. *Cochrane Database of Systematic Reviews* 2021(6). - Sathianathen NJ, Hwang EC, Mian R, Bodie JA, Soubra A, Lyon JA, Sultan S, Dahm P: Selective serotonin re‐uptake inhibitors for premature ejaculation in adult men. *Cochrane Database of Systematic Reviews* 2021(3). - Sasongko TH, Nagalla S: Angiotensin‐converting enzyme (ACE) inhibitors for proteinuria and microalbuminuria in people with sickle cell disease. *Cochrane Database of Systematic Reviews* 2021(12). - Santa Cruz R, Villarejo F, Irrazabal C, Ciapponi A: High versus low positive end‐expiratory pressure (PEEP) levels for mechanically ventilated adult patients with acute lung injury and acute respiratory distress syndrome. *Cochrane Database of Systematic Reviews* 2021(3). - Sandhu A, Jayaram H, Hu K, Bunce C, Gazzard G: Ab interno supraciliary microstent surgery for open‐angle glaucoma. *Cochrane Database of Systematic Reviews* 2021(5). - Safi S, Sethi NJ, Korang SK, Nielsen EE, Feinberg J, Gluud C, Jakobsen JC: Beta‐blockers in patients without heart failure after myocardial infarction. *Cochrane Database of Systematic Reviews* 2021(11). - Rutherford D, Massie EM, Worsley C, Wilson MSJ: Intraperitoneal local anaesthetic instillation versus no intraperitoneal local anaesthetic instillation for laparoscopic cholecystectomy. *Cochrane Database of Systematic Reviews* 2021(10). - Roy M, Banerjee A, Chakravarty M: A study of electrocardiographic and lipid changes in patients with type II diabetes mellitus. *National Journal of Physiology, Pharmacy and Pharmacology* 2021, 11(10):1130-1130. - Rose L, Sutt AL, Amaral AC, Fergusson DA, Smith OM, Dale CM: Interventions to enable communication for adult patients requiring an artificial airway with or without mechanical ventilator support. *Cochrane Database of Systematic Reviews* 2021(10). - Rodriguez MI, Edelman A, Hersh A, Gartoulla P, Henderson J: Medical abortion offered in pharmacy versus clinic‐based settings. *Cochrane Database of Systematic Reviews* 2021(6). - Robertson L, Broderick C, Yeoh SE, Stansby G: Effect of testing for cancer on cancer‐ or venous thromboembolism (VTE)‐related mortality and morbidity in people with unprovoked VTE. *Cochrane Database of Systematic Reviews* 2021(10). - Roberts MT, Lloyd J, Välimäki M, Ho GWK, Freemantle M, Békefi AZ: Video games for people with schizophrenia. *Cochrane Database of Systematic Reviews* 2021(2). - Roaldsen MB, Lindekleiv H, Mathiesen EB: Intravenous thrombolytic treatment and endovascular thrombectomy for ischaemic wake‐up stroke. *Cochrane Database of Systematic Reviews* 2021(12). - Roaldsen MB, Jusufovic M, Berge E, Lindekleiv H: Endovascular thrombectomy and intra‐arterial interventions for acute ischaemic stroke. *Cochrane Database of Systematic Reviews* 2021(6). - Rerkasem A, Orrapin S, Howard DPJ, Nantakool S, Rerkasem K: Local versus general anaesthesia for carotid endarterectomy. *Cochrane Database of Systematic Reviews* 2021(10). - Qureshi N, Da Silva MLR, Abdul-Hamid H, Weng SF, Kai J, Leonardi-Bee J: Strategies for screening for familial hypercholesterolaemia in primary care and other community settings. *Cochrane Database of Systematic Reviews* 2021(10). - Quinn TJ, Fearon P, Noel-Storr AH, Young C, McShane R, Stott DJ: Informant Questionnaire on Cognitive Decline in the Elderly (IQCODE) for the detection of dementia within community dwelling populations. *Cochrane Database of Systematic Reviews* 2021(7). - Qin H, Reid I, Gorelik A, Ng L: Environmental enrichment for stroke and other non‐progressive brain injury. *Cochrane Database of Systematic Reviews* 2021(11). - Pugh D, O'Sullivan ED, Duthie FAI, Masson P, Kavanagh D: Interventions for atypical haemolytic uraemic syndrome. *Cochrane Database of Systematic Reviews* 2021(3). - Prieto JA, Murphy CL, Stewart F, Fader M: Intermittent catheter techniques, strategies and designs for managing long‐term bladder conditions. *Cochrane Database of Systematic Reviews* 2021(10). - Popp M, Stegemann M, Riemer M, Metzendorf MI, Romero CS, Mikolajewska A, Kranke P, Meybohm P, Skoetz N, Weibel S: Antibiotics for the treatment of COVID‐19. *Cochrane Database of Systematic Reviews* 2021(10). - Poot CC, Meijer E, Kruis AL, Smidt N, Chavannes NH, Honkoop PJ: Integrated disease management interventions for patients with chronic obstructive pulmonary disease. *Cochrane Database of Systematic Reviews* 2021(9). - Pontes FG, da Silva EMK, Baptista-Silva JCC, Vasconcelos V: Treatments for unruptured intracranial aneurysms. *Cochrane Database of Systematic Reviews* 2021(5). - Pisano A, Iannone LF, Leo A, Russo E, Coppolino G, Bolignano D: Renal denervation for resistant hypertension. *Cochrane Database of Systematic Reviews* 2021(11). - Petkovic J, Duench S, Trawin J, Dewidar O, Pardo Pardo J, Simeon R, DesMeules M, Gagnon D, Hatcher Roberts J, Hossain A *et al*: Behavioural interventions delivered through interactive social media for health behaviour change, health outcomes, and health equity in the adult population. *Cochrane Database of Systematic Reviews* 2021(5). - Peters SE, Jha B, Ross M: Rehabilitation following surgery for flexor tendon injuries of the hand. *Cochrane Database of Systematic Reviews* 2021(1). - Pellicori P, Doolub G, Wong CM, Lee KS, Mangion K, Ahmad M, Berry C, Squire I, Lambiase PD, Lyon A *et al*: COVID‐19 and its cardiovascular effects: a systematic review of prevalence studies. *Cochrane Database of Systematic Reviews* 2021(3). - Pattanittum P, Ngamjarus C, Buttramee F, Somboonporn C: Roselle for hypertension in adults. *Cochrane Database of Systematic Reviews* 2021(11). - Parsons C, Lim WY, Loy C, McGuinness B, Passmore P, Ward SA, Hughes C: Withdrawal or continuation of cholinesterase inhibitors or memantine or both, in people with dementia. *Cochrane Database of Systematic Reviews* 2021(2). - Parmar A, Macluskey M, Mc Goldrick N, Conway DI, Glenny AM, Clarkson JE, Worthington HV, Chan KKW: Interventions for the treatment of oral cavity and oropharyngeal cancer: chemotherapy. *Cochrane Database of Systematic Reviews* 2021(12). - Panebianco M, Al-Bachari S, Hutton JL, Marson AG: Gabapentin add‐on treatment for drug‐resistant focal epilepsy. *Cochrane Database of Systematic Reviews* 2021(1). - Pandey A, Vaduganathan M, Patel KV, Ayers C, Ballantyne CM, Kosiborod MN, Carnethon M, DeFilippi C, McGuire DK, Khan SS *et al*: Biomarker-Based Risk Prediction of Incident Heart Failure in Pre-Diabetes and Diabetes. *JACC Heart Fail* 2021, 9(3):215-223. - Pandey A, Vaduganathan M, Patel KV, Ayers C, Ballantyne CM, Kosiborod MN, Carnethon M, DeFilippi C, McGuire DK, Khan SS *et al*: Biomarker-Based Risk Prediction of Incident Heart Failure in Pre-Diabetes and Diabetes. *JACC Heart Fail* 2021, 9(3):215-223. - Palmer MJ, Machiyama K, Woodd S, Gubijev A, Barnard S, Russell S, Perel P, Free C: Mobile phone‐based interventions for improving adherence to medication prescribed for the primary prevention of cardiovascular disease in adults. *Cochrane Database of Systematic Reviews* 2021(3). - O'Shea O, Stovold E, Cates CJ: Regular treatment with formoterol and an inhaled corticosteroid versus regular treatment with salmeterol and an inhaled corticosteroid for chronic asthma: serious adverse events. *Cochrane Database of Systematic Reviews* 2021(4). - Orrapin S, Benyakorn T, Howard DPJ, Siribumrungwong B, Rerkasem K: Patches of different types for carotid patch angioplasty. *Cochrane Database of Systematic Reviews* 2021(2). - Onghanseng N, Ng SM, Halim MS, Nguyen QD: Oral antibiotics for chronic blepharitis. *Cochrane Database of Systematic Reviews* 2021(6). - Ong AY, Ng SM, Vedula SS, Friedman DS: Lens extraction for chronic angle‐closure glaucoma. *Cochrane Database of Systematic Reviews* 2021(3). - O'Connor T, Moore ZEH, Patton D: Patient and lay carer education for preventing pressure ulceration in at‐risk populations. *Cochrane Database of Systematic Reviews* 2021(2). - O'Connell NE, Ferraro MC, Gibson W, Rice ASC, Vase L, Coyle D, Eccleston C: Implanted spinal neuromodulation interventions for chronic pain in adults. *Cochrane Database of Systematic Reviews* 2021(12). - Ochodo EA, Guleid F, Deeks JJ, Mallett S: Point‐of‐care tests detecting HIV nucleic acids for diagnosis of HIV‐1 or HIV‐2 infection in infants and children aged 18 months or less. *Cochrane Database of Systematic Reviews* 2021(8). - Nussbaumer-Streit B, Thaler K, Chapman A, Probst T, Winkler D, Sönnichsen A, Gaynes BN, Gartlehner G: Second‐generation antidepressants for treatment of seasonal affective disorder. *Cochrane Database of Systematic Reviews* 2021(3). - Nishizaki D, Ganeko R, Hoshino N, Hida K, Obama K, Furukawa TA, Sakai Y, Watanabe N: Roux‐en‐Y versus Billroth‐I reconstruction after distal gastrectomy for gastric cancer. *Cochrane Database of Systematic Reviews* 2021(9). - Nijpels G, Van Der Heijden AA, Elders P, Beulens JW, De Vet HC: The interobserver agreement of ECG abnormalities using Minnesota codes in people with type 2 diabetes. *Plos one* 2021, 16(8):e0255466. - Ng WS, Jayaram H: Adjunctive modulation of wound healing during cataract surgery to promote survival of a previous trabeculectomy. *Cochrane Database of Systematic Reviews* 2021(8). - Ng SM, Ren M, Lindsley KB, Hawkins BS, Kuo IC: Transepithelial versus epithelium‐off corneal crosslinking for progressive keratoconus. *Cochrane Database of Systematic Reviews* 2021(3). - Nathavitharana RR, Lederer P, Chaplin M, Bjerrum S, Steingart KR, Shah M: Impact of diagnostic strategies for tuberculosis using lateral flow urine lipoarabinomannan assay in people living with HIV. *Cochrane Database of Systematic Reviews* 2021(8). - Nanda A, Hu J, Hodgkinson S, Ali S, Rainsbury R, Roy PG: Oncoplastic breast‐conserving surgery for women with primary breast cancer. *Cochrane Database of Systematic Reviews* 2021(10). - Nakano LCU, Cacione DG, Baptista-Silva JCC, Flumignan RLG: Treatment for telangiectasias and reticular veins. *Cochrane Database of Systematic Reviews* 2021(10). - Nadarevic T, Giljaca V, Colli A, Fraquelli M, Casazza G, Miletic D, Štimac D: Computed tomography for the diagnosis of hepatocellular carcinoma in adults with chronic liver disease. *Cochrane Database of Systematic Reviews* 2021(10). - Musters AH, Mashayekhi S, Harvey J, Axon E, Lax SJ, Flohr C, Drucker AM, Gerbens L, Ferguson J, Ibbotson S *et al*: Phototherapy for atopic eczema. *Cochrane Database of Systematic Reviews* 2021(10). - Mühlbauer V, Möhler R, Dichter MN, Zuidema SU, Köpke S, Luijendijk HJ: Antipsychotics for agitation and psychosis in people with Alzheimer's disease and vascular dementia. *Cochrane Database of Systematic Reviews* 2021(12). - Motaze NV, Chi PC, Ongolo-Zogo P, Ndongo JS, Wiysonge CS: Government regulation of private health insurance. *Cochrane Database of Systematic Reviews* 2021(2). - Morrow B, Argent A, Zampoli M, Human A, Corten L, Toussaint M: Cough augmentation techniques for people with chronic neuromuscular disorders. *Cochrane Database of Systematic Reviews* 2021(4). - Moriarty AS, Meader N, Snell KIE, Riley RD, Paton LW, Chew-Graham CA, Gilbody S, Churchill R, Phillips RS, Ali S *et al*: Prognostic models for predicting relapse or recurrence of major depressive disorder in adults. *Cochrane Database of Systematic Reviews* 2021(5). - Monk EJM, Abba K, Ranganathan LN: Anthelmintics for people with neurocysticercosis. *Cochrane Database of Systematic Reviews* 2021(6). - Mohammady M, Radmehr M, Janani L: Slow versus fast subcutaneous heparin injections for prevention of bruising and site pain intensity. *Cochrane Database of Systematic Reviews* 2021(6). - Mohamed I, Kamarizan MF, Da Silva A: Medical adjuvant treatment to increase patency of arteriovenous fistulae and grafts. *Cochrane Database of Systematic Reviews* 2021(7). - Mishu MP, Uphoff E, Aslam F, Philip S, Wright J, Tirbhowan N, Ajjan RA, Al Azdi Z, Stubbs B, Churchill R *et al*: Interventions for preventing type 2 diabetes in adults with mental disorders in low‐ and middle‐income countries. *Cochrane Database of Systematic Reviews* 2021(2). - Minozzi S, Pifferi S, Brazzi L, Pecoraro V, Montrucchio G, D'Amico R: Topical antibiotic prophylaxis to reduce respiratory tract infections and mortality in adults receiving mechanical ventilation. *Cochrane Database of Systematic Reviews* 2021(1). - Milburn-McNulty P, Panebianco M, Marson AG: Sulthiame monotherapy for epilepsy. *Cochrane Database of Systematic Reviews* 2021(9). - Mikolajewska A, Fischer AL, Piechotta V, Mueller A, Metzendorf MI, Becker M, Dorando E, Pacheco RL, Martimbianco ALC, Riera R *et al*: Colchicine for the treatment of COVID‐19. *Cochrane Database of Systematic Reviews* 2021(10). - Miao C, Yang X, Wong MCM, Zou J, Zhou X, Li C, Wang Y: Rubber dam isolation for restorative treatment in dental patients. *Cochrane Database of Systematic Reviews* 2021(5). - McNicol ED, Ferguson MC, Schumann R: Single‐dose intravenous ketorolac for acute postoperative pain in adults. *Cochrane Database of Systematic Reviews* 2021(5). - McLain NEM, Moore ZEH, Avsar P: Wound cleansing for treating venous leg ulcers. *Cochrane Database of Systematic Reviews* 2021(3). - McGaughey J, Fergusson DA, Van Bogaert P, Rose L: Early warning systems and rapid response systems for the prevention of patient deterioration on acute adult hospital wards. *Cochrane Database of Systematic Reviews* 2021(11). - McCleery J, Laverty J, Quinn TJ: Diagnostic test accuracy of telehealth assessment for dementia and mild cognitive impairment. *Cochrane Database of Systematic Reviews* 2021(7). - Mathes T, Prediger B, Walgenbach M, Siegel R: Mesh fixation techniques in primary ventral or incisional hernia repair. *Cochrane Database of Systematic Reviews* 2021(5). - Martin N, Manoharan K, Davies C, Lumbers RT: Beta‐blockers and inhibitors of the renin‐angiotensin aldosterone system for chronic heart failure with preserved ejection fraction. *Cochrane Database of Systematic Reviews* 2021(5). - Martí-Carvajal AJ, Knight-Madden JM, Martinez-Zapata MJ: Interventions for treating leg ulcers in people with sickle cell disease. *Cochrane Database of Systematic Reviews* 2021(1). - Malaguti C, Dal Corso S, Janjua S, Holland AE: Supervised maintenance programmes following pulmonary rehabilitation compared to usual care for chronic obstructive pulmonary disease. *Cochrane Database of Systematic Reviews* 2021(8). - Mahdian M, Behboodi S, Ogata Y, Natto ZS: Laser therapy for dentinal hypersensitivity. *Cochrane Database of Systematic Reviews* 2021(7). - Maguire MJ, Marson AG, Nevitt SJ: Antidepressants for people with epilepsy and depression. *Cochrane Database of Systematic Reviews* 2021(4). - Mafla AC, Herrera‐López HM, Eraso TF, Melo MA, Munoz N, Schwendicke F: Smartphones addiction associated with academic achievement among dental students: a cross‐sectional study. *Journal of dental education* 2021, 85(11):1802-1809. - Maedel S, Evans JR, Harrer-Seely A, Findl O: Intraocular lens optic edge design for the prevention of posterior capsule opacification after cataract surgery. *Cochrane Database of Systematic Reviews* 2021(8). - Madhukar R, Jagadeesh AT, Moey MYY, Vaglio M, Badilini F, Leban M, Hartemann A, Dureau P, Funck-Brentano C, Bourron O *et al*: Association of thyroid-stimulating hormone with corrected QT interval variation: A prospective cohort study among patients with type 2 diabetes. *Arch Cardiovasc Dis* 2021, 114(10):656-666. - Macey R, Walsh T, Riley P, Hogan R, Glenny AM, Worthington HV, Clarkson JE, Ricketts D: Transillumination and optical coherence tomography for the detection and diagnosis of enamel caries. *Cochrane Database of Systematic Reviews* 2021(1). - Macey R, Walsh T, Riley P, Glenny AM, Worthington HV, O'Malley L, Clarkson JE, Ricketts D: Visual or visual‐tactile examination to detect and inform the diagnosis of enamel caries. *Cochrane Database of Systematic Reviews* 2021(6). - Macey R, Walsh T, Riley P, Glenny AM, Worthington HV, Clarkson JE, Ricketts D: Electrical conductance for the detection of dental caries. *Cochrane Database of Systematic Reviews* 2021(3). - Maagaard M, Karlsson WK, Ovesen C, Gluud C, Jakobsen JC: Interventions for altering blood pressure in people with acute subarachnoid haemorrhage. *Cochrane Database of Systematic Reviews* 2021(11). - Lyhne MK, Vegge A, Povlsen GK, Slaaby R, Kildegaard J, Pedersen-Bjergaard U, Olsen LH: Hyperinsulinaemic hypoglycaemia in non-anaesthetized Göttingen minipigs induces a counter-regulatory endocrine response and electrocardiographic changes. *Scientific reports* 2021, 11(1):5983-5983.   Luvira V, Satitkarnmanee E, Pugkhem A, Kietpeerakool C, Lumbiganon P, Pattanittum P: Postoperative adjuvant chemotherapy for resectable cholangiocarcinoma. *Cochrane Database of Systematic Reviews* 2021(9).   - Lu C, Zheng J, Cao Y, Bresnahan R, Martin-McGill KJ: Carisbamate add‐on therapy for drug‐resistant focal epilepsy. *Cochrane Database of Systematic Reviews* 2021(12). - Lowe D, Ryan R, Schonfeld L, Merner B, Walsh L, Graham-Wisener L, Hill S: Effects of consumers and health providers working in partnership on health services planning, delivery and evaluation. *Cochrane Database of Systematic Reviews* 2021(9). - Longley V, Hazelton C, Heal C, Pollock A, Woodward-Nutt K, Mitchell C, Pobric G, Vail A, Bowen A: Non‐pharmacological interventions for spatial neglect or inattention following stroke and other non‐progressive brain injury. *Cochrane Database of Systematic Reviews* 2021(7). - Lodi G, Azzi L, Varoni EM, Pentenero M, Del Fabbro M, Carrassi A, Sardella A, Manfredi M: Antibiotics to prevent complications following tooth extractions. *Cochrane Database of Systematic Reviews* 2021(2). - Liu C, Chen J, Gao Y, Deng B, Liu K: Endothelin receptor antagonists for pulmonary arterial hypertension. *Cochrane Database of Systematic Reviews* 2021(3). - Liu Ay, Cheuk DKL: Disease‐modifying treatments for primary autoimmune haemolytic anaemia. *Cochrane Database of Systematic Reviews* 2021(3). - Lindson N, Pritchard G, Hong B, Fanshawe TR, Pipe A, Papadakis S: Strategies to improve smoking cessation rates in primary care. *Cochrane Database of Systematic Reviews* 2021(9). - Lim PK, Amer Nordin AS, Yee A, Tan SB: Prevalence of smartphone addiction in patients with depression and its association with depression severity: a cross-sectional study. *International Journal of Mental Health and Addiction* 2021, 19:919-933. - Li Z, Li Z, Zhao L, Cheng Y, Cheng N, Deng Y: Abdominal drainage to prevent intra‐peritoneal abscess after appendectomy for complicated appendicitis. *Cochrane Database of Systematic Reviews* 2021(8). - Li Y, Ma J, Lu G, Dou Z, Knaggs R, Xia J, Zhao S, Dong S, Yang L: Hydromorphone for cancer pain. *Cochrane Database of Systematic Reviews* 2021(8). - Li T, Luo H-H, Feng X-F, Bai Y, Fang Z-Z, Wu G-G, Wu J-L: Plasma free amino acids and risk of cardiovascular disease in Chinese patients with type 2 diabetes. *Frontiers in Endocrinology* 2021, 11:519923. - Li LR, Chaudhary B, You C, Dennis JA, Wakeford H: Glucocorticoid with cyclophosphamide for oral paraquat poisoning. *Cochrane Database of Systematic Reviews* 2021(6). - Lewis SR, Macey R, Eardley WGP, Dixon JR, Cook J, Griffin XL: Internal fixation implants for intracapsular hip fractures in older adults. *Cochrane Database of Systematic Reviews* 2021(3). - Lewis SR, Baker PE, Parker R, Smith AF: High‐flow nasal cannulae for respiratory support in adult intensive care patients. *Cochrane Database of Systematic Reviews* 2021(3). - Leow MQ, Zheng Q, Shi L, Tay SC, Chan ESY: Non‐steroidal anti‐inflammatory drugs (NSAIDs) for trigger finger. *Cochrane Database of Systematic Reviews* 2021(4). - Leone MA, Giussani G, Nevitt SJ, Marson AG, Beghi E: Immediate antiepileptic drug treatment, versus placebo, deferred, or no treatment for first unprovoked seizure. *Cochrane Database of Systematic Reviews* 2021(5). - Legg LA, Rudberg AS, Hua X, Wu S, Hackett ML, Tilney R, Lindgren L, Kutlubaev MA, Hsieh CF, Barugh AJ *et al*: Selective serotonin reuptake inhibitors (SSRIs) for stroke recovery. *Cochrane Database of Systematic Reviews* 2021(11). - Lee HW, Lee MS, Kim TH, Alraek T, Zaslawski C, Kim JW, Moon DG: Ginseng for erectile dysfunction. *Cochrane Database of Systematic Reviews* 2021(4). - Lee AL, Gordon CS, Osadnik CR: Exercise training for bronchiectasis. *Cochrane Database of Systematic Reviews* 2021(4). - Lee A, Tysome JR, Saeed SR: Topical azole treatments for otomycosis. *Cochrane Database of Systematic Reviews* 2021(5). - Leache L, Gutiérrez-Valencia M, Finizola RM, Infante E, Finizola B, Pardo Pardo J, Flores Y, Granero R, Arai KJ: Pharmacotherapy for hypertension‐induced left ventricular hypertrophy. *Cochrane Database of Systematic Reviews* 2021(10). - Kubrusly M, Silva PGdB, Vasconcelos GVd, Leite EDLG, Santos PdA, Rocha HAL: Nomophobia among medical students and its association with depression, anxiety, stress and academic performance. *Revista Brasileira de Educação Médica* 2021, 45:e162. - Kruizinga J, Liemburg E, Burger H, Cipriani A, Geddes J, Robertson L, Vogelaar B, Nolen WA: Pharmacological treatment for psychotic depression. *Cochrane Database of Systematic Reviews* 2021(12). - Kreuzberger N, Hirsch C, Chai KL, Tomlinson E, Khosravi Z, Popp M, Neidhardt M, Piechotta V, Salomon S, Valk SJ *et al*: SARS‐CoV‐2‐neutralising monoclonal antibodies for treatment of COVID‐19. *Cochrane Database of Systematic Reviews* 2021(9). - Kornelsen E, Mahant S, Parkin P, Ren LY, Reginald YA, Shah SS, Gill PJ: Corticosteroids for periorbital and orbital cellulitis. *Cochrane Database of Systematic Reviews* 2021(4). - Komolafe O, Buzzetti E, Linden A, Best LMJ, Madden AM, Roberts D, Chase TJG, Fritche D, Freeman SC, Cooper NJ *et al*: Nutritional supplementation for nonalcohol‐related fatty liver disease: a network meta‐analysis. *Cochrane Database of Systematic Reviews* 2021(7). - Kokkinou M, Beishon LC, Smailagic N, Noel-Storr AH, Hyde C, Ukoumunne O, Worrall RE, Hayen A, Desai M, Ashok AH *et al*: Plasma and cerebrospinal fluid ABeta42 for the differential diagnosis of Alzheimer's disease dementia in participants diagnosed with any dementia subtype in a specialist care setting. *Cochrane Database of Systematic Reviews* 2021(2). - Kohli M, Schiller I, Dendukuri N, Yao M, Dheda K, Denkinger CM, Schumacher SG, Steingart KR: Xpert MTB/RIF Ultra and Xpert MTB/RIF assays for extrapulmonary tuberculosis and rifampicin resistance in adults. *Cochrane Database of Systematic Reviews* 2021(1). - Klifto KM, Elhelali A, Payne RM, Cooney CM, Manahan MA, Rosson GD: Perioperative systemic nonsteroidal anti‐inflammatory drugs (NSAIDs) in women undergoing breast surgery. *Cochrane Database of Systematic Reviews* 2021(11). - Klatte K, Pauli-Magnus C, Love SB, Sydes MR, Benkert P, Bruni N, Ewald H, Arnaiz Jimenez P, Bonde MM, Briel M: Monitoring strategies for clinical intervention studies. *Cochrane Database of Systematic Reviews* 2021(12). - Kavanagh EP, Sultan S, Jordan F, Elhelali A, Devane D, Veerasingam D, Hynes N: Hybrid repair versus conventional open repair for aortic arch dissection. *Cochrane Database of Systematic Reviews* 2021(7). - Kanie T, Mizuno A, Takaoka Y, Suzuki T, Yoneoka D, Nishikawa Y, Tam WW, Morze J, Rynkiewicz A, Xin Y *et al*: Dipeptidyl peptidase‐4 inhibitors, glucagon‐like peptide 1 receptor agonists and sodium‐glucose co‐transporter‐2 inhibitors for people with cardiovascular disease: a network meta‐analysis. *Cochrane Database of Systematic Reviews* 2021(10). - Kampling H, Baumeister H, Bengel J, Mittag O: Prevention of depression in adults with long‐term physical conditions. *Cochrane Database of Systematic Reviews* 2021(3). - Kalra N, Hooker L, Reisenhofer S, Di Tanna GL, García-Moreno C: Training healthcare providers to respond to intimate partner violence against women. *Cochrane Database of Systematic Reviews* 2021(5). - Kahale LA, Matar CF, Tsolakian IG, Hakoum MB, Yosuico VED, Terrenato I, Sperati F, Barba M, Hicks LK, Schünemann H *et al*: Antithrombotic therapy for ambulatory patients with multiple myeloma receiving immunomodulatory agents. *Cochrane Database of Systematic Reviews* 2021(9). - Kahale LA, Matar CF, Tsolakian IG, Hakoum MB, Barba M, Yosuico VED, Terrenato I, Sperati F, Schünemann H, Akl EA: Oral anticoagulation in people with cancer who have no therapeutic or prophylactic indication for anticoagulation. *Cochrane Database of Systematic Reviews* 2021(10). - Kahale LA, Matar CF, Hakoum MB, Tsolakian IG, Yosuico VED, Terrenato I, Sperati F, Barba M, Schünemann H, Akl EA: Anticoagulation for the initial treatment of venous thromboembolism in people with cancer. *Cochrane Database of Systematic Reviews* 2021(12). - Kaelin Agten A, Xia J, Servante JA, Thornton JG, Jones NW: Routine ultrasound for fetal assessment before 24 weeks' gestation. *Cochrane Database of Systematic Reviews* 2021(8). - Jull J, Köpke S, Smith M, Carley M, Finderup J, Rahn AC, Boland L, Dunn S, Dwyer AA, Kasper J *et al*: Decision coaching for people making healthcare decisions. *Cochrane Database of Systematic Reviews* 2021(11). - Jordan F, FitzGibbon B, Kavanagh EP, McHugh P, Veerasingam D, Sultan S, Hynes N: Endovascular versus open surgical repair for complicated chronic Type B aortic dissection. *Cochrane Database of Systematic Reviews* 2021(12). - Jones E, Stewart F, Taylor B, Davis PG, Brown SJ: Early postnatal discharge from hospital for healthy mothers and term infants. *Cochrane Database of Systematic Reviews* 2021(6). - Jiang Q, Chen J, Tian F, Liu Z: Silicone gel sheeting for treating hypertrophic scars. *Cochrane Database of Systematic Reviews* 2021(9). - Jiang J, Zhang C, Li C, Chen Z, Cao X, Wang H, Li W, Wang J: Magnetic seizure therapy for treatment‐resistant depression. *Cochrane Database of Systematic Reviews* 2021(6). - Jia L, Meng Q, Scott A, Yuan B, Zhang L: Payment methods for healthcare providers working in outpatient healthcare settings. *Cochrane Database of Systematic Reviews* 2021(1). - Jeyashree K, Sathiavadivu JS, Suliankatchi A: App-based tracking of smartphone use and its association with perceived stress and sense of coherence among undergraduate medical students in Southern India. *International Journal of Adolescent Medicine and Health* 2021, 33(3):245-251. - Jeyashree K, Sathiavadivu JS, Suliankatchi A: App-based tracking of smartphone use and its association with perceived stress and sense of coherence among undergraduate medical students in Southern India. *International journal of adolescent medicine and health* 2021, 33(3):245-251. - Jat KR, Walia DK, Khairwa A: Anti‐IgE therapy for allergic bronchopulmonary aspergillosis in people with cystic fibrosis. *Cochrane Database of Systematic Reviews* 2021(9). - Janjua S, Pike KC, Carr R, Coles A, Fortescue R, Batavia M: Interventions to improve adherence to pharmacological therapy for chronic obstructive pulmonary disease (COPD). *Cochrane Database of Systematic Reviews* 2021(9). - Janjua S, Mathioudakis AG, Fortescue R, Walker RAE, Sharif S, Threapleton CJD, Dias S: Prophylactic antibiotics for adults with chronic obstructive pulmonary disease: a network meta‐analysis. *Cochrane Database of Systematic Reviews* 2021(1). - Janjua S, Carter D, Threapleton CJD, Prigmore S, Disler RT: Telehealth interventions: remote monitoring and consultations for people with chronic obstructive pulmonary disease (COPD). *Cochrane Database of Systematic Reviews* 2021(7). - Janjua S, Banchoff E, Threapleton CJD, Prigmore S, Fletcher J, Disler RT: Digital interventions for the management of chronic obstructive pulmonary disease. *Cochrane Database of Systematic Reviews* 2021(4). - Jahanfar S, Ho JJ, Jaafar SH, Abraha I, Noura M, Ross CR, Pammi M: Ultrasound for diagnosis of birth weight discordance in twin pregnancies. *Cochrane Database of Systematic Reviews* 2021(3). - Imdad A, Mackoff SP, Urciuoli DM, Syed T, Tanner-Smith EE, Huang D, Gomez-Duarte OG: Interventions for preventing diarrhoea‐associated haemolytic uraemic syndrome. *Cochrane Database of Systematic Reviews* 2021(7). - Hynes N, Kavanagh EP, Sultan S, Jordan F: Surgical and radiological interventions for treating symptomatic extracranial cervical artery dissection. *Cochrane Database of Systematic Reviews* 2021(2). - Hu K, Shah A, Virgili G, Bunce C, Gazzard G: Ab interno trabecular bypass surgery with Trabectome for open‐angle glaucoma. *Cochrane Database of Systematic Reviews* 2021(2). - Htay H, Johnson DW, Craig JC, Teixeira-Pinto A, Hawley CM, Cho Y: Urgent‐start peritoneal dialysis versus haemodialysis for people with chronic kidney disease. *Cochrane Database of Systematic Reviews* 2021(1). - Horn D, Ehret D, Gautham KS, Soll R: Sunlight for the prevention and treatment of hyperbilirubinemia in term and late preterm neonates. *Cochrane Database of Systematic Reviews* 2021(7). - Hoon SN, Lau PH, White AM, Bulsara MK, Banks PD, Redfern AD: Capecitabine for hormone receptor‐positive versus hormone receptor‐negative breast cancer. *Cochrane Database of Systematic Reviews* 2021(5). - Holliday R, Hong B, McColl E, Livingstone-Banks J, Preshaw PM: Interventions for tobacco cessation delivered by dental professionals. *Cochrane Database of Systematic Reviews* 2021(2). - Hohlfeld A, Ebrahim S, Shaik MZ, Kredo T: Circumcision devices versus standard surgical techniques in adolescent and adult male circumcisions. *Cochrane Database of Systematic Reviews* 2021(3). - Henein C, Steel DHW: Photobiomodulation for non‐exudative age‐related macular degeneration. *Cochrane Database of Systematic Reviews* 2021(5). - Hemmingsen B, Metzendorf MI, Richter B: (Ultra‐)long‐acting insulin analogues for people with type 1 diabetes mellitus. *Cochrane Database of Systematic Reviews* 2021(3). - Haveman LM, van Ewijk R, van Dalen EC, Breunis WB, Kremer LCM, van den Berg H, Dirksen U, Merks JHM: High‐dose chemotherapy followed by autologous haematopoietic cell transplantation for children, adolescents, and young adults with primary metastatic Ewing sarcoma. *Cochrane Database of Systematic Reviews* 2021(9). - Hashimoto Y, Kotake K, Watanabe N, Fujiwara T, Sakamoto S: Lamotrigine in the maintenance treatment of bipolar disorder. *Cochrane Database of Systematic Reviews* 2021(9). - Hasegawa T, Nishiwaki H, Ota E, Levack WMM, Noma H: Aldosterone antagonists for people with chronic kidney disease requiring dialysis. *Cochrane Database of Systematic Reviews* 2021(2). - Harms PP, van der Heijden AA, Rutters F, Tan HL, Beulens JW, Nijpels G, Elders P: Prevalence of ECG abnormalities in people with type 2 diabetes: the Hoorn Diabetes Care System cohort. *Journal of Diabetes and its Complications* 2021, 35(2):107810. - Harms PP, van der Heijden AA, Rutters F, Tan HL, Beulens JW, Nijpels G, Elders P: Prevalence of ECG abnormalities in people with type 2 diabetes: the Hoorn Diabetes Care System cohort. *Journal of Diabetes and its Complications* 2021, 35(2):107810. - Harms PP, van der Heijden AA, Rutters F, Tan HL, Beulens JW, Nijpels G, Elders P: Prevalence of ECG abnormalities in people with type 2 diabetes: the Hoorn Diabetes Care System cohort. *Journal of Diabetes and its Complications* 2021, 35(2):107810. - Haraka F, Kakolwa M, Schumacher SG, Nathavitharana RR, Denkinger CM, Gagneux S, Reither K, Ross A: Impact of the diagnostic test Xpert MTB/RIF on patient outcomes for tuberculosis. *Cochrane Database of Systematic Reviews* 2021(5). - Hara T, Hijikata Y, Matsubara Y, Watanabe N: Pharmacological interventions versus placebo, no treatment or usual care for osteoporosis in people with chronic kidney disease stages 3‐5D. *Cochrane Database of Systematic Reviews* 2021(7). - Handoll HHG, Cameron ID, Mak JCS, Panagoda CE, Finnegan TP: Multidisciplinary rehabilitation for older people with hip fractures. *Cochrane Database of Systematic Reviews* 2021(11). - Gutierrez-Arias R, Martinez-Zapata MJ, Gaete-Mahn MC, Osorio D, Bustos L, Melo Tanner J, Hidalgo R, Seron P: Exercise training for adult lung transplant recipients. *Cochrane Database of Systematic Reviews* 2021(7). - Guo W, Zhao L, Mo F, Peng C, Li L, Xu Y, Guo W, Sun A, Yan H, Wang L: The prognostic value of the triglyceride glucose index in patients with chronic heart failure and type 2 diabetes: a retrospective cohort study. *Diabetes Research and Clinical Practice* 2021, 177:108786. - Guo W, Zhao L, Mo F, Peng C, Li L, Xu Y, Guo W, Sun A, Yan H, Wang L: The prognostic value of the triglyceride glucose index in patients with chronic heart failure and type 2 diabetes: A retrospective cohort study. *Diabetes Research and Clinical Practice* 2021, 177:108786. - Greenhalgh J, Boland A, Bates V, Vecchio F, Dundar Y, Chaplin M, Green JA: First‐line treatment of advanced epidermal growth factor receptor (EGFR) mutation positive non‐squamous non‐small cell lung cancer. *Cochrane Database of Systematic Reviews* 2021(3). - Grande AJ, Silva V, Sawaris Neto L, Teixeira Basmage JP, Peccin MS, Maddocks M: Exercise for cancer cachexia in adults. *Cochrane Database of Systematic Reviews* 2021(3). - Gordon M, Sinopoulou V, Iheozor-Ejiofor Z, Iqbal T, Allen P, Hoque S, Engineer J, Akobeng AK: Interventions for treating iron deficiency anaemia in inflammatory bowel disease. *Cochrane Database of Systematic Reviews* 2021(1). - Goel RR, Hardy SC, Brown T: Surgery for deep venous insufficiency. *Cochrane Database of Systematic Reviews* 2021(9). - Glenton C, Carlsen B, Lewin S, Wennekes MD, Winje BA, Eilers R: Healthcare workers’ perceptions and experiences of communicating with people over 50 years of age about vaccination: a qualitative evidence synthesis. *Cochrane Database of Systematic Reviews* 2021(7). - Gilligan C, Powell M, Lynagh MC, Ward BM, Lonsdale C, Harvey P, James EL, Rich D, Dewi SP, Nepal S *et al*: Interventions for improving medical students' interpersonal communication in medical consultations. *Cochrane Database of Systematic Reviews* 2021(2). - Gillies K, Kearney A, Keenan C, Treweek S, Hudson J, Brueton VC, Conway T, Hunter A, Murphy L, Carr PJ *et al*: Strategies to improve retention in randomised trials. *Cochrane Database of Systematic Reviews* 2021(3). - Gill PJ, Anwar MR, Kornelsen E, Parkin P, Mahood Q, Mahant S: Parenteral versus enteral fluid therapy for children hospitalised with bronchiolitis. *Cochrane Database of Systematic Reviews* 2021(12). - Gibbons T, Georgiou EX, Cheong YC, Wise MR: Levonorgestrel‐releasing intrauterine device (LNG‐IUD) for symptomatic endometriosis following surgery. *Cochrane Database of Systematic Reviews* 2021(12). - Gibbons C, Porter I, Gonçalves-Bradley DC, Stoilov S, Ricci-Cabello I, Tsangaris E, Gangannagaripalli J, Davey A, Gibbons EJ, Kotzeva A *et al*: Routine provision of feedback from patient‐reported outcome measurements to healthcare providers and patients in clinical practice. *Cochrane Database of Systematic Reviews* 2021(10). - Garegnani L, Hyland M, Roson Rodriguez P, Escobar Liquitay CM, Franco JVA: Antioxidants to prevent respiratory decline in people with Duchenne muscular dystrophy and progressive respiratory decline. *Cochrane Database of Systematic Reviews* 2021(12). - Gallagher A, Edwards M, Nair P, Drew S, Vyas A, Sharma R, Marsden PA, Wang R, Evans DJW: Anti‐interleukin‐13 and anti‐interleukin‐4 agents versus placebo, anti‐interleukin‐5 or anti‐immunoglobulin‐E agents, for people with asthma. *Cochrane Database of Systematic Reviews* 2021(10). - Fu S, Chen X, Zheng H: Exploring an adverse impact of smartphone overuse on academic performance via health issues: a stimulus-organism-response perspective. *Behaviour & Information Technology* 2021, 40(7):663-675. - Franco JVA, Jung JH, Imamura M, Borofsky M, Omar MI, Escobar Liquitay CM, Young S, Golzarian J, Veroniki AA, Garegnani L *et al*: Minimally invasive treatments for lower urinary tract symptoms in men with benign prostatic hyperplasia: a network meta‐analysis. *Cochrane Database of Systematic Reviews* 2021(7). - Franco JVA, Garegnani L, Escobar Liquitay CM, Borofsky M, Dahm P: Transurethral microwave thermotherapy for the treatment of lower urinary tract symptoms in men with benign prostatic hyperplasia. *Cochrane Database of Systematic Reviews* 2021(6). - Fountain DM, Bryant A, Barone DG, Waqar M, Hart MG, Bulbeck H, Kernohan A, Watts C, Jenkinson MD: Intraoperative imaging technology to maximise extent of resection for glioma: a network meta‐analysis. *Cochrane Database of Systematic Reviews* 2021(1). - Forsetlund L, O'Brien MA, Forsén L, Mwai L, Reinar LM, Okwen MP, Horsley T, Rose CJ: Continuing education meetings and workshops: effects on professional practice and healthcare outcomes. *Cochrane Database of Systematic Reviews* 2021(9). - Fite MB, Roba KT, Oljira L, Tura AK, Yadeta TA: Compliance with Iron and Folic Acid Supplementation (IFAS) and associated factors among pregnant women in Sub-Saharan Africa: A systematic review and meta-analysis. *PLoS One* 2021, 16(4):e0249789. - Fite MB, Assefa N, Mengiste B: Prevalence and determinants of Anemia among pregnant women in sub-Saharan Africa: a systematic review and Meta-analysis. *Archives of Public Health* 2021, 79:1-11. - Fisher J, Linder A, Calevo MG, Bentzer P: Non‐corticosteroid adjuvant therapies for acute bacterial meningitis. *Cochrane Database of Systematic Reviews* 2021(11). - Ferrara R, Imbimbo M, Malouf R, Paget-Bailly S, Calais F, Marchal C, Westeel V: Single or combined immune checkpoint inhibitors compared to first‐line platinum‐based chemotherapy with or without bevacizumab for people with advanced non‐small cell lung cancer. *Cochrane Database of Systematic Reviews* 2021(4). - Ferguson MC, Schumann R, Gallagher S, McNicol ED: Single‐dose intravenous ibuprofen for acute postoperative pain in adults. *Cochrane Database of Systematic Reviews* 2021(9). - Fenton C, Tan AR, Abaraogu UO, McCaslin JE: Prehabilitation exercise therapy before elective abdominal aortic aneurysm repair. *Cochrane Database of Systematic Reviews* 2021(7). - Ellahi A, Stewart F, Kidd EA, Griffiths R, Fernandez R, Omar MI: Strategies for the removal of short‐term indwelling urethral catheters in adults. *Cochrane Database of Systematic Reviews* 2021(6). - Elhelali A, Hynes N, Devane D, Sultan S, Kavanagh EP, Morris L, Veerasingam D, Jordan F: Hybrid repair versus conventional open repair for thoracic aortic arch aneurysms. *Cochrane Database of Systematic Reviews* 2021(6). - D'Souza N, Hicks G, Beable R, Higginson A, Rud B: Magnetic resonance imaging (MRI) for diagnosis of acute appendicitis. *Cochrane Database of Systematic Reviews* 2021(12). - Dong Y, Li G: Cardiac abnormalities in patients with nonalcoholic fatty liver disease : Insights from auxiliary examinations. *Herz* 2021, 46(2):158-163. - Dong Y, Li G: Cardiac abnormalities in patients with nonalcoholic fatty liver disease : Insights from auxiliary examinations. *Herz* 2021, 46(2):158-163. - Doleman B, Leonardi-Bee J, Heinink TP, Boyd-Carson H, Carrick L, Mandalia R, Lund JN, Williams JP: Pre‐emptive and preventive NSAIDs for postoperative pain in adults undergoing all types of surgery. *Cochrane Database of Systematic Reviews* 2021(6). - Dibben G, Faulkner J, Oldridge N, Rees K, Thompson DR, Zwisler AD, Taylor RS: Exercise‐based cardiac rehabilitation for coronary heart disease. *Cochrane Database of Systematic Reviews* 2021(11). - Diaz-Navarro R, Urrútia G, Cleland JGF, Poloni D, Villagran F, Acosta-Dighero R, Bangdiwala SI, Rada G, Madrid E: Stem cell therapy for dilated cardiomyopathy. *Cochrane Database of Systematic Reviews* 2021(7). - Diaconu K, Falconer J, Verbel A, Fretheim A, Witter S: Paying for performance to improve the delivery of health interventions in low‐ and middle‐income countries. *Cochrane Database of Systematic Reviews* 2021(5). - Dennett EJ, Janjua S, Stovold E, Harrison SL, McDonnell MJ, Holland AE: Tailored or adapted interventions for adults with chronic obstructive pulmonary disease and at least one other long‐term condition: a mixed methods review. *Cochrane Database of Systematic Reviews* 2021(7). - Dean RL, Marquardt T, Hurducas C, Spyridi S, Barnes A, Smith R, Cowen PJ, McShane R, Hawton K, Malhi GS *et al*: Ketamine and other glutamate receptor modulators for depression in adults with bipolar disorder. *Cochrane Database of Systematic Reviews* 2021(10). - Dean RL, Hurducas C, Hawton K, Spyridi S, Cowen PJ, Hollingsworth S, Marquardt T, Barnes A, Smith R, McShane R *et al*: Ketamine and other glutamate receptor modulators for depression in adults with unipolar major depressive disorder. *Cochrane Database of Systematic Reviews* 2021(9). - de Bruijn CM, Rexwinkel R, Gordon M, Benninga M, Tabbers MM: Antidepressants for functional abdominal pain disorders in children and adolescents. *Cochrane Database of Systematic Reviews* 2021(2). - de Ávila Oliveira R, Riera R, Vasconcelos V, Baptista-Silva JCC: Injection sclerotherapy for varicose veins. *Cochrane Database of Systematic Reviews* 2021(12). - Crossingham I, Turner S, Ramakrishnan S, Fries A, Gowell M, Yasmin F, Richardson R, Webb P, O'Boyle E, Hinks TSC: Combination fixed‐dose beta agonist and steroid inhaler as required for adults or children with mild asthma. *Cochrane Database of Systematic Reviews* 2021(5). - Crane H, Boam G, Carradice D, Vanicek N, Twiddy M, Smith GE: Through‐knee versus above‐knee amputation for vascular and non‐vascular major lower limb amputations. *Cochrane Database of Systematic Reviews* 2021(12). - Cox NS, Dal Corso S, Hansen H, McDonald CF, Hill CJ, Zanaboni P, Alison JA, O'Halloran P, Macdonald H, Holland AE: Telerehabilitation for chronic respiratory disease. *Cochrane Database of Systematic Reviews* 2021(1). - Correa-Agudelo E, Kim H-Y, Musuka GN, Mukandavire Z, Miller FD, Tanser F, Cuadros DF: The epidemiological landscape of anemia in women of reproductive age in sub-Saharan Africa. *Scientific reports* 2021, 11(1):11955. - Conley MM, McFarlane CM, Johnson DW, Kelly JT, Campbell KL, MacLaughlin HL: Interventions for weight loss in people with chronic kidney disease who are overweight or obese. *Cochrane Database of Systematic Reviews* 2021(3). - Clarke MJ, Broderick C, Hopewell S, Juszczak E, Eisinga A: Compression stockings for preventing deep vein thrombosis in airline passengers. *Cochrane Database of Systematic Reviews* 2021(4). - Cifuentes LI, Gattini D, Torres-Robles R, Gana JC: Beta‐blockers versus placebo or no intervention for primary prophylaxis of oesophageal variceal bleeding in children with chronic liver disease or portal vein thrombosis. *Cochrane Database of Systematic Reviews* 2021(1). - Ciapponi A, Fernandez Nievas SE, Seijo M, Rodríguez MB, Vietto V, García-Perdomo HA, Virgilio S, Fajreldines AV, Tost J, Rose CJ *et al*: Reducing medication errors for adults in hospital settings. *Cochrane Database of Systematic Reviews* 2021(11). - Chong LY, Piromchai P, Sharp S, Snidvongs K, Webster KE, Philpott C, Hopkins C, Burton MJ: Biologics for chronic rhinosinusitis. *Cochrane Database of Systematic Reviews* 2021(3). - Chong LY, Head K, Webster KE, Daw J, Richmond P, Snelling T, Bhutta MF, Schilder AGM, Burton MJ, Brennan-Jones CG: Systemic antibiotics for chronic suppurative otitis media. *Cochrane Database of Systematic Reviews* 2021(2). - Chong LY, Head K, Webster KE, Daw J, Richmond P, Snelling T, Bhutta MF, Schilder AGM, Burton MJ, Brennan-Jones CG: Topical versus systemic antibiotics for chronic suppurative otitis media. *Cochrane Database of Systematic Reviews* 2021(2). - Chhabra A, Roy Chowdhury A, Prabhakar H, Subramaniam R, Arora M, Srivastava A, Kalaivani M: Paravertebral anaes thesia with or without sedation versus general anaesthesia for women undergoing breast cancer surgery. *Cochrane Database of Systematic Reviews* 2021(2). - Cheetham S, Ngo HTT, Liira J, Liira H: Education and training for preventing sharps injuries and splash exposures in healthcare workers. *Cochrane Database of Systematic Reviews* 2021(4). - Charamba B, Liew A, Coen E, Newell J, O’Brien T, Wijns W, Simpkin AJ: Modelling the relationship between continuously measured glucose and electrocardiographic data in adults with type 1 diabetes mellitus. *Endocrinology, diabetes & metabolism* 2021, 4(3):e00263-n/a. - Chang YK, Fan HC, Hsu CC, Lim PS: The association between EKG abnormalities and the development of microalbuminuria in type 2 diabetes. *Medicine (Baltimore)* 2021, 100(51):e28018. - Chan CCH, Fage BA, Burton JK, Smailagic N, Gill SS, Herrmann N, Nikolaou V, Quinn TJ, Noel-Storr AH, Seitz DP: Mini‐Cog for the detection of dementia within a secondary care setting. *Cochrane Database of Systematic Reviews* 2021(7). - Cham KM, Abel LA, Busija L, Kowal L, Bachar Zipori A, Downie LE: Surgical interventions for infantile nystagmus syndrome. *Cochrane Database of Systematic Reviews* 2021(2). - Carson JL, Stanworth SJ, Dennis JA, Trivella M, Roubinian N, Fergusson DA, Triulzi D, Dorée C, Hébert PC: Transfusion thresholds for guiding red blood cell transfusion. *Cochrane Database of Systematic Reviews* 2021(12). - Carroll W, Green J, Gilchrist FJ: Interventions for preventing distal intestinal obstruction syndrome (DIOS) in cystic fibrosis. *Cochrane Database of Systematic Reviews* 2021(12). - Caobelli F, Haaf P, Haenny G, Pfisterer M, Zellweger MJ, Investigators B, on behalf of the BI: Prognostic value of myocardial perfusion scintigraphy in asymptomatic patients with diabetes mellitus at high cardiovascular risk: 5-year follow-up of the prospective multicenter BARDOT trial. *European journal of nuclear medicine and molecular imaging* 2021, 48(11):3512-3521. - Cao L, Li M, Yao L, Yan P, Wang X, Yang Z, Lao Y, Li H, Yang K, Li K: Siponimod for multiple sclerosis. *Cochrane Database of Systematic Reviews* 2021(11). - Cândido RCF, Menezes de Padua CA, Golder S, Junqueira DR: Immediate‐release methylphenidate for attention deficit hyperactivity disorder (ADHD) in adults. *Cochrane Database of Systematic Reviews* 2021(1). - Camargo C, Xia J, Costa CS, Gemperli R, Tatini MDC, Bulsara MK, Riera R: Botulinum toxin type A for facial wrinkles. *Cochrane Database of Systematic Reviews* 2021(7). - Busch N, Jensen MT, Goetze JP, Schou M, Biering-Sørensen T, Fritz-Hansen T, Andersen HU, Vilsbøll T, Rossing P, Jørgensen PG: Prognostic and comparative performance of cardiovascular risk markers in patients with type 2 diabetes. *J Diabetes* 2021, 13(9):754-763. - Burton JK, Stott DJ, McShane R, Noel-Storr AH, Swann-Price RS, Quinn TJ: Informant Questionnaire on Cognitive Decline in the Elderly (IQCODE) for the early detection of dementia across a variety of healthcare settings. *Cochrane Database of Systematic Reviews* 2021(7). - Burton JK, Fearon P, Noel-Storr AH, McShane R, Stott DJ, Quinn TJ: Informant Questionnaire on Cognitive Decline in the Elderly (IQCODE) for the detection of dementia within a secondary care setting. *Cochrane Database of Systematic Reviews* 2021(7). - Burton JK, Fearon P, Noel-Storr AH, McShane R, Stott DJ, Quinn TJ: Informant Questionnaire on Cognitive Decline in the Elderly (IQCODE) for the detection of dementia within a general practice (primary care) setting. *Cochrane Database of Systematic Reviews* 2021(7). - Burton JK, Craig L, Yong SQ, Siddiqi N, Teale EA, Woodhouse R, Barugh AJ, Shepherd AM, Brunton A, Freeman SC *et al*: Non‐pharmacological interventions for preventing delirium in hospitalised non‐ICU patients. *Cochrane Database of Systematic Reviews* 2021(11). - Brown T, Forster RB, Cleanthis M, Mikhailidis DP, Stansby G, Stewart M: Cilostazol for intermittent claudication. *Cochrane Database of Systematic Reviews* 2021(6). - Brown T, Cruickshank S, Noblet M: Specialist breast care nurses for support of women with breast cancer. *Cochrane Database of Systematic Reviews* 2021(2). - Brown JV, Wilson CA, Ayre K, Robertson L, South E, Molyneaux E, Trevillion K, Howard LM, Khalifeh H: Antidepressant treatment for postnatal depression. *Cochrane Database of Systematic Reviews* 2021(2). - Broderick C, Watson L, Armon MP: Thrombolytic strategies versus standard anticoagulation for acute deep vein thrombosis of the lower limb. *Cochrane Database of Systematic Reviews* 2021(1). - Broderick C, Patel JV: Infusion techniques for peripheral arterial thrombolysis. *Cochrane Database of Systematic Reviews* 2021(11). - Brigo F, Igwe SC, Lattanzi S: Ethosuximide, sodium valproate or lamotrigine for absence seizures in children and adolescents. *Cochrane Database of Systematic Reviews* 2021(1). - Bradt J, Dileo C, Myers-Coffman K, Biondo J: Music interventions for improving psychological and physical outcomes in people with cancer. *Cochrane Database of Systematic Reviews* 2021(10). - Botelho FE, Cacione DG, Leite JO, Baptista-Silva JCC: Endoluminal interventions versus surgical interventions for stenosis in vein grafts following infrainguinal bypass. *Cochrane Database of Systematic Reviews* 2021(4). - Bofill Rodriguez M, Lethaby A, Fergusson RJ: Endometrial resection and ablation versus hysterectomy for heavy menstrual bleeding. *Cochrane Database of Systematic Reviews* 2021(2). - Boaden E, Burnell J, Hives L, Dey P, Clegg A, Lyons MW, Lightbody CE, Hurley MA, Roddam H, McInnes E *et al*: Screening for aspiration risk associated with dysphagia in acute stroke. *Cochrane Database of Systematic Reviews* 2021(10). - Blazek EV, East CE, Jauncey-Cooke J, Bogossian F, Grant CA, Hough J: Lung recruitment manoeuvres for reducing mortality and respiratory morbidity in mechanically ventilated neonates. *Cochrane Database of Systematic Reviews* 2021(3). - Bedane DA, Tadesse S, Bariso M, Reta W, Desu G: Assessment of electrocardiogram abnormality and associated factors among apparently healthy adult type 2 diabetic patients on follow-up at Jimma Medical Center, Southwest Ethiopia: Cross-sectional study. *BMC Cardiovascular Disorders* 2021, 21(1):312. - Bedane DA, Tadesse S, Bariso M, Reta W, Desu G: Assessment of electrocardiogram abnormality and associated factors among apparently healthy adult type 2 diabetic patients on follow-up at Jimma Medical Center, Southwest Ethiopia: Cross-sectional study. *BMC cardiovascular disorders* 2021, 21(1):1-312. - Bedane DA, Tadesse S, Bariso M, Reta W, Desu G: Assessment of electrocardiogram abnormality and associated factors among apparently healthy adult type 2 diabetic patients on follow-up at Jimma Medical Center, Southwest Ethiopia: Cross-sectional study. *BMC Cardiovasc Disord* 2021, 21(1):312. - Bedane DA, Tadesse S, Bariso M, Reta W, Desu G: Assessment of electrocardiogram abnormality and associated factors among apparently healthy adult type 2 diabetic patients on follow-up at Jimma Medical Center, Southwest Ethiopia: Cross-sectional study. *BMC Cardiovascular Disorders* 2021, 21(1):312. - Bedane DA, Tadesse S, Bariso M, Reta W, Desu G: Assessment of electrocardiogram abnormality and associated factors among apparently healthy adult type 2 diabetic patients on follow-up at Jimma Medical Center, Southwest Ethiopia: Cross-sectional study. *BMC cardiovascular disorders* 2021, 21(1):312-312. - Bawazeer GA, Alkofide HA, Alsharafi AA, Babakr NO, Altorkistani AM, Kashour TS, Miligkos M, AlFaleh KM, Al-Ansary LA: Interrupted versus uninterrupted anticoagulation therapy for catheter ablation in adults with arrhythmias. *Cochrane Database of Systematic Reviews* 2021(10). - Baldwin C, de van der Schueren MAE, Kruizenga HM, Weekes CE: Dietary advice with or without oral nutritional supplements for disease‐related malnutrition in adults. *Cochrane Database of Systematic Reviews* 2021(12). - Babar RK, Bresnahan R, Gillespie CS, Michael BD: Lacosamide add‐on therapy for focal epilepsy. *Cochrane Database of Systematic Reviews* 2021(5). - Aye SZ, Ni H, Sein HH, Mon ST, Zheng Q, Wong YK: The effectiveness and adverse effects of D‐cycloserine compared with placebo on social and communication skills in individuals with autism spectrum disorder. *Cochrane Database of Systematic Reviews* 2021(2). - Aue-aungkul A, Kietpeerakool C, Rattanakanokchai S, Galaal K, Temtanakitpaisan T, Ngamjarus C, Lumbiganon P: Postoperative interventions for preventing bladder dysfunction after radical hysterectomy in women with early‐stage cervical cancer. *Cochrane Database of Systematic Reviews* 2021(1). - Atieh MA, Alsabeeha NHM, Payne AGT, Ali S, Faggion CM, Jr., Esposito M: Interventions for replacing missing teeth: alveolar ridge preservation techniques for dental implant site development. *Cochrane Database of Systematic Reviews* 2021(4). - Atakpa EC, Thorat MA, Cuzick J, Brentnall AR: Mammographic density, endocrine therapy and breast cancer risk: a prognostic and predictive biomarker review. *Cochrane Database of Systematic Reviews* 2021(10). - Assante R, Mainolfi CG, Zampella E, Gaudieri V, Nappi C, Mannarino T, D’Antonio A, Arumugam P, Petretta M, Cuocolo A *et al*: Relation between myocardial blood flow and cardiac events in diabetic patients with suspected coronary artery disease and normal myocardial perfusion imaging. *Journal of nuclear cardiology* 2021, 28(4):1222-1233. - Arevalo-Rodriguez I, Smailagic N, Roqué-Figuls M, Ciapponi A, Sanchez-Perez E, Giannakou A, Pedraza OL, Bonfill Cosp X, Cullum S: Mini‐Mental State Examination (MMSE) for the early detection of dementia in people with mild cognitive impairment (MCI). *Cochrane Database of Systematic Reviews* 2021(7). - Anton-Vazquez V, Hine P, Krishna S, Chaplin M, Planche T: Rapid versus standard antimicrobial susceptibility testing to guide treatment of bloodstream infection. *Cochrane Database of Systematic Reviews* 2021(5). - Andreas M, Piechotta V, Skoetz N, Grummich K, Becker M, Joos L, Becker G, Meissner W, Boehlke C: Interventions for palliative symptom control in COVID‐19 patients. *Cochrane Database of Systematic Reviews* 2021(8). - Andersson AE, Linderholm B, Giglio D: Delta NT-proBNP predicts cardiotoxicity in HER2-positive breast cancer patients treated with trastuzumab. *Acta Oncol* 2021, 60(4):475-481. - Ammenwerth E, Neyer S, Hörbst A, Mueller G, Siebert U, Schnell-Inderst P: Adult patient access to electronic health records. *Cochrane Database of Systematic Reviews* 2021(2). - Allaf M, Elghazaly H, Mohamed OG, Fareen MF, Zaman S, Salmasi AM, Tsilidis K, Dehghan A: Intermittent fasting for the prevention of cardiovascular disease. *Cochrane Database of Systematic Reviews* 2021(1). - Alhibaly HA, Al-Jameel HH, Bdair BWH, Algraittee SJR: Evaluation of Clinically Significant Cardiac Abnormalities in Patients with Normal Electrocardiogram using Transthoracic Electrocardiography. *Acta Medica Iranica* 2021:97-107. - Agarwal S, Glenton C, Tamrat T, Henschke N, Maayan N, Fønhus MS, Mehl GL, Lewin S: Decision‐support tools via mobile devices to improve quality of care in primary healthcare settings. *Cochrane Database of Systematic Reviews* 2021(7). - Abdel-Latif ME, Davis PG, Wheeler KI, De Paoli AG, Dargaville PA: Surfactant therapy via thin catheter in preterm infants with or at risk of respiratory distress syndrome. *Cochrane Database of Systematic Reviews* 2021(5). - Abdallah KS, Gadalla MA, Breijer MC, Mol BWJ: Uterine distension media for outpatient hysteroscopy. *Cochrane Database of Systematic Reviews* 2021(11). - Abbasi GA, Jagaveeran M, Goh Y-N, Tariq B: The impact of type of content use on smartphone addiction and academic performance: Physical activity as moderator. *Technology in Society* 2021, 64:101521. - Abbasi GA, Jagaveeran M, Goh Y-N, Tariq B: The impact of type of content use on smartphone addiction and academic performance: Physical activity as moderator. *Technology in Society* 2021, 64:101521. - Zhao T, Wu X, Zhang Q, Li C, Worthington HV, Hua F: Oral hygiene care for critically ill patients to prevent ventilator‐associated pneumonia. *Cochrane Database of Systematic Reviews* 2020(12). - Zhang P, Sun X, Jin H, Zhang F-L, Guo Z-N, Yang Y: Association between obesity type and common vascular and metabolic diseases: a cross-sectional study. *Frontiers in Endocrinology* 2020, 10:900. - Yu L, Ye X, Yang Z, Yang W, Zhang B, Diabetes CN, Group MDS: Prevalences and associated factors of electrocardiographic abnormalities in Chinese adults: a cross-sectional study. *BMC Cardiovascular Disorders* 2020, 20:1-11. - Yoo HHB, Nunes‐Nogueira VS, Fortes Villas Boas PJ: Anticoagulant treatment for subsegmental pulmonary embolism. *Cochrane Database of Systematic Reviews* 2020(2). - Yeong JL, Loveman E, Colquitt JL, Royle P, Waugh N, Lois N: Visual cycle modulators versus placebo or observation for the prevention and treatment of geographic atrophy due to age‐related macular degeneration. *Cochrane Database of Systematic Reviews* 2020(12). - Yang Z, Zhang Y, Lazic Mosler E, Hu J, Li H, Zhang Y, Liu J, Zhang Q: Topical benzoyl peroxide for acne. *Cochrane Database of Systematic Reviews* 2020(3). - Willcox ML, Price J, Scott S, Nicholson BD, Stuart B, Roberts NW, Allott H, Mubangizi V, Dumont A, Harnden A: Death audits and reviews for reducing maternal, perinatal and child mortality. *Cochrane Database of Systematic Reviews* 2020(3). - White SK, Schmidt RL, Walker BS, Hanson KE: (1→3)‐β‐D‐glucan testing for the detection of invasive fungal infections in immunocompromised or critically ill people. *Cochrane Database of Systematic Reviews* 2020(7). - Whear R, Thompson‐Coon J, Rogers M, Abbott RA, Anderson L, Ukoumunne O, Matthews J, Goodwin VA, Briscoe S, Perry M *et al*: Patient‐initiated appointment systems for adults with chronic conditions in secondary care. *Cochrane Database of Systematic Reviews* 2020(4). - Weibel S, Rücker G, Eberhart LHJ, Pace NL, Hartl HM, Jordan OL, Mayer D, Riemer M, Schaefer MS, Raj D *et al*: Drugs for preventing postoperative nausea and vomiting in adults after general anaesthesia: a network meta‐analysis. *Cochrane Database of Systematic Reviews* 2020(10). - Watson H, Stackhouse C: Omega‐3 fatty acid supplementation for cystic fibrosis. *Cochrane Database of Systematic Reviews* 2020(4). - Waters V, Ratjen F: Antibiotic treatment for nontuberculous mycobacteria lung infection in people with cystic fibrosis. *Cochrane Database of Systematic Reviews* 2020(6). - Wardle BG, Ambler GK, Radwan RW, Hinchliffe RJ, Twine CP: Atherectomy for peripheral arterial disease. *Cochrane Database of Systematic Reviews* 2020(9). - Wang GM, Li LJ, Tang WL, Wright JM: Renin inhibitors versus angiotensin converting enzyme (ACE) inhibitors for primary hypertension. *Cochrane Database of Systematic Reviews* 2020(10). - Walters GD, Willis NS, Cooper TE, Craig JC: Interventions for renal vasculitis in adults. *Cochrane Database of Systematic Reviews* 2020(1). - Viswanathan M, Kahwati L, Jahn B, Giger K, Dobrescu AI, Hill C, Klerings I, Meixner J, Persad E, Teufer B *et al*: Universal screening for SARS‐CoV‐2 infection: a rapid review. *Cochrane Database of Systematic Reviews* 2020(9). - Vernooij RWM, Lancee M, Cleves A, Dahm P, Bangma CH, Aben KKH: Radical prostatectomy versus deferred treatment for localised prostate cancer. *Cochrane Database of Systematic Reviews* 2020(6). - Verbeek JH, Rajamaki B, Ijaz S, Sauni R, Toomey E, Blackwood B, Tikka C, Ruotsalainen JH, Kilinc Balci FS: Personal protective equipment for preventing highly infectious diseases due to exposure to contaminated body fluids in healthcare staff. *Cochrane Database of Systematic Reviews* 2020(5). - Verbeek HHG, de Groot JWB, Sluiter WJ, Muller Kobold AC, van den Heuvel ER, Plukker JTM, Links TP: Calcitonin testing for detection of medullary thyroid cancer in people with thyroid nodules. *Cochrane Database of Systematic Reviews* 2020(3). - Vasconcellos VF, Marta GN, da Silva EMK, Gois AFT, de Castria TB, Riera R: Cisplatin versus carboplatin in combination with third‐generation drugs for advanced non‐small cell lung cancer. *Cochrane Database of Systematic Reviews* 2020(1). - van Nispen RMA, Virgili G, Hoeben M, Langelaan M, Klevering J, Keunen JEE, van Rens G: Low vision rehabilitation for better quality of life in visually impaired adults. *Cochrane Database of Systematic Reviews* 2020(1). - van Deuren S, Boonstra A, van Dulmen‐den Broeder E, Blijlevens N, Knoop H, Loonen J: Severe fatigue after treatment for childhood cancer. *Cochrane Database of Systematic Reviews* 2020(3). - Vallejo M, Reyes PPA, Martinez Garcia M, Gonzalez Garay AG: Trypanocidal drugs for late‐stage, symptomatic Chagas disease (Trypanosoma cruzi infection). *Cochrane Database of Systematic Reviews* 2020(12). - Valipour A, Jäger M, Wu P, Schmitt J, Bunch C, Weberschock T: Interventions for mycosis fungoides. *Cochrane Database of Systematic Reviews* 2020(7). - Uphoff E, Pires M, Barbui C, Barua D, Churchill R, Cristofalo D, Ekers D, Fottrell E, Mazumdar P, Purgato M *et al*: Behavioural activation therapy for depression in adults with non‐communicable diseases. *Cochrane Database of Systematic Reviews* 2020(8). - Ulug P, Powell JT, Martinez MM, Ballard DJ, Filardo G: Surgery for small asymptomatic abdominal aortic aneurysms. *Cochrane Database of Systematic Reviews* 2020(7). - Uhlig K, Efremov L, Tongers J, Frantz S, Mikolajczyk R, Sedding D, Schumann J: Inotropic agents and vasodilator strategies for the treatment of cardiogenic shock or low cardiac output syndrome. *Cochrane Database of Systematic Reviews* 2020(11). - Tully A, Smyth S, Conway Y, Geddes J, Devane D, Kelly JP, Jordan F: Interventions for the management of obesity in people with bipolar disorder. *Cochrane Database of Systematic Reviews* 2020(7). - Tsujimoto H, Tsujimoto Y, Nakata Y, Fujii T, Takahashi S, Akazawa M, Kataoka Y: Pharmacological interventions for preventing clotting of extracorporeal circuits during continuous renal replacement therapy. *Cochrane Database of Systematic Reviews* 2020(12). - Townsend CM, Nguyen TM, Cepek J, Abbass M, Parker CE, MacDonald JK, Khanna R, Jairath V, Feagan BG: Adalimumab for maintenance of remission in Crohn's disease. *Cochrane Database of Systematic Reviews* 2020(5). - Tomita Y, Moldovan M, Chang Lee R, Hsieh AHC, Townsend A, Price T: Salvage systemic therapy for advanced gastric and oesophago‐gastric junction adenocarcinoma. *Cochrane Database of Systematic Reviews* 2020(11). - Thomson J, Hogan S, Leonardi-Bee J, Williams HC, Bath-Hextall FJ: Interventions for basal cell carcinoma of the skin. *Cochrane Database of Systematic Reviews* 2020(11). - Teshale AB, Tesema GA, Worku MG, Yeshaw Y, Tessema ZT: Anemia and its associated factors among women of reproductive age in eastern Africa: A multilevel mixed-effects generalized linear model. *Plos one* 2020, 15(9):e0238957. - Tan ML, Abrams SA, Osborn DA: Vitamin D supplementation for term breastfed infants to prevent vitamin D deficiency and improve bone health. *Cochrane Database of Systematic Reviews* 2020(12). - Swierz MJ, Storman D, Riemsma RP, Wolff R, Mitus JW, Pedziwiatr M, Kleijnen J, Bala MM: Transarterial (chemo)embolisation versus no intervention or placebo for liver metastases. *Cochrane Database of Systematic Reviews* 2020(3). - Swierz MJ, Storman D, Riemsma RP, Wolff R, Mitus JW, Pedziwiatr M, Kleijnen J, Bala MM: Percutaneous ethanol injection for liver metastases. *Cochrane Database of Systematic Reviews* 2020(2). - Surace SJ, Deitch J, Johnston RV, Buchbinder R: Shock wave therapy for rotator cuff disease with or without calcification. *Cochrane Database of Systematic Reviews* 2020(3). - Storebø OJ, Stoffers-Winterling JM, Völlm BA, Kongerslev MT, Mattivi JT, Jørgensen MS, Faltinsen E, Todorovac A, Sales CP, Callesen HE *et al*: Psychological therapies for people with borderline personality disorder. *Cochrane Database of Systematic Reviews* 2020(5). - Stegeman I, Ochodo EA, Guleid F, Holtman GA, Yang B, Davenport C, Deeks JJ, Dinnes J, Dittrich S, Emperador D *et al*: Routine laboratory testing to determine if a patient has COVID‐19. *Cochrane Database of Systematic Reviews* 2020(11). - Spelten E, Thomas B, O'Meara PF, Maguire BJ, FitzGerald D, Begg SJ: Organisational interventions for preventing and minimising aggression directed towards healthcare workers by patients and patient advocates. *Cochrane Database of Systematic Reviews* 2020(4). - Sousa L, Baptista-Silva JCC, Vasconcelos V, Flumignan RLG, Nakano LCU: Internal iliac artery revascularisation versus internal iliac artery occlusion for endovascular treatment of aorto‐iliac aneurysms. *Cochrane Database of Systematic Reviews* 2020(7). - Song L, Liu F, Liu Y, Zhang R, Ji H, Jia Y: Clonazepam add‐on therapy for drug‐resistant epilepsy. *Cochrane Database of Systematic Reviews* 2020(4). - Smith V, Devane D, Nichol A, Roche D: Care bundles for improving outcomes in patients with COVID‐19 or related conditions in intensive care – a rapid scoping review. *Cochrane Database of Systematic Reviews* 2020(12). - Smith TO, Gilbert AW, Sreekanta A, Sahota O, Griffin XL, Cross JL, Fox C, Lamb SE: Enhanced rehabilitation and care models for adults with dementia following hip fracture surgery. *Cochrane Database of Systematic Reviews* 2020(2). - Siu JTP, Nguyen T, Turgeon RD: N‐acetylcysteine for non‐paracetamol (acetaminophen)‐related acute liver failure. *Cochrane Database of Systematic Reviews* 2020(12). - Simonetti RG, Perricone G, Robbins HL, Battula NR, Weickert MO, Sutton R, Khan S: Portosystemic shunts versus endoscopic intervention with or without medical treatment for prevention of rebleeding in people with cirrhosis. *Cochrane Database of Systematic Reviews* 2020(10). - Silva S, Borges L, Santiago L, Lucena L, Lindquist AR, Ribeiro T: Motor imagery for gait rehabilitation after stroke. *Cochrane Database of Systematic Reviews* 2020(9). - Shimizu W, Kubota Y, Hoshika Y, Mozawa K, Tara S, Tokita Y, Yodogawa K, Iwasaki Y-k, Yamamoto T, Takano H: Effects of empagliflozin versus placebo on cardiac sympathetic activity in acute myocardial infarction patients with type 2 diabetes mellitus: the EMBODY trial. *Cardiovascular diabetology* 2020, 19:1-12. - Shi R, Niu Z, Wu B, Hu F: Study on the risk factors for hyperuricaemia and related vascular complications in patients with type 2 diabetes mellitus. *Risk Management and Healthcare Policy* 2020:1661-1675. - Shi R, Niu Z, Wu B, Hu F: Study on the Risk Factors for Hyperuricaemia and Related Vascular Complications in Patients with Type 2 Diabetes Mellitus. *Risk management and healthcare policy* 2020, 13:1661-1675. - Shi R, Niu Z, Wu B, Hu F: Study on the Risk Factors for Hyperuricaemia and Related Vascular Complications in Patients with Type 2 Diabetes Mellitus. *Risk Management and Healthcare Policy* 2020, 13:1661. - Shaikh KJ, Osio VA, Leeflang MMG, Shaikh N: Procalcitonin, C‐reactive protein, and erythrocyte sedimentation rate for the diagnosis of acute pyelonephritis in children. *Cochrane Database of Systematic Reviews* 2020(9). - Shahrestanaki E, Maajani K, Safarpour M, Ghahremanlou HH, Tiyuri A, Sahebkar M: The relationship between smartphone addiction and quality of life among students at Tehran University of medical sciences. *Addicta: The Turkish Journal on Addictions* 2020, 7(1):23-32. - Shahrestanaki E, Maajani K, Safarpour M, Ghahremanlou HH, Tiyuri A, Sahebkar M: The relationship between smartphone addiction and quality of life among students at Tehran University of medical sciences. *Addicta: The Turkish Journal on Addictions* 2020, 7(1):61-66. - Seyed Ahmadi S, Svensson A-M, Pivodic A, Rosengren A, Lind M: Risk of atrial fibrillation in persons with type 2 diabetes and the excess risk in relation to glycaemic control and renal function: a Swedish cohort study. *Cardiovascular diabetology* 2020, 19:1-12. - Semlitsch T, Engler J, Siebenhofer A, Jeitler K, Berghold A, Horvath K: (Ultra‐)long‐acting insulin analogues versus NPH insulin (human isophane insulin) for adults with type 2 diabetes mellitus. *Cochrane Database of Systematic Reviews* 2020(11). - Schwartz SG, Flynn Jr HW, Wang X, Kuriyan AE, Abariga SA, Lee WH: Tamponade in surgery for retinal detachment associated with proliferative vitreoretinopathy. *Cochrane Database of Systematic Reviews* 2020(5). - Schmidt AF, Carter JP, Pearce LS, Wilkins JT, Overington JP, Hingorani AD, Casas J: PCSK9 monoclonal antibodies for the primary and secondary prevention of cardiovascular disease. *Cochrane Database of Systematic Reviews* 2020(10). - Sawangjit R, Dilokthornsakul P, Lloyd-Lavery A, Lai NM, Dellavalle R, Chaiyakunapruk N: Systemic treatments for eczema: a network meta‐analysis. *Cochrane Database of Systematic Reviews* 2020(9). - Savla K, Le JT, Pucker AD: Tea tree oil for Demodex blepharitis. *Cochrane Database of Systematic Reviews* 2020(6). - Saunders DH, Sanderson M, Hayes S, Johnson L, Kramer S, Carter DD, Jarvis H, Brazzelli M, Mead GE: Physical fitness training for stroke patients. *Cochrane Database of Systematic Reviews* 2020(3). - Santino TA, Chaves GSS, Freitas DA, Fregonezi GAF, Mendonça K: Breathing exercises for adults with asthma. *Cochrane Database of Systematic Reviews* 2020(3). - Rutjes AWS, Porreca E, Candeloro M, Valeriani E, Di Nisio M: Primary prophylaxis for venous thromboembolism in ambulatory cancer patients receiving chemotherapy. *Cochrane Database of Systematic Reviews* 2020(12). - Rueda JR, Mugueta-Aguinaga I, Vilaró J, Rueda-Etxebarria M: Myofunctional therapy (oropharyngeal exercises) for obstructive sleep apnoea. *Cochrane Database of Systematic Reviews* 2020(11). - Rosenberg JE, Jung JH, Edgerton Z, Lee H, Lee S, Bakker CJ, Dahm P: Retzius‐sparing versus standard robotic‐assisted laparoscopic prostatectomy for the treatment of clinically localized prostate cancer. *Cochrane Database of Systematic Reviews* 2020(8). - Romantsik O, Calevo MG, Bruschettini M: Head midline position for preventing the occurrence or extension of germinal matrix‐intraventricular haemorrhage in preterm infants. *Cochrane Database of Systematic Reviews* 2020(7). - Rohwer AC, Oladapo OT, Hofmeyr GJ: Strategies for optimising antenatal corticosteroid administration for women with anticipated preterm birth. *Cochrane Database of Systematic Reviews* 2020(5). - Robinson J, Hartling L, Vandermeer B, Sebastianski M, Klassen TP: Intravenous immunoglobulin for presumed viral myocarditis in children and adults. *Cochrane Database of Systematic Reviews* 2020(8). - Rittiphairoj T, Mir TA, Li T, Virgili G: Intravitreal steroids for macular edema in diabetes. *Cochrane Database of Systematic Reviews* 2020(11). - Rerkasem A, Orrapin S, Howard DPJ, Rerkasem K: Carotid endarterectomy for symptomatic carotid stenosis. *Cochrane Database of Systematic Reviews* 2020(9). - Ream E, Hughes AE, Cox A, Skarparis K, Richardson A, Pedersen VH, Wiseman T, Forbes A, Bryant A: Telephone interventions for symptom management in adults with cancer. *Cochrane Database of Systematic Reviews* 2020(6). - Ravichandran S, Srivastav S, Haridas Kamble P, Shukla R, Sharma P, Sharma R: Effect of Vitamin D status on QTc interval in type 2 diabetes mellitus. *J Basic Clin Physiol Pharmacol* 2020, 32(3):163-167. - Qutishat M, Lazarus ER, Razmy AM, Packianathan S: University students’ nomophobia prevalence, sociodemographic factors and relationship with academic performance at a University in Oman. *International Journal of Africa Nursing Sciences* 2020, 13:100206. - Puntis S, Minichino A, De Crescenzo F, Harrison R, Cipriani A, Lennox B: Specialised early intervention teams (extended time) for recent‐onset psychosis. *Cochrane Database of Systematic Reviews* 2020(11). - Puntis S, Minichino A, De Crescenzo F, Harrison R, Cipriani A, Lennox B: Specialised early intervention teams for recent‐onset psychosis. *Cochrane Database of Systematic Reviews* 2020(11). - Pruimboom T, Schols RM, Van Kuijk SMJ, Van der Hulst R, Qiu SS: Indocyanine green angiography for preventing postoperative mastectomy skin flap necrosis in immediate breast reconstruction. *Cochrane Database of Systematic Reviews* 2020(4). - Pollock A, Campbell P, Cheyne J, Cowie J, Davis B, McCallum J, McGill K, Elders A, Hagen S, McClurg D *et al*: Interventions to support the resilience and mental health of frontline health and social care professionals during and after a disease outbreak, epidemic or pandemic: a mixed methods systematic review. *Cochrane Database of Systematic Reviews* 2020(11). - Phelps PO, Abariga SA, Cowling BJ, Selva D, Marcet MM: Antimetabolites as an adjunct to dacryocystorhinostomy for nasolacrimal duct obstruction. *Cochrane Database of Systematic Reviews* 2020(4). - Pelland-Marcotte MC, Amiri N, Avila ML, Brandão LR: Low molecular weight heparin for prevention of central venous catheter‐related thrombosis in children. *Cochrane Database of Systematic Reviews* 2020(6). - Peinemann F, Harari M, Peternel S, Chan T, Chan D, Labeit AM, Gambichler T: Indoor salt water baths followed by artificial ultraviolet B light for chronic plaque psoriasis. *Cochrane Database of Systematic Reviews* 2020(5). - Peer N, Balakrishna Y, Durao S: Screening for type 2 diabetes mellitus. *Cochrane Database of Systematic Reviews* 2020(5). - Parry Smith WR, Papadopoulou A, Thomas E, Tobias A, Price MJ, Meher S, Alfirevic Z, Weeks AD, Hofmeyr GJ, Gülmezoglu AM *et al*: Uterotonic agents for first‐line treatment of postpartum haemorrhage: a network meta‐analysis. *Cochrane Database of Systematic Reviews* 2020(11). - Parks NE, Jackson-Tarlton CS, Vacchi L, Merdad R, Johnston BC: Dietary interventions for multiple sclerosis‐related outcomes. *Cochrane Database of Systematic Reviews* 2020(5). - Papola D, Purgato M, Gastaldon C, Bovo C, van Ommeren M, Barbui C, Tol WA: Psychological and social interventions for the prevention of mental disorders in people living in low‐ and middle‐income countries affected by humanitarian crises. *Cochrane Database of Systematic Reviews* 2020(9). - Panebianco M, Prabhakar H, Marson AG: Rufinamide add‐on therapy for drug‐resistant epilepsy. *Cochrane Database of Systematic Reviews* 2020(11). - Pandey A, Patel KV, Bahnson JL, Gaussoin SA, Martin CK, Balasubramanyam A, Johnson KC, McGuire DK, Bertoni AG, Kitzman D: Association of intensive lifestyle intervention, fitness, and body mass index with risk of heart failure in overweight or obese adults with type 2 diabetes mellitus: an analysis from the Look AHEAD trial. *Circulation* 2020, 141(16):1295-1306. - Palmer J, Pymer S, Smith GE, Harwood AE, Ingle L, Huang C, Chetter IC: Presurgery exercise‐based conditioning interventions (prehabilitation) in adults undergoing lower limb surgery for peripheral arterial disease. *Cochrane Database of Systematic Reviews* 2020(9). - Palareti L, Melotti G, Cassis F, Nevitt SJ, Iorio A: Psychological interventions for people with hemophilia. *Cochrane Database of Systematic Reviews* 2020(3). - Palaniappan SK, Than NN, Thein AW, van Mourik I: Interventions for preventing and managing advanced liver disease in cystic fibrosis. *Cochrane Database of Systematic Reviews* 2020(3). - Pahl A, Young L, Buus-Frank ME, Marcellus L, Soll R: Non‐pharmacological care for opioid withdrawal in newborns. *Cochrane Database of Systematic Reviews* 2020(12). - Packer M: Do most patients with obesity or type 2 diabetes, and atrial fibrillation, also have undiagnosed heart failure? A critical conceptual framework for understanding mechanisms and improving diagnosis and treatment. *European journal of heart failure* 2020, 22(2):214-227. - Oswal RM, Pal S, Patel SV, Patel A, Doshi V, Gandhi RR: Smartphone addiction among undergraduate medical students and its association with academic performance. *Open journal of psychiatry & allied sciences* 2020, 11(2):111-116. - Orelio CC, van Hessen C, Sanchez-Manuel FJ, Aufenacker TJ, Scholten R: Antibiotic prophylaxis for prevention of postoperative wound infection in adults undergoing open elective inguinal or femoral hernia repair. *Cochrane Database of Systematic Reviews* 2020(4). - Omer O: Smartphone addiction and fear of missing out: Does smartphone use matter for students’ academic performance? *Journal of Computer and Education Research* 2020, 8(15):344-355. - Oliveira VHB, Mendonça K, Monteiro KS, Silva IS, Santino TA, Nogueira P: Physical therapies for postural abnormalities in people with cystic fibrosis. *Cochrane Database of Systematic Reviews* 2020(3). - Oliveira CB, Maher CG, Ferreira ML, Hancock MJ, Oliveira VC, McLachlan AJ, Koes BW, Ferreira PH, Cohen SP, Pinto RZ: Epidural corticosteroid injections for lumbosacral radicular pain. *Cochrane Database of Systematic Reviews* 2020(4). - Ohlsson A, Shah SS: Ibuprofen for the prevention of patent ductus arteriosus in preterm and/or low birth weight infants. *Cochrane Database of Systematic Reviews* 2020(1). - Obeid G, Do G, Kirby L, Hughes C, Sbidian E, Le Cleach L: Interventions for chronic palmoplantar pustulosis. *Cochrane Database of Systematic Reviews* 2020(1). - Nishikawa Y, Hiroyama N, Fukahori H, Ota E, Mizuno A, Miyashita M, Yoneoka D, Kwong JSW: Advance care planning for adults with heart failure. *Cochrane Database of Systematic Reviews* 2020(2). - Niaz OS, Rao A, Abidia A, Parrott R, Refson J, Somaiya P: Surgical and medical interventions for abdominal aortic graft infections. *Cochrane Database of Systematic Reviews* 2020(8). - Nejstgaard CH, Bero L, Hróbjartsson A, Jørgensen AW, Jørgensen KJ, Le M, Lundh A: Conflicts of interest in clinical guidelines, advisory committee reports, opinion pieces, and narrative reviews: associations with recommendations. *Cochrane Database of Systematic Reviews* 2020(12). - Natale P, Palmer SC, Ruospo M, Saglimbene VM, Strippoli GFM: Potassium binders for chronic hyperkalaemia in people with chronic kidney disease. *Cochrane Database of Systematic Reviews* 2020(6). - Natale P, Palmer SC, Ruospo M, Saglimbene VM, Craig JC, Vecchio M, Samuels JA, Molony DA, Schena FP, Strippoli GFM: Immunosuppressive agents for treating IgA nephropathy. *Cochrane Database of Systematic Reviews* 2020(3). - Mylona M, Liatis S, Anastasiadis G, Kapelios C, Kokkinos A: Severe iatrogenic hypoglycaemia requiring medical assistance is associated with concurrent prolongation of the QTc interval. *Diabetes Res Clin Pract* 2020, 161:108038. - Murray M, Hine P: Treating progressive disseminated histoplasmosis in people living with HIV. *Cochrane Database of Systematic Reviews* 2020(4). - Murray A, Nguyen TM, Parker CE, Feagan BG, MacDonald JK: Oral 5‐aminosalicylic acid for induction of remission in ulcerative colitis. *Cochrane Database of Systematic Reviews* 2020(8). - Murray A, Nguyen TM, Parker CE, Feagan BG, MacDonald JK: Oral 5‐aminosalicylic acid for maintenance of remission in ulcerative colitis. *Cochrane Database of Systematic Reviews* 2020(8). - Mulvaney CA, Duarte GS, Handley J, Evans DJW, Menon S, Wyse R, Emsley HCA: GLP‐1 receptor agonists for Parkinson's disease. *Cochrane Database of Systematic Reviews* 2020(7). - Mulia EPB, Nugraha RA, A'Yun M Q, Juwita RR, Yofrido FM, Julario R, Alkaff FF: Electrocardiographic abnormalities among late-stage non-dialysis chronic kidney disease patients. *J Basic Clin Physiol Pharmacol* 2020, 32(3):155-162. - Mulia EPB, Nugraha RA, A'Yun M Q, Juwita RR, Yofrido FM, Julario R, Alkaff FF: Electrocardiographic abnormalities among late-stage non-dialysis chronic kidney disease patients. *J Basic Clin Physiol Pharmacol* 2020, 32(3):155-162. - Mulia EPB, Nugraha RA, A’yun MQ, Juwita RR, Yofrido FM, Julario R, Alkaff FF: Electrocardiographic abnormalities among late-stage non-dialysis chronic kidney disease patients. *Journal of basic and clinical physiology and pharmacology* 2020, 32(3):155-162. - Mugendi GA, Mutua FM, Natale P, Esterhuizen TM, Strippoli GFM: Calcium channel blockers for people with chronic kidney disease requiring dialysis. *Cochrane Database of Systematic Reviews* 2020(10). - Mu J, Furlan AD, Lam WY, Hsu MY, Ning Z, Lao L: Acupuncture for chronic nonspecific low back pain. *Cochrane Database of Systematic Reviews* 2020(12). - Morrison L, Milroy S: Oscillating devices for airway clearance in people with cystic fibrosis. *Cochrane Database of Systematic Reviews* 2020(4). - Moore ZEH, Corcoran MA, Patton D: Nutritional interventions for treating foot ulcers in people with diabetes. *Cochrane Database of Systematic Reviews* 2020(7). - Monteiro J, Tanday A, Ashley PF, Parekh S, Alamri H: Interventions for increasing acceptance of local anaesthetic in children and adolescents having dental treatment. *Cochrane Database of Systematic Reviews* 2020(2). - Möhler R, Renom A, Renom H, Meyer G: Personally tailored activities for improving psychosocial outcomes for people with dementia in community settings. *Cochrane Database of Systematic Reviews* 2020(8). - Mittermeier T, Farrant C, Wise MR: Levonorgestrel‐releasing intrauterine system for endometrial hyperplasia. *Cochrane Database of Systematic Reviews* 2020(9). - Mitra S, Scrivens A, von Kursell AM, Disher T: Early treatment versus expectant management of hemodynamically significant patent ductus arteriosus for preterm infants. *Cochrane Database of Systematic Reviews* 2020(12). - Minozzi S, Amato L, Jahanfar S, Bellisario C, Ferri M, Davoli M: Maintenance agonist treatments for opiate‐dependent pregnant women. *Cochrane Database of Systematic Reviews* 2020(11). - Minakaran N, Ezra DG, Allan BDS: Topical anaesthesia plus intracameral lidocaine versus topical anaesthesia alone for phacoemulsification cataract surgery in adults. *Cochrane Database of Systematic Reviews* 2020(7). - Milligan R, Daher A, Villanueva G, Bergman H, Graves PM: Primaquine alternative dosing schedules for preventing malaria relapse in people with Plasmodium vivax. *Cochrane Database of Systematic Reviews* 2020(8). - Michaelis R, Tang V, Nevitt SJ, Wagner JL, Modi AC, LaFrance Jr W, Goldstein LH, Gandy M, Bresnahan R, Valente K *et al*: Psychological treatments for people with epilepsy. *Cochrane Database of Systematic Reviews* 2020(8). - Mendes LA, Lima I, Souza T, do Nascimento GC, Resqueti VR, Fregonezi GAF: Motor neuroprosthesis for promoting recovery of function after stroke. *Cochrane Database of Systematic Reviews* 2020(1). - Mehrholz J, Thomas S, Kugler J, Pohl M, Elsner B: Electromechanical‐assisted training for walking after stroke. *Cochrane Database of Systematic Reviews* 2020(10). - McGoldrick E, Stewart F, Parker R, Dalziel SR: Antenatal corticosteroids for accelerating fetal lung maturation for women at risk of preterm birth. *Cochrane Database of Systematic Reviews* 2020(12). - Mbizvo GK, Chandrasekar B, Nevitt SJ, Dixon P, Hutton JL, Marson AG: Levetiracetam add‐on for drug‐resistant focal epilepsy. *Cochrane Database of Systematic Reviews* 2020(6). - Mattioni A, Cenciarelli S, Eusebi P, Brazzelli M, Mazzoli T, Del Sette M, Gandolfo C, Marinoni M, Finocchi C, Saia V *et al*: Transcranial Doppler sonography for detecting stenosis or occlusion of intracranial arteries in people with acute ischaemic stroke. *Cochrane Database of Systematic Reviews* 2020(2). - Martinez-Zapata MJ, Vernooij RWM, Simancas-Racines D, Uriona Tuma SM, Stein AT, Moreno Carriles RMM, Vargas E, Bonfill Cosp X: Phlebotonics for venous insufficiency. *Cochrane Database of Systematic Reviews* 2020(11). - Martí-Carvajal AJ, Valli C, Martí-Amarista CE, Solà I, Martí-Fàbregas J, Bonfill Cosp X: Citicoline for treating people with acute ischemic stroke. *Cochrane Database of Systematic Reviews* 2020(8). - Martí-Carvajal AJ, Dayer M, Conterno LO, Gonzalez Garay AG, Martí-Amarista CE: A comparison of different antibiotic regimens for the treatment of infective endocarditis. *Cochrane Database of Systematic Reviews* 2020(5). - Mah JY, Choy SW, Roberts MA, Desai AM, Corken M, Gwini SM, McMahon LP: Oral protein‐based supplements versus placebo or no treatment for people with chronic kidney disease requiring dialysis. *Cochrane Database of Systematic Reviews* 2020(5). - Maguire MJ, Jackson CF, Marson AG, Nevitt SJ: Treatments for the prevention of Sudden Unexpected Death in Epilepsy (SUDEP). *Cochrane Database of Systematic Reviews* 2020(4). - Magrinelli F, Fabrizi GM, Santoro L, Manganelli F, Zanette G, Cavallaro T, Tamburin S: Pharmacological treatment for familial amyloid polyneuropathy. *Cochrane Database of Systematic Reviews* 2020(4). - Madsen BK, Zetner D, Møller AM, Rosenberg J: Melatonin for preoperative and postoperative anxiety in adults. *Cochrane Database of Systematic Reviews* 2020(12). - Mackintosh NJ, Davis RE, Easter A, Rayment-Jones H, Sevdalis N, Wilson S, Adams M, Sandall J: Interventions to increase patient and family involvement in escalation of care for acute life‐threatening illness in community health and hospital settings. *Cochrane Database of Systematic Reviews* 2020(12). - Macey R, Walsh T, Riley P, Glenny AM, Worthington HV, Fee PA, Clarkson JE, Ricketts D: Fluorescence devices for the detection of dental caries. *Cochrane Database of Systematic Reviews* 2020(12). - Lunney M, Ruospo M, Natale P, Quinn RR, Ronksley PE, Konstantinidis I, Palmer SC, Tonelli M, Strippoli GFM, Ravani P: Pharmacological interventions for heart failure in people with chronic kidney disease. *Cochrane Database of Systematic Reviews* 2020(2). - Lubberding AF, Pereira L, Xue J, Gottlieb LA, Matchkov VV, Gomez AM, Thomsen MB: Aberrant sinus node firing during β‐adrenergic stimulation leads to cardiac arrhythmias in diabetic mice. *Acta Physiologica* 2020, 229(1):e13444-n/a. - Lorentzen AK, Davis C, Penninga L: Interventions for frostbite injuries. *Cochrane Database of Systematic Reviews* 2020(12). - Lord R, Jones AM, Horsley A: Antibiotic treatment for Burkholderia cepacia complex in people with cystic fibrosis experiencing a pulmonary exacerbation. *Cochrane Database of Systematic Reviews* 2020(4). - Lo Kevin B, Gul F, Ram P, Kluger Aaron Y, Tecson Kristen M, McCullough Peter A, Rangaswami J: The Effects of SGLT2 Inhibitors on Cardiovascular and Renal Outcomes in Diabetic Patients: A Systematic Review and Meta-Analysis. *Cardiorenal medicine* 2020, 10(1):1-10. - Lo C, Toyama T, Oshima M, Jun M, Chin KL, Hawley CM, Zoungas S: Glucose‐lowering agents for treating pre‐existing and new‐onset diabetes in kidney transplant recipients. *Cochrane Database of Systematic Reviews* 2020(8). - Liu H, Yu H, Xia J, Liu L, Liu GJ, Sang H, Peinemann F: Topical azelaic acid, salicylic acid, nicotinamide, sulphur, zinc and fruit acid (alpha‐hydroxy acid) for acne. *Cochrane Database of Systematic Reviews* 2020(5). - Lin PT, Wang SH, Chi CC: Low molecular weight heparin for prevention of microvascular occlusion in digital replantation. *Cochrane Database of Systematic Reviews* 2020(4). - Li Y, Ma J, Jin Y, Li N, Zheng R, Mu W, Wang J, Si JH, Chen J, Shang HC: Benzodiazepines for treatment of patients with delirium excluding those who are cared for in an intensive care unit. *Cochrane Database of Systematic Reviews* 2020(2). - Li T, Luo H-H, Feng X-F, Bai Y, Fang Z-Z, Wu G-G, Wu J-L: Plasma Free Amino Acids and Risk of Cardiovascular Disease in Chinese Patients With Type 2 Diabetes. *Frontiers in endocrinology* 2020, 11. - Lewis SR, Baker PE, Andrews PJD, Cheng A, Deol K, Hammond N, Saxena M: Interventions to reduce body temperature to 35 ⁰C to 37 ⁰C in adults and children with traumatic brain injury. *Cochrane Database of Systematic Reviews* 2020(10). - Leon N, Balakrishna Y, Hohlfeld A, Odendaal WA, Schmidt BM, Zweigenthal V, Anstey Watkins J, Daniels K: Routine Health Information System (RHIS) improvements for strengthened health system management. *Cochrane Database of Systematic Reviews* 2020(8). - Law SK, Wang L, Li T: Acupuncture for glaucoma. *Cochrane Database of Systematic Reviews* 2020(2). - Law CCY, Bell C, Koh D, Bao Y, Jairath V, Narula N: Risk of postoperative infectious complications from medical therapies in inflammatory bowel disease. *Cochrane Database of Systematic Reviews* 2020(10). - Laver KE, Adey‐Wakeling Z, Crotty M, Lannin NA, George S, Sherrington C: Telerehabilitation services for stroke. *Cochrane Database of Systematic Reviews* 2020(1). - Langhorne P, Ramachandra S: Organised inpatient (stroke unit) care for stroke: network meta‐analysis. *Cochrane Database of Systematic Reviews* 2020(4). - Kunzler AM, Helmreich I, Chmitorz A, König J, Binder H, Wessa M, Lieb K: Psychological interventions to foster resilience in healthcare professionals. *Cochrane Database of Systematic Reviews* 2020(7). - Kumbargere Nagraj S, Eachempati P, Paisi M, Nasser M, Sivaramakrishnan G, Verbeek JH: Interventions to reduce contaminated aerosols produced during dental procedures for preventing infectious diseases. *Cochrane Database of Systematic Reviews* 2020(10). - Kuhle S, Hoffmann DU, Mitra S, Urschitz MS: Anti‐inflammatory medications for obstructive sleep apnoea in children. *Cochrane Database of Systematic Reviews* 2020(1). - Kreuzberger N, Damen J, Trivella M, Estcourt LJ, Aldin A, Umlauff L, Vazquez-Montes M, Wolff R, Moons KG, Monsef I *et al*: Prognostic models for newly‐diagnosed chronic lymphocytic leukaemia in adults: a systematic review and meta‐analysis. *Cochrane Database of Systematic Reviews* 2020(7). - Kopsaftis Z, Carson‐Chahhoud KV, Austin MA, Wood‐Baker R: Oxygen therapy in the pre‐hospital setting for acute exacerbations of chronic obstructive pulmonary disease. *Cochrane Database of Systematic Reviews* 2020(1). - Kongwattanakul K, Rojanapithayakorn N, Laopaiboon M, Lumbiganon P: Anaesthesia/analgesia for manual removal of retained placenta. *Cochrane Database of Systematic Reviews* 2020(6). - Kim SE, Nowak V, Quartilho A, Larkin F, Hingorani M, Tuft S, Dahlmann-Noor A: Systemic interventions for severe atopic and vernal keratoconjunctivitis in children and young people up to the age of 16 years. *Cochrane Database of Systematic Reviews* 2020(10). - Kim HN, Lee JH, Park HS, Yang DH, Jang SY, Bae MH, Cho Y, Chae SC, Lee YH: A Case of COVID-19 with Acute Myocardial Infarction and Cardiogenic Shock. *J Korean Med Sci* 2020, 35(27):e258. - Kim HN, Lee JH, Park HS, Yang DH, Jang SY, Bae MH, Cho Y, Chae SC, Lee YH: A Case of COVID-19 with Acute Myocardial Infarction and Cardiogenic Shock. *J Korean Med Sci* 2020, 35(27):e258. - Khattri S, Kumbargere Nagraj S, Arora A, Eachempati P, Kusum CK, Bhat KG, Johnson TM, Lodi G: Adjunctive systemic antimicrobials for the non‐surgical treatment of periodontitis. *Cochrane Database of Systematic Reviews* 2020(11). - Khan L, Soliman H, Sahgal A, Perry J, Xu W, Tsao MN: External beam radiation dose escalation for high grade glioma. *Cochrane Database of Systematic Reviews* 2020(5). - Khalifa NR, Gibbon S, Völlm BA, Cheung N-Y, McCarthy L: Pharmacological interventions for antisocial personality disorder. *Cochrane Database of Systematic Reviews* 2020(9). - Kejela G, Wakgari A, Tesfaye T, Turi E, Adugna M, Alemu N, Jebessa L: Prevalence of anemia and its associated factors among pregnant women attending antenatal care follow up at Wollega University referral hospital, Western Ethiopia. *Contraception and reproductive medicine* 2020, 5:1-8. - Kaur L, Gordon M, Baines PA, Iheozor-Ejiofor Z, Sinopoulou V, Akobeng AK: Probiotics for induction of remission in ulcerative colitis. *Cochrane Database of Systematic Reviews* 2020(3). - Kaufner L, von Heymann C, Henkelmann A, Pace NL, Weibel S, Kranke P, Meerpohl JJ, Gill R: Erythropoietin plus iron versus control treatment including placebo or iron for preoperative anaemic adults undergoing non‐cardiac surgery. *Cochrane Database of Systematic Reviews* 2020(8). - Karki S, Singh JP, Paudel G, Khatiwada S, Timilsina S: How addicted are newly admitted undergraduate medical students to smartphones?: a cross-sectional study from Chitwan medical college, Nepal. *BMC psychiatry* 2020, 20:1-7. - Karantana A, Handoll HHG, Sabouni A: Percutaneous pinning for treating distal radial fractures in adults. *Cochrane Database of Systematic Reviews* 2020(2). - Jullien S, Dissanayake HA, Chaplin M: Rapid diagnostic tests for plague. *Cochrane Database of Systematic Reviews* 2020(6). - Jongsma H, Bekken J, Ayez N, Hoogewerf CJ, Van Weel V, Fioole B: Angioplasty versus stenting for iliac artery lesions. *Cochrane Database of Systematic Reviews* 2020(12). - Jindal A, Ctori I, Virgili G, Lucenteforte E, Lawrenson JG: Non‐contact tests for identifying people at risk of primary angle closure glaucoma. *Cochrane Database of Systematic Reviews* 2020(5). - Jeyaraman MM, Al‐Yousif NSH, Singh Mann A, Dolinsky VW, Rabbani R, Zarychanski R, Abou‐Setta AM: Resveratrol for adults with type 2 diabetes mellitus. *Cochrane Database of Systematic Reviews* 2020(1). - Jansen SCP, Abaraogu UO, Lauret GJ, Fakhry F, Fokkenrood HJP, Teijink JAW: Modes of exercise training for intermittent claudication. *Cochrane Database of Systematic Reviews* 2020(8). - Jakob T, Tesfamariam YM, Macherey S, Kuhr K, Adams A, Monsef I, Heidenreich A, Skoetz N: Bisphosphonates or RANK‐ligand‐inhibitors for men with prostate cancer and bone metastases: a network meta‐analysis. *Cochrane Database of Systematic Reviews* 2020(12). - Jagannath VA, Thaker V, Chang AB, Price AI: Vitamin K supplementation for cystic fibrosis. *Cochrane Database of Systematic Reviews* 2020(6). - Ipsen E, Madsen KS, Chi Y, Pedersen-Bjergaard U, Richter B, Metzendorf MI, Hemmingsen B: Pioglitazone for prevention or delay of type 2 diabetes mellitus and its associated complications in people at risk for the development of type 2 diabetes mellitus. *Cochrane Database of Systematic Reviews* 2020(11). - Imamura M, Scott NW, Wallace SA, Ogah JA, Ford AA, Dubos YA, Brazzelli M: Interventions for treating people with symptoms of bladder pain syndrome: a network meta‐analysis. *Cochrane Database of Systematic Reviews* 2020(7). - Iheozor-Ejiofor Z, Kaur L, Gordon M, Baines PA, Sinopoulou V, Akobeng AK: Probiotics for maintenance of remission in ulcerative colitis. *Cochrane Database of Systematic Reviews* 2020(3). - Iguchi M, Noguchi Y, Yamamoto S, Tanaka Y, Tsujimoto H: Diagnostic test accuracy of jolt accentuation for headache in acute meningitis in the emergency setting. *Cochrane Database of Systematic Reviews* 2020(6). - Htay H, Johnson DW, Craig JC, Teixeira-Pinto A, Hawley CM, Cho Y: Urgent‐start peritoneal dialysis versus conventional‐start peritoneal dialysis for people with chronic kidney disease. *Cochrane Database of Systematic Reviews* 2020(12). - Houghton C, Meskell P, Delaney H, Smalle M, Glenton C, Booth A, Chan XHS, Devane D, Biesty LM: Barriers and facilitators to healthcare workers’ adherence with infection prevention and control (IPC) guidelines for respiratory infectious diseases: a rapid qualitative evidence synthesis. *Cochrane Database of Systematic Reviews* 2020(4). - Houghton C, Dowling M, Meskell P, Hunter A, Gardner H, Conway A, Treweek S, Sutcliffe K, Noyes J, Devane D *et al*: Factors that impact on recruitment to randomised trials in health care: a qualitative evidence synthesis. *Cochrane Database of Systematic Reviews* 2020(10). - Hoshino N, Takada T, Hida K, Hasegawa S, Furukawa TA, Sakai Y: Daikenchuto for reducing postoperative ileus in patients undergoing elective abdominal surgery. *Cochrane Database of Systematic Reviews* 2020(3). - Hoogewerf CJ, Hop MJ, Nieuwenhuis MK, Oen I, Middelkoop E, Van Baar ME: Topical treatment for facial burns. *Cochrane Database of Systematic Reviews* 2020(7). - Hofmann F, Hwang EC, Lam TBL, Bex A, Yuan Y, Marconi LSO, Ljungberg B: Targeted therapy for metastatic renal cell carcinoma. *Cochrane Database of Systematic Reviews* 2020(10). - Hercz D, Jiang SH, Webster AC: Interventions for itch in people with advanced chronic kidney disease. *Cochrane Database of Systematic Reviews* 2020(12). - Head K, Chong LY, Bhutta MF, Morris PS, Vijayasekaran S, Burton MJ, Schilder AGM, Brennan‐Jones CG: Topical antiseptics for chronic suppurative otitis media. *Cochrane Database of Systematic Reviews* 2020(1). - Head K, Chong LY, Bhutta MF, Morris PS, Vijayasekaran S, Burton MJ, Schilder AGM, Brennan-Jones CG: Antibiotics versus topical antiseptics for chronic suppurative otitis media. *Cochrane Database of Systematic Reviews* 2020(1). - He J, Wu B, Chen Y, Tang J, Liu Q, Zhou S, Chen C, Qin Q, Huang K, Lv J: Characteristic electrocardiographic manifestations in patients with COVID-19. *Canadian Journal of Cardiology* 2020, 36(6):966. e961-966. e964. - He J, Wu B, Chen Y, Tang J, Liu Q, Zhou S, Chen C, Qin Q, Huang K, Lv J: Characteristic electrocardiographic manifestations in patients with COVID-19. *Canadian Journal of Cardiology* 2020, 36(6):966. e961-966. e964. - Hassim SR, Arifin WN, Kueh YC, Yaacob NA: Confirmatory factor analysis of the Malay version of the smartphone addiction scale among medical students in Malaysia. *International journal of environmental research and public health* 2020, 17(11):3820. - Hardman J, Sharma N, Smith J, Nankivell P: Conservative management of oesophageal soft food bolus impaction. *Cochrane Database of Systematic Reviews* 2020(5). - Hanna C, Lawrie TA, Rogozińska E, Kernohan A, Jefferies S, Bulbeck H, Ali UM, Robinson T, Grant R: Treatment of newly diagnosed glioblastoma in the elderly: a network meta‐analysis. *Cochrane Database of Systematic Reviews* 2020(3). - Hameed M, O'Doherty L, Gilchrist G, Tirado-Muñoz J, Taft A, Chondros P, Feder G, Tan M, Hegarty K: Psychological therapies for women who experience intimate partner violence. *Cochrane Database of Systematic Reviews* 2020(7). - Hahn D, Hodson EM, Fouque D: Low protein diets for non‐diabetic adults with chronic kidney disease. *Cochrane Database of Systematic Reviews* 2020(10). - Guay J, Kopp S: Peripheral nerve blocks for hip fractures in adults. *Cochrane Database of Systematic Reviews* 2020(11). - Grzegorzewska AE, Ostromecka K, Adamska P, Mostowska A, Warchoł W, Jagodziński PP: Paraoxonase 1 gene polymorphisms concerning non-insulin-dependent diabetes mellitus nephropathy in hemodialysis patients. *Journal of diabetes and its complications* 2020, 34(11):107687-107687. - Grillo‐Ardila CF, Torres M, Gaitán HG: Rapid point of care test for detecting urogenital Chlamydia trachomatis infection in nonpregnant women and men at reproductive age. *Cochrane Database of Systematic Reviews* 2020(1). - Greenhalgh J, Weston J, Dundar Y, Nevitt SJ, Marson AG: Antiepileptic drugs as prophylaxis for postcraniotomy seizures. *Cochrane Database of Systematic Reviews* 2020(4). - Grant R, Dowswell T, Tomlinson E, Brennan PM, Walter FM, Ben-Shlomo Y, Hunt D, Bulbeck H, Kernohan A, Robinson T *et al*: Interventions to reduce the time to diagnosis of brain tumours. *Cochrane Database of Systematic Reviews* 2020(9). - Grande AJ, Keogh J, Silva V, Scott AM: Exercise versus no exercise for the occurrence, severity, and duration of acute respiratory infections. *Cochrane Database of Systematic Reviews* 2020(4). - Goto S, Sakamoto T, Ganeko R, Hida K, Furukawa TA, Sakai Y: Subcuticular sutures for skin closure in non‐obstetric surgery. *Cochrane Database of Systematic Reviews* 2020(4). - Gonçalves-Bradley DC, J Maria AR, Ricci-Cabello I, Villanueva G, Fønhus MS, Glenton C, Lewin S, Henschke N, Buckley BS, Mehl GL *et al*: Mobile technologies to support healthcare provider to healthcare provider communication and management of care. *Cochrane Database of Systematic Reviews* 2020(8). - Gois PF, Souza EdM: Pharmacotherapy for hyperuricaemia in hypertensive patients. *Cochrane Database of Systematic Reviews* 2020(9). - Gnesin F, Thuesen AC, Kähler LK, Madsbad S, Hemmingsen B: Metformin monotherapy for adults with type 2 diabetes mellitus. *Cochrane Database of Systematic Reviews* 2020(6). - Glujovsky D, Pesce R, Sueldo C, Quinteiro Retamar AM, Hart RJ, Ciapponi A: Endometrial preparation for women undergoing embryo transfer with frozen embryos or embryos derived from donor oocytes. *Cochrane Database of Systematic Reviews* 2020(10). - Gillespie BM, Walker RM, Latimer SL, Thalib L, Whitty JA, McInnes E, Chaboyer WP: Repositioning for pressure injury prevention in adults. *Cochrane Database of Systematic Reviews* 2020(6). - Giguère A, Zomahoun HT, Carmichael PH, Uwizeye CB, Légaré F, Grimshaw JM, Gagnon MP, Auguste DU, Massougbodji J: Printed educational materials: effects on professional practice and healthcare outcomes. *Cochrane Database of Systematic Reviews* 2020(8). - Gibbon S, Khalifa NR, Cheung N-Y, Völlm BA, McCarthy L: Psychological interventions for antisocial personality disorder. *Cochrane Database of Systematic Reviews* 2020(9). - Gibbison B, Villalobos Lizardi JC, Avilés Martínez KI, Fudulu DP, Medina Andrade M, Pérez-Gaxiola G, Schadenberg AWL, Stoica SC, Lightman SL, Angelini GD *et al*: Prophylactic corticosteroids for paediatric heart surgery with cardiopulmonary bypass. *Cochrane Database of Systematic Reviews* 2020(10). - Ghaeminia H, Nienhuijs MEL, Toedtling V, Perry J, Tummers M, Hoppenreijs TJM, Van der Sanden WJM, Mettes TG: Surgical removal versus retention for the management of asymptomatic disease‐free impacted wisdom teeth. *Cochrane Database of Systematic Reviews* 2020(5). - Geoffrion S, Hills DJ, Ross HM, Pich J, Hill AT, Dalsbø TK, Riahi S, Martínez-Jarreta B, Guay S: Education and training for preventing and minimizing workplace aggression directed toward healthcare workers. *Cochrane Database of Systematic Reviews* 2020(9). - Genco RJ, Graziani F, Hasturk H: Effects of periodontal disease on glycemic control, complications, and incidence of diabetes mellitus. *Periodontology 2000* 2020, 83(1):59-65. - Gattini D, Cifuentes LI, Torres-Robles R, Gana JC: Sclerotherapy versus beta‐blockers for primary prophylaxis of oesophageal variceal bleeding in children and adolescents with chronic liver disease or portal vein thrombosis. *Cochrane Database of Systematic Reviews* 2020(1). - Garrison SR, Korownyk CS, Kolber MR, Allan GM, Musini VM, Sekhon RK, Dugré N: Magnesium for skeletal muscle cramps. *Cochrane Database of Systematic Reviews* 2020(9). - Garjón J, Saiz LC, Azparren A, Gaminde I, Ariz MJ, Erviti J: First‐line combination therapy versus first‐line monotherapy for primary hypertension. *Cochrane Database of Systematic Reviews* 2020(2). - Garegnani L, Franco JVA, Ciapponi A, Garrote V, Vietto V, Portillo Medina SA: Ventriculo‐peritoneal shunting devices for hydrocephalus. *Cochrane Database of Systematic Reviews* 2020(6). - Gao Y, Ren Y, Guo Y-k, Liu X, Xie L-j, Jiang L, Shen M-t, Deng M-y, Yang Z-g: Metabolic syndrome and myocardium steatosis in subclinical type 2 diabetes mellitus: a 1 H-magnetic resonance spectroscopy study. *Cardiovascular Diabetology* 2020, 19:1-11. - Gao Y, Ren Y, Guo Y-k, Liu X, Xie L-j, Jiang L, Shen M-t, Deng M-y, Yang Z-g: Metabolic syndrome and myocardium steatosis in subclinical type 2 diabetes mellitus: a 1 H-magnetic resonance spectroscopy study. *Cardiovascular Diabetology* 2020, 19:1-11. - Gao Y, Ren Y, Guo Y-k, Liu X, Xie L-j, Jiang L, Shen M-t, Deng M-y, Yang Z-g: Metabolic syndrome and myocardium steatosis in subclinical type 2 diabetes mellitus: a 1 H-magnetic resonance spectroscopy study. *Cardiovascular Diabetology* 2020, 19:1-11. - Ganslev CA, Storebø OJ, Callesen HE, Ruddy R, Søgaard U: Psychosocial interventions for conversion and dissociative disorders in adults. *Cochrane Database of Systematic Reviews* 2020(7). - Gana JC, Cifuentes LI, Gattini D, Torres-Robles R: Band ligation versus sclerotherapy for primary prophylaxis of oesophageal variceal bleeding in children with chronic liver disease or portal vein thrombosis. *Cochrane Database of Systematic Reviews* 2020(11). - Gambir K, Kim C, Necastro KA, Ganatra B, Ngo TD: Self‐administered versus provider‐administered medical abortion. *Cochrane Database of Systematic Reviews* 2020(3). - Fuller T, Cima R, Langguth B, Mazurek B, Vlaeyen JWS, Hoare DJ: Cognitive behavioural therapy for tinnitus. *Cochrane Database of Systematic Reviews* 2020(1). - Fransen AF, van de Ven J, Banga FR, Mol BWJ, Oei SG: Multi‐professional simulation‐based team training in obstetric emergencies for improving patient outcomes and trainees' performance. *Cochrane Database of Systematic Reviews* 2020(12). - Fraison E, Kostova E, Moran LJ, Bilal S, Ee CC, Venetis C, Costello MF: Metformin versus the combined oral contraceptive pill for hirsutism, acne, and menstrual pattern in polycystic ovary syndrome. *Cochrane Database of Systematic Reviews* 2020(8). - Fortescue R, Kew KM, Leung MT: Sublingual immunotherapy for asthma. *Cochrane Database of Systematic Reviews* 2020(9). - Foley K, Gupta AK, Versteeg S, Mays R, Villanueva E, John D: Topical and device‐based treatments for fungal infections of the toenails. *Cochrane Database of Systematic Reviews* 2020(1). - Fee PA, Riley P, Worthington HV, Clarkson JE, Boyers D, Beirne PV: Recall intervals for oral health in primary care patients. *Cochrane Database of Systematic Reviews* 2020(10). - Fee PA, Macey R, Walsh T, Clarkson JE, Ricketts D: Tests to detect and inform the diagnosis of root caries. *Cochrane Database of Systematic Reviews* 2020(12). - Farrell D, Artom M, Czuber‐Dochan W, Jelsness‐Jørgensen LP, Norton C, Savage E: Interventions for fatigue in inflammatory bowel disease. *Cochrane Database of Systematic Reviews* 2020(4). - Estcourt LJ, Kohli R, Hopewell S, Trivella M, Wang WC: Blood transfusion for preventing primary and secondary stroke in people with sickle cell disease. *Cochrane Database of Systematic Reviews* 2020(7). - Erken Pamukcu H, Hepşen S, Şahan HF, Biçer T, Çakal E, Çimen T, Efe TH, Sunman H: Diabetic microvascular complications associated with myocardial repolarization heterogeneity evaluated by Tp-e interval and Tp-e/QTc ratio. *Journal of diabetes and its complications* 2020, 34(12):107726-107726. - Elsner B, Kugler J, Pohl M, Mehrholz J: Transcranial direct current stimulation (tDCS) for improving activities of daily living, and physical and cognitive functioning, in people after stroke. *Cochrane Database of Systematic Reviews* 2020(11). - Elkholy H, Elhabiby M, Ibrahim I: Rates of alexithymia and its association with smartphone addiction among a sample of university students in Egypt. *Frontiers in Psychiatry* 2020, 11:304. - Egger SJ, Chan MM, Luo Q, Wilcken N: Platinum‐containing regimens for triple‐negative metastatic breast cancer. *Cochrane Database of Systematic Reviews* 2020(10). - Ebadi S, Henschke N, Forogh B, Nakhostin Ansari N, van Tulder MW, Babaei-Ghazani A, Fallah E: Therapeutic ultrasound for chronic low back pain. *Cochrane Database of Systematic Reviews* 2020(7). - Dubourg J, Perrimond-Dauchy S, Felices M, Bolze S, Voiriot P, Fouqueray P: Absence of QTc prolongation in a thorough QT study with imeglimin, a first in class oral agent for type 2 diabetes mellitus. *Eur J Clin Pharmacol* 2020, 76(10):1393-1400. - Dubourg J, Perrimond-Dauchy S, Felices M, Bolze S, Voiriot P, Fouqueray P: Absence of QTc prolongation in a thorough QT study with imeglimin, a first in class oral agent for type 2 diabetes mellitus. *Eur J Clin Pharmacol* 2020, 76(10):1393-1400. - 1030. Duarte GS, Rodrigues FB, Castelão M, Marques RE, Ferreira J, Sampaio C, Moore AP, Costa J: Botulinum toxin type A therapy for hemifacial spasm. *Cochrane Database of Systematic Reviews* 2020(11). - Donovan T, Milan SJ, Wang R, Banchoff E, Bradley P, Crossingham I: Anti‐IL‐5 therapies for chronic obstructive pulmonary disease. *Cochrane Database of Systematic Reviews* 2020(12). - Doets AY, Hughes RAC, Brassington R, Hadden RDM, Pritchard J: Pharmacological treatment other than corticosteroids, intravenous immunoglobulin and plasma exchange for Guillain‐Barré syndrome. *Cochrane Database of Systematic Reviews* 2020(1). - Dipper A, Jones HE, Bhatnagar R, Preston NJ, Maskell N, Clive AO: Interventions for the management of malignant pleural effusions: a network meta‐analysis. *Cochrane Database of Systematic Reviews* 2020(4). - Dhawan S, Patil CG, Chen C, Venteicher AS: Early versus delayed postoperative radiotherapy for treatment of low‐grade gliomas. *Cochrane Database of Systematic Reviews* 2020(1). - Dhaliwal KK, Upadhya B, Soliman EZ, Beaty EH, Yeboah J, Bhave PD, Whalen SP, Singleton MJ: Association of P-Wave Axis With Incident Atrial Fibrillation in Diabetes Mellitus (from the ACCORD Trial). *Am J Cardiol* 2020, 128:191-195. - Dhaliwal KK, Upadhya B, Soliman EZ, Beaty EH, Yeboah J, Bhave PD, Whalen SP, Singleton MJ: Association of P-Wave Axis With Incident Atrial Fibrillation in Diabetes Mellitus (from the ACCORD Trial). *Am J Cardiol* 2020, 128:191-195. - Denison HJ, Worswick J, Bond CM, Grimshaw JM, Mayhew A, Gnani Ramadoss S, Robertson C, Schaafsma ME, Watson MC: Oral versus intra‐vaginal imidazole and triazole anti‐fungal treatment of uncomplicated vulvovaginal candidiasis (thrush). *Cochrane Database of Systematic Reviews* 2020(8). - Davies SC, Hussein IM, Nguyen TM, Parker CE, Khanna R, Jairath V: Oral Janus kinase inhibitors for maintenance of remission in ulcerative colitis. *Cochrane Database of Systematic Reviews* 2020(1). - Datta SS, Daruvala R, Kumar A: Psychological interventions for psychosis in adolescents. *Cochrane Database of Systematic Reviews* 2020(7). - Cumpstey AF, Oldman AH, Smith AF, Martin D, Grocott MPW: Oxygen targets in the intensive care unit during mechanical ventilation for acute respiratory distress syndrome: a rapid review. *Cochrane Database of Systematic Reviews* 2020(9). - Crepinsek MA, Taylor EA, Michener K, Stewart F: Interventions for preventing mastitis after childbirth. *Cochrane Database of Systematic Reviews* 2020(9). - Craig SS, Dalziel SR, Powell CVE, Graudins A, Babl FE, Lunny C: Interventions for escalation of therapy for acute exacerbations of asthma in children: an overview of Cochrane Reviews. *Cochrane Database of Systematic Reviews* 2020(8). - Craig RS, Goodier H, Singh JA, Hopewell S, Rees JL: Shoulder replacement surgery for osteoarthritis and rotator cuff tear arthropathy. *Cochrane Database of Systematic Reviews* 2020(4). - Cowdell F, Jadotte YT, Ersser SJ, Danby S, Lawton S, Roberts A, Dyson J: Hygiene and emollient interventions for maintaining skin integrity in older people in hospital and residential care settings. *Cochrane Database of Systematic Reviews* 2020(1). - Coveney S, McCabe JJ, Murphy S, O'Donnell M, Kelly PJ: Anti‐inflammatory therapy for preventing stroke and other vascular events after ischaemic stroke or transient ischaemic attack. *Cochrane Database of Systematic Reviews* 2020(5). - Cote S, Zhang AC, Ahmadzai V, Maleken A, Li C, Oppedisano J, Nair K, Busija L, Downie LE: Intense pulsed light (IPL) therapy for the treatment of meibomian gland dysfunction. *Cochrane Database of Systematic Reviews* 2020(3). - Cornelisse S, Zagers M, Kostova E, Fleischer K, van Wely M, Mastenbroek S: Preimplantation genetic testing for aneuploidies (abnormal number of chromosomes) in in vitro fertilisation. *Cochrane Database of Systematic Reviews* 2020(9). - Collinson S, Deans A, Padua-Zamora A, Gregorio GV, Li C, Dans LF, Allen SJ: Probiotics for treating acute infectious diarrhoea. *Cochrane Database of Systematic Reviews* 2020(12). - Cohen JF, Pauchard JY, Hjelm N, Cohen R, Chalumeau M: Efficacy and safety of rapid tests to guide antibiotic prescriptions for sore throat. *Cochrane Database of Systematic Reviews* 2020(6). - Coffey MJ, Garg M, Homaira N, Jaffe A, Ooi CY: Probiotics for people with cystic fibrosis. *Cochrane Database of Systematic Reviews* 2020(1). - Chung EYM, Ruospo M, Natale P, Bolignano D, Navaneethan SD, Palmer SC, Strippoli GFM: Aldosterone antagonists in addition to renin angiotensin system antagonists for preventing the progression of chronic kidney disease. *Cochrane Database of Systematic Reviews* 2020(10). - Christensen RH, von Scholten BJ, Lehrskov LL, Rossing P, Jørgensen PG: Epicardial adipose tissue: an emerging biomarker of cardiovascular complications in type 2 diabetes? *Therapeutic Advances in Endocrinology and Metabolism* 2020, 11:2042018820928824. - Chiu HC, Ada L, Bania TA: Mechanically assisted walking training for walking, participation, and quality of life in children with cerebral palsy. *Cochrane Database of Systematic Reviews* 2020(11). - Chen M, Ng SM, Akpek EK, Ahmad S: Artificial corneas versus donor corneas for repeat corneal transplants. *Cochrane Database of Systematic Reviews* 2020(5). - Chen H, Song Z, Dennis JA: Hypertonic saline versus other intracranial pressure–lowering agents for people with acute traumatic brain injury. *Cochrane Database of Systematic Reviews* 2020(1). - Chandelia S, Kumar D, Chadha N, Jaiswal N: Magnesium sulphate for treating acute bronchiolitis in children up to two years of age. *Cochrane Database of Systematic Reviews* 2020(12). - Chan S, Ng S, Chan HP, Pascoe EM, Playford EG, Wong G, Chapman JR, Lim WH, Francis RS, Isbel NM *et al*: Perioperative antibiotics for preventing post‐surgical site infections in solid organ transplant recipients. *Cochrane Database of Systematic Reviews* 2020(8). - Chan KK, Joo DA, McRae AD, Takwoingi Y, Premji ZA, Lang E, Wakai A: Chest ultrasonography versus supine chest radiography for diagnosis of pneumothorax in trauma patients in the emergency department. *Cochrane Database of Systematic Reviews* 2020(7). - Ceraso A, Lin JJ, Schneider-Thoma J, Siafis S, Tardy M, Komossa K, Heres S, Kissling W, Davis JM, Leucht S: Maintenance treatment with antipsychotic drugs for schizophrenia. *Cochrane Database of Systematic Reviews* 2020(8). - Celikkalp U, Bilgic S, Temel M, Varol G: The smartphone addiction levels and the association with communication skills in nursing and medical school students. *Journal of Nursing Research* 2020, 28(3):e93. - Carney T, Van Hout MC, Norman I, Dada S, Siegfried N, Parry CDH: Dihydrocodeine for detoxification and maintenance treatment in individuals with opiate use disorders. *Cochrane Database of Systematic Reviews* 2020(2). - Candia R, Bravo-Soto GA, Monrroy H, Hernandez C, Nguyen GC: Colonoscopy‐guided therapy for the prevention of post‐operative recurrence of Crohn’s disease. *Cochrane Database of Systematic Reviews* 2020(8). - Campbell P, Bain B, Furlanetto DLC, Brady MC: Interventions for improving oral health in people after stroke. *Cochrane Database of Systematic Reviews* 2020(12). - Campbell K, Coleman-Haynes T, Bowker K, Cooper SE, Connelly S, Coleman T: Factors influencing the uptake and use of nicotine replacement therapy and e‐cigarettes in pregnant women who smoke: a qualitative evidence synthesis. *Cochrane Database of Systematic Reviews* 2020(5). - Cahill LS, Carey LM, Lannin NA, Turville M, Neilson CL, Lynch EA, McKinstry CE, Han JX, O'Connor D: Implementation interventions to promote the uptake of evidence‐based practices in stroke rehabilitation. *Cochrane Database of Systematic Reviews* 2020(10). - Cacione DG, Macedo CR, do Carmo Novaes F, Baptista-Silva JCC: Pharmacological treatment for Buerger's disease. *Cochrane Database of Systematic Reviews* 2020(5). - Burton MJ, Clarkson JE, Goulao B, Glenny AM, McBain AJ, Schilder AGM, Webster KE, Worthington HV: Antimicrobial mouthwashes (gargling) and nasal sprays to protect healthcare workers when undertaking aerosol‐generating procedures (AGPs) on patients without suspected or confirmed COVID‐19 infection. *Cochrane Database of Systematic Reviews* 2020(9). - Burton MJ, Clarkson JE, Goulao B, Glenny AM, McBain AJ, Schilder AGM, Webster KE, Worthington HV: Use of antimicrobial mouthwashes (gargling) and nasal sprays by healthcare workers to protect them when treating patients with suspected or confirmed COVID‐19 infection. *Cochrane Database of Systematic Reviews* 2020(9). - Burton MJ, Clarkson JE, Goulao B, Glenny AM, McBain AJ, Schilder AGM, Webster KE, Worthington HV: Antimicrobial mouthwashes (gargling) and nasal sprays administered to patients with suspected or confirmed COVID‐19 infection to improve patient outcomes and to protect healthcare workers treating them. *Cochrane Database of Systematic Reviews* 2020(9). - Burge AT, Cox NS, Abramson MJ, Holland AE: Interventions for promoting physical activity in people with chronic obstructive pulmonary disease (COPD). *Cochrane Database of Systematic Reviews* 2020(4). - Bui KT, Willson ML, Goel S, Beith J, Goodwin A: Ovarian suppression for adjuvant treatment of hormone receptor‐positive early breast cancer. *Cochrane Database of Systematic Reviews* 2020(3). - Bugge C, Adams EJ, Gopinath D, Stewart F, Dembinsky M, Sobiesuo P, Kearney R: Pessaries (mechanical devices) for managing pelvic organ prolapse in women. *Cochrane Database of Systematic Reviews* 2020(11). - Bruschettini M, Moresco L, Calevo MG, Romantsik O: Postnatal corticosteroids for transient tachypnoea of the newborn. *Cochrane Database of Systematic Reviews* 2020(3). - Broderick C, Pagnamenta F, Forster R: Dressings and topical agents for arterial leg ulcers. *Cochrane Database of Systematic Reviews* 2020(1). - Brigo F, Lattanzi S, Igwe SC, Behzadifar M, Bragazzi NL: Zonisamide add‐on therapy for focal epilepsy. *Cochrane Database of Systematic Reviews* 2020(7). - Bresnahan R, Panebianco M, Marson AG: Lamotrigine add‐on therapy for drug‐resistant generalised tonic‐clonic seizures. *Cochrane Database of Systematic Reviews* 2020(7). - Brennan‐Jones CG, Head K, Chong LY, Burton MJ, Schilder AGM, Bhutta MF: Topical antibiotics for chronic suppurative otitis media. *Cochrane Database of Systematic Reviews* 2020(1). - Brennan-Jones CG, Chong LY, Head K, Burton MJ, Schilder AGM, Bhutta MF: Topical antibiotics with steroids for chronic suppurative otitis media. *Cochrane Database of Systematic Reviews* 2020(8). - Bonora E, Trombetta M, Dauriz M, Travia D, Cacciatori V, Brangani C, Negri C, Perrone F, Pichiri I, Stoico V: Chronic complications in patients with newly diagnosed type 2 diabetes: prevalence and related metabolic and clinical features: the Verona Newly Diagnosed Type 2 Diabetes Study (VNDS) 9. *BMJ Open Diabetes Research and Care* 2020, 8(1):e001549. - Bofill Rodriguez M, Lethaby A, Farquhar C, Duffy JMN: Interventions commonly available during pandemics for heavy menstrual bleeding: an overview of Cochrane Reviews. *Cochrane Database of Systematic Reviews* 2020(7). - Bina S, Pacey V, Barnes EH, Burns J, Gray K: Interventions for congenital talipes equinovarus (clubfoot). *Cochrane Database of Systematic Reviews* 2020(5). - Bhutta MF, Head K, Chong LY, Daw J, Schilder AGM, Burton MJ, Brennan-Jones CG: Aural toilet (ear cleaning) for chronic suppurative otitis media. *Cochrane Database of Systematic Reviews* 2020(9). - Best LMJ, Leung J, Freeman SC, Sutton AJ, Cooper NJ, Milne EJ, Cowlin M, Payne A, Walshaw D, Thorburn D *et al*: Induction immunosuppression in adults undergoing liver transplantation: a network meta‐analysis. *Cochrane Database of Systematic Reviews* 2020(1). - Benstoem C, Kalvelage C, Breuer T, Heussen N, Marx G, Stoppe C, Brandenburg V: Ivabradine as adjuvant treatment for chronic heart failure. *Cochrane Database of Systematic Reviews* 2020(11). - Bennett C, Green S, DeCaestecker J, Almond M, Barr H, Bhandari P, Ragunath K, Singh R, Jankowski J: Surgery versus radical endotherapies for early cancer and high‐grade dysplasia in Barrett's oesophagus. *Cochrane Database of Systematic Reviews* 2020(5). - Beevers Z, Hussain S, Boele FW, Rooney AG: Pharmacological treatment of depression in people with a primary brain tumour. *Cochrane Database of Systematic Reviews* 2020(7). - Ball EL, Owen-Booth B, Gray A, Shenkin SD, Hewitt J, McCleery J: Aromatherapy for dementia. *Cochrane Database of Systematic Reviews* 2020(8). - Balikai F, Deshpande N, Javali S, Shetty D, Benni J, Shindhe V, Jaalam K, Kapoor N: The relationship between serum triglyceride level and heart rate variability in type 2 diabetes mellitus patients of North Karnataka. *Journal of diabetology* 2020, 11(3):191-197. - Bala MM, Malecka-Massalska TJ, Koperny M, Zajac JF, Jarczewski JD, Szczeklik W: Anti‐cytokine targeted therapies for ANCA‐associated vasculitis. *Cochrane Database of Systematic Reviews* 2020(9). - Bajwah S, Oluyase AO, Yi D, Gao W, Evans CJ, Grande G, Todd C, Costantini M, Murtagh FE, Higginson IJ: The effectiveness and cost‐effectiveness of hospital‐based specialist palliative care for adults with advanced illness and their caregivers. *Cochrane Database of Systematic Reviews* 2020(9). - Bailey E, Kashbour W, Shah N, Worthington HV, Renton TF, Coulthard P: Surgical techniques for the removal of mandibular wisdom teeth. *Cochrane Database of Systematic Reviews* 2020(7). - Aziz A, Ohlsson A: Surfactant for pulmonary haemorrhage in neonates. *Cochrane Database of Systematic Reviews* 2020(2). - Avila ML, Shah PS, Brandão LR: Different unfractionated heparin doses for preventing arterial thrombosis in children undergoing cardiac catheterization. *Cochrane Database of Systematic Reviews* 2020(2). - Askland K, Wright L, Wozniak DR, Emmanuel T, Caston J, Smith I: Educational, supportive and behavioural interventions to improve usage of continuous positive airway pressure machines in adults with obstructive sleep apnoea. *Cochrane Database of Systematic Reviews* 2020(4). - Arnold SV, Bhatt DL, Barsness GW, Beatty AL, Deedwania PC, Inzucchi SE, Kosiborod M, Leiter LA, Lipska KJ, Newman JD: Clinical management of stable coronary artery disease in patients with type 2 diabetes mellitus: a scientific statement from the American Heart Association. *Circulation* 2020, 141(19):e779-e806. - Arnold SV, Bhatt DL, Barsness GW, Beatty AL, Deedwania PC, Inzucchi SE, Kosiborod M, Leiter LA, Lipska KJ, Newman JD: Clinical management of stable coronary artery disease in patients with type 2 diabetes mellitus: a scientific statement from the American Heart Association. *Circulation* 2020, 141(19):e779-e806. - Arguedas JA, Leiva V, Wright JM: Blood pressure targets in adults with hypertension. *Cochrane Database of Systematic Reviews* 2020(12). - Argaw D, Hussen Kabthymer R, Birhane M: Magnitude of anemia and its associated factors among pregnant women attending antenatal care in Southern Ethiopia: a cross-sectional study. *Journal of blood medicine* 2020:335-344. - Anglemyer A, Moore THM, Parker L, Chambers T, Grady A, Chiu K, Parry M, Wilczynska M, Flemyng E, Bero L: Digital contact tracing technologies in epidemics: a rapid review. *Cochrane Database of Systematic Reviews* 2020(8). - Andrade A, Kuah CY, Martin‐Lopez JE, Chua S, Shpadaruk V, Sanclemente G, Franco JVA: Interventions for chronic pruritus of unknown origin. *Cochrane Database of Systematic Reviews* 2020(1). - Andersen A, Jørgensen PG, Knop FK, Vilsbøll T: Hypoglycaemia and cardiac arrhythmias in diabetes. *Therapeutic Advances in Endocrinology and Metabolism* 2020, 11:2042018820911803. - Andersen A, Jørgensen PG, Knop FK, Vilsbøll T: Hypoglycaemia and cardiac arrhythmias in diabetes. *Therapeutic Advances in Endocrinology and Metabolism* 2020, 11:2042018820911803. - Amez S, Baert S: Smartphone use and academic performance: A literature review. *International journal of educational research* 2020, 103:101618. - Allida S, Du H, Xu X, Prichard R, Chang S, Hickman LD, Davidson PM, Inglis SC: mHealth education interventions in heart failure. *Cochrane Database of Systematic Reviews* 2020(7). - Allida S, Cox KL, Hsieh CF, House A, Hackett ML: Pharmacological, psychological and non‐invasive brain stimulation interventions for preventing depression after stroke. *Cochrane Database of Systematic Reviews* 2020(5). - Alfirevic Z, Gyte GML, Nogueira Pileggi V, Plachcinski R, Osoti AO, Finucane EM: Home versus inpatient induction of labour for improving birth outcomes. *Cochrane Database of Systematic Reviews* 2020(8). - Al-aqeel S, Gershuni O, Al-sabhan J, Hiligsmann M: Strategies for improving adherence to antiepileptic drug treatment in people with epilepsy. *Cochrane Database of Systematic Reviews* 2020(10). - Al Asqah MI, Al Orainey AI, Shukr MA, Al Oraini HM, Al Turki YA: The prevalence of internet gaming disorder among medical students at King Saud University, Riyadh, Saudi Arabia. A cross-sectional study. *Saudi Med J* 2020, 41(12):1359-1363. - Akalu Y, Belsti Y: Hypertension and its associated factors among type 2 diabetes mellitus patients at Debre Tabor general hospital, northwest Ethiopia. *Diabetes, Metabolic Syndrome and Obesity* 2020:1621-1631. - Ahmed RR, Salman F, Malik SA, Streimikiene D, Soomro RH, Pahi MH: Smartphone use and academic performance of university students: a mediation and moderation analysis. *Sustainability* 2020, 12(1):439. - Ahmed RR, Salman F, Malik SA, Streimikiene D, Soomro RH, Pahi MH: Smartphone use and academic performance of university students: A mediation and moderation analysis. *Sustainability* 2020, 12(1):439. - Ahmed I, Chawla A, Underwood M, Price AJ, Metcalfe A, Hutchinson C, Warwick J, Seers K, Parsons H, Wall PDH: Tourniquet use for knee replacement surgery. *Cochrane Database of Systematic Reviews* 2020(12). - Agarwal R, Choi L, Johnson S, Takwoingi Y: Rapid diagnostic tests for Plasmodium vivax malaria in endemic countries. *Cochrane Database of Systematic Reviews* 2020(11). - Acin MT, Rueda JR, Saiz LC, Parent Mathias V, Alzueta N, Solà I, Garjón J, Erviti J: Alcohol intake reduction for controlling hypertension. *Cochrane Database of Systematic Reviews* 2020(9). - Aali G, Kariotis T, Shokraneh F: Avatar Therapy for people with schizophrenia or related disorders. *Cochrane Database of Systematic Reviews* 2020(5). - Znaor L, Medic A, Binder S, Vucinovic A, Marin Lovric J, Puljak L: Pars plana vitrectomy versus scleral buckling for repairing simple rhegmatogenous retinal detachments. *Cochrane Database of Systematic Reviews* 2019(3). - Zhelev Z, Walker G, Henschke N, Fridhandler J, Yip S: Prehospital stroke scales as screening tools for early identification of stroke and transient ischemic attack. *Cochrane Database of Systematic Reviews* 2019(4). - Ye L, Cao Y, Yang W, Wu F, Lin J, Li L, Li C: Graft interposition for preventing Frey's syndrome in patients undergoing parotidectomy. *Cochrane Database of Systematic Reviews* 2019(10). - Yang B, de Vries SG, Ahmed A, Visser BJ, Nagel IM, Spijker R, Grobusch MP, Hartskeerl RA, Goris MGA, Leeflang MMG: Nucleic acid and antigen detection tests for leptospirosis. *Cochrane Database of Systematic Reviews* 2019(8). - Yamazaki H, So R, Matsuoka K, Kobayashi T, Shinzaki S, Matsuura M, Okabayashi S, Kataoka Y, Tsujimoto Y, Furukawa TA *et al*: Certolizumab pegol for induction of remission in Crohn's disease. *Cochrane Database of Systematic Reviews* 2019(8). - Yamamoto S, Yamaga T, Nishie K, Nagata C, Mori R: Positive airway pressure therapy for the treatment of central sleep apnoea associated with heart failure. *Cochrane Database of Systematic Reviews* 2019(12). - Wrzosek A, Jakowicka‐Wordliczek J, Zajaczkowska R, Serednicki WT, Jankowski M, Bala MM, Swierz MJ, Polak M, Wordliczek J: Perioperative restrictive versus goal‐directed fluid therapy for adults undergoing major non‐cardiac surgery. *Cochrane Database of Systematic Reviews* 2019(12). - Wilsdon TD, Whittle SL, Thynne TRJ, Mangoni AA: Methotrexate for psoriatic arthritis. *Cochrane Database of Systematic Reviews* 2019(1). - West S, Nevitt SJ, Cotton J, Gandhi S, Weston J, Sudan A, Ramirez R, Newton R: Surgery for epilepsy. *Cochrane Database of Systematic Reviews* 2019(6). - Werawatakul Y, Sothornwit J, Laopaiboon M, Lumbiganon P, Kietpeerakool C: Interventions for intra‐operative pain relief during postpartum mini‐laparotomy tubal ligation. *Cochrane Database of Systematic Reviews* 2019(2). - Webster J, Osborne S, Rickard CM, Marsh N: Clinically‐indicated replacement versus routine replacement of peripheral venous catheters. *Cochrane Database of Systematic Reviews* 2019(1). - Wang Y, Lee D-c, Brellenthin AG, Sui X, Church TS, Lavie CJ, Blair SN: Association of muscular strength and incidence of type 2 diabetes. In: *Mayo Clinic Proceedings: 2019*: Elsevier; 2019: 643-651. - Wang Y, Lee D-c, Brellenthin AG, Sui X, Church TS, Lavie CJ, Blair SN: Association of muscular strength and incidence of type 2 diabetes. In: *Mayo Clinic Proceedings: 2019*: Elsevier; 2019: 643-651. - Walsh A, Perrem L, Khashan AS, Henry MT, Ni Chroinin M: Statins versus placebo for people with chronic obstructive pulmonary disease. *Cochrane Database of Systematic Reviews* 2019(7). - Walker GJA, Walker D, Molano Franco D, Grillo‐Ardila CF: Antibiotic treatment for newborns with congenital syphilis. *Cochrane Database of Systematic Reviews* 2019(2). - Wadman RI, van der Pol WL, Bosboom WMJ, Asselman FL, van den Berg LH, Iannaccone ST, Vrancken A: Drug treatment for spinal muscular atrophy type I. *Cochrane Database of Systematic Reviews* 2019(12). - Verhoef LM, van den Bemt BJF, van der Maas A, Vriezekolk JE, Hulscher ME, van den Hoogen FHJ, Jacobs WCH, van Herwaarden N, den Broeder AA: Down‐titration and discontinuation strategies of tumour necrosis factor–blocking agents for rheumatoid arthritis in patients with low disease activity. *Cochrane Database of Systematic Reviews* 2019(5). - van Galen KPM, Engelen ET, Mauser‐Bunschoten EP, van Es RJJ, Schutgens REG: Antifibrinolytic therapy for preventing oral bleeding in patients with haemophilia or Von Willebrand disease undergoing minor oral surgery or dental extractions. *Cochrane Database of Systematic Reviews* 2019(4). - van de Vrie R, Rutten MJ, Asseler JD, Leeflang MMG, Kenter GG, Mol BWJ, Buist M: Laparoscopy for diagnosing resectability of disease in women with advanced ovarian cancer. *Cochrane Database of Systematic Reviews* 2019(3). - Vadera S, Yong CWK, Gluud LL, Morgan MY: Band ligation versus no intervention for primary prevention of upper gastrointestinal bleeding in adults with cirrhosis and oesophageal varices. *Cochrane Database of Systematic Reviews* 2019(6). - Tsujimoto Y, Tsujimoto H, Nakata Y, Kataoka Y, Kimachi M, Shimizu S, Ikenoue T, Fukuma S, Yamamoto Y, Fukuhara S: Dialysate temperature reduction for intradialytic hypotension for people with chronic kidney disease requiring haemodialysis. *Cochrane Database of Systematic Reviews* 2019(7). - Trotti LM, Becker LA: Iron for the treatment of restless legs syndrome. *Cochrane Database of Systematic Reviews* 2019(1). - Treanor CJ, Santin O, Prue G, Coleman H, Cardwell CR, O'Halloran P, Donnelly M: Psychosocial interventions for informal caregivers of people living with cancer. *Cochrane Database of Systematic Reviews* 2019(6). - Townsend CM, Parker CE, MacDonald JK, Nguyen TM, Jairath V, Feagan BG, Khanna R: Antibiotics for induction and maintenance of remission in Crohn's disease. *Cochrane Database of Systematic Reviews* 2019(2). - Toupin April K, Bisaillon J, Welch V, Maxwell LJ, Jüni P, Rutjes AWS, Husni ME, Vincent J, El Hindi T, Wells GA *et al*: Tramadol for osteoarthritis. *Cochrane Database of Systematic Reviews* 2019(5). - Torres MFS, Porfírio GJM, Carvalho APV, Riera R: Non‐invasive positive pressure ventilation for prevention of complications after pulmonary resection in lung cancer patients. *Cochrane Database of Systematic Reviews* 2019(3). - Threapleton CJD, Janjua S, Fortescue R, Baker EH: Head‐to‐head oral prophylactic antibiotic therapy for chronic obstructive pulmonary disease. *Cochrane Database of Systematic Reviews* 2019(5). - Thompson G, Lawrie TA, Kernohan A, Jenkinson MD: Interval brain imaging for adults with cerebral glioma. *Cochrane Database of Systematic Reviews* 2019(12). - Thomas LH, Coupe J, Cross LD, Tan AL, Watkins CL: Interventions for treating urinary incontinence after stroke in adults. *Cochrane Database of Systematic Reviews* 2019(2). - Thebyane L: Antecedents of smartphone purchasing behaviour amongst South African Generation Y students. North-West University (South Africa). Vanderbijlpark Campus; 2019. - Tamaoki MJS, Lenza M, Matsunaga FT, Belloti JC, Matsumoto MH, Faloppa F: Surgical versus conservative interventions for treating acromioclavicular dislocation of the shoulder in adults. *Cochrane Database of Systematic Reviews* 2019(10). - Takeda A, Martin N, Taylor RS, Taylor SJC: Disease management interventions for heart failure. *Cochrane Database of Systematic Reviews* 2019(1). - Taha MH, Shehzad K, Alamro AS, Wadi M: Internet use and addiction among medical students in Qassim University, Saudi Arabia. *Sultan Qaboos University Medical Journal* 2019, 19(2):e142. - Sweeney C, Ryan F, Ledwidge M, Ryan C, McDonald K, Watson C, Pharithi RB, Gallagher J: Natriuretic peptide‐guided treatment for the prevention of cardiovascular events in patients without heart failure. *Cochrane Database of Systematic Reviews* 2019(10). - Suomi A, Evans L, Rodgers B, Taplin S, Cowlishaw S: Couple and family therapies for post‐traumatic stress disorder (PTSD). *Cochrane Database of Systematic Reviews* 2019(12). - Suleman F, Movik E: Pharmaceutical policies: effects of educational or regulatory policies targeting prescribers. *Cochrane Database of Systematic Reviews* 2019(11). - Stevenson JK, Campbell ZC, Webster AC, Chow CK, Tong A, Craig JC, Campbell KL, Lee VWS: eHealth interventions for people with chronic kidney disease. *Cochrane Database of Systematic Reviews* 2019(8). - Stern A, Carrara E, Bitterman R, Yahav D, Leibovici L, Paul M: Early discontinuation of antibiotics for febrile neutropenia versus continuation until neutropenia resolution in people with cancer. *Cochrane Database of Systematic Reviews* 2019(1). - Steed L, Sohanpal R, Todd A, Madurasinghe VW, Rivas C, Edwards EA, Summerbell CD, Taylor SJC, Walton RT: Community pharmacy interventions for health promotion: effects on professional practice and health outcomes. *Cochrane Database of Systematic Reviews* 2019(12). - Srijithesh PR, Aghoram R, Goel A, Dhanya J: Positional therapy for obstructive sleep apnoea. *Cochrane Database of Systematic Reviews* 2019(5). - Soomro KA, Zai SAY, Hina QA: Investigating the impact of university students’ smartphone addiction on their satisfaction with classroom connectedness. *Education and Information Technologies* 2019, 24(6):3523-3535. - Solomon SD, Lindsley K, Vedula SS, Krzystolik MG, Hawkins BS: Anti‐vascular endothelial growth factor for neovascular age‐related macular degeneration. *Cochrane Database of Systematic Reviews* 2019(3). - Singh B, Cocker D, Ryan H, Sloan DJ: Linezolid for drug‐resistant pulmonary tuberculosis. *Cochrane Database of Systematic Reviews* 2019(3). - Sinclair DJM, Zhao S, Qi F, Nyakyoma K, Kwong JSW, Adams CE: Electroconvulsive therapy for treatment‐resistant schizophrenia. *Cochrane Database of Systematic Reviews* 2019(3). - Simó R, Bañeras J, Hernández C, Rodríguez-Palomares J, Valente F, Gutierrez L, González-Alujas T, Ferreira I, Aguadé-Bruix S, Montaner J *et al*: Diabetic retinopathy as an independent predictor of subclinical cardiovascular disease: baseline results of the PRECISED study. *BMJ open diabetes research & care* 2019, 7(1):e000845-e000845. - Shimmin D, Lowdon J, Remmington T: Enteral tube feeding for cystic fibrosis. *Cochrane Database of Systematic Reviews* 2019(7). - Shao Q, Meng L, Lee S, Tse G, Gong M, Zhang Z, Zhao J, Zhao Y, Li G, Liu T: Empagliflozin, a sodium glucose co-transporter-2 inhibitor, alleviates atrial remodeling and improves mitochondrial function in high-fat diet/streptozotocin-induced diabetic rats. *Cardiovascular diabetology* 2019, 18(1):165-165. - Segboer C, Gevorgyan A, Avdeeva K, Chusakul S, Kanjanaumporn J, Aeumjaturapat S, Reeskamp LF, Snidvongs K, Fokkens W: Intranasal corticosteroids for non‐allergic rhinitis. *Cochrane Database of Systematic Reviews* 2019(11). - Schouten B, Avau B, Bekkering G, Vankrunkelsven P, Mebis J, Hellings J, Van Hecke A: Systematic screening and assessment of psychosocial well‐being and care needs of people with cancer. *Cochrane Database of Systematic Reviews* 2019(3). - Schnabel A, Reichl SU, Weibel S, Zahn PK, Kranke P, Pogatzki‐Zahn E, Meyer‐Frießem CH: Adductor canal blocks for postoperative pain treatment in adults undergoing knee surgery. *Cochrane Database of Systematic Reviews* 2019(10). - Schmidt L, Phelps E, Friedel J, Shokraneh F: Acetylsalicylic acid (aspirin) for schizophrenia. *Cochrane Database of Systematic Reviews* 2019(8). - Schildmann J, Nadolny S, Haltaufderheide J, Gysels M, Vollmann J, Bausewein C: Ethical case interventions for adult patients. *Cochrane Database of Systematic Reviews* 2019(7). - Schenkel AB, Veitz‐Keenan A: Dental cavity liners for Class I and Class II resin‐based composite restorations. *Cochrane Database of Systematic Reviews* 2019(3). - Schell LK, Monsef I, Wöckel A, Skoetz N: Mindfulness‐based stress reduction for women diagnosed with breast cancer. *Cochrane Database of Systematic Reviews* 2019(3). - Santoso DIS, Murthi AK, Yolanda S, Amani P, Ujianti I, Sianipar IR: The impact of cobalamin deficiency on heart function: A study on abnormalities in electrocardiography patterns. *Int J Appl Pharm* 2019, 11(6):33-36. - Santoso DIS, Murthi AK, Yolanda S, Amani P, Ujianti I, Sianipar IR: The impact of cobalamin deficiency on heart function: A study on abnormalities in electrocardiography patterns. *Int J Appl Pharm* 2019, 11. - Santos S, Voerman E, Amiano P, Barros H, Beilin LJ, Bergström A, Charles MA, Chatzi L, Chevrier C, Chrousos GP: Impact of maternal body mass index and gestational weight gain on pregnancy complications: an individual participant data meta‐analysis of European, North American and Australian cohorts. *BJOG: An International Journal of Obstetrics & Gynaecology* 2019, 126(8):984-995. - Santiago de Araújo Pio C, Chaves GSS, Davies P, Taylor RS, Grace SL: Interventions to promote patient utilisation of cardiac rehabilitation. *Cochrane Database of Systematic Reviews* 2019(2). - Salati JA, Leathersich SJ, Williams MJ, Cuthbert A, Tolosa JE: Prophylactic oxytocin for the third stage of labour to prevent postpartum haemorrhage. *Cochrane Database of Systematic Reviews* 2019(4). - Sahuquillo J, Dennis JA: Decompressive craniectomy for the treatment of high intracranial pressure in closed traumatic brain injury. *Cochrane Database of Systematic Reviews* 2019(12). - Safi S, Sethi NJ, Nielsen EE, Feinberg J, Gluud C, Jakobsen JC: Beta‐blockers for suspected or diagnosed acute myocardial infarction. *Cochrane Database of Systematic Reviews* 2019(12). - Rud B, Vejborg TS, Rappeport ED, Reitsma JB, Wille‐Jørgensen P: Computed tomography for diagnosis of acute appendicitis in adults. *Cochrane Database of Systematic Reviews* 2019(11). - Rosenberg JB, Andersen J, Barmettler A: Types of materials for frontalis sling surgery for congenital ptosis. *Cochrane Database of Systematic Reviews* 2019(4). - Roelsgaard IK, Esbensen BA, Østergaard M, Rollefstad S, Semb AG, Christensen R, Thomsen T: Smoking cessation intervention for reducing disease activity in chronic autoimmune inflammatory joint diseases. *Cochrane Database of Systematic Reviews* 2019(9). - Rivas‐Ruiz R, Villasis‐Keever M, Miranda‐Novales G, Castelán‐Martínez OD, Rivas‐Contreras S: Outpatient treatment for people with cancer who develop a low‐risk febrile neutropaenic event. *Cochrane Database of Systematic Reviews* 2019(3). - Rivas C, Vigurs C, Cameron J, Yeo L: A realist review of which advocacy interventions work for which abused women under what circumstances. *Cochrane Database of Systematic Reviews* 2019(6). - Regnaux JP, Davergne T, Palazzo C, Roren A, Rannou F, Boutron I, Lefevre‐Colau MM: Exercise programmes for ankylosing spondylitis. *Cochrane Database of Systematic Reviews* 2019(10). - Rees K, Takeda A, Martin N, Ellis L, Wijesekara D, Vepa A, Das A, Hartley L, Stranges S: Mediterranean‐style diet for the primary and secondary prevention of cardiovascular disease. *Cochrane Database of Systematic Reviews* 2019(3). - Rai BP, Bondad J, Vasdev N, Adshead J, Lane T, Ahmed K, Khan MS, Dasgupta P, Guru K, Chlosta PL *et al*: Robotic versus open radical cystectomy for bladder cancer in adults. *Cochrane Database of Systematic Reviews* 2019(4). - Qutishat M, Sharour LA: Relationship between fear of missing out and academic performance among Omani university students: a descriptive correlation study. *Oman medical journal* 2019, 34(5):404. - Poole P, Sathananthan K, Fortescue R: Mucolytic agents versus placebo for chronic bronchitis or chronic obstructive pulmonary disease. *Cochrane Database of Systematic Reviews* 2019(5). - Pollok J, van Agteren JEM, Esterman AJ, Carson‐Chahhoud KV: Psychological therapies for the treatment of depression in chronic obstructive pulmonary disease. *Cochrane Database of Systematic Reviews* 2019(3). - Pieske B, Tschöpe C, De Boer RA, Fraser AG, Anker SD, Donal E, Edelmann F, Fu M, Guazzi M, Lam CS: How to diagnose heart failure with preserved ejection fraction: the HFA–PEFF diagnostic algorithm: a consensus recommendation from the Heart Failure Association (HFA) of the European Society of Cardiology (ESC). *European heart journal* 2019, 40(40):3297-3317. - Pieske B, Tschöpe C, De Boer RA, Fraser AG, Anker SD, Donal E, Edelmann F, Fu M, Guazzi M, Lam CS: How to diagnose heart failure with preserved ejection fraction: the HFA–PEFF diagnostic algorithm: a consensus recommendation from the Heart Failure Association (HFA) of the European Society of Cardiology (ESC). *European heart journal* 2019, 40(40):3297-3317. - Piechotta V, Jakob T, Langer P, Monsef I, Scheid C, Estcourt LJ, Ocheni S, Theurich S, Kuhr K, Scheckel B *et al*: Multiple drug combinations of bortezomib, lenalidomide, and thalidomide for first‐line treatment in adults with transplant‐ineligible multiple myeloma: a network meta‐analysis. *Cochrane Database of Systematic Reviews* 2019(11). - Pedlow K, McDonough S, Lennon S, Kerr C, Bradbury I: Assisted standing for Duchenne muscular dystrophy. *Cochrane Database of Systematic Reviews* 2019(10). - Peddle‐McIntyre CJ, Singh F, Thomas R, Newton RU, Galvão DA, Cavalheri V: Exercise training for advanced lung cancer. *Cochrane Database of Systematic Reviews* 2019(2). - Peckham EJ, Cooper K, Roberts ER, Agrawal A, Brabyn S, Tew G: Homeopathy for treatment of irritable bowel syndrome. *Cochrane Database of Systematic Reviews* 2019(9). - Patel SR, Rosenberg JB, Barmettler A: Interventions for orbital lymphangioma. *Cochrane Database of Systematic Reviews* 2019(5). - Pantoja T, Grimshaw JM, Colomer N, Castañon C, Leniz Martelli J: Manually‐generated reminders delivered on paper: effects on professional practice and patient outcomes. *Cochrane Database of Systematic Reviews* 2019(12). - Pang D, Hildebrand D, Bachoo P: Thoracic endovascular repair (TEVAR) versus open surgery for blunt traumatic thoracic aortic injury. *Cochrane Database of Systematic Reviews* 2019(2). - Palmer SC, Chung EYM, McGregor DO, Bachmann F, Strippoli GFM: Interventions for preventing bone disease in kidney transplant recipients. *Cochrane Database of Systematic Reviews* 2019(10). - 1198. Palmer JS, Monk AP, Hopewell S, Bayliss LE, Jackson W, Beard DJ, Price AJ: Surgical interventions for symptomatic mild to moderate knee osteoarthritis. *Cochrane Database of Systematic Reviews* 2019(7). - Ordonez M, Hwang EC, Borofsky M, Bakker CJ, Gandhi S, Dahm P: Ureteral stent versus no ureteral stent for ureteroscopy in the management of renal and ureteral calculi. *Cochrane Database of Systematic Reviews* 2019(2). - Ong TG, Gordon M, Banks SSC, Thomas MR, Akobeng AK: Probiotics to prevent infantile colic. *Cochrane Database of Systematic Reviews* 2019(3). - O'Lone EL, Hodson EM, Nistor I, Bolignano D, Webster AC, Craig JC: Parenteral versus oral iron therapy for adults and children with chronic kidney disease. *Cochrane Database of Systematic Reviews* 2019(2). - Nyachieo A, Siristatidis CS, Vaidakis D: Nonsteroidal anti‐inflammatory drugs for assisted reproductive technology. *Cochrane Database of Systematic Reviews* 2019(10). - Nussbaumer‐Streit B, Greenblatt A, Kaminski‐Hartenthaler A, Van Noord MG, Forneris CA, Morgan LC, Gaynes BN, Wipplinger J, Lux LJ, Winkler D *et al*: Melatonin and agomelatine for preventing seasonal affective disorder. *Cochrane Database of Systematic Reviews* 2019(6). - Nussbaumer‐Streit B, Forneris CA, Morgan LC, Van Noord MG, Gaynes BN, Greenblatt A, Wipplinger J, Lux LJ, Winkler D, Gartlehner G: Light therapy for preventing seasonal affective disorder. *Cochrane Database of Systematic Reviews* 2019(3). - Nielsen KM, Zwisler AD, Taylor RS, Svendsen JH, Lindschou J, Anderson L, Jakobsen JC, Berg SK: Exercise‐based cardiac rehabilitation for adult patients with an implantable cardioverter defibrillator. *Cochrane Database of Systematic Reviews* 2019(2). - Nguyen N, Zhang B, Holubar SD, Pardi DS, Singh S: Treatment and prevention of pouchitis after ileal pouch‐anal anastomosis for chronic ulcerative colitis. *Cochrane Database of Systematic Reviews* 2019(11). - Ng O, Keeler BD, Mishra A, Simpson JA, Neal K, Al‐Hassi HO, Brookes MJ, Acheson AG: Iron therapy for preoperative anaemia. *Cochrane Database of Systematic Reviews* 2019(12). - Nevitt SJ, Tudur Smith C, Marson AG: Phenobarbitone versus phenytoin monotherapy for epilepsy: an individual participant data review. *Cochrane Database of Systematic Reviews* 2019(7). - Ndegwa SK: Anemia & its associated factors among pregnant women attending antenatal clinic at Mbagathi county hospital, Nairobi county, Kenya. *African Journal of Health Sciences* 2019, 32(1):59-73. - Natale P, Ruospo M, Saglimbene VM, Palmer SC, Strippoli GFM: Interventions for improving sleep quality in people with chronic kidney disease. *Cochrane Database of Systematic Reviews* 2019(5). - Natale P, Palmer SC, Ruospo M, Saglimbene VM, Rabindranath KS, Strippoli GFM: Psychosocial interventions for preventing and treating depression in dialysis patients. *Cochrane Database of Systematic Reviews* 2019(12). - Musini VM, Tejani AM, Bassett K, Puil L, Wright JM: Pharmacotherapy for hypertension in adults 60 years or older. *Cochrane Database of Systematic Reviews* 2019(6). - Mulder RL, Bresters D, Van den Hof M, Koot BGP, Castellino SM, Loke YKK, Post PN, Postma A, Szőnyi LP, Levitt GA *et al*: Hepatic late adverse effects after antineoplastic treatment for childhood cancer. *Cochrane Database of Systematic Reviews* 2019(4). - Mudano AS, Tugwell P, Wells GA, Singh JA: Tai Chi for rheumatoid arthritis. *Cochrane Database of Systematic Reviews* 2019(9). - Moore ZEH, Patton D: Risk assessment tools for the prevention of pressure ulcers. *Cochrane Database of Systematic Reviews* 2019(1). - Molano Franco D, Arevalo‐Rodriguez I, Roqué i Figuls M, Montero Oleas NG, Nuvials X, Zamora J: Plasma interleukin‐6 concentration for the diagnosis of sepsis in critically ill adults. *Cochrane Database of Systematic Reviews* 2019(4). - Mohammad JA, Yusoff MSB, Taha MH, Shehzad K, Alamro AS, Wadi M: Internet Use and Addiction Among Medical Students in Qassim University, Saudi Arabia. *BMC Psychol* 2019, 19(2):e142-e147. - Miles LF, Litton E, Imberger G, Story D: Intravenous iron therapy for non‐anaemic, iron‐deficient adults. *Cochrane Database of Systematic Reviews* 2019(12). - Merriel A, Ficquet J, Barnard K, Kunutsor SK, Soar J, Lenguerrand E, Caldwell DM, Burden C, Winter C, Draycott T *et al*: The effects of interactive training of healthcare providers on the management of life‐threatening emergencies in hospital. *Cochrane Database of Systematic Reviews* 2019(9). - McShane R, Westby MJ, Roberts E, Minakaran N, Schneider L, Farrimond LE, Maayan N, Ware J, Debarros J: Memantine for dementia. *Cochrane Database of Systematic Reviews* 2019(3). - McKnight RF, de La Motte de Broöns de Vauvert SJ, Chesney E, Amit BH, Geddes J, Cipriani A: Lithium for acute mania. *Cochrane Database of Systematic Reviews* 2019(6). - McIlwaine M, Button B, Nevitt SJ: Positive expiratory pressure physiotherapy for airway clearance in people with cystic fibrosis. *Cochrane Database of Systematic Reviews* 2019(11). - Matterne U, Böhmer MM, Weisshaar E, Jupiter A, Carter B, Apfelbacher CJ: Oral H1 antihistamines as ‘add‐on’ therapy to topical treatment for eczema. *Cochrane Database of Systematic Reviews* 2019(1). - Mathes T, Pieper D, Morche J, Polus S, Jaschinski T, Eikermann M: Pay for performance for hospitals. *Cochrane Database of Systematic Reviews* 2019(7). - Mateo‐Urdiales A, Johnson S, Smith R, Nachega JB, Eshun‐Wilson I: Rapid initiation of antiretroviral therapy for people living with HIV. *Cochrane Database of Systematic Reviews* 2019(6). - Martimbianco ALC, Porfírio GJM, Pacheco RL, Torloni MR, Riera R: Transcutaneous electrical nerve stimulation (TENS) for chronic neck pain. *Cochrane Database of Systematic Reviews* 2019(12). - Martí‐Carvajal AJ, Gluud C, Arevalo‐Rodriguez I, Martí‐Amarista CE: Acetyl‐L‐carnitine for patients with hepatic encephalopathy. *Cochrane Database of Systematic Reviews* 2019(1). - Markkula SP, Leung N, Allen VB, Furniss D: Surgical interventions for the prevention or treatment of lymphoedema after breast cancer treatment. *Cochrane Database of Systematic Reviews* 2019(2). - Maqsood U, Ho TN, Palmer K, Eccles FJR, Munavvar M, Wang R, Crossingham I, Evans DJW: Once daily long‐acting beta2‐agonists and long‐acting muscarinic antagonists in a combined inhaler versus placebo for chronic obstructive pulmonary disease. *Cochrane Database of Systematic Reviews* 2019(3). - Manzotti C, Casazza G, Stimac T, Nikolova D, Gluud C: Total serum bile acids or serum bile acid profile, or both, for the diagnosis of intrahepatic cholestasis of pregnancy. *Cochrane Database of Systematic Reviews* 2019(7). - Malone H, Biggar S, Javadpour S, Edworthy Z, Sheaf G, Coyne I: Interventions for promoting participation in shared decision‐making for children and adolescents with cystic fibrosis. *Cochrane Database of Systematic Reviews* 2019(5). - Mahsan AM: Effect of Social Networking Sites on Students Academic Performance within Private Universities in Kenya. A Case of United States International University-Africa. 2019. - Mahapatra S: Smartphone addiction and associated consequences: Role of loneliness and self-regulation. *Behaviour & Information Technology* 2019, 38(8):833-844. - Madsen KS, Kähler P, Kähler LKA, Madsbad S, Gnesin F, Metzendorf MI, Richter B, Hemmingsen B: Metformin and second‐ or third‐generation sulphonylurea combination therapy for adults with type 2 diabetes mellitus. *Cochrane Database of Systematic Reviews* 2019(4). - Madsen KS, Chi Y, Metzendorf MI, Richter B, Hemmingsen B: Metformin for prevention or delay of type 2 diabetes mellitus and its associated complications in persons at increased risk for the development of type 2 diabetes mellitus. *Cochrane Database of Systematic Reviews* 2019(12). - Livingstone‐Banks J, Ordóñez‐Mena JM, Hartmann‐Boyce J: Print‐based self‐help interventions for smoking cessation. *Cochrane Database of Systematic Reviews* 2019(1). - Livingstone‐Banks J, Norris E, Hartmann‐Boyce J, West R, Jarvis M, Chubb E, Hajek P: Relapse prevention interventions for smoking cessation. *Cochrane Database of Systematic Reviews* 2019(10). - Liu ID, Willis NS, Craig JC, Hodson EM: Interventions for idiopathic steroid‐resistant nephrotic syndrome in children. *Cochrane Database of Systematic Reviews* 2019(11). - Lindson N, Thompson TP, Ferrey A, Lambert JD, Aveyard P: Motivational interviewing for smoking cessation. *Cochrane Database of Systematic Reviews* 2019(7). - Lin Y, He S, Gong J, Liu Z, Ding X, Gong J, Zeng Z, Cheng Y: Continuous veno‐venous hemofiltration for severe acute pancreatitis. *Cochrane Database of Systematic Reviews* 2019(10). - Liang Y, Zhang L, Zeng L, Gordon M, Wen J: Racecadotril for acute diarrhoea in children. *Cochrane Database of Systematic Reviews* 2019(12). - Liang SS, Ying AJ, Affan ET, Kakala BF, Strippoli GFM, Bullingham A, Currow H, Dunn DW, Yeh ZYT: Continuous local anaesthetic wound infusion for postoperative pain after midline laparotomy for colorectal resection in adults. *Cochrane Database of Systematic Reviews* 2019(10). - Liang N, Kong DZ, Ma SS, Lu CL, Yang M, Feng LD, Shen C, Diao RH, Cui LJ, Lu XY *et al*: Radix Sophorae flavescentis versus no intervention or placebo for chronic hepatitis B. *Cochrane Database of Systematic Reviews* 2019(4). - Liang N, Kong DZ, Lu CL, Ma SS, Li YQ, Nikolova D, Jakobsen JC, Gluud C, Liu JP: Radix Sophorae flavescentis versus other drugs or herbs for chronic hepatitis B. *Cochrane Database of Systematic Reviews* 2019(6). - Lewis SR, Schofield‐Robinson OJ, Rhodes S, Smith AF: Chlorhexidine bathing of the critically ill for the prevention of hospital‐acquired infection. *Cochrane Database of Systematic Reviews* 2019(8). - Lewis SR, Pritchard MW, Thomas CM, Smith AF: Pharmacological agents for adults with acute respiratory distress syndrome. *Cochrane Database of Systematic Reviews* 2019(7). - Lewis SR, Pritchard MW, Fawcett LJ, Punjasawadwong Y: Bispectral index for improving intraoperative awareness and early postoperative recovery in adults. *Cochrane Database of Systematic Reviews* 2019(9). - Lenza M, Buchbinder R, Johnston RV, Ferrari BAS, Faloppa F: Surgical versus conservative interventions for treating fractures of the middle third of the clavicle. *Cochrane Database of Systematic Reviews* 2019(1). - Lee T-I, Chen Y-C, Lin Y-K, Chung C-C, Lu Y-Y, Kao Y-H, Chen Y-J: Empagliflozin Attenuates Myocardial Sodium and Calcium Dysregulation and Reverses Cardiac Remodeling in Streptozotocin-Induced Diabetic Rats. *International journal of molecular sciences* 2019, 20(7):1680. - Lee S, Crowe M, Seow CH, Kotze PG, Kaplan GG, Metcalfe A, Ricciuto A, Benchimol EI, Kuenzig ME: The impact of surgical therapies for inflammatory bowel disease on female fertility. *Cochrane Database of Systematic Reviews* 2019(7). - Le JT, Bicket AK, Wang L, Li T: Ab interno trabecular bypass surgery with iStent for open‐angle glaucoma. *Cochrane Database of Systematic Reviews* 2019(3). - Lawrie TA, Liu H, Lu D, Dowswell T, Song H, Wang L, Shi G: Robot‐assisted surgery in gynaecology. *Cochrane Database of Systematic Reviews* 2019(4). - Lawrie TA, Gillespie D, Dowswell T, Evans J, Erridge S, Vale L, Kernohan A, Grant R: Long‐term neurocognitive and other side effects of radiotherapy, with or without chemotherapy, for glioma. *Cochrane Database of Systematic Reviews* 2019(8). - Latorraca COC, Martimbianco ALC, Pachito DV, Torloni MR, Pacheco RL, Pereira JG, Riera R: Palliative care interventions for people with multiple sclerosis. *Cochrane Database of Systematic Reviews* 2019(10). - Larun L, Brurberg KG, Odgaard-Jensen J, Price JR: Exercise therapy for chronic fatigue syndrome. *Cochrane Database of Systematic Reviews* 2019(10). - Lansbury L, Rodrigo C, Leonardi‐Bee J, Nguyen‐Van‐Tam J, Lim WS: Corticosteroids as adjunctive therapy in the treatment of influenza. *Cochrane Database of Systematic Reviews* 2019(2). - Lai NM, Chang SMW, Ng SS, Tan SL, Chaiyakunapruk N, Stanaway F: Animal‐assisted therapy for dementia. *Cochrane Database of Systematic Reviews* 2019(11). - Kwah LK, Webb MT, Goh L, Harvey LA: Rigid dressings versus soft dressings for transtibial amputations. *Cochrane Database of Systematic Reviews* 2019(6). - Kurnaz E, Erdeve Ş S, Özgür S, Keskin M, Özbudak P, Çetinkaya S, Aycan Z: Congenital long-QT syndrome in type 1 diabetes: a unique association. *Turk J Pediatr* 2019, 61(5):791-793. - Kunath F, Jensen K, Pinart M, Kahlmeyer A, Schmidt S, Price CL, Lieb V, Dahm P: Early versus deferred standard androgen suppression therapy for advanced hormone‐sensitive prostate cancer. *Cochrane Database of Systematic Reviews* 2019(6). - Kumbargere Nagraj S, Eachempati P, Uma E, Singh VP, Ismail NM, Varghese E: Interventions for managing halitosis. *Cochrane Database of Systematic Reviews* 2019(12). - Kumar VA, Chandrasekaran V, Brahadeeswari H: Prevalence of smartphone addiction and its effects on sleep quality: A cross-sectional study among medical students. *Industrial psychiatry journal* 2019, 28(1):82-85. - Kumar VA, Chandrasekaran V, Brahadeeswari H: Prevalence of smartphone addiction and its effects on sleep quality: A cross-sectional study among medical students. *Industrial psychiatry journal* 2019, 28(1):82. - Krogsbøll LT, Jørgensen KJ, Gøtzsche PC: General health checks in adults for reducing morbidity and mortality from disease. *Cochrane Database of Systematic Reviews* 2019(1). - Kong DZ, Liang N, Yang GL, Zhang Z, Liu Y, Yang Y, Liu YX, Wang QG, Zhang F, Zhang HY *et al*: Acupuncture for chronic hepatitis B. *Cochrane Database of Systematic Reviews* 2019(8). - Kong DZ, Liang N, Yang GL, Zhang Z, Liu Y, Li J, Liu X, Liang S, Nikolova D, Jakobsen JC *et al*: Xiao Chai Hu Tang, a herbal medicine, for chronic hepatitis B. *Cochrane Database of Systematic Reviews* 2019(11). - Knips L, Bergenthal N, Streckmann F, Monsef I, Elter T, Skoetz N: Aerobic physical exercise for adult patients with haematological malignancies. *Cochrane Database of Systematic Reviews* 2019(1). - Kietpeerakool C, Aue‐aungkul A, Galaal K, Ngamjarus C, Lumbiganon P: Nerve‐sparing radical hysterectomy compared to standard radical hysterectomy for women with early stage cervical cancer (stage Ia2 to IIa). *Cochrane Database of Systematic Reviews* 2019(2). - Khan AA, Khalid A, Iqbal R: Revealing the relationship between smartphone addiction and academic performance of students: Evidences from higher educational Institutes of Pakistan. *Pakistan Administrative Review* 2019, 3(2):74-83. - Keay L, Lindsley K, Tielsch J, Katz J, Schein O: Routine preoperative medical testing for cataract surgery. *Cochrane Database of Systematic Reviews* 2019(1). - Kayssi A, Al‐Jundi W, Papia G, Kucey DS, Forbes T, Rajan DK, Neville R, Dueck AD: Drug‐eluting balloon angioplasty versus uncoated balloon angioplasty for the treatment of in‐stent restenosis of the femoropopliteal arteries. *Cochrane Database of Systematic Reviews* 2019(1). - Kassa GM, Arowojolu AO, Odukogbe A, Yalew AW: Adverse neonatal outcomes of adolescent pregnancy in Northwest Ethiopia. *PloS one* 2019, 14(6):e0218259. - Kashangura R, Jullien S, Garner P, Johnson S: MVA85A vaccine to enhance BCG for preventing tuberculosis. *Cochrane Database of Systematic Reviews* 2019(4). - Karjalainen TV, Jain NB, Heikkinen J, Johnston RV, Page CM, Buchbinder R: Surgery for rotator cuff tears. *Cochrane Database of Systematic Reviews* 2019(12). - Karimi‐Shahanjarini A, Shakibazadeh E, Rashidian A, Hajimiri K, Glenton C, Noyes J, Lewin S, Laurant M, Colvin CJ: Barriers and facilitators to the implementation of doctor‐nurse substitution strategies in primary care: a qualitative evidence synthesis. *Cochrane Database of Systematic Reviews* 2019(4). - Kalyani B, Reddi N, Ampalam P, Kishore R, Elluru S: Depression, anxiety and smartphone addiction among medical students. *IOSR Journal of Dental and Medical Sciences* 2019, 18(2):33-37. - Kaloo P, Armstrong S, Kaloo C, Jordan V: Interventions to reduce shoulder pain following gynaecological laparoscopic procedures. *Cochrane Database of Systematic Reviews* 2019(1). - Jung JH, Reddy B, McCutcheon KA, Borofsky M, Narayan V, Kim MH, Dahm P: Prostatic urethral lift for the treatment of lower urinary tract symptoms in men with benign prostatic hyperplasia. *Cochrane Database of Systematic Reviews* 2019(5). - Jun JE, Lee S-E, Choi MS, Park SW, Hwang Y-C, Kim JH: Clinical factors associated with the recovery of cardiovascular autonomic neuropathy in patients with type 2 diabetes mellitus. *Cardiovascular Diabetology* 2019, 18:1-13. - Jun JE, Lee S-E, Choi MS, Park SW, Hwang Y-C, Kim JH: Clinical factors associated with the recovery of cardiovascular autonomic neuropathy in patients with type 2 diabetes mellitus. *Cardiovascular diabetology* 2019, 18(1):1-13. - Joshi D, Gupta Y, Ganai B, Mortensen C: Endovascular versus open repair of asymptomatic popliteal artery aneurysm. *Cochrane Database of Systematic Reviews* 2019(12). - Jochim J, Rifkin‐Zybutz RP, Geddes J, Cipriani A: Valproate for acute mania. *Cochrane Database of Systematic Reviews* 2019(10). - Jewer JK, Wong MJ, Bird SJ, Habib AS, Parker R, George RB: Supplemental perioperative intravenous crystalloids for postoperative nausea and vomiting. *Cochrane Database of Systematic Reviews* 2019(3). - Jensen J, Schou M, Kistorp C, Faber J, Hansen TW, Jensen MT, Andersen HU, Rossing P, Vilsbøll T, Jørgensen PG: Prevalence of heart failure and the diagnostic value of MR‐proANP in outpatients with type 2 diabetes. *Diabetes, obesity and metabolism* 2019, 21(3):736-740. - Jensen J, Schou M, Kistorp C, Faber J, Hansen TW, Jensen MT, Andersen HU, Rossing P, Vilsbøll T, Jørgensen PG: Prevalence of heart failure and the diagnostic value of MR‐proANP in outpatients with type 2 diabetes. *Diabetes, obesity and metabolism* 2019, 21(3):736-740. - Jeffery M, Hickey BE, Hider PN: Follow‐up strategies for patients treated for non‐metastatic colorectal cancer. *Cochrane Database of Systematic Reviews* 2019(9). - Jeffers L, Reid J, Fitzsimons D, Morrison PJ, Dempster M: Interventions to improve psychosocial well‐being in female BRCA‐mutation carriers following risk‐reducing surgery. *Cochrane Database of Systematic Reviews* 2019(10). - Janjua S, Schmidt S, Ferrer M, Cates CJ: Inhaled steroids with and without regular formoterol for asthma: serious adverse events. *Cochrane Database of Systematic Reviews* 2019(9). - Jain P, Gedam SR, Patil PS: Study of smartphone addiction: prevalence, pattern of use, and personality dimensions among medical students from rural region of central India. *Open Journal of Psychiatry & Allied Sciences* 2019, 10(2):132-138. - Jagannath VA, Pucci E, Asokan GV, Robak EW: Percutaneous transluminal angioplasty for treatment of chronic cerebrospinal venous insufficiency (CCSVI) in people with multiple sclerosis. *Cochrane Database of Systematic Reviews* 2019(5). - Iogna Prat L, Wilson P, Freeman SC, Sutton AJ, Cooper NJ, Roccarina D, Benmassaoud A, Plaz Torres MC, Hawkins N, Cowlin M *et al*: Antibiotic treatment for spontaneous bacterial peritonitis in people with decompensated liver cirrhosis: a network meta‐analysis. *Cochrane Database of Systematic Reviews* 2019(9). - Iheozor‐Ejiofor Z, Gordon M, Clegg A, Freeman SC, Gjuladin‐Hellon T, MacDonald JK, Akobeng AK: Interventions for maintenance of surgically induced remission in Crohn’s disease: a network meta‐analysis. *Cochrane Database of Systematic Reviews* 2019(9). - Hwang EC, Sathianathen NJ, Jung JH, Kim MH, Dahm P, Risk MC: Single‐dose intravesical chemotherapy after nephroureterectomy for upper tract urothelial carcinoma. *Cochrane Database of Systematic Reviews* 2019(5). - Hwang EC, Sathianathen NJ, Imamura M, Kuntz GM, Risk MC, Dahm P: Extended versus standard lymph node dissection for urothelial carcinoma of the bladder in patients undergoing radical cystectomy. *Cochrane Database of Systematic Reviews* 2019(5). - Hwang EC, Jung JH, Borofsky M, Kim MH, Dahm P: Aquablation of the prostate for the treatment of lower urinary tract symptoms in men with benign prostatic hyperplasia. *Cochrane Database of Systematic Reviews* 2019(2). - Hunt GE, Siegfried N, Morley K, Brooke‐Sumner C, Cleary M: Psychosocial interventions for people with both severe mental illness and substance misuse. *Cochrane Database of Systematic Reviews* 2019(12). - Huber J, Stanworth SJ, Doree C, Fortin PM, Trivella M, Brunskill SJ, Hopewell S, Wilkinson KL, Estcourt LJ: Prophylactic plasma transfusion for patients without inherited bleeding disorders or anticoagulant use undergoing non‐cardiac surgery or invasive procedures. *Cochrane Database of Systematic Reviews* 2019(11). - Htay H, Johnson DW, Craig JC, Schena FP, Strippoli GFM, Tong A, Cho Y: Catheter type, placement and insertion techniques for preventing catheter‐related infections in chronic peritoneal dialysis patients. *Cochrane Database of Systematic Reviews* 2019(5). - Høeg BL, Bidstrup PE, Karlsen RV, Friberg AS, Albieri V, Dalton SO, Saltbæk L, Andersen KK, Horsboel TA, Johansen C: Follow‐up strategies following completion of primary cancer treatment in adult cancer survivors. *Cochrane Database of Systematic Reviews* 2019(11). - Ho JJ, Adnan AS, Kueh YC, Ambak NJ, Van Rostenberghe H, Jummaat F: Human albumin infusion for treating oedema in people with nephrotic syndrome. *Cochrane Database of Systematic Reviews* 2019(7). - Herbert G, Perry R, Andersen HK, Atkinson C, Penfold C, Lewis SJ, Ness AR, Thomas S: Early enteral nutrition within 24 hours of lower gastrointestinal surgery versus later commencement for length of hospital stay and postoperative complications. *Cochrane Database of Systematic Reviews* 2019(7). - Henneberger PK, Patel JR, de Groene GJ, Beach J, Tarlo SM, Pal TM, Curti S: Workplace interventions for treatment of occupational asthma. *Cochrane Database of Systematic Reviews* 2019(10). - Hendry K, Green C, McShane R, Noel‐Storr AH, Stott DJ, Anwer S, Sutton AJ, Burton JK, Quinn TJ: AD‐8 for detection of dementia across a variety of healthcare settings. *Cochrane Database of Systematic Reviews* 2019(3). - Hegewald J, Wegewitz UE, Euler U, van Dijk JL, Adams J, Fishta A, Heinrich P, Seidler A: Interventions to support return to work for people with coronary heart disease. *Cochrane Database of Systematic Reviews* 2019(3). - Haywood A, Duc J, Good P, Khan S, Rickett K, Vayne-Bossert P, Hardy JR: Systemic corticosteroids for the management of cancer‐related breathlessness (dyspnoea) in adults. *Cochrane Database of Systematic Reviews* 2019(2). - Hayden JA, Wilson MN, Riley RD, Iles R, Pincus T, Ogilvie R: Individual recovery expectations and prognosis of outcomes in non‐specific low back pain: prognostic factor review. *Cochrane Database of Systematic Reviews* 2019(11). - Hay PJ, Touyz S, Claudino AM, Lujic S, Smith CA, Madden S: Inpatient versus outpatient care, partial hospitalisation and waiting list for people with eating disorders. *Cochrane Database of Systematic Reviews* 2019(1). - Hart MG, Grant GRL, Solyom EF, Grant R: Biopsy versus resection for high‐grade glioma. *Cochrane Database of Systematic Reviews* 2019(6). - Hansen C, Lundh A, Rasmussen K, Hróbjartsson A: Financial conflicts of interest in systematic reviews: associations with results, conclusions, and methodological quality. *Cochrane Database of Systematic Reviews* 2019(8). - Hahn D, Hodson EM, Hamiwka LA, Lee VWS, Chapman JR, Craig JC, Webster AC: Target of rapamycin inhibitors (TOR‐I; sirolimus and everolimus) for primary immunosuppression in kidney transplant recipients. *Cochrane Database of Systematic Reviews* 2019(12). - Gutierrez M, Rodriguez JL, Zamora‐de La Cruz D, Flores Pimentel MA, Jimenez‐Corona A, Novak LC, Cano Hidalgo R, Graue F: Pars plana vitrectomy combined with scleral buckle versus pars plana vitrectomy for giant retinal tear. *Cochrane Database of Systematic Reviews* 2019(12). - Guo Q, Goldenberg JZ, Humphrey C, El Dib R, Johnston BC: Probiotics for the prevention of pediatric antibiotic‐associated diarrhea. *Cochrane Database of Systematic Reviews* 2019(4). - Guay J, Suresh S, Kopp S, Johnson RL: Postoperative epidural analgesia versus systemic analgesia for thoraco‐lumbar spine surgery in children. *Cochrane Database of Systematic Reviews* 2019(1). - Guay J, Kopp S: Epidural analgesia for adults undergoing cardiac surgery with or without cardiopulmonary bypass. *Cochrane Database of Systematic Reviews* 2019(3). - Grant JE, Lust K, Chamberlain SR: Problematic smartphone use associated with greater alcohol consumption, mental health issues, poorer academic performance, and impulsivity. *Journal of behavioral addictions* 2019, 8(2):335-342. - Gonzales MLM, Dans LF, Sio‐Aguilar J: Antiamoebic drugs for treating amoebic colitis. *Cochrane Database of Systematic Reviews* 2019(1). - Gligor Ș, Mozoș I: Indicators of smartphone addiction and stress score in university students. *Wiener klinische Wochenschrift* 2019, 131:120-125. - Gjuladin‐Hellon T, Iheozor‐Ejiofor Z, Gordon M, Akobeng AK: Azathioprine and 6‐mercaptopurine for maintenance of surgically‐induced remission in Crohn's disease. *Cochrane Database of Systematic Reviews* 2019(8). - Gjuladin‐Hellon T, Gordon M, Iheozor‐Ejiofor Z, Akobeng AK: Oral 5‐aminosalicylic acid for maintenance of surgically‐induced remission in Crohn's disease. *Cochrane Database of Systematic Reviews* 2019(6). - Gibbs JC, MacIntyre NJ, Ponzano M, Templeton JA, Thabane L, Papaioannou A, Giangregorio LM: Exercise for improving outcomes after osteoporotic vertebral fracture. *Cochrane Database of Systematic Reviews* 2019(7). - Gerstein HC, Colhoun HM, Dagenais GR, Diaz R, Lakshmanan M, Pais P, Probstfield J, Riesmeyer JS, Riddle MC, Rydén L: Dulaglutide and cardiovascular outcomes in type 2 diabetes (REWIND): a double-blind, randomised placebo-controlled trial. *The Lancet* 2019, 394(10193):121-130. - Gerstein HC, Colhoun HM, Dagenais GR, Diaz R, Lakshmanan M, Pais P, Probstfield J, Riesmeyer JS, Riddle MC, Rydén L: Dulaglutide and cardiovascular outcomes in type 2 diabetes (REWIND): a double-blind, randomised placebo-controlled trial. *The Lancet* 2019, 394(10193):121-130. - George SMC, Karanovic S, Harrison DA, Rani A, Birnie AJ, Bath‐Hextall FJ, Ravenscroft JC, Williams HC: Interventions to reduce Staphylococcus aureus in the management of eczema. *Cochrane Database of Systematic Reviews* 2019(10). - Geng J, Dong J, Li Y, Ni H, Jiang K, Shi LL, Wang G: Intravenous immunoglobulins for epilepsy. *Cochrane Database of Systematic Reviews* 2019(12). - Gaspar L, Murin J, Oravec S, Bulas J, Caprnda M: Holter ECG findings in diabetics with medial arterial calcification. *Bratisl Lek Listy* 2019, 120(9):676-679. - Gaspar L, Murin J, Oravec S, Bulas J, Caprnda M: Holter ECG findings in diabetics with medial arterial calcification. *Bratisl Lek Listy* 2019, 120(9):676-679. - Gartlehner G, Nussbaumer‐Streit B, Gaynes BN, Forneris CA, Morgan LC, Greenblatt A, Wipplinger J, Lux LJ, Van Noord MG, Winkler D: Second‐generation antidepressants for preventing seasonal affective disorder in adults. *Cochrane Database of Systematic Reviews* 2019(3). - Gana JC, Cifuentes LI, Gattini D, Villarroel del Pino LA, Peña A, Torres-Robles R: Band ligation versus beta‐blockers for primary prophylaxis of oesophageal variceal bleeding in children with chronic liver disease or portal vein thrombosis. *Cochrane Database of Systematic Reviews* 2019(9). - Gan L-M, Lagerström-Fermér M, Carlsson LG, Arfvidsson C, Egnell A-C, Rudvik A, Kjaer M, Collén A, Thompson JD, Joyal J: Intradermal delivery of modified mRNA encoding VEGF-A in patients with type 2 diabetes. *Nature communications* 2019, 10(1):871. - Gan L-M, Lagerström-Fermér M, Carlsson LG, Arfvidsson C, Egnell A-C, Rudvik A, Kjaer M, Collén A, Thompson JD, Joyal J: Intradermal delivery of modified mRNA encoding VEGF-A in patients with type 2 diabetes. *Nature communications* 2019, 10(1):1-9. - Galvin IM, Levy R, Day AG, Gilron I: Pharmacological interventions for the prevention of acute postoperative pain in adults following brain surgery. *Cochrane Database of Systematic Reviews* 2019(11). - Gallagher M, Jones DJ, Bell‐Syer SV: Prophylactic antibiotics to prevent surgical site infection after breast cancer surgery. *Cochrane Database of Systematic Reviews* 2019(9). - Fuentes Padilla P, Martínez G, Vernooij RWM, Urrútia G, Roqué i Figuls M, Bonfill Cosp X: Early enteral nutrition (within 48 hours) versus delayed enteral nutrition (after 48 hours) with or without supplemental parenteral nutrition in critically ill adults. *Cochrane Database of Systematic Reviews* 2019(10). - Franco JVA, Turk T, Jung JH, Xiao YT, Iakhno S, Tirapegui FI, Garrote V, Vietto V: Pharmacological interventions for treating chronic prostatitis/chronic pelvic pain syndrome. *Cochrane Database of Systematic Reviews* 2019(10). - Forneris CA, Nussbaumer‐Streit B, Morgan LC, Greenblatt A, Van Noord MG, Gaynes BN, Wipplinger J, Lux LJ, Winkler D, Gartlehner G: Psychological therapies for preventing seasonal affective disorder. *Cochrane Database of Systematic Reviews* 2019(5). - Forget P, Borovac JA, Thackeray EM, Pace NL: Transient neurological symptoms (TNS) following spinal anaesthesia with lidocaine versus other local anaesthetics in adult surgical patients: a network meta‐analysis. *Cochrane Database of Systematic Reviews* 2019(12). - Foo VHX, Htoon HM, Welsbie DS, Perera SA: Aqueous shunts with mitomycin C versus aqueous shunts alone for glaucoma. *Cochrane Database of Systematic Reviews* 2019(4). - Fontes LES, Martimbianco ALC, Zanin C, Riera R: N‐acetylcysteine as an adjuvant therapy for Helicobacter pylori eradication. *Cochrane Database of Systematic Reviews* 2019(2). - Flodgren G, O'Brien MA, Parmelli E, Grimshaw JM: Local opinion leaders: effects on professional practice and healthcare outcomes. *Cochrane Database of Systematic Reviews* 2019(6). - Fisher SA, Cutler A, Doree C, Brunskill SJ, Stanworth SJ, Navarrete C, Girdlestone J: Mesenchymal stromal cells as treatment or prophylaxis for acute or chronic graft‐versus‐host disease in haematopoietic stem cell transplant (HSCT) recipients with a haematological condition. *Cochrane Database of Systematic Reviews* 2019(1). - Fisher E, Law E, Dudeney J, Eccleston C, Palermo TM: Psychological therapies (remotely delivered) for the management of chronic and recurrent pain in children and adolescents. *Cochrane Database of Systematic Reviews* 2019(4). - Finn J, Jacobs I, Williams TA, Gates S, Perkins GD: Adrenaline and vasopressin for cardiac arrest. *Cochrane Database of Systematic Reviews* 2019(1). - Felder S, Rasmussen MS, King R, Sklow B, Kwaan M, Madoff R, Jensen C: Prolonged thromboprophylaxis with low molecular weight heparin for abdominal or pelvic surgery. *Cochrane Database of Systematic Reviews* 2019(8). - Esu EB, Effa EE, Opie ON, Meremikwu MM: Artemether for severe malaria. *Cochrane Database of Systematic Reviews* 2019(6). - Elsner B, Kugler J, Pohl M, Mehrholz J: Transcranial direct current stimulation (tDCS) for improving aphasia in adults with aphasia after stroke. *Cochrane Database of Systematic Reviews* 2019(5). - El‐Rabbany M, Duchnay M, Raziee HR, Zych M, Tenenbaum H, Shah PS, Azarpazhooh A: Interventions for preventing osteoradionecrosis of the jaws in adults receiving head and neck radiotherapy. *Cochrane Database of Systematic Reviews* 2019(11). - Eachempati P, Kumbargere Nagraj S, Kiran Kumar Krishanappa S, George RP, Soe HH, Karanth L: Management of gag reflex for patients undergoing dental treatment. *Cochrane Database of Systematic Reviews* 2019(11). - Dushianthan A, Cusack R, Burgess VA, Grocott MPW, Calder PC: Immunonutrition for acute respiratory distress syndrome (ARDS) in adults. *Cochrane Database of Systematic Reviews* 2019(1). - Durak HY: Investigation of nomophobia and smartphone addiction predictors among adolescents in Turkey: Demographic variables and academic performance. *The Social Science Journal* 2019, 56(4):492-517. - Dupire G, Droitcourt C, Hughes C, Le Cleach L: Antistreptococcal interventions for guttate and chronic plaque psoriasis. *Cochrane Database of Systematic Reviews* 2019(3). - Dunlop JL, Vandal AC, Marshall MR: Low dialysate sodium levels for chronic haemodialysis. *Cochrane Database of Systematic Reviews* 2019(1). - Duby J, Lassi ZS, Bhutta ZA: Community‐based antibiotic delivery for possible serious bacterial infections in neonates in low‐ and middle‐income countries. *Cochrane Database of Systematic Reviews* 2019(4). - Drevin L, Kim JD: IT STUDENTS’AWARENESS OF THE NEGATIVE EFFECTS OF TECHNOLOGY. *Institute of Science and Technology Education College of Graduate Studies University of South Africa PO Box* 2019, 329. - Dinnes J, Ferrante di Ruffano L, Takwoingi Y, Cheung ST, Nathan P, Matin RN, Chuchu N, Chan SA, Durack A, Bayliss SE *et al*: Ultrasound, CT, MRI, or PET‐CT for staging and re‐staging of adults with cutaneous melanoma. *Cochrane Database of Systematic Reviews* 2019(7). - Dierselhuis EF, Goulding KA, Stevens M, Jutte PC: Intralesional treatment versus wide resection for central low‐grade chondrosarcoma of the long bones. *Cochrane Database of Systematic Reviews* 2019(3). - Dharmadhikari SP, Harshe SD, Bhide PP: Prevalence and correlates of excessive smartphone use among medical students: A cross-sectional study. *Indian journal of psychological medicine* 2019, 41(6):549-555. - Derry S, Bell RF, Straube S, Wiffen PJ, Aldington D, Moore RA: Pregabalin for neuropathic pain in adults. *Cochrane Database of Systematic Reviews* 2019(1). - Denissen S, Staring W, Kunkel D, Pickering RM, Lennon S, Geurts ACH, Weerdesteyn V, Verheyden G: Interventions for preventing falls in people after stroke. *Cochrane Database of Systematic Reviews* 2019(10). - de Paiva CS, Pflugfelder SC, Ng SM, Akpek EK: Topical cyclosporine A therapy for dry eye syndrome. *Cochrane Database of Systematic Reviews* 2019(9). - de Heer K, Gerritsen MG, Visser CE, Leeflang MMG: Galactomannan detection in broncho‐alveolar lavage fluid for invasive aspergillosis in immunocompromised patients. *Cochrane Database of Systematic Reviews* 2019(5). - Day PF, Duggal M, Nazzal H: Interventions for treating traumatised permanent front teeth: avulsed (knocked out) and replanted. *Cochrane Database of Systematic Reviews* 2019(2). - Davies P, Ijaz S, Williams CJ, Kessler D, Lewis G, Wiles N: Pharmacological interventions for treatment‐resistant depression in adults. *Cochrane Database of Systematic Reviews* 2019(12). - Cruciani M, Mengoli C, Barnes R, Donnelly JP, Loeffler J, Jones BL, Klingspor L, Maertens J, Morton CO, White LP: Polymerase chain reaction blood tests for the diagnosis of invasive aspergillosis in immunocompromised people. *Cochrane Database of Systematic Reviews* 2019(9). - Cooper TE, Hambleton IR, Ballas SK, Cashmore BA, Wiffen PJ: Pharmacological interventions for painful sickle cell vaso‐occlusive crises in adults. *Cochrane Database of Systematic Reviews* 2019(11). - Christoffers WA, Coenraads PJ, Svensson Å, Diepgen TL, Dickinson‐Blok JL, Xia J, Williams HC: Interventions for hand eczema. *Cochrane Database of Systematic Reviews* 2019(4). - Chien WT, Clifton AV, Zhao S, Lui S: Peer support for people with schizophrenia or other serious mental illness. *Cochrane Database of Systematic Reviews* 2019(4). - Chen MF, Kim CH, Coleman AL: Cyclodestructive procedures for refractory glaucoma. *Cochrane Database of Systematic Reviews* 2019(3). - Cassidy S, Vaidya V, Houghton D, Zalewski P, Seferovic JP, Hallsworth K, MacGowan GA, Trenell MI, Jakovljevic DG: Unsupervised high-intensity interval training improves glycaemic control but not cardiovascular autonomic function in type 2 diabetes patients: A randomised controlled trial. *Diabetes and Vascular Disease Research* 2019, 16(1):69-76. - Cassidy S, Vaidya V, Houghton D, Zalewski P, Seferovic JP, Hallsworth K, MacGowan GA, Trenell MI, Jakovljevic DG: Unsupervised high-intensity interval training improves glycaemic control but not cardiovascular autonomic function in type 2 diabetes patients: A randomised controlled trial. *Diabetes and Vascular Disease Research* 2019, 16(1):69-76. - Butterworth JE, Hays R, McDonagh STJ, Richards SH, Bower P, Campbell J: Interventions for involving older patients with multi‐morbidity in decision‐making during primary care consultations. *Cochrane Database of Systematic Reviews* 2019(10). - Butler M, Schultz TJ, Halligan P, Sheridan A, Kinsman L, Rotter T, Beaumier J, Kelly RG, Drennan J: Hospital nurse‐staffing models and patient‐ and staff‐related outcomes. *Cochrane Database of Systematic Reviews* 2019(4). - Burton MJ, Pollard AJ, Ramsden JD, Chong LY, Venekamp RP: Tonsillectomy for periodic fever, aphthous stomatitis, pharyngitis and cervical adenitis syndrome (PFAPA). *Cochrane Database of Systematic Reviews* 2019(12). - Burry L, Hutton B, Williamson DR, Mehta S, Adhikari NKJ, Cheng W, Ely EW, Egerod I, Fergusson DA, Rose L: Pharmacological interventions for the treatment of delirium in critically ill adults. *Cochrane Database of Systematic Reviews* 2019(9). - Burden S, Jones DJ, Sremanakova J, Sowerbutts AM, Lal S, Pilling M, Todd C: Dietary interventions for adult cancer survivors. *Cochrane Database of Systematic Reviews* 2019(11). - Burden S, Billson HA, Lal S, Owen KA, Muneer A: Perioperative nutrition for the treatment of bladder cancer by radical cystectomy. *Cochrane Database of Systematic Reviews* 2019(5). - Buitrago‐Garcia D, Martí‐Carvajal AJ, Jimenez A, Conterno LO, Pardo R: Antibiotic therapy for adults with neurosyphilis. *Cochrane Database of Systematic Reviews* 2019(5). - Brown JVE, Walton N, Meader N, Todd A, Webster LAD, Steele R, Sampson SJ, Churchill R, McMillan D, Gilbody S *et al*: Pharmacy‐based management for depression in adults. *Cochrane Database of Systematic Reviews* 2019(12). - Bresnahan R, Martin‐McGill KJ, Williamson J, Michael BD, Marson AG: Clobazam add‐on therapy for drug‐resistant epilepsy. *Cochrane Database of Systematic Reviews* 2019(10). - Bresnahan R, Martin‐McGill KJ, Hutton JL, Marson AG: Tiagabine add‐on therapy for drug‐resistant focal epilepsy. *Cochrane Database of Systematic Reviews* 2019(10). - Bresnahan R, Hounsome J, Jette N, Hutton JL, Marson AG: Topiramate add‐on therapy for drug‐resistant focal epilepsy. *Cochrane Database of Systematic Reviews* 2019(10). - Breilmann J, Girlanda F, Guaiana G, Barbui C, Cipriani A, Castellazzi M, Bighelli I, Davies SJC, Furukawa TA, Koesters M: Benzodiazepines versus placebo for panic disorder in adults. *Cochrane Database of Systematic Reviews* 2019(3). - Braun C, McRobert CJ: Conservative management following closed reduction of traumatic anterior dislocation of the shoulder. *Cochrane Database of Systematic Reviews* 2019(5). - Brand M, Grieve A: Prophylactic antibiotics for penetrating abdominal trauma. *Cochrane Database of Systematic Reviews* 2019(12). - Braganza Menezes D, Menezes B, Dedicoat M: Contact tracing strategies in household and congregate environments to identify cases of tuberculosis in low‐ and moderate‐incidence populations. *Cochrane Database of Systematic Reviews* 2019(8). - Boncoraglio GB, Ranieri M, Bersano A, Parati EA, Del Giovane C: Stem cell transplantation for ischemic stroke. *Cochrane Database of Systematic Reviews* 2019(5). - Bohren MA, Berger BO, Munthe‐Kaas H, Tunçalp Ö: Perceptions and experiences of labour companionship: a qualitative evidence synthesis. *Cochrane Database of Systematic Reviews* 2019(3). - Bofill Rodriguez M, Lethaby A, Grigore M, Brown J, Hickey M, Farquhar C: Endometrial resection and ablation techniques for heavy menstrual bleeding. *Cochrane Database of Systematic Reviews* 2019(1). - Boele FW, Rooney AG, Bulbeck H, Sherwood P: Interventions to help support caregivers of people with a brain or spinal cord tumour. *Cochrane Database of Systematic Reviews* 2019(7). - Blumetti FC, Belloti JC, Tamaoki MJS, Pinto JA: Botulinum toxin type A in the treatment of lower limb spasticity in children with cerebral palsy. *Cochrane Database of Systematic Reviews* 2019(10). - Blessberger H, Lewis SR, Pritchard MW, Fawcett LJ, Domanovits H, Schlager O, Wildner B, Kammler J, Steinwender C: Perioperative beta‐blockers for preventing surgery‐related mortality and morbidity in adults undergoing cardiac surgery. *Cochrane Database of Systematic Reviews* 2019(9). - Blessberger H, Lewis SR, Pritchard MW, Fawcett LJ, Domanovits H, Schlager O, Wildner B, Kammler J, Steinwender C: Perioperative beta‐blockers for preventing surgery‐related mortality and morbidity in adults undergoing non‐cardiac surgery. *Cochrane Database of Systematic Reviews* 2019(9). - Bjerrum S, Schiller I, Dendukuri N, Kohli M, Nathavitharana RR, Zwerling AA, Denkinger CM, Steingart KR, Shah M: Lateral flow urine lipoarabinomannan assay for detecting active tuberculosis in people living with HIV. *Cochrane Database of Systematic Reviews* 2019(10). - Bizino MB, Jazet IM, Westenberg JJ, van Eyk HJ, Paiman EH, Smit JW, Lamb HJ: Effect of liraglutide on cardiac function in patients with type 2 diabetes mellitus: randomized placebo-controlled trial. *Cardiovascular diabetology* 2019, 18:1-12. - Bizino MB, Jazet IM, Westenberg JJ, van Eyk HJ, Paiman EH, Smit JW, Lamb HJ: Effect of liraglutide on cardiac function in patients with type 2 diabetes mellitus: randomized placebo-controlled trial. *Cardiovascular diabetology* 2019, 18(1):1-12. - Bhuyan AK, Baro A, Sarma D, Choudhury B: A study of cardiac autonomic neuropathy in patients with type 2 diabetes mellitus: A Northeast India experience. *Indian journal of endocrinology and metabolism* 2019, 23(2):246-250. - Bhuyan AK, Baro A, Sarma D, Choudhury B: A study of cardiac autonomic neuropathy in patients with type 2 diabetes mellitus: a Northeast India experience. *Indian journal of endocrinology and metabolism* 2019, 23(2):246. - Bhaumik S, Kirubakaran R, Chaudhuri S: ​Primary closure versus delayed or no closure for traumatic wounds due to mammalian bite. *Cochrane Database of Systematic Reviews* 2019(12). - Berbenetz N, Wang Y, Brown J, Godfrey C, Ahmad M, Vital FMR, Lambiase P, Banerjee A, Bakhai A, Chong M: Non‐invasive positive pressure ventilation (CPAP or bilevel NPPV) for cardiogenic pulmonary oedema. *Cochrane Database of Systematic Reviews* 2019(4). - Benson PE, Parkin N, Dyer F, Millett DT, Germain P: Fluorides for preventing early tooth decay (demineralised lesions) during fixed brace treatment. *Cochrane Database of Systematic Reviews* 2019(11). - Belsher BE, Beech E, Evatt D, Smolenski DJ, Shea MT, Otto JL, Rosen CS, Schnurr PP: Present‐centered therapy (PCT) for post‐traumatic stress disorder (PTSD) in adults. *Cochrane Database of Systematic Reviews* 2019(11). - Beishon LC, Batterham AP, Quinn TJ, Nelson CP, Panerai RB, Robinson T, Haunton VJ: Addenbrooke’s Cognitive Examination III (ACE‐III) and mini‐ACE for the detection of dementia and mild cognitive impairment. *Cochrane Database of Systematic Reviews* 2019(12). - Bashier A, Hussain AB, Abdelgadir E, Alawadi F, Sabbour H, Chilton R: Consensus recommendations for management of patients with type 2 diabetes mellitus and cardiovascular diseases. *Diabetology & metabolic syndrome* 2019, 11(1):1-28. - Bashier A, Bin Hussain A, Abdelgadir E, Alawadi F, Sabbour H, Chilton R: Consensus recommendations for management of patients with type 2 diabetes mellitus and cardiovascular diseases. *Diabetology & metabolic syndrome* 2019, 11:1-28. - Barry T, Doheny MC, Masterson S, Conroy N, Klimas J, Segurado R, Codd M, Bury G: Community first responders for out‐of‐hospital cardiac arrest in adults and children. *Cochrane Database of Systematic Reviews* 2019(7). - Barreto L, Jung JH, Abdelrahim A, Ahmed M, Dawkins GPC, Kazmierski M: Medical and surgical interventions for the treatment of urinary stones in children. *Cochrane Database of Systematic Reviews* 2019(10). - Barnes H, Yeoh HL, Fothergill T, Burns A, Humbert M, Williams T: Prostacyclin for pulmonary arterial hypertension. *Cochrane Database of Systematic Reviews* 2019(5). - Barnes H, Brown Z, Burns A, Williams T: Phosphodiesterase 5 inhibitors for pulmonary hypertension. *Cochrane Database of Systematic Reviews* 2019(1). - Bamat N, Fierro J, Wang Y, Millar D, Kirpalani H: Positive end‐expiratory pressure for preterm infants requiring conventional mechanical ventilation for respiratory distress syndrome or bronchopulmonary dysplasia. *Cochrane Database of Systematic Reviews* 2019(2). - Bala MM, Riemsma RP, Wolff R, Pedziwiatr M, Mitus JW, Storman D, Swierz MJ, Kleijnen J: Cryotherapy for liver metastases. *Cochrane Database of Systematic Reviews* 2019(7). - Ba DM, Ssentongo P, Kjerulff KH, Na M, Liu G, Gao X, Du P: Adherence to iron supplementation in 22 sub-Saharan African countries and associated factors among pregnant women: a large population-based study. *Current developments in nutrition* 2019, 3(12):nzz120. - Azirar S, Appelen D, Prins MH, Neumann M, de Feiter ANP, Kolbach DN: Compression therapy for treating post‐thrombotic syndrome. *Cochrane Database of Systematic Reviews* 2019(9). - Axelsson I, Naumburg E, Prietsch SOM, Zhang L: Inhaled corticosteroids in children with persistent asthma: effects of different drugs and delivery devices on growth. *Cochrane Database of Systematic Reviews* 2019(6). - Assalman I, Ahmed A, Alhajjar R, Bewley AP, Taylor R: Treatments for primary delusional infestation. *Cochrane Database of Systematic Reviews* 2019(12). - Asnani MR, Francis DK, Brandow AM, Hammond Gabbadon CEO, Ali A: Interventions for treating neuropathic pain in people with sickle cell disease. *Cochrane Database of Systematic Reviews* 2019(7). - Arts S, Boogaarts HD, van Lindert EJ: Route of antibiotic prophylaxis for prevention of cerebrospinal fluid‐shunt infection. *Cochrane Database of Systematic Reviews* 2019(6). - Armstrong S, Bhide P, Jordan V, Pacey A, Marjoribanks J, Farquhar C: Time‐lapse systems for embryo incubation and assessment in assisted reproduction. *Cochrane Database of Systematic Reviews* 2019(5). - Antequera Martín AM, Barea Mendoza JA, Muriel A, Sáez I, Chico‐Fernández M, Estrada‐Lorenzo JM, Plana MN: Buffered solutions versus 0.9% saline for resuscitation in critically ill adults and children. *Cochrane Database of Systematic Reviews* 2019(7). - Ansari SH, Lassi ZS, Khowaja SM, Adil SO, Shamsi TS: Hydroxyurea (hydroxycarbamide) for transfusion‐dependent β‐thalassaemia. *Cochrane Database of Systematic Reviews* 2019(3). - Annane D, Bellissant E, Bollaert PE, Briegel J, Keh D, Kupfer Y, Pirracchio R, Rochwerg B: Corticosteroids for treating sepsis in children and adults. *Cochrane Database of Systematic Reviews* 2019(12). - Aminian A, Zajichek A, Arterburn DE, Wolski KE, Brethauer SA, Schauer PR, Kattan MW, Nissen SE: Association of metabolic surgery with major adverse cardiovascular outcomes in patients with type 2 diabetes and obesity. *Jama* 2019, 322(13):1271-1282. - Aminian A, Zajichek A, Arterburn DE, Wolski KE, Brethauer SA, Schauer PR, Kattan MW, Nissen SE: Association of metabolic surgery with major adverse cardiovascular outcomes in patients with type 2 diabetes and obesity. *Jama* 2019, 322(13):1271-1282. - Ames HMR, Glenton C, Lewin S, Tamrat T, Akama E, Leon N: Clients’ perceptions and experiences of targeted digital communication accessible via mobile devices for reproductive, maternal, newborn, child, and adolescent health: a qualitative evidence synthesis. *Cochrane Database of Systematic Reviews* 2019(10). - Amatya B, Khan F, Galea M: Rehabilitation for people with multiple sclerosis: an overview of Cochrane Reviews. *Cochrane Database of Systematic Reviews* 2019(1). - Altmann ES, Crossingham I, Wilson S, Davies HR: Intra‐pleural fibrinolytic therapy versus placebo, or a different fibrinolytic agent, in the treatment of adult parapneumonic effusions and empyema. *Cochrane Database of Systematic Reviews* 2019(10). - Alsalameh AM, Harisi MJ, Alduayji MA, Almutham AA, Mahmood FM: Evaluating the relationship between smartphone addiction/overuse and musculoskeletal pain among medical students at Qassim University. *Journal of family medicine and primary care* 2019, 8(9):2953-2959. - Alfirevic A, Pirmohamed M, Marinovic B, Harcourt‐Smith L, Jorgensen AL, Cooper TE: Genetic testing for prevention of severe drug‐induced skin rash. *Cochrane Database of Systematic Reviews* 2019(7). - Alexander CE, Scullion MMF, Omar MI, Yuan Y, Mamoulakis C, N'Dow JMO, Chen C, Lam TBL: Bipolar versus monopolar transurethral resection of the prostate for lower urinary tract symptoms secondary to benign prostatic obstruction. *Cochrane Database of Systematic Reviews* 2019(12). - Albursan IS, Al Qudah MF, Dutton E, Hassan EMAH, Bakhiet SFA, Alfnan AA, Aljomaa SS, Hammad HI: National, sex and academic discipline difference in smartphone addiction: A study of students in Jordan, Saudi Arabia, Yemen and Sudan. *Community Mental Health Journal* 2019, 55:825-830. - Ahmad MI, Mujtaba M, Anees MA, Li Y, Soliman EZ: Interrelation between electrocardiographic left atrial abnormality, left ventricular hypertrophy, and mortality in participants with hypertension. *The American Journal of Cardiology* 2019, 124(6):886-891. - Ahmad G, Baker J, Finnerty J, Phillips K, Watson A: Laparoscopic entry techniques. *Cochrane Database of Systematic Reviews* 2019(1). - Agnihotry A, Thompson W, Fedorowicz Z, van Zuuren EJ, Sprakel J: Antibiotic use for irreversible pulpitis. *Cochrane Database of Systematic Reviews* 2019(5). - Abdul Wahid SF, Law ZK, Ismail NA, Lai NM: Cell‐based therapies for amyotrophic lateral sclerosis/motor neuron disease. *Cochrane Database of Systematic Reviews* 2019(12). - Zonneveld TP, Richard E, Vergouwen MDI, Nederkoorn PJ, de Haan RJ, Roos Y, Kruyt ND: Blood pressure‐lowering treatment for preventing recurrent stroke, major vascular events, and dementia in patients with a history of stroke or transient ischaemic attack. *Cochrane Database of Systematic Reviews* 2018(7). - Zhao W, Zhang J, Sadowsky MG, Meng R, Ding Y, Ji X: Remote ischaemic conditioning for preventing and treating ischaemic stroke. *Cochrane Database of Systematic Reviews* 2018(7). - Zaugg V, Korb‐Savoldelli V, Durieux P, Sabatier B: Providing physicians with feedback on medication adherence for people with chronic diseases taking long‐term medication. *Cochrane Database of Systematic Reviews* 2018(1). - Zalmanovici Trestioreanu A, Barua A, Pertzov B: Cyclamen europaeum extract for acute sinusitis. *Cochrane Database of Systematic Reviews* 2018(5). - Yu Y, Zhang K, Zhang L, Zong H, Meng L, Han R: Cerebral near‐infrared spectroscopy (NIRS) for perioperative monitoring of brain oxygenation in children and adults. *Cochrane Database of Systematic Reviews* 2018(1). - Yu A, Wu S, Zhang Z, Dening T, Zhao S, Pinner G, Xia J, Yang D: Cholinesterase inhibitors for the treatment of delirium in non‐ICU settings. *Cochrane Database of Systematic Reviews* 2018(6). - 1439. Ye J, Deng G, Gao F: Theoretical overview of clinical and pharmacological aspects of the use of etelcalcetide in diabetic patients undergoing hemodialysis. *Drug design, development and therapy* 2018, 12:901-909. - Yamamoto S, Hotta K, Ota E, Matsunaga A, Mori R: Exercise‐based cardiac rehabilitation for people with implantable ventricular assist devices. *Cochrane Database of Systematic Reviews* 2018(9). - Yadav D, Sharma S, Sharma L, Kanwar S: Smartphone usage and attitude among medical students as a new learning aid in medical education in northwest India: a questionnaire based study. *addiction* 2018, 11:12. - Xu M, Li D, Zhang S: Acupuncture for acute stroke. *Cochrane Database of Systematic Reviews* 2018(3). - Xiong Y, Tao J, Cai L, Tang Y, Li Q: The role of Enterobacteria, TNF-α, IL-6, and IL-10 in the development of myocardial ischemia with type 2 diabetes mellitus. *European journal of inflammation* 2018, 16:205873921879232. - Wu P-Y, Huang J-C, Chen S-C, Chen L-I: Type 2 diabetes mellitus-related changes in left ventricular structure and function in patients with chronic kidney disease. *Oncotarget* 2018, 9(18):14661-14668. - Wright JM, Musini VM, Gill R: First‐line drugs for hypertension. *Cochrane Database of Systematic Reviews* 2018(4). - Williams MA, Srikesavan C, Heine PJ, Bruce J, Brosseau L, Hoxey‐Thomas N, Lamb SE: Exercise for rheumatoid arthritis of the hand. *Cochrane Database of Systematic Reviews* 2018(7). - Wennmacker SZ, Lamberts MP, Di Martino M, Drenth JPH, Gurusamy KS, van Laarhoven C: Transabdominal ultrasound and endoscopic ultrasound for diagnosis of gallbladder polyps. *Cochrane Database of Systematic Reviews* 2018(8). - Welsch P, Üçeyler N, Klose P, Walitt B, Häuser W: Serotonin and noradrenaline reuptake inhibitors (SNRIs) for fibromyalgia. *Cochrane Database of Systematic Reviews* 2018(2). - Welsch P, Bernardy K, Derry S, Moore RA, Häuser W: Mirtazapine for fibromyalgia in adults. *Cochrane Database of Systematic Reviews* 2018(8). - Weibel S, Jelting Y, Pace NL, Helf A, Eberhart LHJ, Hahnenkamp K, Hollmann MW, Poepping DM, Schnabel A, Kranke P: Continuous intravenous perioperative lidocaine infusion for postoperative pain and recovery in adults. *Cochrane Database of Systematic Reviews* 2018(6). - Wei D, Heus P, van de Wetering FT, van Tienhoven G, Verleye L, Scholten R: Probiotics for the prevention or treatment of chemotherapy‐ or radiotherapy‐related diarrhoea in people with cancer. *Cochrane Database of Systematic Reviews* 2018(8). - Wegner I, Hall DA, Smit AL, McFerran D, Stegeman I: Betahistine for tinnitus. *Cochrane Database of Systematic Reviews* 2018(12). - Warttig S, Alderson P, Evans DJW, Lewis SR, Kourbeti IS, Smith AF: Automated monitoring compared to standard care for the early detection of sepsis in critically ill patients. *Cochrane Database of Systematic Reviews* 2018(6). - Wang Y, Yang H, Huynh Q, Nolan M, Negishi K, Marwick TH: Diagnosis of nonischemic stage B heart failure in type 2 diabetes mellitus: optimal parameters for prediction of heart failure. *JACC: Cardiovascular Imaging* 2018, 11(10):1390-1400. - Wang Y, Yang H, Huynh Q, Nolan M, Negishi K, Marwick TH: Diagnosis of nonischemic stage B heart failure in type 2 diabetes mellitus: optimal parameters for prediction of heart failure. *JACC: Cardiovascular Imaging* 2018, 11(10):1390-1400. - Wang PL, Brooks SC: Mechanical versus manual chest compressions for cardiac arrest. *Cochrane Database of Systematic Reviews* 2018(8). - Wang H, Li L, Qin LL, Song Y, Vidal‐Alaball J, Liu TH: Oral vitamin B12 versus intramuscular vitamin B12 for vitamin B12 deficiency. *Cochrane Database of Systematic Reviews* 2018(3). - Walters JAE, Tan DJ, White CJ, Wood‐Baker R: Different durations of corticosteroid therapy for exacerbations of chronic obstructive pulmonary disease. *Cochrane Database of Systematic Reviews* 2018(3). - Wall ECB, Ajdukiewicz KMB, Bergman H, Heyderman RS, Garner P: Osmotic therapies added to antibiotics for acute bacterial meningitis. *Cochrane Database of Systematic Reviews* 2018(2). - Walker KF, Kibuka M, Thornton JG, Jones NW: Maternal position in the second stage of labour for women with epidural anaesthesia. *Cochrane Database of Systematic Reviews* 2018(11). - Wade RG, Wormald JCR, Figus A: Absorbable versus non‐absorbable sutures for skin closure after carpal tunnel decompression surgery. *Cochrane Database of Systematic Reviews* 2018(2). - Vollenweider DJ, Frei A, Steurer‐Stey CA, Garcia‐Aymerich J, Puhan MA: Antibiotics for exacerbations of chronic obstructive pulmonary disease. *Cochrane Database of Systematic Reviews* 2018(10). - Virgili G, Acosta R, Bentley SA, Giacomelli G, Allcock C, Evans JR: Reading aids for adults with low vision. *Cochrane Database of Systematic Reviews* 2018(4). - Vietto V, Franco JVA, Saenz V, Cytryn D, Chas J, Ciapponi A: Prostanoids for critical limb ischaemia. *Cochrane Database of Systematic Reviews* 2018(1). - Vettoretto N, Arezzo A, Famiglietti F, Cirocchi R, Moja L, Morino M: Laparoscopic‐endoscopic rendezvous versus preoperative endoscopic sphincterotomy in people undergoing laparoscopic cholecystectomy for stones in the gallbladder and bile duct. *Cochrane Database of Systematic Reviews* 2018(4). - Vermeij JD, Westendorp WF, Dippel DWJ, van de Beek D, Nederkoorn PJ: Antibiotic therapy for preventing infections in people with acute stroke. *Cochrane Database of Systematic Reviews* 2018(1). - Vaona A, Banzi R, Kwag KH, Rigon G, Cereda D, Pecoraro V, Tramacere I, Moja L: E‐learning for health professionals. *Cochrane Database of Systematic Reviews* 2018(1). - van den Blink QU, Garcez K, Henson CC, Davidson SE, Higham CE: Pharmacological interventions for the prevention of insufficiency fractures and avascular necrosis associated with pelvic radiotherapy in adults. *Cochrane Database of Systematic Reviews* 2018(4). - Tzelnick S, Alkan U, Leshno M, Hwang P, Soudry E: Sinonasal debridement versus no debridement for the postoperative care of patients undergoing endoscopic sinus surgery. *Cochrane Database of Systematic Reviews* 2018(11). - Turner RR, Steed L, Quirk H, Greasley RU, Saxton JM, Taylor SJC, Rosario DJ, Thaha MA, Bourke L: Interventions for promoting habitual exercise in people living with and beyond cancer. *Cochrane Database of Systematic Reviews* 2018(9). - Tunnicliffe DJ, Palmer SC, Henderson L, Masson P, Craig JC, Tong A, Singh‐Grewal D, Flanc RS, Roberts MA, Webster AC *et al*: Immunosuppressive treatment for proliferative lupus nephritis. *Cochrane Database of Systematic Reviews* 2018(6). - Treweek S, Pitkethly M, Cook J, Fraser C, Mitchell E, Sullivan F, Jackson C, Taskila TK, Gardner H: Strategies to improve recruitment to randomised trials. *Cochrane Database of Systematic Reviews* 2018(2). - Tougouma SJ, Kambiré Y, Bado J, Yaméogo AA, Yaméogo TM, Sidibé S, Kyelem CG, Ilboudo A, Ouédraogo M: [Electrocardiography coupled with transthoracic echocardiography at rest in the diagnosis of cardiac impairments in type 2 diabetics: lessons learned from a cross-sectional case series in Burkina Faso]. *Pan Afr Med J* 2018, 31:169. - Tougouma SJ, Kambiré Y, Bado J, Yaméogo AA, Yaméogo TM, Sidibé S, Kyelem CG, Ilboudo A, Ouédraogo M: [Electrocardiography coupled with transthoracic echocardiography at rest in the diagnosis of cardiac impairments in type 2 diabetics: lessons learned from a cross-sectional case series in Burkina Faso]. *Pan Afr Med J* 2018, 31:169. - Tosello G, Torloni MR, Mota BS, Neeman T, Riera R: Breast surgery for metastatic breast cancer. *Cochrane Database of Systematic Reviews* 2018(3). - Toews I, George AT, Peter JV, Kirubakaran R, Fontes LES, Ezekiel JPB, Meerpohl JJ: Interventions for preventing upper gastrointestinal bleeding in people admitted to intensive care units. *Cochrane Database of Systematic Reviews* 2018(6). - Thorpe J, Shum B, Moore RA, Wiffen PJ, Gilron I: Combination pharmacotherapy for the treatment of fibromyalgia in adults. *Cochrane Database of Systematic Reviews* 2018(2). - Thompson ER, Hosgood SA, Nicholson ML, Wilson CH: Early versus late ureteric stent removal after kidney transplantation. *Cochrane Database of Systematic Reviews* 2018(1). - Thomas RE, Lorenzetti DL: Interventions to increase influenza vaccination rates of those 60 years and older in the community. *Cochrane Database of Systematic Reviews* 2018(5). - The Prophylactic Cranial Irradiation Overview Collaborative G: Cranial irradiation for preventing brain metastases of small cell lung cancer in patients in complete remission. *Cochrane Database of Systematic Reviews* 2018(2). - Thabrew H, Stasiak K, Hetrick SE, Wong S, Huss JH, Merry SN: E‐Health interventions for anxiety and depression in children and adolescents with long‐term physical conditions. *Cochrane Database of Systematic Reviews* 2018(8). - Tenforde MW, Shapiro AE, Rouse B, Jarvis JN, Li T, Eshun‐Wilson I, Ford N: Treatment for HIV‐associated cryptococcal meningitis. *Cochrane Database of Systematic Reviews* 2018(7). - Temmingh HS, Williams T, Siegfried N, Stein DJ: Risperidone versus other antipsychotics for people with severe mental illness and co‐occurring substance misuse. *Cochrane Database of Systematic Reviews* 2018(1). - Tammenmaa‐Aho I, Asher R, Soares‐Weiser K, Bergman H: Cholinergic medication for antipsychotic‐induced tardive dyskinesia. *Cochrane Database of Systematic Reviews* 2018(3). - Tam KW, Wu MY, Siddiqui FJ, Chan ESY, Zhu Y, Jafar TH: Omega‐3 fatty acids for dialysis vascular access outcomes in patients with chronic kidney disease. *Cochrane Database of Systematic Reviews* 2018(11). - Syrmis W, Richard R, Jenkins-Marsh S, Chia SC, Good P: Oral water soluble contrast for malignant bowel obstruction. *Cochrane Database of Systematic Reviews* 2018(3). - Sutherland A, Naessens K, Plugge E, Ware L, Head K, Burton MJ, Wee B: Olanzapine for the prevention and treatment of cancer‐related nausea and vomiting in adults. *Cochrane Database of Systematic Reviews* 2018(9). - Strøm C, Stefansson JS, Fabritius ML, Rasmussen LS, Schmidt TA, Jakobsen JC: Hospitalisation in short‐stay units for adults with internal medicine diseases and conditions. *Cochrane Database of Systematic Reviews* 2018(8). - Strametz R, Bergold MN, Weberschock T: Laryngeal mask airway versus endotracheal tube for percutaneous dilatational tracheostomy in critically ill adults. *Cochrane Database of Systematic Reviews* 2018(11). - Storebø OJ, Pedersen N, Ramstad E, Kielsholm ML, Nielsen SS, Krogh HB, Moreira‐Maia CR, Magnusson FL, Holmskov M, Gerner T *et al*: Methylphenidate for attention deficit hyperactivity disorder (ADHD) in children and adolescents – assessment of adverse events in non‐randomised studies. *Cochrane Database of Systematic Reviews* 2018(5). - Stocco FG, Evaristo E, Shah NR, Cheezum MK, Hainer J, Foster C, Nearing BD, Gervino E, Verrier RL: Marked exercise‐induced T‐wave heterogeneity in symptomatic diabetic patients with nonflow‐limiting coronary artery stenosis. *Annals of noninvasive electrocardiology* 2018, 23(2):e12503-n/a. - Stengel D, Leisterer J, Ferrada P, Ekkernkamp A, Mutze S, Hoenning A: Point‐of‐care ultrasonography for diagnosing thoracoabdominal injuries in patients with blunt trauma. *Cochrane Database of Systematic Reviews* 2018(12). - St George G, Morgan A, Meechan J, Moles DR, Needleman I, Ng YL, Petrie A: Injectable local anaesthetic agents for dental anaesthesia. *Cochrane Database of Systematic Reviews* 2018(7). - Spencer S, Johnson P, Smith IC: De‐escalation techniques for managing non‐psychosis induced aggression in adults. *Cochrane Database of Systematic Reviews* 2018(7). - Spencer S, Felix LM, Milan SJ, Normansell R, Goeminne PC, Chalmers JD, Donovan T: Oral versus inhaled antibiotics for bronchiectasis. *Cochrane Database of Systematic Reviews* 2018(3). - Sowerbutts AM, Lal S, Sremanakova J, Clamp A, Todd C, Jayson GC, Teubner A, Raftery AM, Sutton EJ, Hardy L *et al*: Home parenteral nutrition for people with inoperable malignant bowel obstruction. *Cochrane Database of Systematic Reviews* 2018(8). - Soldani FA, Lamont T, Jones K, Young L, Walsh T, Lala R, Clarkson JE: One‐to‐one oral hygiene advice provided in a dental setting for oral health. *Cochrane Database of Systematic Reviews* 2018(10). - Soares‐Weiser K, Maayan N, Bergman H: Vitamin E for antipsychotic‐induced tardive dyskinesia. *Cochrane Database of Systematic Reviews* 2018(1). - Smith LA, Burns E, Cuthbert A: Parenteral opioids for maternal pain management in labour. *Cochrane Database of Systematic Reviews* 2018(6). - Smith CA, Levett KM, Collins CT, Armour M, Dahlen HG, Suganuma M: Relaxation techniques for pain management in labour. *Cochrane Database of Systematic Reviews* 2018(3). - Sliwka A, Jankowski M, Gross‐Sondej I, Storman M, Nowobilski R, Bala MM: Once‐daily long‐acting beta₂‐agonists/inhaled corticosteroids combined inhalers versus inhaled long‐acting muscarinic antagonists for people with chronic obstructive pulmonary disease. *Cochrane Database of Systematic Reviews* 2018(8). - Sim EHA, Yang IA, Wood‐Baker R, Bowman RV, Fong KM: Gefitinib for advanced non‐small cell lung cancer. *Cochrane Database of Systematic Reviews* 2018(1). - Shetty RM, Bellini A, Wijayatilake DS, Hamilton MA, Jain R, Karanth S, Namachivayam A: BIS monitoring versus clinical assessment for sedation in mechanically ventilated adults in the intensive care unit and its impact on clinical outcomes and resource utilization. *Cochrane Database of Systematic Reviews* 2018(2). - Shakur H, Beaumont D, Pavord S, Gayet‐Ageron A, Ker K, Mousa HA: Antifibrinolytic drugs for treating primary postpartum haemorrhage. *Cochrane Database of Systematic Reviews* 2018(2). - Shah A, Brunskill SJ, Desborough MJR, Doree C, Trivella M, Stanworth SJ: Transfusion of red blood cells stored for shorter versus longer duration for all conditions. *Cochrane Database of Systematic Reviews* 2018(12). - Sethuraman AR, Rao S, Charlette L, Thatkar PV, Vincent V: Smartphone addiction among medical college students in the Andaman and Nicobar Islands. *International Journal of Community Medicine and Public Health* 2018, 5(10):4273-4277. - Sereda M, Xia J, El Refaie A, Hall DA, Hoare DJ: Sound therapy (using amplification devices and/or sound generators) for tinnitus. *Cochrane Database of Systematic Reviews* 2018(12). - Schofield‐Robinson OJ, Lewis SR, Smith AF, McPeake J, Alderson P: Follow‐up services for improving long‐term outcomes in intensive care unit (ICU) survivors. *Cochrane Database of Systematic Reviews* 2018(11). - Sathianathen NJ, Philippou YA, Kuntz GM, Konety BR, Gupta S, Lamb AD, Dahm P: Taxane‐based chemohormonal therapy for metastatic hormone‐sensitive prostate cancer. *Cochrane Database of Systematic Reviews* 2018(10). - Samara MT, Klupp E, Helfer B, Rothe PH, Schneider‐Thoma J, Leucht S: Increasing antipsychotic dose for non response in schizophrenia. *Cochrane Database of Systematic Reviews* 2018(5). - Salicath JH, Yeoh ECY, Bennett MH: Epidural analgesia versus patient‐controlled intravenous analgesia for pain following intra‐abdominal surgery in adults. *Cochrane Database of Systematic Reviews* 2018(8). - Saleem HT, Narasimhan M, Ganatra B, Kennedy CE: Medical and surgical abortion for women living with HIV. *Cochrane Database of Systematic Reviews* 2018(12). - Sachdeva A, Dalton M, Lees T: Graduated compression stockings for prevention of deep vein thrombosis. *Cochrane Database of Systematic Reviews* 2018(11). - Rüschen H, Aravinth K, Bunce C, Bokre D: Use of hyaluronidase as an adjunct to local anaesthetic eye blocks to reduce intraoperative pain in adults. *Cochrane Database of Systematic Reviews* 2018(3). - Ruospo M, Palmer SC, Natale P, Craig JC, Vecchio M, Elder GJ, Strippoli GFM: Phosphate binders for preventing and treating chronic kidney disease‐mineral and bone disorder (CKD‐MBD). *Cochrane Database of Systematic Reviews* 2018(8). - Roth D, Pace NL, Lee A, Hovhannisyan K, Warenits AM, Arrich J, Herkner H: Airway physical examination tests for detection of difficult airway management in apparently normal adult patients. *Cochrane Database of Systematic Reviews* 2018(5). - Rösner S, Englbrecht C, Wehrle R, Hajak G, Soyka M: Eszopiclone for insomnia. *Cochrane Database of Systematic Reviews* 2018(10). - Rodgers JL, Samal E, Mohapatra S, Panguluri SK: Hyperoxia-induced cardiotoxicity and ventricular remodeling in type-II diabetes mice. *Heart Vessels* 2018, 33(5):561-572. - Rodgers JL, Samal E, Mohapatra S, Panguluri SK: Hyperoxia-induced cardiotoxicity and ventricular remodeling in type-II diabetes mice. *Heart Vessels* 2018, 33(5):561-572. - Ren Y, Ren Q, Lu J, Guo X, Huo X, Ji L, Yang X: Low triglyceride as a marker for increased risk of cardiovascular diseases in patients with long‐term type 2 diabetes: a cross‐sectional survey in China. *Diabetes/metabolism research and reviews* 2018, 34(2):e2960. - Ren Y, Ren Q, Lu J, Guo X, Huo X, Ji L, Yang X: Low triglyceride as a marker for increased risk of cardiovascular diseases in patients with long‐term type 2 diabetes: A cross‐sectional survey in China. *Diabetes/metabolism research and reviews* 2018, 34(2):e2960. - Redmond P, Grimes TC, McDonnell R, Boland F, Hughes C, Fahey T: Impact of medication reconciliation for improving transitions of care. *Cochrane Database of Systematic Reviews* 2018(8). - Rahimi‐Movaghar A, Gholami J, Amato L, Hoseinie L, Yousefi‐Nooraie R, Amin‐Esmaeili M: Pharmacological therapies for management of opium withdrawal. *Cochrane Database of Systematic Reviews* 2018(6). - Purgato M, Gastaldon C, Papola D, van Ommeren M, Barbui C, Tol WA: Psychological therapies for the treatment of mental disorders in low‐ and middle‐income countries affected by humanitarian crises. *Cochrane Database of Systematic Reviews* 2018(7). - Punjasawadwong Y, Chau‐in W, Laopaiboon M, Punjasawadwong S, Pin‐on P: Processed electroencephalogram and evoked potential techniques for amelioration of postoperative delirium and cognitive dysfunction following non‐cardiac and non‐neurosurgical procedures in adults. *Cochrane Database of Systematic Reviews* 2018(5). - Porter‐Armstrong AP, Moore ZEH, Bradbury I, McDonough S: Education of healthcare professionals for preventing pressure ulcers. *Cochrane Database of Systematic Reviews* 2018(5). - Pollok J, van Agteren JEM, Carson‐Chahhoud KV: Pharmacological interventions for the treatment of depression in chronic obstructive pulmonary disease. *Cochrane Database of Systematic Reviews* 2018(12). - Polderman JAW, Farhang‐Razi V, Van Dieren S, Kranke P, DeVries JH, Hollmann MW, Preckel B, Hermanides J: Adverse side effects of dexamethasone in surgical patients. *Cochrane Database of Systematic Reviews* 2018(11). - Pillay S, Hift R, Aldous C: A retrospective analysis of electrocardiographic abnormalities found in black South African patients with diabetes attending a regional hospital in KwaZulu-Natal. *JEMDSA : the journal of endocrinology, metabolism and diabetes of South Africa* 2018, 23(1):9-16. - Perry LA, Ramson D, Stricklin S: Mirtazapine adjunct for people with schizophrenia. *Cochrane Database of Systematic Reviews* 2018(5). - Perman MI, Ciapponi A, Franco JVA, Loudet C, Crivelli A, Garrote V, Perman G: Prescribed hypocaloric nutrition support for critically‐ill adults. *Cochrane Database of Systematic Reviews* 2018(6). - Pérez‐Gaxiola G, Cuello‐García CA, Florez ID, Pérez‐Pico VM: Smectite for acute infectious diarrhoea in children. *Cochrane Database of Systematic Reviews* 2018(4). - Payne AGT, Alsabeeha NHM, Atieh MA, Esposito M, Ma S, Anas El‐Wegoud M: Interventions for replacing missing teeth: attachment systems for implant overdentures in edentulous jaws. *Cochrane Database of Systematic Reviews* 2018(10). - Pasquali S, Hadjinicolaou AV, Chiarion Sileni V, Rossi CR, Mocellin S: Systemic treatments for metastatic cutaneous melanoma. *Cochrane Database of Systematic Reviews* 2018(2). - Parker CE, Nguyen TM, Segal D, MacDonald JK, Chande N: Low dose naltrexone for induction of remission in Crohn's disease. *Cochrane Database of Systematic Reviews* 2018(4). - Pandey S, Srivanitchapoom P, Kirubakaran R, Berman BD: Botulinum toxin for motor and phonic tics in Tourette's syndrome. *Cochrane Database of Systematic Reviews* 2018(1). - Othieno R, Okpo E, Forster R: Home versus in‐patient treatment for deep vein thrombosis. *Cochrane Database of Systematic Reviews* 2018(1). - 1538. Ostinelli EG, Hussein M, Ahmed U, Rehman FU, Miramontes K, Adams CE: Risperidone for psychosis‐induced aggression or agitation (rapid tranquillisation). *Cochrane Database of Systematic Reviews* 2018(4). - 1539. O'Connell NE, Marston L, Spencer S, DeSouza LH, Wand BM: Non‐invasive brain stimulation techniques for chronic pain. *Cochrane Database of Systematic Reviews* 2018(4). - Novoa M, Baselga E, Beltran S, Giraldo L, Shahbaz A, Pardo‐Hernandez H, Arevalo‐Rodriguez I: Interventions for infantile haemangiomas of the skin. *Cochrane Database of Systematic Reviews* 2018(4). - Normansell R, Sayer B, Waterson S, Dennett EJ, Del Forno M, Dunleavy A: Antibiotics for exacerbations of asthma. *Cochrane Database of Systematic Reviews* 2018(6). - Norman G, Westby MJ, Rithalia AD, Stubbs N, Soares MO, Dumville JC: Dressings and topical agents for treating venous leg ulcers. *Cochrane Database of Systematic Reviews* 2018(6). - Ngesi N, Landa N, Madikiza N, Cekiso MP, Tshotsho B, Walters LM: Use of mobile phones as supplementary teaching and learning tools to learners in South Africa. *Reading & Writing-Journal of the Reading Association of South Africa* 2018, 9(1):1-12. - Nganou-Gnindjio CN, Mba CM, Azabji-Kenfack M, Dehayem MY, Mfeukeu-Kuate L, Mbanya J-C, Sobngwi E: Poor glycemic control impacts heart rate variability in patients with type 2 diabetes mellitus: a cross sectional study. *BMC Research Notes* 2018, 11:1-4. - Nganou-Gnindjio CN, Mba CM, Azabji-Kenfack M, Dehayem MY, Mfeukeu-Kuate L, Mbanya J-C, Sobngwi E: Poor glycemic control impacts heart rate variability in patients with type 2 diabetes mellitus: a cross sectional study. *BMC research notes* 2018, 11(1):1-4. - Nevitt SJ, Tudur Smith C, Marson AG: Oxcarbazepine versus phenytoin monotherapy for epilepsy: an individual participant data review. *Cochrane Database of Systematic Reviews* 2018(10). - Nevitt SJ, Marson AG, Tudur Smith C: Carbamazepine versus phenobarbitone monotherapy for epilepsy: an individual participant data review. *Cochrane Database of Systematic Reviews* 2018(10). - Nelson SML, Nguyen TM, McDonald JWD, MacDonald JK: Natalizumab for induction of remission in Crohn's disease. *Cochrane Database of Systematic Reviews* 2018(8). - Nayak JK: Relationship among smartphone usage, addiction, academic performance and the moderating role of gender: A study of higher education students in India. *Computers & Education* 2018, 123:164-173. - Narula N, Dhillon A, Zhang D, Sherlock ME, Tondeur M, Zachos M: Enteral nutritional therapy for induction of remission in Crohn's disease. *Cochrane Database of Systematic Reviews* 2018(4). - Narayan V, Kahlmeyer A, Dahm P, Skoetz N, Risk MC, Bongiorno C, Patel N, Hwang EC, Jung JH, Gartlehner G *et al*: Pembrolizumab monotherapy versus chemotherapy for treatment of advanced urothelial carcinoma with disease progression during or following platinum‐containing chemotherapy. A Cochrane Rapid Review. *Cochrane Database of Systematic Reviews* 2018(7). - Nama V, Angelopoulos G, Twigg J, Murdoch JB, Bailey J, Lawrie TA: Type II or type III radical hysterectomy compared to chemoradiotherapy as a primary intervention for stage IB2 cervical cancer. *Cochrane Database of Systematic Reviews* 2018(10). - Naka KK, Papathanassiou K, Bechlioulis A, Pappas K, Tigas S, Makriyiannis D, Antoniou S, Kazakos N, Margeli A, Papassotiriou I: Association of vascular indices with novel circulating biomarkers as prognostic factors for cardiovascular complications in patients with type 2 diabetes mellitus. *Clinical biochemistry* 2018, 53:31-37. - Naka KK, Papathanassiou K, Bechlioulis A, Pappas K, Tigas S, Makriyiannis D, Antoniou S, Kazakos N, Margeli A, Papassotiriou I: Association of vascular indices with novel circulating biomarkers as prognostic factors for cardiovascular complications in patients with type 2 diabetes mellitus. *Clinical biochemistry* 2018, 53:31-37. - Nagler EV, Haller MC, Van Biesen W, Vanholder R, Craig JC, Webster AC: Interventions for chronic non‐hypovolaemic hypotonic hyponatraemia. *Cochrane Database of Systematic Reviews* 2018(6). - Mücke M, Phillips T, Radbruch L, Petzke F, Häuser W: Cannabis‐based medicines for chronic neuropathic pain in adults. *Cochrane Database of Systematic Reviews* 2018(3). - Moutray T, Evans JR, Lois N, Armstrong DJ, Peto T, Azuara‐Blanco A: Different lasers and techniques for proliferative diabetic retinopathy. *Cochrane Database of Systematic Reviews* 2018(3). - Moţăţăianu A, Maier S, Bajko Z, Voidazan S, Bălaşa R, Stoian A: Cardiac autonomic neuropathy in type 1 and type 2 diabetes patients. *BMC neurology* 2018, 18:1-9. - Moţăţăianu A, Maier S, Bajko Z, Voidazan S, Bălaşa R, Stoian A: Cardiac autonomic neuropathy in type 1 and type 2 diabetes patients. *BMC neurology* 2018, **18**(1):1-9. - Morrison J, Thoma C, Goodall RJ, Lyons TJ, Gaitskell K, Wiggans AJ, Bryant A: Epidermal growth factor receptor blockers for the treatment of ovarian cancer. *Cochrane Database of Systematic Reviews* 2018(10). - Morling JR, Yeoh SE, Kolbach DN: Rutosides for prevention of post‐thrombotic syndrome. *Cochrane Database of Systematic Reviews* 2018(11). - Morling JR, Broderick C, Yeoh SE, Kolbach DN: Rutosides for treatment of post‐thrombotic syndrome. *Cochrane Database of Systematic Reviews* 2018(11). - Morgan AT, Murray E, Liégeois FJ: Interventions for childhood apraxia of speech. *Cochrane Database of Systematic Reviews* 2018(5). - Moreno DH, Cacione DG, Baptista‐Silva JCC: Controlled hypotension versus normotensive resuscitation strategy for people with ruptured abdominal aortic aneurysm. *Cochrane Database of Systematic Reviews* 2018(6). - Moralejo D, El Dib R, Prata RA, Barretti P, Corrêa I: Improving adherence to Standard Precautions for the control of health care‐associated infections. *Cochrane Database of Systematic Reviews* 2018(2). - Moore ZEH, Webster J: Dressings and topical agents for preventing pressure ulcers. *Cochrane Database of Systematic Reviews* 2018(12). - Moore PM, Rivera S, Bravo‐Soto GA, Olivares C, Lawrie TA: Communication skills training for healthcare professionals working with people who have cancer. *Cochrane Database of Systematic Reviews* 2018(7). - Montero N, Favà A, Rodriguez E, Barrios C, Cruzado JM, Pascual J, Soler MJ: Treatment for hepatitis C virus‐associated mixed cryoglobulinaemia. *Cochrane Database of Systematic Reviews* 2018(5). - Mohammed Vashist N, Samaan M, Mosli MH, Parker CE, MacDonald JK, Nelson SA, Zou GY, Feagan BG, Khanna R, Jairath V: Endoscopic scoring indices for evaluation of disease activity in ulcerative colitis. *Cochrane Database of Systematic Reviews* 2018(1). - Milligan R, Paul M, Richardson M, Neuberger A: Vaccines for preventing typhoid fever. *Cochrane Database of Systematic Reviews* 2018(5). - Miller D, Lewis SR, Pritchard MW, Schofield‐Robinson OJ, Shelton CL, Alderson P, Smith AF: Intravenous versus inhalational maintenance of anaesthesia for postoperative cognitive outcomes in elderly people undergoing non‐cardiac surgery. *Cochrane Database of Systematic Reviews* 2018(8). - Metcalfe D, Rios Diaz AJ, Olufajo OA, Massa MS, Ketelaar N, Flottorp SA, Perry DC: Impact of public release of performance data on the behaviour of healthcare consumers and providers. *Cochrane Database of Systematic Reviews* 2018(9). - Mehta H, Hennings C, Gillies MC, Nguyen V, Campain A, Fraser‐Bell S: Anti‐vascular endothelial growth factor combined with intravitreal steroids for diabetic macular oedema. *Cochrane Database of Systematic Reviews* 2018(4). - McNicol ED, Rowe E, Cooper TE: Ketorolac for postoperative pain in children. *Cochrane Database of Systematic Reviews* 2018(7). - McNicol ED, Ferguson MC, Schumann R: Single‐dose intravenous diclofenac for acute postoperative pain in adults. *Cochrane Database of Systematic Reviews* 2018(8). - McMurray JJ, Ponikowski P, Bolli GB, Lukashevich V, Kozlovski P, Kothny W, Lewsey JD, Krum H, Committees VT, Investigators: Effects of vildagliptin on ventricular function in patients with type 2 diabetes mellitus and heart failure: a randomized placebo-controlled trial. *JACC: Heart Failure* 2018, 6(1):8-17. - McMurray JJ, Ponikowski P, Bolli GB, Lukashevich V, Kozlovski P, Kothny W, Lewsey JD, Krum H, Committees VT, Investigators: Effects of vildagliptin on ventricular function in patients with type 2 diabetes mellitus and heart failure: a randomized placebo-controlled trial. *JACC: Heart Failure* 2018, 6(1):8-17. - McInnes E, Jammali‐Blasi A, Bell‐Syer SEM, Leung V: Support surfaces for treating pressure ulcers. *Cochrane Database of Systematic Reviews* 2018(10). - Matthews PRL, Horder J, Pearce M: Selective noradrenaline reuptake inhibitors for schizophrenia. *Cochrane Database of Systematic Reviews* 2018(1). - Matar HE, Almerie MQ, Sampson SJ: Fluphenazine (oral) versus placebo for schizophrenia. *Cochrane Database of Systematic Reviews* 2018(6). - Mantovani A, Rigolon R, Turino T, Pichiri I, Falceri A, Rossi A, Temporelli PL, Bonapace S, Lippi G, Zoppini G *et al*: Association between decreasing estimated glomerular filtration rate and risk of cardiac conduction defects in patients with type 2 diabetes. *Diabetes Metab* 2018, 44(6):473-481. - Mantovani A, Rigolon R, Turino T, Pichiri I, Falceri A, Rossi A, Temporelli PL, Bonapace S, Lippi G, Zoppini G *et al*: Association between decreasing estimated glomerular filtration rate and risk of cardiac conduction defects in patients with type 2 diabetes. *Diabetes Metab* 2018, 44(6):473-481. - Mantovani A, Rigolon R, Civettini A, Bolzan B, Morani G, Bonapace S, Dugo C, Zoppini G, Bonora E, Targher G: Hyperuricemia is associated with an increased prevalence of paroxysmal atrial fibrillation in patients with type 2 diabetes referred for clinically indicated 24-h Holter monitoring. *Journal of endocrinological investigation* 2018, 41:223-231. - Mantovani A, Rigolon R, Civettini A, Bolzan B, Morani G, Bonapace S, Dugo C, Zoppini G, Bonora E, Targher G: Hyperuricemia is associated with an increased prevalence of paroxysmal atrial fibrillation in patients with type 2 diabetes referred for clinically indicated 24-h Holter monitoring. *Journal of endocrinological investigation* 2018, 41(2):223-231. - Manresa C, Sanz‐Miralles EC, Twigg J, Bravo M: Supportive periodontal therapy (SPT) for maintaining the dentition in adults treated for periodontitis. *Cochrane Database of Systematic Reviews* 2018(1). - Manheimer E, Cheng K, Wieland LS, Shen X, Lao L, Guo M, Berman BM: Acupuncture for hip osteoarthritis. *Cochrane Database of Systematic Reviews* 2018(5). - Mandall NA, Hickman J, Macfarlane TV, Mattick RCR, Millett DT, Worthington HV: Adhesives for fixed orthodontic brackets. *Cochrane Database of Systematic Reviews* 2018(4). - Malouf R, Ashraf A, Hadjinicolaou AV, Doree C, Hopewell S, Estcourt LJ: Comparison of a therapeutic‐only versus prophylactic platelet transfusion policy for people with congenital or acquired bone marrow failure disorders. *Cochrane Database of Systematic Reviews* 2018(5). - Mallya NV, DR SK, Mashal S: A study to evaluate the behavioral dimensions of nomophobia and attitude toward smartphone usage among medical students in Bengaluru. *National Journal of Physiology, Pharmacy and Pharmacology* 2018, 8(11):1553-1553. - Makrgeorgou A, Leonardi‐Bee J, Bath‐Hextall FJ, Murrell DF, Tang MLK, Roberts A, Boyle RJ: Probiotics for treating eczema. *Cochrane Database of Systematic Reviews* 2018(11). - Lyu Y, Luo Y, Li C, Guo X, Lu J, Wu H, Huo X, Gu W, Yang G, Ji L: Regional differences in the prevalence of coronary heart disease and stroke in patients with type 2 diabetes in China. *The Journal of Clinical Endocrinology & Metabolism* 2018, 103(9):3319-3330. - Lyu Y, Luo Y, Li C, Guo X, Lu J, Wu H, Huo X, Gu W, Yang G, Ji L: Regional differences in the prevalence of coronary heart disease and stroke in patients with type 2 diabetes in China. *The Journal of Clinical Endocrinology & Metabolism* 2018, 103(9):3319-3330. - Lynch EA, Jones TM, Simpson DB, Fini NA, Kuys SS, Borschmann K, Kramer S, Johnson L, Callisaya ML, Mahendran N *et al*: Activity monitors for increasing physical activity in adult stroke survivors. *Cochrane Database of Systematic Reviews* 2018(7). - Loughney LA, West MA, Kemp GJ, Grocott MPW, Jack S: Exercise interventions for people undergoing multimodal cancer treatment that includes surgery. *Cochrane Database of Systematic Reviews* 2018(12). - López‐Alcalde J, Rodriguez‐Barrientos R, Redondo‐Sánchez J, Muñoz‐Gutiérrez J, Molero García JM, Rodríguez‐Fernández C, Heras‐Mosteiro J, Marin‐Cañada J, Casanova‐Colominas J, Azcoaga‐Lorenzo A *et al*: Short‐course versus long‐course therapy of the same antibiotic for community‐acquired pneumonia in adolescent and adult outpatients. *Cochrane Database of Systematic Reviews* 2018(9). - Long L, Anderson L, Dewhirst AM, He J, Bridges C, Gandhi M, Taylor RS: Exercise‐based cardiac rehabilitation for adults with stable angina. *Cochrane Database of Systematic Reviews* 2018(2). - Liu Z, Sun YY, Zhong BL: Mindfulness‐based stress reduction for family carers of people with dementia. *Cochrane Database of Systematic Reviews* 2018(8). - Liu Z, Dumville JC, Norman G, Westby MJ, Blazeby J, McFarlane E, Welton NJ, O'Connor L, Cawthorne J, George RP *et al*: Intraoperative interventions for preventing surgical site infection: an overview of Cochrane Reviews. *Cochrane Database of Systematic Reviews* 2018(2). - Liu J, Zhang J, Wang LN: Gamma aminobutyric acid (GABA) receptor agonists for acute stroke. *Cochrane Database of Systematic Reviews* 2018(10). - Li D, Li X, Cui W, Shen H, Zhu H, Xia Y: Liberal versus conservative fluid therapy in adults and children with sepsis or septic shock. *Cochrane Database of Systematic Reviews* 2018(12). - Lewis SR, Schofield‐Robinson OJ, Alderson P, Smith AF: Enteral versus parenteral nutrition and enteral versus a combination of enteral and parenteral nutrition for adults in the intensive care unit. *Cochrane Database of Systematic Reviews* 2018(6). - Lewis SR, Schofield‐Robinson OJ, Alderson P, Smith AF: Propofol for the promotion of sleep in adults in the intensive care unit. *Cochrane Database of Systematic Reviews* 2018(1). - Lewis SR, Pritchard MW, Schofield‐Robinson OJ, Evans DJW, Alderson P, Smith AF: Information or education interventions for adult intensive care unit (ICU) patients and their carers. *Cochrane Database of Systematic Reviews* 2018(10). - Lewis SR, Pritchard MW, Schofield‐Robinson OJ, Alderson P, Smith AF: Continuation versus discontinuation of antiplatelet therapy for bleeding and ischaemic events in adults undergoing non‐cardiac surgery. *Cochrane Database of Systematic Reviews* 2018(7). - Lewis SR, Pritchard MW, Schofield‐Robinson OJ, Alderson P, Smith AF: Melatonin for the promotion of sleep in adults in the intensive care unit. *Cochrane Database of Systematic Reviews* 2018(5). - Légaré F, Adekpedjou R, Stacey D, Turcotte S, Kryworuchko J, Graham ID, Lyddiatt A, Politi MC, Thomson R, Elwyn G *et al*: Interventions for increasing the use of shared decision making by healthcare professionals. *Cochrane Database of Systematic Reviews* 2018(7). - Lawton K, Royals K, Carson‐Chahhoud KV, Campbell F, Smith BJ: Nurse‐led versus doctor‐led care for bronchiectasis. *Cochrane Database of Systematic Reviews* 2018(6). - Lawrenson JG, Graham‐Rowe E, Lorencatto F, Burr J, Bunce C, Francis JJ, Aluko P, Rice S, Vale L, Peto T *et al*: Interventions to increase attendance for diabetic retinopathy screening. *Cochrane Database of Systematic Reviews* 2018(1). - Laurant M, van der Biezen M, Wijers N, Watananirun K, Kontopantelis E, van Vught A: Nurses as substitutes for doctors in primary care. *Cochrane Database of Systematic Reviews* 2018(7). - Langhorne P, Collier JM, Bate PJ, Thuy MNT, Bernhardt J: Very early versus delayed mobilisation after stroke. *Cochrane Database of Systematic Reviews* 2018(10). - Lamont T, Worthington HV, Clarkson JE, Beirne PV: Routine scale and polish for periodontal health in adults. *Cochrane Database of Systematic Reviews* 2018(12). - Kwan I, Wang R, Pearce E, Bhattacharya S: Pain relief for women undergoing oocyte retrieval for assisted reproduction. *Cochrane Database of Systematic Reviews* 2018(5). - Kuzu F: The effect of type 2 diabetes on electrocardiographic markers of significant cardiac events. *Pakistan journal of medical sciences* 2018, 34(3):626. - Kuzu F: The effect of type 2 diabetes on electrocardiographic markers of significant cardiac events. *Pakistan journal of medical sciences* 2018, 34(3):626-632. - Kumbargere Nagraj S, Prashanti E, Aggarwal H, Lingappa A, Muthu MS, Kiran Kumar Krishanappa S, Hassan H: Interventions for treating post‐extraction bleeding. *Cochrane Database of Systematic Reviews* 2018(3). - Küley‐Bagheri Y, Kreuzer KA, Monsef I, Lübbert M, Skoetz N: Effects of all‐trans retinoic acid (ATRA) in addition to chemotherapy for adults with acute myeloid leukaemia (AML) (non‐acute promyelocytic leukaemia (non‐APL)). *Cochrane Database of Systematic Reviews* 2018(8). - Kubota Y, Yamamoto T, Tara S, Tokita Y, Yodogawa K, Iwasaki Y, Takano H, Tsukada Y, Asai K, Miyamoto M: Effect of empagliflozin versus placebo on cardiac sympathetic activity in acute myocardial infarction patients with type 2 diabetes mellitus: rationale. *Diabetes Therapy* 2018, 9:2107-2116. - Kubota Y, Yamamoto T, Tara S, Tokita Y, Yodogawa K, Iwasaki Y, Takano H, Tsukada Y, Asai K, Miyamoto M: Effect of empagliflozin versus placebo on cardiac sympathetic activity in acute myocardial infarction patients with type 2 diabetes mellitus: rationale. *Diabetes Therapy* 2018, 9(5):2107-2116. - Krishnaiah B, Ramaratnam S, Ranganathan LN: Subpial transection surgery for epilepsy. *Cochrane Database of Systematic Reviews* 2018(11). - Kopsaftis Z, Wood‐Baker R, Poole P: Influenza vaccine for chronic obstructive pulmonary disease (COPD). *Cochrane Database of Systematic Reviews* 2018(6). - Köpke S, Solari A, Rahn A, Khan F, Heesen C, Giordano A: Information provision for people with multiple sclerosis. *Cochrane Database of Systematic Reviews* 2018(10). - Kolkailah AA, Alreshq RS, Muhammed AM, Zahran ME, Anas El‐Wegoud M, Nabhan AF: Transradial versus transfemoral approach for diagnostic coronary angiography and percutaneous coronary intervention in people with coronary artery disease. *Cochrane Database of Systematic Reviews* 2018(4). - Kobayashi S, Nagao M, Asai A, Fukuda I, Oikawa S, Sugihara H: Severity and multiplicity of microvascular complications are associated with QT interval prolongation in patients with type 2 diabetes. *Journal of diabetes investigation* 2018, 9(4):946-951. - Kobayashi S, Nagao M, Asai A, Fukuda I, Oikawa S, Sugihara H: Severity and multiplicity of microvascular complications are associated with QT interval prolongation in patients with type 2 diabetes. *Journal of diabetes investigation* 2018, 9(4):946-951. - Kloukos D, Fudalej P, Sequeira‐Byron P, Katsaros C: Maxillary distraction osteogenesis versus orthognathic surgery for cleft lip and palate patients. *Cochrane Database of Systematic Reviews* 2018(8). - Kirkland SW, Cross E, Campbell S, Villa‐Roel C, Rowe BH: Intramuscular versus oral corticosteroids to reduce relapses following discharge from the emergency department for acute asthma. *Cochrane Database of Systematic Reviews* 2018(6). - Kimani SW: Uses and Gratifications of Facebook among Millennials in Kenya: A Case of United States International University-Africa Students. United States International University-Africa; 2018. - Kim KH, Lee MS, Choi TY, Kim TH: Acupuncture for symptomatic gastroparesis. *Cochrane Database of Systematic Reviews* 2018(12). - Khatib MN, Shankar AH, Kirubakaran R, Gaidhane A, Gaidhane S, Simkhada P, Quazi Syed Z: Ghrelin for the management of cachexia associated with cancer. *Cochrane Database of Systematic Reviews* 2018(2). - Khan R, Plahouras J, Johnston BC, Scaffidi MA, Grover SC, Walsh CM: Virtual reality simulation training for health professions trainees in gastrointestinal endoscopy. *Cochrane Database of Systematic Reviews* 2018(8). - Kelly C, Grundy S, Lynes D, Evans DJW, Gudur S, Milan SJ, Spencer S: Self‐management for bronchiectasis. *Cochrane Database of Systematic Reviews* 2018(2). - Kelly C, Chalmers JD, Crossingham I, Relph N, Felix LM, Evans DJ, Milan SJ, Spencer S: Macrolide antibiotics for bronchiectasis. *Cochrane Database of Systematic Reviews* 2018(3). - Kebede A, Gerensea H, Amare F, Tesfay Y, Teklay G: The magnitude of anemia and associated factors among pregnant women attending public institutions of Shire Town, Shire, Tigray, Northern Ethiopia, 2018. *BMC research notes* 2018, 11:1-6. - Kapur N, Petsky HL, Bell S, Kolbe J, Chang AB: Inhaled corticosteroids for bronchiectasis. *Cochrane Database of Systematic Reviews* 2018(5). - Kaner EFS, Beyer FR, Muirhead C, Campbell F, Pienaar ED, Bertholet N, Daeppen JB, Saunders JB, Burnand B: Effectiveness of brief alcohol interventions in primary care populations. *Cochrane Database of Systematic Reviews* 2018(2). - Kahn SR, Morrison DR, Diendéré G, Piché A, Filion KB, Klil‐Drori AJ, Douketis JD, Emed J, Roussin A, Tagalakis V *et al*: Interventions for implementation of thromboprophylaxis in hospitalized patients at risk for venous thromboembolism. *Cochrane Database of Systematic Reviews* 2018(4). - Kafil TS, Nguyen TM, MacDonald JK, Chande N: Cannabis for the treatment of Crohn's disease. *Cochrane Database of Systematic Reviews* 2018(11). - Kafil TS, Nguyen TM, MacDonald JK, Chande N: Cannabis for the treatment of ulcerative colitis. *Cochrane Database of Systematic Reviews* 2018(11). - Kaehne A, Milan SJ, Felix LM, Sheridan E, Marsden PA, Spencer S: Head‐to‐head trials of antibiotics for bronchiectasis. *Cochrane Database of Systematic Reviews* 2018(9). - Joyce P, Moore ZEH, Christie J: Organisation of health services for preventing and treating pressure ulcers. *Cochrane Database of Systematic Reviews* 2018(12). - Joseph J, Martinez‐Devesa P, Bellorini J, Burton MJ: Tranexamic acid for patients with nasal haemorrhage (epistaxis). *Cochrane Database of Systematic Reviews* 2018(12). - Jørgensen PG, Jensen MT, Biering‐Sørensen T, Mogelvang R, Fritz‐Hansen T, Vilsbøll T, Rossing P, Jensen JS: Burden of uncontrolled metabolic risk factors and left ventricular structure and function in patients with type 2 diabetes mellitus. *Journal of the American Heart Association* 2018, 7(19):e008856. - Jørgensen PG, Jensen MT, Biering‐Sørensen T, Mogelvang R, Fritz‐Hansen T, Vilsbøll T, Rossing P, Jensen JS: Burden of uncontrolled metabolic risk factors and left ventricular structure and function in patients with type 2 diabetes mellitus. *Journal of the American Heart Association* 2018, 7(19):e008856. - Jones HG, Rees M, Aboumarzouk OM, Brown J, Cragg J, Billings P, Carter B, Chandran P: Prosthetic mesh placement for the prevention of parastomal herniation. *Cochrane Database of Systematic Reviews* 2018(7). - Jones C, Hacker D, Xia J, Meaden A, Irving CB, Zhao S, Chen J, Shi C: Cognitive behavioural therapy plus standard care versus standard care for people with schizophrenia. *Cochrane Database of Systematic Reviews* 2018(12). - Jones C, Hacker D, Meaden A, Cormac I, Irving CB, Xia J, Zhao S, Shi C, Chen J: Cognitive behavioural therapy plus standard care versus standard care plus other psychosocial treatments for people with schizophrenia. *Cochrane Database of Systematic Reviews* 2018(11). - Johnson S, Henschke N, Maayan N, Mills I, Buckley BS, Kakourou A, Marshall R: Ribavirin for treating Crimean Congo haemorrhagic fever. *Cochrane Database of Systematic Reviews* 2018(6). - Jenkinson MD, Barone DG, Bryant A, Vale L, Bulbeck H, Lawrie TA, Hart MG, Watts C: Intraoperative imaging technology to maximise extent of resection for glioma. *Cochrane Database of Systematic Reviews* 2018(1). - Jagannath VA, Filippini G, Borges do Nascimento IJ, Di Pietrantonj C, Robak EW, Whamond L: Vitamin D for the management of multiple sclerosis. *Cochrane Database of Systematic Reviews* 2018(9). - Jacobson Vann JC, Jacobson RM, Coyne‐Beasley T, Asafu‐Adjei JK, Szilagyi PG: Patient reminder and recall interventions to improve immunization rates. *Cochrane Database of Systematic Reviews* 2018(1). - Inagaki N, Sano H, Seki Y, Kuroda S, Kaku K: Efficacy and safety of once‐weekly oral trelagliptin switched from once‐daily dipeptidyl peptidase‐4 inhibitor in patients with type 2 diabetes mellitus: an open‐label, phase 3 exploratory study. *Journal of Diabetes Investigation* 2018, 9(2):354-359. - Inagaki N, Sano H, Seki Y, Kuroda S, Kaku K: Efficacy and safety of once‐weekly oral trelagliptin switched from once‐daily dipeptidyl peptidase‐4 inhibitor in patients with type 2 diabetes mellitus: an open‐label, phase 3 exploratory study. *Journal of diabetes investigation* 2018, 9(2):354-359. - Ijaz S, Davies P, Williams CJ, Kessler D, Lewis G, Wiles N: Psychological therapies for treatment‐resistant depression in adults. *Cochrane Database of Systematic Reviews* 2018(5). - Iheozor‐Ejiofor Z, Newton K, Dumville JC, Costa ML, Norman G, Bruce J: Negative pressure wound therapy for open traumatic wounds. *Cochrane Database of Systematic Reviews* 2018(7). - Ibrahim NK, Baharoon BS, Banjar WF, Jar AA, Ashor RM, Aman AA, Al-Ahmadi JR: Mobile phone addiction and its relationship to sleep quality and academic achievement of medical students at King Abdulaziz University, Jeddah, Saudi Arabia. *Journal of research in health sciences* 2018, 18(3):e00420. - Hussain K, Murdin L, Schilder AGM: Restriction of salt, caffeine and alcohol intake for the treatment of Ménière's disease or syndrome. *Cochrane Database of Systematic Reviews* 2018(12). - Hurley M, Dickson K, Hallett R, Grant R, Hauari H, Walsh N, Stansfield C, Oliver S: Exercise interventions and patient beliefs for people with hip, knee or hip and knee osteoarthritis: a mixed methods review. *Cochrane Database of Systematic Reviews* 2018(4). - Hua C, Bosc R, Sbidian E, De Prost N, Hughes C, Jabre P, Chosidow O, Le Cleach L: Interventions for necrotizing soft tissue infections in adults. *Cochrane Database of Systematic Reviews* 2018(5). - Htay H, Johnson DW, Wiggins KJ, Badve SV, Craig JC, Strippoli GFM, Cho Y: Biocompatible dialysis fluids for peritoneal dialysis. *Cochrane Database of Systematic Reviews* 2018(10). - Hsu CCT, Kwan GNC, Singh D, Rophael JA, Anthony C, van Driel ML: Angioplasty versus stenting for infrapopliteal arterial lesions in chronic limb‐threatening ischaemia. *Cochrane Database of Systematic Reviews* 2018(12). - Hsu CCT, Kwan GNC, Evans‐Barns H, van Driel ML: Embolisation for pulmonary arteriovenous malformation. *Cochrane Database of Systematic Reviews* 2018(1). - Howes N, Atkinson C, Thomas S, Lewis SJ: Immunonutrition for patients undergoing surgery for head and neck cancer. *Cochrane Database of Systematic Reviews* 2018(8). - Howard J, Dwivedi RC, Masterson L, Kothari P, Quon H, Holsinger FC: De‐intensified adjuvant (chemo)radiotherapy versus standard adjuvant chemoradiotherapy post transoral minimally invasive surgery for resectable HPV‐positive oropharyngeal carcinoma. *Cochrane Database of Systematic Reviews* 2018(12). - Hopewell S, Adedire O, Copsey BJ, Boniface GJ, Sherrington C, Clemson L, Close JCT, Lamb SE: Multifactorial and multiple component interventions for preventing falls in older people living in the community. *Cochrane Database of Systematic Reviews* 2018(7). - Hoe VCW, Urquhart DM, Kelsall HL, Zamri EN, Sim MR: Ergonomic interventions for preventing work‐related musculoskeletal disorders of the upper limb and neck among office workers. *Cochrane Database of Systematic Reviews* 2018(10). - Hines S, Steels E, Chang A, Gibbons K: Aromatherapy for treatment of postoperative nausea and vomiting. *Cochrane Database of Systematic Reviews* 2018(3). - Herling SF, Greve IE, Vasilevskis EE, Egerod I, Bekker Mortensen C, Møller AM, Svenningsen H, Thomsen T: Interventions for preventing intensive care unit delirium in adults. *Cochrane Database of Systematic Reviews* 2018(11). - Herke M, Fink A, Langer G, Wustmann T, Watzke S, Hanff AM, Burckhardt M: Environmental and behavioural modifications for improving food and fluid intake in people with dementia. *Cochrane Database of Systematic Reviews* 2018(7). - Herath SC, Normansell R, Maisey S, Poole P: Prophylactic antibiotic therapy for chronic obstructive pulmonary disease (COPD). *Cochrane Database of Systematic Reviews* 2018(10). - Henning RJ: Type-2 diabetes mellitus and cardiovascular disease. *Future cardiology* 2018, 14(6):491-509. - Henning RJ: Type-2 diabetes mellitus and cardiovascular disease. *Future cardiology* 2018, 14(6):491-509. - Head K, Snidvongs K, Glew S, Scadding G, Schilder AGM, Philpott C, Hopkins C: Saline irrigation for allergic rhinitis. *Cochrane Database of Systematic Reviews* 2018(6). - Head K, Sharp S, Chong LY, Hopkins C, Philpott C: Topical and systemic antifungal therapy for chronic rhinosinusitis. *Cochrane Database of Systematic Reviews* 2018(9). - Hassan S, Haridas A, Sundaram V: Adjustable versus non‐adjustable sutures for strabismus. *Cochrane Database of Systematic Reviews* 2018(3). - Harvey AR, Baker LB, Reddihough DS, Scheinberg A, Williams K: Trihexyphenidyl for dystonia in cerebral palsy. *Cochrane Database of Systematic Reviews* 2018(5). - Haroon MZ, Zeb Z, Javed Z, Awan Z, Aftab Z, Talat W: Internet Addiction In Medical Students. *J Ayub Med Coll Abbottabad* 2018, 30(Suppl 1)(4):S659-s663. - Harada T, Tsutomi H, Mori R, Wilson DB: Cognitive‐behavioural treatment for amphetamine‐type stimulants (ATS)‐use disorders. *Cochrane Database of Systematic Reviews* 2018(12). - Hall KK, Petsky HL, Chang AB, O'Grady KF: Caseworker‐assigned discharge plans to prevent hospital readmission for acute exacerbations in children with chronic respiratory illness. *Cochrane Database of Systematic Reviews* 2018(11). - Hageman D, Fokkenrood HJP, Gommans LNM, van den Houten MML, Teijink JAW: Supervised exercise therapy versus home‐based exercise therapy versus walking advice for intermittent claudication. *Cochrane Database of Systematic Reviews* 2018(4). - Gurusamy KS, Best LMJ, Tanguay C, Lennan E, Korva M, Bussières JF: Closed‐system drug‐transfer devices plus safe handling of hazardous drugs versus safe handling alone for reducing exposure to infusional hazardous drugs in healthcare staff. *Cochrane Database of Systematic Reviews* 2018(3). - Guay J, Ochroch EA, Kopp S: Intraoperative use of low volume ventilation to decrease postoperative mortality, mechanical ventilation, lengths of stay and lung injury in adults without acute lung injury. *Cochrane Database of Systematic Reviews* 2018(7). - Grabosch SM, Shariff OM, Helm CW: Non‐steroidal anti‐inflammatory agents to induce regression and prevent the progression of cervical intraepithelial neoplasia. *Cochrane Database of Systematic Reviews* 2018(2). - Gonçalves‐Bradley D, Khangura JK, Flodgren G, Perera R, Rowe BH, Shepperd S: Primary care professionals providing non‐urgent care in hospital emergency departments. *Cochrane Database of Systematic Reviews* 2018(2). - Goldkuhle M, Dimaki M, Gartlehner G, Monsef I, Dahm P, Glossmann JP, Engert A, von Tresckow B, Skoetz N: Nivolumab for adults with Hodgkin's lymphoma (a rapid review using the software RobotReviewer). *Cochrane Database of Systematic Reviews* 2018(7). - Gladius Jennifer H, Sowmiya K, Vidya D, Archana Lakshmi P, William RF: A study of mobile phone usage on sleep disturbance, stress and academic performance among medical students in Tamil Nadu. *Int J Commun Med Publ Health* 2018, 5(1):365. - Givron H, Berrewaerts J, Houbeau G, Desseilles M: [Problematic Use of Internet and Video Games in Students in Medicine]. *Adv Physiol Educ* 2018, 43(1):101-121. - Gendron LM, Nyberg A, Saey D, Maltais F, Lacasse Y: Active mind‐body movement therapies as an adjunct to or in comparison with pulmonary rehabilitation for people with chronic obstructive pulmonary disease. *Cochrane Database of Systematic Reviews* 2018(10). - Fullerton B, Siebenhofer A, Jeitler K, Horvath K, Semlitsch T, Berghold A, Gerlach FM: Short‐acting insulin analogues versus regular human insulin for adult, non‐pregnant persons with type 2 diabetes mellitus. *Cochrane Database of Systematic Reviews* 2018(12). - Fujiwara T, Kuriyama A, Kato Y, Fukuoka T, Ota E: Perioperative local anaesthesia for reducing pain following septal surgery. *Cochrane Database of Systematic Reviews* 2018(8). - Franco JVA, Turk T, Jung JH, Xiao YT, Iakhno S, Garrote V, Vietto V: Non‐pharmacological interventions for treating chronic prostatitis/chronic pelvic pain syndrome. *Cochrane Database of Systematic Reviews* 2018(5). - Fortin PM, Hopewell S, Estcourt LJ: Red blood cell transfusion to treat or prevent complications in sickle cell disease: an overview of Cochrane reviews. *Cochrane Database of Systematic Reviews* 2018(8). - Forster R, Liew A, Bhattacharya V, Shaw J, Stansby G: Gene therapy for peripheral arterial disease. *Cochrane Database of Systematic Reviews* 2018(10). - Fønhus MS, Dalsbø TK, Johansen M, Fretheim A, Skirbekk H, Flottorp SA: Patient‐mediated interventions to improve professional practice. *Cochrane Database of Systematic Reviews* 2018(9). - Fiorini HJ, Tamaoki MJ, Lenza M, Gomes dos Santos JB, Faloppa F, Belloti JC: Surgery for trigger finger. *Cochrane Database of Systematic Reviews* 2018(2). - Ferrante di Ruffano L, Takwoingi Y, Dinnes J, Chuchu N, Bayliss SE, Davenport C, Matin RN, Godfrey K, O'Sullivan C, Gulati A *et al*: Computer‐assisted diagnosis techniques (dermoscopy and spectroscopy‐based) for diagnosing skin cancer in adults. *Cochrane Database of Systematic Reviews* 2018(12). - Ferrante di Ruffano L, Dinnes J, Deeks JJ, Chuchu N, Bayliss SE, Davenport C, Takwoingi Y, Godfrey K, O'Sullivan C, Matin RN *et al*: Optical coherence tomography for diagnosing skin cancer in adults. *Cochrane Database of Systematic Reviews* 2018(12). - Ferrante di Ruffano L, Dinnes J, Chuchu N, Bayliss SE, Takwoingi Y, Davenport C, Matin RN, O'Sullivan C, Roskell D, Deeks JJ *et al*: Exfoliative cytology for diagnosing basal cell carcinoma and other skin cancers in adults. *Cochrane Database of Systematic Reviews* 2018(12). - Fakhry F, Fokkenrood HJP, Spronk S, Teijink JAW, Rouwet EV, Hunink MGM: Endovascular revascularisation versus conservative management for intermittent claudication. *Cochrane Database of Systematic Reviews* 2018(3). - Fairfield C, Penninga L, Powell J, Harrison EM, Wigmore SJ: Glucocorticosteroid‐free versus glucocorticosteroid‐containing immunosuppression for liver transplanted patients. *Cochrane Database of Systematic Reviews* 2018(4). - Fabes J, Brunskill SJ, Curry N, Doree C, Stanworth SJ: Pro‐coagulant haemostatic factors for the prevention and treatment of bleeding in people without haemophilia. *Cochrane Database of Systematic Reviews* 2018(12). - Everitt H, Baldwin DS, Stuart B, Lipinska G, Mayers A, Malizia AL, Manson CCF, Wilson S: Antidepressants for insomnia in adults. *Cochrane Database of Systematic Reviews* 2018(5). - Evaristo E, Stocco FG, Shah NR, Cheezum MK, Hainer J, Foster C, Nearing BD, Di Carli M, Verrier RL: Ranolazine reduces repolarization heterogeneity in symptomatic patients with diabetes and non–flow‐limiting coronary artery stenosis. *Annals of noninvasive electrocardiology* 2018, 23(1):n/a-n/a. - Estcourt LJ, Malouf R, Hopewell S, Doree C, Van Veen J: Use of platelet transfusions prior to lumbar punctures or epidural anaesthesia for the prevention of complications in people with thrombocytopenia. *Cochrane Database of Systematic Reviews* 2018(4). - Essali A, Soares‐Weiser K, Bergman H, Adams CE: Calcium channel blockers for antipsychotic‐induced tardive dyskinesia. *Cochrane Database of Systematic Reviews* 2018(3). - Eshun‐Wilson I, Siegfried N, Akena DH, Stein DJ, Obuku EA, Joska JA: Antidepressants for depression in adults with HIV infection. *Cochrane Database of Systematic Reviews* 2018(1). - Engelen ET, Schutgens REG, Mauser‐Bunschoten EP, van Es RJJ, van Galen KPM: Antifibrinolytic therapy for preventing oral bleeding in people on anticoagulants undergoing minor oral surgery or dental extractions. *Cochrane Database of Systematic Reviews* 2018(7). - El Sayed I, Liu Q, Wee I, Hine P: Antibiotics for treating scrub typhus. *Cochrane Database of Systematic Reviews* 2018(9). - El Moheb M, Nicolas J, Khamis AM, Iskandarani G, Akl EA, Refaat M: Implantable cardiac defibrillators for people with non‐ischaemic cardiomyopathy. *Cochrane Database of Systematic Reviews* 2018(12). - Egholm JWM, Pedersen B, Møller AM, Adami J, Juhl CB, Tønnesen H: Perioperative alcohol cessation intervention for postoperative complications. *Cochrane Database of Systematic Reviews* 2018(11). - Ede CJ, Nikolova D, Brand M: Surgical portosystemic shunts versus devascularisation procedures for prevention of variceal rebleeding in people with hepatosplenic schistosomiasis. *Cochrane Database of Systematic Reviews* 2018(8). - Eamer G, Taheri A, Chen SS, Daviduck Q, Chambers T, Shi X, Khadaroo RG: Comprehensive geriatric assessment for older people admitted to a surgical service. *Cochrane Database of Systematic Reviews* 2018(1). - Dudas R, Malouf R, McCleery J, Dening T: Antidepressants for treating depression in dementia. *Cochrane Database of Systematic Reviews* 2018(8). - Downie LE, Busija L, Keller PR: Blue‐light filtering intraocular lenses (IOLs) for protecting macular health. *Cochrane Database of Systematic Reviews* 2018(5). - Doleman B, Leonardi‐Bee J, Heinink TP, Bhattacharjee D, Lund JN, Williams JP: Pre‐emptive and preventive opioids for postoperative pain in adults undergoing all types of surgery. *Cochrane Database of Systematic Reviews* 2018(12). - Doiron KA, Hoffmann TC, Beller EM: Early intervention (mobilization or active exercise) for critically ill adults in the intensive care unit. *Cochrane Database of Systematic Reviews* 2018(3). - Do DV, Gichuhi S, Vedula SS, Hawkins BS: Surgery for postvitrectomy cataract. *Cochrane Database of Systematic Reviews* 2018(1). - Dinnes J, Deeks JJ, Saleh D, Chuchu N, Bayliss SE, Patel L, Davenport C, Takwoingi Y, Godfrey K, Matin RN *et al*: Reflectance confocal microscopy for diagnosing cutaneous melanoma in adults. *Cochrane Database of Systematic Reviews* 2018(12). - Dinnes J, Deeks JJ, Grainge MJ, Chuchu N, Ferrante di Ruffano L, Matin RN, Thomson DR, Wong KY, Aldridge RB, Abbott R *et al*: Visual inspection for diagnosing cutaneous melanoma in adults. *Cochrane Database of Systematic Reviews* 2018(12). - Dinnes J, Deeks JJ, Chuchu N, Saleh D, Bayliss SE, Takwoingi Y, Davenport C, Patel L, Matin RN, O'Sullivan C *et al*: Reflectance confocal microscopy for diagnosing keratinocyte skin cancers in adults. *Cochrane Database of Systematic Reviews* 2018(12). - Dinnes J, Deeks JJ, Chuchu N, Matin RN, Wong KY, Aldridge RB, Durack A, Gulati A, Chan SA, Johnston L *et al*: Visual inspection and dermoscopy, alone or in combination, for diagnosing keratinocyte skin cancers in adults. *Cochrane Database of Systematic Reviews* 2018(12). - Dinnes J, Deeks JJ, Chuchu N, Ferrante di Ruffano L, Matin RN, Thomson DR, Wong KY, Aldridge RB, Abbott R, Fawzy M *et al*: Dermoscopy, with and without visual inspection, for diagnosing melanoma in adults. *Cochrane Database of Systematic Reviews* 2018(12). - Dinnes J, Bamber J, Chuchu N, Bayliss SE, Takwoingi Y, Davenport C, Godfrey K, O'Sullivan C, Matin RN, Deeks JJ *et al*: High‐frequency ultrasound for diagnosing skin cancer in adults. *Cochrane Database of Systematic Reviews* 2018(12). - Deshmukh SR, Holmes J, Cardno A: Art therapy for people with dementia. *Cochrane Database of Systematic Reviews* 2018(9). - Delgado‐Noguera MF, Forero Delgadillo JM, Franco AA, Vazquez JC, Calvache JA: Corticosteroids for septic arthritis in children. *Cochrane Database of Systematic Reviews* 2018(11). - Del Fabbro M, Karanxha L, Panda S, Bucchi C, Nadathur Doraiswamy J, Sankari M, Ramamoorthi S, Varghese S, Taschieri S: Autologous platelet concentrates for treating periodontal infrabony defects. *Cochrane Database of Systematic Reviews* 2018(11). - de Barra M, Scott CL, Scott NW, Johnston M, de Bruin M, Nkansah N, Bond CM, Matheson CI, Rackow P, Williams AJ *et al*: Pharmacist services for non‐hospitalised patients. *Cochrane Database of Systematic Reviews* 2018(9). - Damor RB, Gamit SP, Modi A, Patel J, Kosambiya J: Pattern of smart phone and Internet usage among medical students in Surat, Gujarat–a cross sectional study. *National Journal of Community Medicine* 2018, 9(07):469-473. - Damor RB, Gamit SP, Modi A, Patel J, Kosambiya J: Pattern of smart phone and internet usage among medical students in Surat, Gujarat–A cross sectional study. *Natl J Community Med* 2018, 9:469-473. - Custodio E, López‐Alcalde J, Herrero M, Bouza C, Jimenez C, Storcksdieck genannt Bonsmann S, Mouratidou T, López‐Cuadrado T, Benito A, Alvar J: Nutritional supplements for patients being treated for active visceral leishmaniasis. *Cochrane Database of Systematic Reviews* 2018(3). - Coussement J, Scemla A, Abramowicz D, Nagler EV, Webster AC: Antibiotics for asymptomatic bacteriuria in kidney transplant recipients. *Cochrane Database of Systematic Reviews* 2018(2). - Cooper B, Bachoo P: Extracorporeal shock wave therapy for the healing and management of venous leg ulcers. *Cochrane Database of Systematic Reviews* 2018(6). - Comunián‐Carrasco G, Peña‐Martí GE, Martí‐Carvajal AJ: Antibiotics for treating gonorrhoea in pregnancy. *Cochrane Database of Systematic Reviews* 2018(2). - Colunga‐Lozano LE, Gonzalez Torres FJ, Delgado‐Figueroa N, Gonzalez‐Padilla DA, Hernandez AV, Roman Y, Cuello‐García CA: Sliding scale insulin for non‐critically ill hospitalised adults with diabetes mellitus. *Cochrane Database of Systematic Reviews* 2018(11). - Codella R, Ialacqua M, Terruzzi I, Luzi L: May the force be with you: why resistance training is essential for subjects with type 2 diabetes mellitus without complications. *Endocrine* 2018, 62:14-25. - Codella R, Ialacqua M, Terruzzi I, Luzi L: May the force be with you: why resistance training is essential for subjects with type 2 diabetes mellitus without complications. *Endocrine* 2018, 62(1):14-25. - Claassen YHM, van der Valk MJM, Breugom AJ, Frouws MA, Bastiaannet E, Liefers GJ, van de Velde CJH, Kapiteijn E: Survival differences with immediate versus delayed chemotherapy for asymptomatic incurable metastatic colorectal cancer. *Cochrane Database of Systematic Reviews* 2018(11). - Chung C, Bryant A, Brown PD: Interventions for the treatment of brain radionecrosis after radiotherapy or radiosurgery. *Cochrane Database of Systematic Reviews* 2018(7). - Chuchu N, Takwoingi Y, Dinnes J, Matin RN, Bassett O, Moreau JF, Bayliss SE, Davenport C, Godfrey K, O'Connell S *et al*: Smartphone applications for triaging adults with skin lesions that are suspicious for melanoma. *Cochrane Database of Systematic Reviews* 2018(12). - Chuchu N, Dinnes J, Takwoingi Y, Matin RN, Bayliss SE, Davenport C, Moreau JF, Bassett O, Godfrey K, O'Sullivan C *et al*: Teledermatology for diagnosing skin cancer in adults. *Cochrane Database of Systematic Reviews* 2018(12). - Chin V, Nagrial A, Sjoquist K, O'Connor CA, Chantrill L, Biankin AV, Scholten R, Yip D: Chemotherapy and radiotherapy for advanced pancreatic cancer. *Cochrane Database of Systematic Reviews* 2018(3). - Chen I, Opiyo N, Tavender E, Mortazhejri S, Rader T, Petkovic J, Yogasingam S, Taljaard M, Agarwal S, Laopaiboon M *et al*: Non‐clinical interventions for reducing unnecessary caesarean section. *Cochrane Database of Systematic Reviews* 2018(9). - Chaputula AH, Mutula S: Factors impacting library-related uses of mobile phones by students in public universities in Malawi. *South African Journal of Libraries and Information Science* 2018, 84(1):35-46. - Chaputula AH, Mutula S: Factors impacting library-related uses of mobile phones by students in public universities in Malawi. *South African Journal of Libraries and Information Science* 2018, 84(1):35-46. - Chambrone L, Salinas Ortega MA, Sukekava F, Rotundo R, Kalemaj Z, Buti J, Pini Prato GP: Root coverage procedures for treating localised and multiple recession‐type defects. *Cochrane Database of Systematic Reviews* 2018(10). - Cha S-A, Park Y-M, Yun J-S, Lee S-H, Ahn Y-B, Kim S-R, Ko S-H: Time-and frequency-domain measures of heart rate variability predict cardiovascular outcome in patients with type 2 diabetes. *Diabetes research and clinical practice* 2018, 143:159-169. - Cha S-A, Park Y-M, Yun J-S, Lee S-H, Ahn Y-B, Kim S-R, Ko S-H: Time-and frequency-domain measures of heart rate variability predict cardiovascular outcome in patients with type 2 diabetes. *Diabetes research and clinical practice* 2018, 143:159-169. - Castells X, Blanco‐Silvente L, Cunill R: Amphetamines for attention deficit hyperactivity disorder (ADHD) in adults. *Cochrane Database of Systematic Reviews* 2018(8). - Carr PJ, Higgins NS, Cooke ML, Mihala G, Rickard CM: Vascular access specialist teams for device insertion and prevention of failure. *Cochrane Database of Systematic Reviews* 2018(3). - Carbine NE, Lostumbo L, Wallace J, Ko H: Risk‐reducing mastectomy for the prevention of primary breast cancer. *Cochrane Database of Systematic Reviews* 2018(4). - Cao Y, Zeng W, Cui Y, Kong X, Wang M, Yu J, Zhang S, Song J, Yan X, Greiser A *et al*: Increased myocardial extracellular volume assessed by cardiovascular magnetic resonance T1 mapping and its determinants in type 2 diabetes mellitus patients with normal myocardial systolic strain. *Cardiovascular diabetology* 2018, 17(1):7-7. - Cao Y, Zeng W, Cui Y, Kong X, Wang M, Yu J, Zhang S, Song J, Yan X, Greiser A: Increased myocardial extracellular volume assessed by cardiovascular magnetic resonance T1 mapping and its determinants in type 2 diabetes mellitus patients with normal myocardial systolic strain. *Cardiovascular diabetology* 2018, 17:1-12. - Cao Y, Zeng W, Cui Y, Kong X, Wang M, Yu J, Zhang S, Song J, Yan X, Greiser A: Increased myocardial extracellular volume assessed by cardiovascular magnetic resonance T1 mapping and its determinants in type 2 diabetes mellitus patients with normal myocardial systolic strain. *Cardiovascular diabetology* 2018, 17(1):1-12. - Campschroer T, Zhu X, Vernooij RWM, Lock M: Alpha‐blockers as medical expulsive therapy for ureteral stones. *Cochrane Database of Systematic Reviews* 2018(4). - Cacione DG, do Carmo Novaes F, Moreno DH: Stem cell therapy for treatment of thromboangiitis obliterans (Buerger's disease). *Cochrane Database of Systematic Reviews* 2018(10). - Burry L, Mehta S, Perreault MM, Luxenberg JS, Siddiqi N, Hutton B, Fergusson DA, Bell C, Rose L: Antipsychotics for treatment of delirium in hospitalised non‐ICU patients. *Cochrane Database of Systematic Reviews* 2018(6). - Buggeskov KB, Grønlykke L, Risom EC, Wei ML, Wetterslev J: Pulmonary artery perfusion versus no perfusion during cardiopulmonary bypass for open heart surgery in adults. *Cochrane Database of Systematic Reviews* 2018(2). - Buchbinder R, Johnston RV, Rischin KJ, Homik J, Jones CA, Golmohammadi K, Kallmes DF: Percutaneous vertebroplasty for osteoporotic vertebral compression fracture. *Cochrane Database of Systematic Reviews* 2018(11). - Bruins Slot KMH, Berge E: Factor Xa inhibitors versus vitamin K antagonists for preventing cerebral or systemic embolism in patients with atrial fibrillation. *Cochrane Database of Systematic Reviews* 2018(3). - Brinck ECV, Tiippana E, Heesen M, Bell RF, Straube S, Moore RA, Kontinen V: Perioperative intravenous ketamine for acute postoperative pain in adults. *Cochrane Database of Systematic Reviews* 2018(12). - Brignell A, Chenausky KV, Song H, Zhu J, Suo C, Morgan AT: Communication interventions for autism spectrum disorder in minimally verbal children. *Cochrane Database of Systematic Reviews* 2018(11). - Bridgwood B, Lager KE, Mistri AK, Khunti K, Wilson AD, Modi P: Interventions for improving modifiable risk factor control in the secondary prevention of stroke. *Cochrane Database of Systematic Reviews* 2018(5). - Boyapati RK, Torres J, Palmela C, Parker CE, Silverberg OM, Upadhyaya SD, Nguyen TM, Colombel JF: Withdrawal of immunosuppressant or biologic therapy for patients with quiescent Crohn's disease. *Cochrane Database of Systematic Reviews* 2018(5). - Bouthoorn S, Valstar GB, Gohar A, den Ruijter HM, Reitsma HB, Hoes AW, Rutten FH: The prevalence of left ventricular diastolic dysfunction and heart failure with preserved ejection fraction in men and women with type 2 diabetes : A systematic review and meta-analysis. *Diabetes & vascular disease research* 2018, 15(6):477-493. - Bourke JP, Bueser T, Quinlivan R: Interventions for preventing and treating cardiac complications in Duchenne and Becker muscular dystrophy and X‐linked dilated cardiomyopathy. *Cochrane Database of Systematic Reviews* 2018(10). - Boumosleh J, Jaalouk D: Smartphone addiction among university students and its relationship with academic performance. *Global Journal of Health Science* 2018, 10(1):48-59. - Boumosleh J, Jaalouk D: Smartphone addiction among university students and its relationship with academic performance. *Global Journal of Health Science* 2018, 10(1):48-59. - Blessberger H, Kammler J, Domanovits H, Schlager O, Wildner B, Azar D, Schillinger M, Wiesbauer F, Steinwender C: Perioperative beta‐blockers for preventing surgery‐related mortality and morbidity. *Cochrane Database of Systematic Reviews* 2018(3). - Bitterman R, Eliakim‐Raz N, Vinograd I, Zalmanovici Trestioreanu A, Leibovici L, Paul M: Influenza vaccines in immunosuppressed adults with cancer. *Cochrane Database of Systematic Reviews* 2018(2). - Birks JS, Harvey RJ: Donepezil for dementia due to Alzheimer's disease. *Cochrane Database of Systematic Reviews* 2018(6). - Bighelli I, Castellazzi M, Cipriani A, Girlanda F, Guaiana G, Koesters M, Turrini G, Furukawa TA, Barbui C: Antidepressants versus placebo for panic disorder in adults. *Cochrane Database of Systematic Reviews* 2018(4). - Best LMJ, Takwoingi Y, Siddique S, Selladurai A, Gandhi A, Low B, Yaghoobi M, Gurusamy KS: Non‐invasive diagnostic tests for Helicobacter pylori infection. *Cochrane Database of Systematic Reviews* 2018(3). - Bergman H, Soares‐Weiser K: Anticholinergic medication for antipsychotic‐induced tardive dyskinesia. *Cochrane Database of Systematic Reviews* 2018(1). - Bergman H, Rathbone J, Agarwal V, Soares‐Weiser K: Antipsychotic reduction and/or cessation and antipsychotics as specific treatments for tardive dyskinesia. *Cochrane Database of Systematic Reviews* 2018(2). - Bergman H, Kornør H, Nikolakopoulou A, Hanssen‐Bauer K, Soares‐Weiser K, Tollefsen TK, Bjørndal A: Client feedback in psychological therapy for children and adolescents with mental health problems. *Cochrane Database of Systematic Reviews* 2018(8). - Bergman H, Bhoopathi PS, Soares‐Weiser K: Benzodiazepines for antipsychotic‐induced tardive dyskinesia. *Cochrane Database of Systematic Reviews* 2018(1). - Benichou T, Pereira B, Mermillod M, Tauveron I, Pfabigan D, Maqdasy S, Dutheil F: Heart rate variability in type 2 diabetes mellitus: A systematic review and meta–analysis. *PloS one* 2018, 13(4):e0195166. - Benichou T, Pereira B, Mermillod M, Tauveron I, Pfabigan D, Maqdasy S, Dutheil F: Heart rate variability in type 2 diabetes mellitus: A systematic review and meta–analysis. *PloS one* 2018, 13(4):e0195166. - Bell S, Rennie T, Marwick CA, Davey P: Effects of peri‐operative nonsteroidal anti‐inflammatory drugs on post‐operative kidney function for adults with normal kidney function. *Cochrane Database of Systematic Reviews* 2018(11). - Bayramoğlu A, Taşolar H, Kaya Y, Bektaş O, Kaya A, Yaman M, Günaydın ZY: Fragmented QRS complexes are associated with left ventricular dysfunction in patients with type-2 diabetes mellitus: a two-dimensional speckle tracking echocardiography study. *Acta Cardiologica* 2018, 73(5):449-456. - Bauer A, Rönsch H, Elsner P, Dittmar D, Bennett C, Schuttelaar MLA, Lukács J, John SM, Williams HC: Interventions for preventing occupational irritant hand dermatitis. *Cochrane Database of Systematic Reviews* 2018(4). - Batista K, Thiruvenkatachari B, Harrison JE, O'Brien KD: Orthodontic treatment for prominent upper front teeth (Class II malocclusion) in children and adolescents. *Cochrane Database of Systematic Reviews* 2018(3). - Bassi D, Santos-de-Araújo AD, Camargo PF, Dibai-Filho AV, da Fonseca MA, Mendes RG, Borghi-Silva A: Inter and Intra-Rater Reliability of Short-Term Measurement of Heart Rate Variability on Rest in Diabetic Type 2 Patients. *Journal of medical systems* 2018, 42(12):1-7. - Barr S, Howe TE: Prosthetic rehabilitation for older dysvascular people following a unilateral transfemoral amputation. *Cochrane Database of Systematic Reviews* 2018(10). - Barnes H, Holland AE, Westall GP, Goh NSL, Glaspole IN: Cyclophosphamide for connective tissue disease–associated interstitial lung disease. *Cochrane Database of Systematic Reviews* 2018(1). - Barbato A, D'Avanzo B, Parabiaghi A: Couple therapy for depression. *Cochrane Database of Systematic Reviews* 2018(6). - Baldassarre MPA, Andersen A, Consoli A, Knop FK, Vilsbøll T: Cardiovascular biomarkers in clinical studies of type 2 diabetes. *Diabetes, obesity & metabolism* 2018, 20(6):1350-1360. - Bala MM, Paszek E, Lesniak W, Wloch‐Kopec D, Jasinska K, Undas A: Antiplatelet and anticoagulant agents for primary prevention of thrombosis in individuals with antiphospholipid antibodies. *Cochrane Database of Systematic Reviews* 2018(7). - Baillon SF, Narayana U, Luxenberg JS, Clifton AV: Valproate preparations for agitation in dementia. *Cochrane Database of Systematic Reviews* 2018(10). - Baandrup L, Ebdrup BH, Rasmussen J, Lindschou J, Gluud C, Glenthøj BY: Pharmacological interventions for benzodiazepine discontinuation in chronic benzodiazepine users. *Cochrane Database of Systematic Reviews* 2018(3). - Awotiwon AA, Johnson S, Rutherford GW, Meintjes G, Eshun‐Wilson I: Primary antifungal prophylaxis for cryptococcal disease in HIV‐positive people. *Cochrane Database of Systematic Reviews* 2018(8). - Aves T, Tambe J, Siemieniuk RAC, Mbuagbaw L: Antiretroviral resistance testing in HIV‐positive people. *Cochrane Database of Systematic Reviews* 2018(11). - Avau B, Borra V, Vanhove AC, Vandekerckhove P, De Paepe P, De Buck E: First aid interventions by laypeople for acute oral poisoning. *Cochrane Database of Systematic Reviews* 2018(12). - Ashley PF, Chaudhary M, Lourenço‐Matharu L: Sedation of children undergoing dental treatment. *Cochrane Database of Systematic Reviews* 2018(12). - Arechabala MC, Catoni MI, Claro JC, Rojas NP, Rubio ME, Calvo MA, Letelier LM: Antimicrobial lock solutions for preventing catheter‐related infections in haemodialysis. *Cochrane Database of Systematic Reviews* 2018(4). - Anim‐Somuah M, Smyth RMD, Cyna AM, Cuthbert A: Epidural versus non‐epidural or no analgesia for pain management in labour. *Cochrane Database of Systematic Reviews* 2018(5). - Ameratunga M, Pavlakis N, Wheeler H, Grant R, Simes J, Khasraw M: Anti‐angiogenic therapy for high‐grade glioma. *Cochrane Database of Systematic Reviews* 2018(11). - Ambler GK, Twine CP: Graft type for femoro‐popliteal bypass surgery. *Cochrane Database of Systematic Reviews* 2018(2). - Amatya B, Young J, Khan F: Non‐pharmacological interventions for chronic pain in multiple sclerosis. *Cochrane Database of Systematic Reviews* 2018(12). - AlOlaiwi LA, AlHarbi TJ, Tourkmani AM: Prevalence of cardiovascular autonomic neuropathy and gastroparesis symptoms among patients with type 2 diabetes who attend a primary health care center. *PLoS One* 2018, 13(12):e0209500. - AlOlaiwi LA, AlHarbi TJ, Tourkmani AM: Prevalence of cardiovascular autonomic neuropathy and gastroparesis symptoms among patients with type 2 diabetes who attend a primary health care center. *PLoS One* 2018, 13(12):e0209500. - Allen EN, Chandler CIR, Mandimika N, Leisegang C, Barnes K: Eliciting adverse effects data from participants in clinical trials. *Cochrane Database of Systematic Reviews* 2018(1). - Alkhalaf AM, Tekian A, Park YS: The impact of WhatsApp use on academic achievement among Saudi medical students. *Medical teacher* 2018, 40(sup1):S10-S14. - Alfadhul S: Ghazi hameed H, Mohammed SJ (2018) Internet Addiction Disorder among Medical Students in University of Kufa: A Cross Sectional Study. *J Gen Pract* 2018, 6:369. - Alabed S, Latifeh Y, Mohammad HA, Bergman H: Gamma‐aminobutyric acid agonists for antipsychotic‐induced tardive dyskinesia. *Cochrane Database of Systematic Reviews* 2018(4). - Akobeng AK, Zhang D, Gordon M, MacDonald JK: Enteral nutrition for maintenance of remission in Crohn's disease. *Cochrane Database of Systematic Reviews* 2018(8). - Akechi T, Okuyama T, Onishi J, Morita T, Furukawa TA: Psychotherapy for depression among incurable cancer patients. *Cochrane Database of Systematic Reviews* 2018(11). - Aitken LM, Bucknall T, Kent B, Mitchell M, Burmeister E, Keogh SJ: Protocol‐directed sedation versus non‐protocol‐directed sedation in mechanically ventilated intensive care adults and children. *Cochrane Database of Systematic Reviews* 2018(11). - Abrigo JM, Fountain DM, Provenzale JM, Law EK, Kwong JSW, Hart MG, Tam WWS: Magnetic resonance perfusion for differentiating low‐grade from high‐grade gliomas at first presentation. *Cochrane Database of Systematic Reviews* 2018(1). - Abraha I, Aristei C, Palumbo I, Lupattelli M, Trastulli S, Cirocchi R, De Florio R, Valentini V: Preoperative radiotherapy and curative surgery for the management of localised rectal carcinoma. *Cochrane Database of Systematic Reviews* 2018(10). - Abdul Wahid SF, Ismail NA, Wan Jamaludin WF, Muhamad NA, Abdul Hamid MKA, Harunarashid H, Lai NM: Autologous cells derived from different sources and administered using different regimens for 'no‐option' critical lower limb ischaemia patients. *Cochrane Database of Systematic Reviews* 2018(8). - Abdel‐Rahman O, Elsayed Z, Mohamed H, Eltobgy M: Radical multimodality therapy for malignant pleural mesothelioma. *Cochrane Database of Systematic Reviews* 2018(1). - Aaron K, Cooper TE, Warner L, Burton MJ: Ear drops for the removal of ear wax. *Cochrane Database of Systematic Reviews* 2018(7). - Zhou Y, Jelinek H, Hambly BD, McLachlan CS: Electrocardiogram QRS duration and associations with telomere length: A cross-sectional analysis in Australian rural diabetic and non-diabetic population. *J Electrocardiol* 2017, 50(4):450-456. - Zhou W-W, Huang B, Liu M-L: An Increase of Heart Rate and Electrocardiographic Changes after Subcutaneous Liraglutide. *Chinese medical journal* 2017, 130(23):2893-2894. - Zhang X, Chuai Y, Nie W, Wang A, Dai G: Thrombopoietin receptor agonists for prevention and treatment of chemotherapy‐induced thrombocytopenia in patients with solid tumours. *Cochrane Database of Systematic Reviews* 2017(11). - Zhang L, Weizer JS, Musch DC: Perioperative medications for preventing temporarily increased intraocular pressure after laser trabeculoplasty. *Cochrane Database of Systematic Reviews* 2017(2). - Zhan L, Yang LJ, Huang Y, He Q, Liu GJ: Continuous chest compression versus interrupted chest compression for cardiopulmonary resuscitation of non‐asphyxial out‐of‐hospital cardiac arrest. *Cochrane Database of Systematic Reviews* 2017(3). - Zekarias B, Meleko A, Hayder A, Nigatu A, Yetagessu T: Prevalence of anemia and its associated factors among pregnant women attending antenatal care (ANC) in Mizan Tepi University Teaching Hospital, South West Ethiopia. *Health Science Journal* 2017, 11(5):1-8. - Zee AAG, van Lieshout K, van der Heide M, Janssen L, Janzing HMJ: Low molecular weight heparin for prevention of venous thromboembolism in patients with lower‐limb immobilization. *Cochrane Database of Systematic Reviews* 2017(8). - Yuan B, He L, Meng Q, Jia L: Payment methods for outpatient care facilities. *Cochrane Database of Systematic Reviews* 2017(3). - Young C, Hall AM, Gonçalves‐Bradley DC, Quinn TJ, Hooft L, van Munster BC, Stott DJ: Home or foster home care versus institutional long‐term care for functionally dependent older people. *Cochrane Database of Systematic Reviews* 2017(4). - Xie HY, Feng D, Wei DM, Mei L, Chen H, Wang X, Fang F: Probiotics for vulvovaginal candidiasis in non‐pregnant women. *Cochrane Database of Systematic Reviews* 2017(11). - Wiysonge CS, Paulsen E, Lewin S, Ciapponi A, Herrera CA, Opiyo N, Pantoja T, Rada G, Oxman AD: Financial arrangements for health systems in low‐income countries: an overview of systematic reviews. *Cochrane Database of Systematic Reviews* 2017(9). - Wiysonge CS, Ntsekhe M, Thabane L, Volmink J, Majombozi D, Gumedze F, Pandie S, Mayosi BM: Interventions for treating tuberculous pericarditis. *Cochrane Database of Systematic Reviews* 2017(9). - Wiysonge CS, Bradley HA, Volmink J, Mayosi BM, Opie LH: Beta‐blockers for hypertension. *Cochrane Database of Systematic Reviews* 2017(1). - Wijedoru L, Mallett S, Parry CM: Rapid diagnostic tests for typhoid and paratyphoid (enteric) fever. *Cochrane Database of Systematic Reviews* 2017(5). - Wiffen PJ, Derry S, Moore RA, McNicol ED, Bell RF, Carr DB, McIntyre M, Wee B: Oral paracetamol (acetaminophen) for cancer pain. *Cochrane Database of Systematic Reviews* 2017(7). - Wiffen PJ, Derry S, Bell RF, Rice ASC, Tölle TR, Phillips T, Moore RA: Gabapentin for chronic neuropathic pain in adults. *Cochrane Database of Systematic Reviews* 2017(6). - Whitelaw A, Lee‐Kelland R: Repeated lumbar or ventricular punctures in newborns with intraventricular haemorrhage. *Cochrane Database of Systematic Reviews* 2017(4). - Westby MJ, Dumville JC, Soares MO, Stubbs N, Norman G: Dressings and topical agents for treating pressure ulcers. *Cochrane Database of Systematic Reviews* 2017(6). - Weibel S, Jelting Y, Afshari A, Pace NL, Eberhart LHJ, Jokinen J, Artmann T, Kranke P: Patient‐controlled analgesia with remifentanil versus alternative parenteral methods for pain management in labour. *Cochrane Database of Systematic Reviews* 2017(4). - Webster AC, Wu S, Tallapragada K, Park MY, Chapman JR, Carr SJ: Polyclonal and monoclonal antibodies for treating acute rejection episodes in kidney transplant recipients. *Cochrane Database of Systematic Reviews* 2017(7). - Wang HT, Yuan JQ, Zhang B, Dong ML, Mao C, Hu D: Phototherapy for treating foot ulcers in people with diabetes. *Cochrane Database of Systematic Reviews* 2017(6). - Walters JAE, Tang JNQ, Poole P, Wood‐Baker R: Pneumococcal vaccines for preventing pneumonia in chronic obstructive pulmonary disease. *Cochrane Database of Systematic Reviews* 2017(1). - Wall BF, Magee K, Campbell SG, Zed PJ: Capnography versus standard monitoring for emergency department procedural sedation and analgesia. *Cochrane Database of Systematic Reviews* 2017(3). - Walker RM, Gillespie BM, Thalib L, Higgins NS, Whitty JA: Foam dressings for treating pressure ulcers. *Cochrane Database of Systematic Reviews* 2017(10). - Wagner AD, Syn NLX, Moehler M, Grothe W, Yong WP, Tai BC, Ho J, Unverzagt S: Chemotherapy for advanced gastric cancer. *Cochrane Database of Systematic Reviews* 2017(8). - Vogel N, Schandelmaier S, Zumbrunn T, Ebrahim S, de Boer WEL, Busse JW, Kunz R: Return‐to‐work coordination programmes for improving return to work in workers on sick leave. *Cochrane Database of Systematic Reviews* 2017(3). - Verbeeck W, Bekkering GE, Van den Noortgate W, Kramers C: Bupropion for attention deficit hyperactivity disorder (ADHD) in adults. *Cochrane Database of Sy
[truncated: 820,683 more chars]
